# No Evidence of Unexpected Transgenic Insertions in T1190 – A Transgenic Apple Used in Rapid Cycle Breeding – Following Whole Genome Sequencing

**DOI:** 10.3389/fpls.2021.715737

**Published:** 2021-08-11

**Authors:** Andrea Patocchi, Jens Keilwagen, Thomas Berner, Stefanie Wenzel, Giovanni A. L. Broggini, Lothar Altschmied, Magda-Viola Hanke, Henryk Flachowsky

**Affiliations:** ^1^Research Division Plant Breeding, Agroscope, Wädenswil, Switzerland; ^2^Julius Kühn-Institut (JKI), Federal Research Centre for Cultivated Plants, Institute for Biosafety in Plant Biotechnology, Quedlinburg, Germany; ^3^Julius Kühn-Institut (JKI), Federal Research Centre for Cultivated Plants, Institute for Breeding Research on Fruit Crops, Dresden, Germany; ^4^Swiss Federal Institute of Technology, Molecular Plant Breeding, Institute of Agricultural Sciences, ETH Zurich, Zurich, Switzerland; ^5^Leibniz-Institut für Pflanzengenetik und Kulturpflanzenforschung (IPK), Gatersleben, Germany

**Keywords:** *Malus*× *domestica* (Borkh), fast breeding, *BpMADS4*, juvenility, null segregant, new plant breeding techniques, whole genome sequencing

## Abstract

Rapid cycle breeding uses transgenic early flowering plants as crossbreed parents to facilitate the shortening of breeding programs for perennial crops with long-lasting juvenility. Rapid cycle breeding in apple was established using the transgenic genotype T1190 expressing the *BpMADS4* gene of silver birch. In this study, the genomes of T1190 and its non-transgenic wild-type PinS (F1-offspring of ‘Pinova’ and ‘Idared’) were sequenced by Illumina short-read sequencing in two separate experiments resulting in a mean sequencing depth of 182× for T1190 and 167× for PinS. The sequencing revealed 8,450 reads, which contain sequences of ≥20 bp identical to the plant transformation vector. These reads were assembled into 125 contigs, which were examined to see whether they contained transgenic insertions or if they are not using a five-step procedure. The sequence of one contig represents the known T-DNA insertion on chromosome 4 of T1190. The sequences of the remaining contigs were either equally present in T1190 and PinS, their part with sequence identity to the vector was equally present in apple reference genomes, or they seem to result from endophytic contaminations rather than from additional transgenic insertions. Therefore, we conclude that the transgenic apple plant T1190 contains only one transgenic insertion, located on chromosome 4, and shows no further partial insertions of the transformation vector.

Accession Numbers: JQ974028.1.

## Introduction

Rapid cycle breeding, also called accelerated breeding, is one of the “New Plant Breeding Techniques,” which is based on the use of transgenic early-flowering plants as crossbreed parents to shorten breeding cycles (Lusser et al., 2012). The concept is that when a plant with a single copy integration of a cassette containing a transgene for early flowering is crossed with another plant that possesses a monogenic trait of interest (e.g., disease resistance trait); about one-quarter of the resulting F1 plants will contain both the early flowering gene and the trait of interest (Flachowsky et al., 2009). Such transgenic plants are selected in a breeding program aiming to introgress desired traits from closely related wild species. Since early flowering is induced, these plants have a shortened juvenile period and can rapidly be used in modified (pseudo-) backcrosses to reduce the remaining portion of the wild plant genome. After four to five cycles of modified backcrossing, non-transgenic null segregants containing only the trait of interest, and not the transgenic cassette, are selected. The subsequently selected individual is an advanced selection, which is free of any unwanted transgenic DNA resulting from the transformation process, but which was produced in much less time than that required by traditional breeding. Therefore, rapid cycle breeding offers new horizons especially in tree breeding of species with a long juvenility phase (up to 10 years) such as apple, mango, or date palm (Hanke et al., 2012). For such species, breeding programs usually take15–25 years if the F1 progeny contains the desired trait required in a new cultivar being developed (Fenning and Gershenzon, 2002; Lespinasse, 2009; Sansavini and Tartarini, 2013). In introgression breeding programs, where numerous (pseudo)-backcross cycles are required, it can take up to several decades (Bakker et al., 1999).

For apple (*Malus domestica* Borkh.), the first rapid cycle breeding approach was established about 10 years ago (Flachowsky et al., 2009). PinS, an F1 offspring from a cross between German apple cultivars ‘Pinova’ and ‘Idared’ (Luo et al., 2019), was transformed *via Agrobacterium-mediated* gene transfer with the *BpMADS4* transcription factor gene of silver birch *Betula pendula* Roth (Flachowsky et al., 2007). *BpMADS4* transgenic lines of PinS started flowering already during *in vitro* cultivation and/or a few weeks following the transfer to the greenhouse. The transgenic event T1190 was selected for breeding. T1190 contains a single copy of the early flowering-inducing cassette on linkage group 4 of the apple genome (Flachowsky et al., 2011). The exact position of the transgenic cassette was detected by isolating the T-DNA flanking regions of T1190, reconstructing the genomic site of integration in PinS, and comparing this sequence with the apple whole genome sequence v1.1 (Daccord et al., 2017). The transgenic cassette was found to be integrated at position 25,457,909-25,457,921, corresponding to an approximate genetic position of 41.25 cM on chromosome 4 (Luo et al., 2019). The integration of the cassette resulted in a loss of 11 bp of the apple genome directly at the site of integration (Flachowsky et al., 2011). Consequently, T1190 corresponds to the insert sequence knowledge (ISK) class 1 scenario (Holst-Jensen et al., 2013), which describes genetically modified organisms (GMOs) for which both the complete insert and flanking DNA sequences are known.

T1190 is characterized by an early flowering phenotype combined with a tree size and habit, which enables the tree to bear a sufficient number of fruits and seeds. A proof-of-concept experiment was initiated using T1190 as maternal parent (Flachowsky et al., 2009, 2011) to introgress resistance to fire blight caused by *Erwinia amylovora* (Burr. Winslow et al., 1920) from the wild apple genotype *Malus fusca* (Raf.) C.K. Schneid. MAL0045 (Emeriewen et al., 2020). Transgenic early flowering F1 seedlings were identified and crossed with donors of other resistance genes and/or QTL; for instance, *Venturia inaequalis* (Cooke) G. Winter, 1875 (apple scab), *Podosphaera leucotricha* (Ellis and Everh.) Salmon (powdery mildew), and Erwinia amylovora (fire blight). Flachowsky et al. (2011) demonstrated that a generation time of less than a year could be achieved using T1190. This is significantly faster compared with classical apple breeding, where one generation cycle (seed-to-seed) takes 3–5 years or even more (Fischer, 1994). Le Roux et al. (2012) further improved the rapid cycle breeding system using a cross between the T1190 line and the ornamental apple ‘Evereste’, which carries on linkage group 12 the fire blight resistance locus *Fb_E* (Durel et al., 2009; Parravicini et al., 2011). The F1 seedlings were crossed again with different elite apple cultivars. Twenty-four BC’1 seedlings were screened for background selection to estimate the remaining genome portion of ‘Evereste’ using a set of simple sequence repeat (SSR) markers evenly distributed across the apple genome. Two BC’1 seedlings carrying less than 15% of the genome of ‘Evereste’ were identified. Schlathölter et al. (2018) furthered the study and established seven advanced selections of the fourth (BC’3) and 11th generation (BC’4) within 7 years. The null segregants obtained in Schlathölter et al. (2018) possessed a regular habit and a high level of fire blight resistance. Eight of the 18 null segregants were shown to still contain on linkage group 12 only 4% of the ‘Evereste’ genome associated with the resistance gene *FB_E* (genetic drag). Recently, Luo et al. (2020) reported the start of the introgression of blue mold resistance from *Malus sieversii* PI 613981 into elite apple germplasm using T1190 for rapid cycle breeding.

Furthermore, T1190 was used to pyramid and combine fire blight and scab resistance from apple cultivars ‘Splendor’ and ‘Enterprise’ into a single individual within the RosBREED project^1^ (Iezzoni and Peace, 2019). This individual, which is now available as a disease-resistant donor parent as null segregant obtained by the rapid cycle breeding approach, can be freely planted in the United States without any restriction (Callahan et al., 2016).

In Europe, however, there is no explicit decision on whether or not null segregants of the rapid cycle breeding approach fall under the existing regulations of genetically modified organisms. European apple breeders fear a competitive disadvantage if these genotypes are not deregulated (Laurens et al., 2018). However, before any legal classification is expected, it must be shown that no additional and unexpected transgenic insertions are inherited by T1190. Various protocols based on next-generation sequencing technologies have already been established in *Arabidopsis* (Schouten et al., 2017), rice (Yang et al., 2013; Park et al., 2015), and poplar (Kersten et al., 2020) to demonstrate that GMO plants contain the T-DNA insertion at the known location in the genome, and that there are no additional insertions of T-DNAs (not detected by PCR and Southern hybridization before) or short T-DNA fragments (splinters). Illumina paired-end short read sequencing has been shown to be an effective method for the detection of unintended transgenic insertions. Yang et al. (2013) defined five types of sequencing reads for this purpose. In type A reads, both paired-ends perfectly map back to the host genome. In type B reads, one end matches to the host genome, whereas the other end matches to the transgene. In reads of type C, both paired-ends match to the transgene and in reads of types D and E, one end matches to the host genome or the transgene, whereas the other end spans the junction region between the host genome and the transgene (Yang et al., 2013). Accordingly, reads of classes B, C, D, and E are to be considered as transgenic insertions.

In this study, whole genome sequencing of T1190 and its non-transgenic wild-type PinS was performed to verify if short sequences (≥20 bp) of the plant transformation vector pHTT602-CaMV35S::*BpMADS4* used to generate the T1190 line are present in regions of its genome other than the known insertion on chromosome 4 of T1190. The 20 bp was used as a selection criterion on the basis of EFSA’s “20 bp rule” (EFSA Panel on Genetically Modified Organisms, 2012). This selection criterion is often used in practice to decide whether or not a sequence is regarded as a new combination of genetic material, although the 20 bp criterion does not have a scientifically sound base (Whelan and Lema, 2015).

## Results and Discussion

### Re-sequencing of the Transformation Vector

The 10,699 bp plant transformation vector pHTT602-CaMV35S::*BpMADS4*, which was used to produce the transgenic apple line T1190, was re-sequenced with 4× coverage. The sequence following additional Sanger sequencing of single regions, for which the coverage was less than 3× after shotgun DNA-Sanger-sequencing, was assembled. The resulting sequence was fully identical to the sequence expected based on the cloning procedure described by Elo et al. (2001). The sequence was submitted to the gene bank of the National Center for Biotechnology Information (NCBI, GenBank: JQ974028.1).

### Re-sequencing of PinS and T1190

Whole genome sequencing of genomic DNA isolated from plants grown in the greenhouse (experiment 1) resulted in ∼310 Mio. raw reads and 280 Mio. trimmed reads per genotype (Table 1). Each data set (PinS and T1190) corresponds to a 44×-fold theoretical mean coverage of the GDDH13 reference genome sequence. Mapping of each data set against the GDDH13 genome sequence resulted in a sequence coverage of ∼86% (∼98% without stretches of “N”) of the reference genome.

**TABLE 1:** Whole genome sequencing results.

Numbers	Experiment 1^1^		Experiment 2^2^		Total^5^	
	T1190	PinS	T1190	PinS	T1190	PinS
Raw reads	308,925,214	310,739,352	649,641,728	576,343,434	958,566,942	887,082,786
Trimmed reads	276,761,471	279,241,264	625,550,494	546,185,778	902,311,965	825,427,042
Sequence covered in bp	609,594,873	610,245,201	612,392,244	612,143,045	613,552,632	613,464,262
Reference covered (%)^3^	85.91	86.00	86.31	86.27	86.47	86.46
Reference covered no “N” (%)^4^	97.56	97.66	98.01	97.97	98.19	98.18
Mean coverage (x-fold)^3^	44	44	138	123	182	167
N° of reads with sequence identity to the vector^6^	2,238	–	6,212	–	8,450	–
N° of fragments with sequence identity to the vector^6^	1,879	–	5,273	–	7,152	–
Contigs					125	–
Mapped reads					15,270,575	11,142,536

*^1^Genomic DNA isolated from plants grown in the greenhouse. ^2^Genomic DNA isolated from plants grown in vitro. ^3^The GDDH13 genome sequence of the apple cultivar ‘Golden Delicious’ published by Daccord et al. (2017) was used as a reference for comparison. The genome size used for comparison is 709,561,391 bp. ^4^The GDDH13 genome sequence excluding stretches of “N” was used as a reference for comparison. The genome size used for this comparison is 624,851,160 bp. ^5^Summarized results of experiments 1 and 2. ^6^N° of reads/fragments containing reads with at least one 20-mer with identity to the vector sequence and a Phred quality score of 20 (99% base call accuracy).*

Whole genome sequencing of genomic DNA isolated from plantlets grown *in vitro* (experiment 2) resulted in ∼650 Mio. raw reads (∼626 Mio. trimmed reads) for T1190 and ∼626 Mio. raw reads (∼546 Mio. trimmed reads) for PinS (Table 1). The sequence data correspond to a 138 × -fold (T1190) and 123 × -fold (PinS) theoretical mean coverage of the GDDH13 reference genome sequence. Mapping against the GDDH13 genome sequence resulted in a sequence coverage of ∼86% (∼98% without stretches of “N”) of the reference genome for each data set.

Merging the sequencing data of experiments 1 and 2 resulted in ∼959 Mio. raw reads (∼902 Mio. trimmed reads) for T1190 and ∼887 Mio. raw reads (∼825 Mio. trimmed reads) for PinS (Table 1). The sequence data correspond to a 182 × -fold (T1190) and 167 × -fold (PinS) theoretical mean coverage of the GDDH13 reference genome sequence. Mapping against the GDDH13 genome sequence resulted in a slightly increased sequence coverage of 87% (∼98% without stretches of “N”) of the reference genome for each data set.

The sequencing depth is expected to be sufficient for detecting unexpected insertions of transgenic DNA in T1190. Other studies succeeded with sequencing depths of 24× to 72× when investigating transgenic rice (Yang et al., 2013; Park et al., 2015). In transgenic *Arabidopsis*, 25× to 49×, was sufficient (Schouten et al., 2017) using a very similar approach. Furthermore, it has been shown that an increase in the sequencing depth to three times (experiment 2 compared with experiment 1) did not result in significant higher genome coverage (Table 1). For this reason, a further increase in the sequencing depth does not appear to be expedient.

### Identification of Contigs With Vector Identity

**FIGURE 1:**
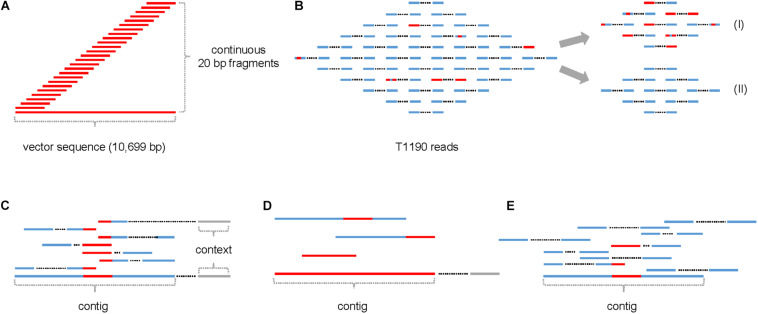
Schematic of the procedure for identifying small insertions of foreign DNA into the genome of T1190. **(A)** *In silico* generation of continuous 20-mers that cover the entire sequence of the plant transformation vector; **(B)** read pool of T1190 containing reads with (red) and without (blue) ≥20 bp sequence identity to the plant transformation vector, reads with (I) and without (II) were separated from each other; **(C)** assembly of reads with vector identity to contigs; **(D)** examples for contigs containing fragments of ≥20 bp with sequence identity to the plant transformation vector; **(E)** mapping of contigs against the read pools of PinS and T1190. Context (gray): sequence information originating from the paired end of a read that maps to one of the two ends of a contig.

All continuous 20-mers from the plant transformation vector pHTT602-CaMV35S::*BpMADS4* were extracted *in silico* (Figure 1A). These fragments were used to screen the ∼902 million trimmed reads of T1190 (Figure 1B and Table 1) to identify those which contain at least one 20-mer with sequence identity to the vector sequence and a Phred quality score of ≥20 (≥99% base call accuracy).

**FIGURE 2:**
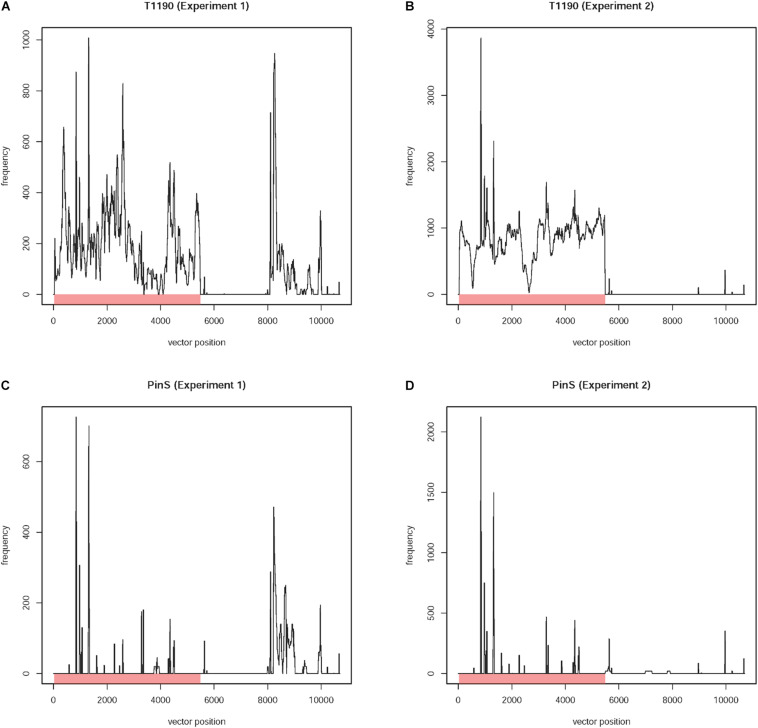
Frequency of occurrence of 20-mers with identity to the plant transformation vector present in the four sequencing data sets (experiments 1 and 2) of T1190 and PinS, respectively. Plots for the sequence data of T1190 in experiments **(A)** 1 and **(B)** 2 as well as the control genotype PinS in experiment **(C)** 1 and **(D)** 2. The *Y*-axis provides information on how often a 20-mer, which is identical to the transformation vector pHTT602-CaMV35S::BpMADS4 (total length 10,699 bp) starting at the position listed on the *X*-axis, occurs in the sequence data set. The region between positions 8 and 5,726 bp (red bar) represents the T-DNA including the right border (position 8 bp to 32 bp) and left border (positions 5,703 bp to 5,726 bp) motifs. The remaining sequence is vector backbone. The part of the T-DNA, which is integrated into the genome of T1190 as published by Flachowsky et al. (2011), is between positions 29 and 5,484 bp.

Based on this approach, 8,450 reads were identified. These reads belonged to 7,152 paired-end DNA fragments. Mapping of these reads against the plant transformation vector sequence revealed large differences between the data sets obtained from the two experiments (Figures 2A,B).

High sequence coverage was found for the first ∼6 kbp of the vector sequence using the reads of T1190 of experiments 1 and 2 separately. Using the reads of experiment 1, an additional region with high sequence coverage was found. This region, which is located at the vector backbone between ∼8 and ∼10 kbp (Figure 2A), was not found using the data of experiment 2 (Figure 2B), even though the sequencing depth was threefold higher in this experiment. The same procedure was applied using the sequencing data of PinS. A comparable sequence coverage was found for this vector backbone region using the sequencing data of experiment 1 (Figure 2C). Again, this region was not detectable using the data of experiment 2 (Figure 2D).

An insertion of this region (or fragments of it) in the genome of T1190 is not plausible, because (i) a comparable sequence coverage is detectable in the non-transformed genotype PinS in experiment 1 and (ii) the sequence coverage is lacking in T1190 and PinS in experiment 2 (Figures 2A–D). Sequence blast analysis revealed that this region of the vector backbone contains the beta-lactamase encoding *bla* gene and the pBR322 replication origin. These sequences are also present in different bacterial species, of which some are known to be endophytes. The occurrence of sequence coverage for this region in experiment 1 may be a consequence of bacterial contaminations originating from those endophytes. It was shown that a large variety of endophytic bacteria and fungi can be detected in greenhouse plants of apple (Liu et al., 2018).

### Evaluation of 125 Contigs for Transgenic Insertions

The 8,450 reads were assembled *de novo* into 125 contigs (Figures 1C,D). The length of fragments with vector identity ranged between 21 bp (e.g., contigs 5, 9, and 11) and 5,457 bp for contig 0, the latter corresponding to the known T-DNA insertion in T1190 (Supporting Information 1).

All trimmed reads of T1190 and PinS of both experiments were mapped against the 125 contigs (Figure 1E). Seventy-eight contigs were found to be present in the sequence data of both experiments. Thirty-four contigs were only present in the data of experiment 1, whereas only 13 contigs were found in experiment 2.

A six-step procedure was established to evaluate the 125 contigs with a sequence identity of ≥20 bp to the transformation vector for transgenic insertions. First, the contig representing the known T-DNA integration on chromosome 4 was identified and excluded from further investigation. Second, all contigs, which are equally present in T1190 and PinS, were also excluded from further investigation, because the sequences were obviously not introduced in T1190 with the transformation procedure but were already present in PinS. Third, all contigs missing in PinS but found in apple reference genome were also not considered further, as these contigs were only found because of sequencing biases or assembly errors. Fourth, underrepresented contigs (consisting of one or two reads only) were removed from the data set. Such contigs seem to result from sequencing errors or contaminations rather than transgenic insertions. Fifth, the origin of the remaining contigs was investigated by *in silico* sequence analyses (e.g., blast searches in different databases).

When the analyses of contigs suggested unexpected transgenic insertion, their presence in the genome of T1190 and PinS was tested by polymerase chain reaction (PCR).

#### Step 1: Exclude the Contig That Represents the Known T-DNA Insertion

**FIGURE 3:**
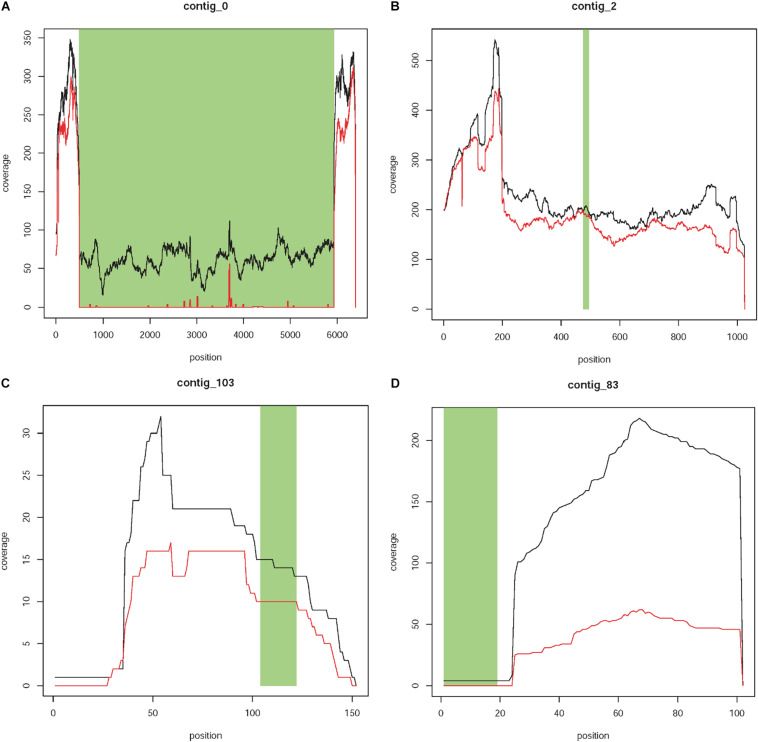
Coverage plots of different types of contigs. **(A)** Contig 0 represents the known T-DNA integration on linkage group 4 of T1190. This contig is characterized by a region with 100% sequence identity to the T-DNA of the plant transformation vector (green area), which is present in T1190 (black line) but missing in PinS (red line). Furthermore, the contig is flanked by two sequences (white areas left and right), which show 100% sequence identity to sequences on chromosome 4 of the apple genome, which are located very close to each other. **(B,C)** Contigs with sequence identity to the vector sequence (green section) that are present in both PinS and T1190. **(B)** Contig 2 represents an example for contigs, which are present over the whole length of the contig in both genotypes (Type 1 contigs). **(C)** Contig 103 represents an example for contigs containing small parts, which are present in PinS or T1190 only, but the region with vector identity is present in both genotypes and show a similar sequence coverage (type 2 contigs). **(D)** Contig 83 shows an example for contigs containing at least ≥20 bp, which are present in T1190 with low coverage but are absent in PinS (green section). The *y*-axis represents the coverage of a sequence region along the contig (*x*-axis).

Contig 0 has a size of 6,382 bp, of which 5,457 (positions 484 to 5,940) showed 100% sequence identity to the T-DNA of the plant transformation vector between positions 29 and 5,484 (Figure 3A). The contig represents the known T-DNA insertion on chromosome 4. The first 484 bp of contig 0 showed 100% (484/484 bp) sequence identity to a sequence located on chromosome 4 (position 25,457,921 to 25,458,404) of the GDDH13 V1.1 reference genome, which contains the 393 bpT-DNA right border flanking sequence described by Flachowsky et al. (2011). The 484 bp also showed a high level of sequence identity (92.2%, 449/487 bp) to a region on chromosome 12 (position 25,684,575 to 25,685,061). Comparable results were obtained for the last 442 bp of contig 0. This sequence showed 100% (442/442 bp) to a sequence located on chromosome 4 (positions 25,457,465 to 25,457,906) representing the T-DNA left boarder flanking sequence identified by Flachowsky et al. (2011). High level of sequence identity (94.34%, 417/442bp) to a region located on chromosome 12 (positions 25,684,119 to 25,684,560) was also found. The high levels of sequence identity between chromosomes 4 and 12 are not surprising, since Velasco et al. (2010) and Daccord et al. (2017) showed collinearity between the segments of both chromosomes. The existing collinearity is the reason for the high genome coverage of the two flanking regions of contig 0 (Figure 3A). Whereas these sequences are present in both homologs of each chromosome (4 and 12), the remaining sequence of the contig, which represents the T-DNA insertion, is only present in one homolog of chromosome 4. This explains the approximately four times less sequence coverage.

The segment of chromosome 4 containing the transgenic insertion will only be present in plants, which are used as parents during the rapid cycle breeding approach, i.e., expressing the early flowering gene. Mendelian segregation eliminates this insertion in the final cross. Thus, the final product of the rapid cycle breeding approach does not contain this sequence (Flachowsky et al., 2009). Therefore, contig 0 was not analyzed further.

#### Step 2: Exclusion of Contigs With Similar Coverage in Both Genotypes

Coverage plots of the remaining 124 contigs were also established for each genotype (Supporting Informations 1,2). A comparison of these plots resulted in contigs with vector identity that show similar coverage in both genotypes and those that are present (at least in part) in T1190 only. Seventeen contigs are present only in T1190, while 107 are present in T1190 and PinS. These 107 contigs were assigned to two different types. For type 1 contigs, the coverage across the whole contig is similar for both PinS and T1190 (Figure 3B), whereas type 2 contigs contain small-sequence parts, which are present in PinS or T1190 only (Figure 3C). However, the region with vector identity of both types is present in both apple genotypes and shows a similar coverage. Therefore, the 107 contigs were not analyzed further.

The 17 contigs (37, 80, 81, 83, 84, 86, 92, 94, 96, 98, 99, 100, 104, 109, 112, 118, and 119) that contain at least 20 bp with vector identity and are present in T1190 only (Figure 3D) were further investigated.

#### Step 3: Exclusion of Contigs That Show No or Extremely Low Coverage in PinS but Are Also Present in Other Apple Reference Genome Sequences

Five of the 17 contigs (81, 92, 98, 112, and 119) were selected, because their sequences with vector identity were present in T1190, while the corresponding sequence was not covered in PinS. The sequences of these contigs were blasted to the available apple reference genomes of ‘Golden Delicious’ (Velasco et al., 2010), GDDH13 v1.1 (Daccord et al., 2017) and ‘Gala Galaxy’ (Broggini et al., 2020). The sequences with identity to the plant transformation vector of these five contigs sequences also showed homology to the apple reference genomes (Supporting Information 3).

**FIGURE 4:**
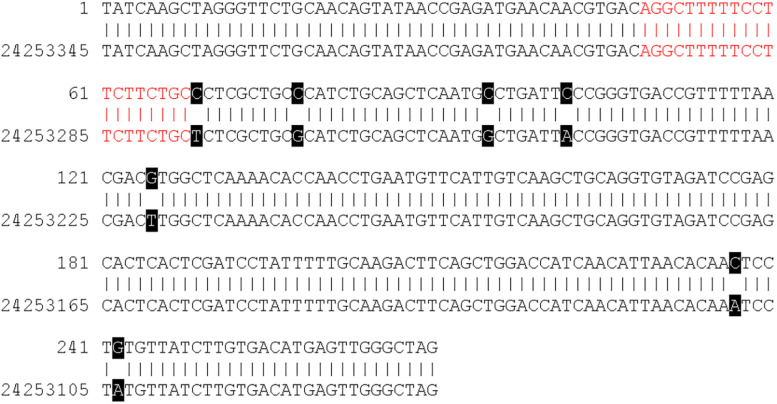
DNA sequence alignment of contig 112 (C_112) and regions 24,253,076 to 24,253,345 on chromosome 16 of the GDDH13v1.1 genome sequence. Black boxes indicate single nucleotide polymorphism (SNP) between the contig sequence and the genome sequence of GDDH13v1.1. The region with sequence identity to the plant transformation vector is indicated in red.

Figure 4 shows a DNA sequence alignment of contig 112 and a region on chromosome 16 of the GDDH13v1.1 genome sequence as an example. Contig 112 was assembled from two reads that originated following experiment 1 sequencing. The contig has a length of 270 bp and contains a 20-mer, which is identical to the plant transformation vector. The sequence of contig 112 shows a sequence identity of 97.41% (263/270 bp) to the region on chromosome 16 of GDDH13v1.1. The 20-mer with vector identity of contig 112 (highlighted in red in Figure 4) is identically present in this region of GDDH13v1.1.

#### Step 4: Removal of Contigs Underrepresented in Sequencing Data

Seven of the remaining 12 contigs (94, 96, 99, 100, 104, 109, and 118) are underrepresented in the data sets. They were only detectable in one of the two experiments. Six contigs were only present in sequencing experiment 1, whereas contig 104 was only detectable in experiment 2. Each of the seven contigs consisted of only one or two reads. In the first step, the contigs were blasted to the available apple reference genomes of ‘Golden Delicious’ (Velasco et al., 2010), GDDH13 v1.1 (Daccord et al., 2017) and ‘Gala Galaxy’ (Broggini et al., 2020). No similar sequences could be identified. Five contigs were without context. Context information was only available for contigs 99 (one sequence) and 104 (one sequence). These context sequences were also blasted against the three available genome sequences, but no identity to apple was found. These sequence analyses strongly affirmed that none of these seven contigs possessed any significant sequence identity to apple.

Subsequently, these contigs were blasted against the entire NCBI database. All the seven contigs showed different levels of sequence identity to the vector sequence, and to different bacterial sequences (Supporting Information 4). The levels of sequence identity to other bacterial, fungal, or amoeboid sequences were higher for contigs 96, 99, 100, 104, 109, and 118 compared with the level of sequence identity between these contigs and the vector sequence. These six contigs seem to originate from contaminations rather than insertions of transgenic DNA into the apple genome.

The origin of contig 94 is unclear. Ninety-six out of the 99bp of this contig showed 100% sequence identity to the sequence of the plant transformation vector. A level of sequence identity same with that of several other bacterial sequences was found. The two reads representing this contig could have originated from the plant transformation vector or bacterial contaminations. However, no sequence identity to apple was found.

None of the seven contigs provided clear evidence for an additional transgenic insertion and could not be assigned to any of the five sequence types defined by Yang et al. (2013), which are expected for GM events. In addition, the contigs did not consist of split reads, nor did they contain splinter sequences, which are known in *Arabidopsis* (Schouten et al., 2017). The number of reads per contig is too low compared with the mean sequencing depth of 182×. None of these contigs showed any sequence identity to apple, and their sequence identity to the plant transformation vector sequence is equal (contig 94) or lower (contigs 96, 99, 100, 104, 109, and 118) compared with other bacterial, fungal or amoeboid sequences.

#### Step 5: Assessment of the Remaining Contigs

The remaining five contigs (37, 80, 83, 84, and 86) were only detectable in sequencing experiment 1.

**FIGURE 5:**
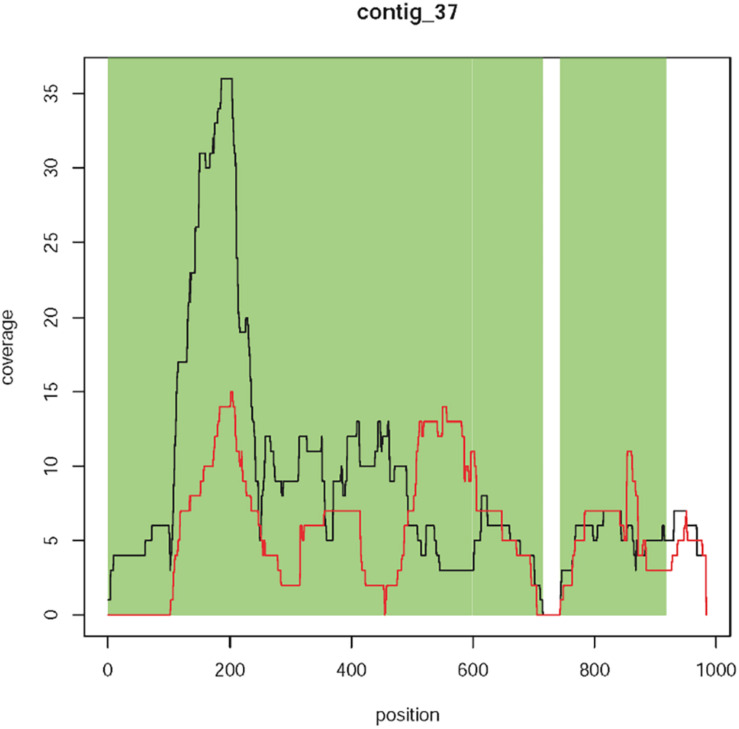
Coverage plot of contig 37. Sequence coverage along the contig is variable, but comparably low. Black line, T1190; red line, PinS.

Contig 37 has a size of 1,119 bp and is represented by 122 reads. This contig is equally present in T1190 and PinS except for the first ∼100 bp, which were only found in T1190 (Figure 5). From bases 1 to 714, the contig shows sequence identities of 99% (713/714 bp) to the plant transformation vector and 100% (714/714 bp) to other bacterial sequences (e.g., *Staphylococcus cohnii*, *Eschericha coli*). The first 100 bp of contig 37 are identical to the vector sequence and to sequences of several bacterial species. A complete sequence identity (100%, 176/176 bp) to the vector sequence and the bacterial sequences was also found for the region between 744 bp to 919 bp.

Four context sequences are available (two on each side) for contig 37 (Supporting Information 1). The sequence identity between these context sequences and the vector sequence ranged between 65 and 78.2%. Three context sequences showed much higher sequence identities of 94 to 100% to the ColE1 replication origin of several bacterial species (including *E. coli*). However, one context sequence showed a sequence identity of 65% to the plant transformation vector and a slightly lower identity of 61% to the ColE1 origin of several bacterial species. The sequence identity to the vector does not come about through a coherent piece of sequence but through several short sequence segments. Although these small segments have sequence identity to the vector sequence, the sequence identity of the entire sequence is not sufficient to speak of the same sequence. It is not homology per se but rather sequence similarity. It should be noted that the individual segments with vector identity were all very short (only a few bp) and interrupted by the segments without vector identity.

No sequence identity was found when the contig sequence (including the first 100 bp) or the context sequences were blasted against the apple reference genome sequences. Because of the lack of sequence identity to apple, the comparably low and variable sequence coverage (∼10×), the high concordance with sequences of different bacterial species, and the lack of this contig in sequencing experiment 2, for this contig there is no evidence for an additional insertion. It is more likely that this contig originated from bacterial contamination.

Contig 80 has a size of 284bp and is represented by eight reads. The contig shows 100% (284/284 bp) sequence identity to the vector sequence. No context sequence is available. The contig also shows 100% sequence identity to the broad-spectrum beta-lactamase TEM-116 gene of different bacteria and fungi. No sequence identity with apple exists. The origin of contig 80 is not fully clear. The contig could have originated from the plant transformation vector or microbial contaminations. Nevertheless, there was no evidence of an additional insertion, since this contig does not show any sequence identity to apple, and it was observed only in experiment 1.

Contig 83 has a size of 101 bp and is represented by eight reads. The first 20 bp are identical to the vector sequence. One context sequence of 100 bp is available, which is connected to the right end. No sequence identity with apple exists. The context sequence does not show any sequence identity to the vector sequence. Both sequences (contig and context) were combined and blasted against the three available apple reference genomes. No sequence identity to apple was found.

Subsequently, a BLASTn search against the entire NCBI database was performed. The contig sequence (starting from base 25) and the context sequence showed a sequence identity of 100% to a genome assembly of *Staphylococcus haemolyticus* (Sequence ID: LT963441.1). *S. haemolyticus* occurs in the saprophytic skin flora of humans. *Staphylococcus* species have been described several times in connection with bacterial contaminations in apple especially on plant surfaces (Fatema et al., 2013) as well as on *in vitro* cultures (Flachowsky and Hanke, 2009).

Contig 83 originated most likely from bacterial contaminations. The contig does not provide evidence for transgenic insertion. The contig is represented by only few reads, detectable only in one sequencing experiment, lacks any sequence identity to apple, and shows its highest level of sequence identity to other bacterial sequences.

**FIGURE 6:**
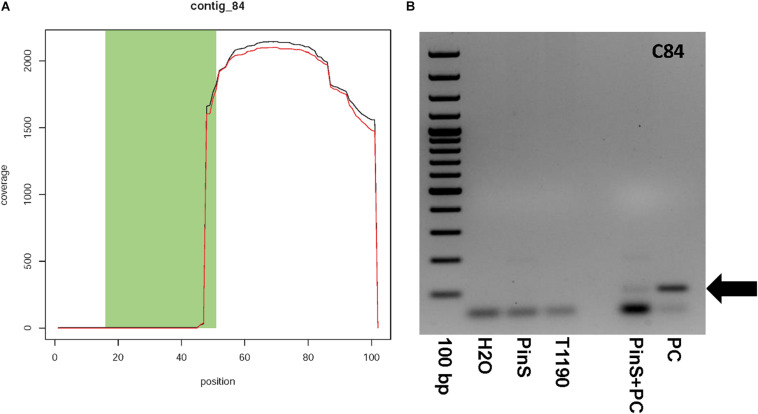
Coverage plot of **(A)** contig 84 and **(B)** verification of the existence of contig 84 by PCR. Contig 84 could not be detected in both PinS and T1190. 100 bp, DNA size standard GeneRuler^TM^ 100 bp Plus DNA Ladder (Thermo Fisher Scientific, Erlangen, Germany); H_2_O – water used as PCR-negative control, PinS – genomic DNA of the non-transformed genotype PinS, T1190 – genomic DNA of the transgenic genotype T1190, PinS + PC – genomic DNA of the non-transformed genotype PinS supplemented with DNA of the plasmid vector pEX-A128 (Eurofins Scientific, Hamburg, Germany) containing the sequence of contig 84, PC – plasmid DNA of pEX-A128 containing the sequence of contig 84 used as positive control. The black arrows mark the expected fragment.

Contig 84 has a size of 101 bp and contains one context sequence of 98 bp on its right end. Fifty-two base pairs of the first 53 bp of contig 84 are identical to the sequence of the plant transformation vector (Figure 6A). The contig is represented by only six reads (Supporting Information 1). If blasted against all sequencing data, then more than 2,000 reads map to this contig, but only in the region where this contig shows no match to the vector DNA (Figure 6A). A BLASTn search using a combined sequence of contig and context resulted in a sequence identity of 91% (138/152bp) to a *M. domestica* retrotransposon Ty1-copia element (GenBank: FJ705356.1). This Ty1-copia element is frequently present on all apple chromosomes. Contig 84 could have originated from a transgenic insertion, as part of this contig shows sequence identity to the plant transformation vector, whereas another part shows identity to apple. The large difference in the coverage of the two parts of contig 84 makes it questionable whether this contig is an additional insertion of transgenic DNA. A PCR analysis was performed to ascertain the existence of contig 84 in the genome of T1190. An amplicon of the size of the putative transgenic insertion (100bp) could be detected in control reactions with the synthetic template, but not in T1190or PinS (Figure 6B). The PCR was repeated several times using different DNA samples, but the result was always the same. We, therefore, concluded that this chimeric sequence is an artifact and not an insertion of a part of the vector.

Contig 86 has a size of 112 bp and consists of six reads all found in T1190. Sequence identity to the plant transformation vector was found within the first 84 bp. The longest fragment with a sequence identity of 100% had a size of 43 bp. Neither the contig nor the context sequence (101 bp) showed any sequence identity to apple. However, the contig showed 100% sequence identity (112/112 bp) to the ColE1 replication origin present in different other plant transformation vectors (e.g., MH142259.1). The context sequence also showed a sequence identity of 95% (55/58 bp) to these sequences. Contig 86 originated most likely from endophytic contaminations and, thus, does not provide evidence for transgenic insertion. The contig is represented by only few reads, detectable only in one sequencing experiment, lacks any sequence identity to apple, and shows its highest level of sequence identity to other bacterial sequences.

## Conclusion

Comparative whole genome sequencing of the transgenic apple event T1190 and its non-transgenic wild-type PinS revealed 125 contigs containing subsequences with a length of ≥20 bp and with sequence identity to plant transformation vector pHTT602-CaMV35S::BpMADS4. None of these contigs provided evidence for additional transgenic insertion. Splinter sequences similar to those found in *Arabidopsis* (Schouten et al., 2017) were not detected. However, the occurrence of splinters in a transgenic plant used for rapid cycle breeding will not be problematic per se. Known splinters could be selectively outcrossed, while other undetected transgene insertions will most likely be lost during repeated backcrossing (Schouten et al., 2017). More so, if desired traits of *Malus* wild species (with astringent fruits) are introgressed into *M.* × *domestica*, at least five generations of crossings are necessary to remove most of the unwanted wild apple genome (Joshi et al., 2009). Consequently, an apple with improved fruit quality can be expected in the BC’4 generation at the earliest using accelerated breeding technology. Genotypes of this generation have on average of only 3.125% of the genomes of each parent used in the first cross. The probability that undetected transgenic insertions originating from T1190 will be present in the final product of such crossbreed program is relatively low.

The sequencing depth of this study is far more than that of previously reported studies (Yang et al., 2013; Park et al., 2015; Schouten et al., 2017). Therefore, we can safely rule out sufficiency of the sequencing depth. A further increase in the sequencing depth will not necessarily improve the detection of transgenic insertions. The differences in the coverage of the reference genome were not significant between the two experiments. However, some of the suspicious contigs (37, 80, 83, 84, 86, 94, 96, 99, 100, 109, 112, and 118) could only be detected in experiment 1, and for some contigs (i.e., 37, 80, 83, 84, 86, and 94) the origin could not be definitively clarified. The fact that some contigs were generated only in experiment 1 could be attributed to the plant material used for DNA isolation rather than the differences in sequencing depth, as the sequencing depth was higher in experiment 2. In experiment 1, the DNA was isolated from potted greenhouse-grown apple plants, which are exposed to natural-like environmental conditions such as microorganisms. Contaminations with sequences originating from those microorganisms can be expected. We, therefore, suggest using the genomic DNA of *in vitro* grown plantlets (if available) for sequencing-based detection of transgenic insertions to avoid such problems in the future. New sequencing technologies such as PacBio or Oxford Nanopore Technologies, which produce longer read lengths, have been shown to be helpful for detecting transgenic insertions (full and partial fragments), multiple insertions, as well as intra- and inter-chromosomal rearrangements at the site of integration (Jupe et al., 2019). These sequencing platforms provide direct evidence of transgene inversions, reveal bacterial contaminations, and validate the integrity of neighboring genes (Nicholls et al., 2019).

Optimization of the plant transformation vector sequence so that it does not contain any fragments with ≥20 bp sequence identity to the genome of the genotype to be transformed would additionally facilitate all subsequent analyses.

In conclusion, this study did not find unequivocal evidence that the transgenic apple clone T1190 contains previously unknown insertions of ≥20 bp sequence identity to the plant transformation vector pHTT602-CaMV35S::BpMADS4. We provided evidence that T1190 contains only one transgenic insertion, which is located on chromosome 4. Therefore, a progeny of T1190 can only contain splinter sequences if meiotic recombination has occurred in the region of this T-DNA insertion. However, this is very unlikely. Consequently, there is no further need to investigate progenies obtained from T1190 to exclude further unexpected foreign DNA insertions.

## Experimental Procedures

### Plant Material

Proliferating axillary shoot cultures of PinS were propagated *in vitro* on a shoot proliferation medium as described by Flachowsky et al. (2007). The shoot cultures of T1190 were grown on the same medium supplemented with 100 mg L^–1^ kanamycin under identical light and temperature conditions.

Own-rooted plants of PinS and T1190 were grown in 25-cm plastic pots in a greenhouse at 20°C with natural day/night cycle without extra light.

### DNA Isolation

Plasmid DNA was isolated from the *Escherichia coli* strain JM 109/pHTT602-CaMV35S::*BpMADS4* (Elo et al., 2001) with Gene JET^TM^ Plasmid Miniprep Kit (Thermo Fisher Scientific, Erlangen, Germany) according to the instructions of the manufacturer.

For the extraction of genomic DNA, young leaves of T1190 and PinS were collected either from greenhouse-grown potted trees (sequencing experiment 1) or *in vitro*-grown plantlets (sequencing experiment 2). Genomic DNA was extracted using DNeasy Plant Mini Kit (Qiagen, Hilden, Germany) according to the instructions of the manufacturer.

### Plasmid- and Plant-DNA Sequencing

Re-sequencing of the pHTT602-CaMV35S::*BpMADS4* plant transformation vector (Elo et al., 2001) was performed by shotgun DNA-Sanger-sequencing, and 3 μg of plasmid DNA was used as template. Plasmid DNA preparation, ultra-sonication, vector ligation and transformation, picking of 192 single colonies (for 4× coverage), sequencing, and assembly was performed by LGC Genomics (Berlin, Germany). Additional sequencing of single regions of the plasmid was performed by DNA-Sanger sequencing in collaboration with Eurofins MWG Operon (Ebersberg, Germany).

The first whole gene sequencing experiment of T1190 and PinS was performed at IPK Gatersleben (Gatersleben, Germany) using DNA isolated from the leaf material of own-rooted greenhouse plants (sequencing experiment 1). For library preparation, 400 ng of genomic DNA of each sample were fragmented by ultrasound (S220, Covaris, Inc., Woburn, MA, United States), ligated to Y-shaped adapters, and PCR-amplified following the TruSeqDNA v2 protocol (Illumina, San Diego, CA, United States). Each library, size selected on an agarose gel for an insert size of 300–400 bp, was sequenced on one lane of an Illumina high-throughput flow cell with a HiSeq2000 (Illumina, San Diego, CA, United States) instrument using the paired-end 100 bp module.

The second whole genome sequencing experiment of T1190 and PinS was performed at the Functional Genomics Center Zurich (Switzerland) using DNA isolated from the tissue of the *in vitro* plantlets as described above (sequencing experiment 2). The DNA was then used to prepare Illumina libraries (Illumina TruSeq Nano DNA Library Preparation, Illumina, San Francisco, CA, United States) with an average insert size of 500 bp. The library was sequenced on an Illumina HiSeq4000 instrument using the paired-end 150 bp module.

### Bioinformatics Analyses

Raw sequencing data were adapted and quality trimmed with Trim Galore^2^ (version 0.4.0, non-default parameter: quality ≥ 30, read length ≥ 50). The trimmed reads were mapped against the chromosomes of the reference genome GDDH13 (Daccord et al., 2017) using BWA-mem (v0.7.15-r1140) (Li, 2013). Subsequently, the percentage of covered bases within the chromosomes was determined for each sample using bedtoolsgenomecov (Version v2.29.2, -dz –split) (Quinlan and Hall, 2010).

The trimmed read pairs were filtered for the occurrence of any 20-mer that occurs in the plant transformation vector with a minimum base quality of 20. The frequency of each 20-mer of the plant transformation vector was computed using the filtered reads and visualized with R (R Core Team, 2020).

In addition, filtered reads from T1190 of both experiments were clustered according to the occurrence of relevant 20-mers and assembled into contigs by an overlay-layout consensus assembly in a custom script. Publicly available assemblers could not be used for this, since these assemblers can discard reads, which needs to be avoided here. Trimmed unfiltered reads of all the samples were mapped against these contigs using bwa to analyze the occurrence of these contigs in the samples T1190 and PinS. Coverage plots for each contig were created with R (R Core Team, 2020).

Spurious contigs were blasted against the *Malus* × *domestica* v3.0.a1 (Velasco et al., 2010), the GDDH13 (Daccord et al., 2017) and the ‘Gala Galaxy’ (Broggini et al., 2020) apple reference genomes. In parallel, a BLASTn search was performed at the database of the National Centre for Biotechnology Information (NCBI) to identify highly similar sequences (megablast).

### PCR Analyses

The sequence of contig84 was synthesized by Eurofins Genomics (Ebersberg, Germany) and cloned into the plasmid vector pEX-a128 (Eurofins Genomics, Ebersberg, Germany). PCR primers 84 for 5′-TAAGTTTTGGCCTTTGCTTGGT-3′ and 84rev 5′-GTTGAGGAAAGAGTTGTGTCAAAA-3′ were designed based on the contig sequence using the Primer3Plus software (Untergasser et al., 2012)^3^. The primer84rev was designed flanking the region with homology to the vector. The primer 84for was located in the regions 24–2 bp upstream of the region with vector identity. The last 8 bp of the 3′ end of this primer were designed on the potential insertion. Primer synthesis was performed by Eurofins Genomics (Edersberg, Germany).

Polymerase chain reaction (PCR) was carried out in 20 μl consisting of 20 ng of plant genomic plant DNA or 1 ng plasmid DNA, 2 μl 10× DreamTaq Buffer, 2 μl dNTP Mix (.2 mM of each),.5 μM of each primer (F and R), and 1.25 Unit DreamTaq DNA Polymerase (Thermo Fisher Scientific, Erlangen, Germany). The PCR was performed by denaturation at 94°C for 5 min, followed by 35 cycles of 30-s denaturation at 94°C, 30-s annealing at 63°C, and 1-min extension at 72°C. After the final extension at 72°C for 5 min, the PCR product was cooled down to 4°C and stored until it was used for gel electrophoresis. The amplified products were separated on a 1% agarose gel.

## Data Availability Statement

All data generated for this study are deposited in the European Nucleotide Archive (ENA; https://www.ebi.ac. uk/ena/browser/view/PRJNA267731?show=reads). All data of Experiment 1 will be released under the accession number: PRJEB46678. All data of Experiment 2 will be released under the accession number: PRJEB46739.

## Author Contributions

AP, HF, M-VH, and JK conceived and designed the experiments. AP, GB, and LA were involved in whole-genome sequencing. JK and TB performed the bioinformatics analyses. AP, SW, GB, and HF have done the analysis of the initial bioinformatics results such as PCR analyses. All the authors participated in data interpretation and writing of the manuscript.

## Conflict of Interest

The authors declare that the research was conducted in the absence of any commercial or financial relationships that could be construed as a potential conflict of interest.

## Publisher’s Note

All claims expressed in this article are solely those of the authors and do not necessarily represent those of their affiliated organizations, or those of the publisher, the editors and the reviewers. Any product that may be evaluated in this article, or claim that may be made by its manufacturer, is not guaranteed or endorsed by the publisher.

## Footnotes

1
https://www.rosbreed.org/

2
https://github.com/FelixKrueger/TrimGalore

3
https://primer3plus.com/

## Supplementary Material

Supporting Information 1 Detailed information of the 125 contigs, which contain sequences of ≥ 20 bp with identity to the plant transformation vector.

Supporting Information 2 Coverage plots of all 125 contigs. The y-axis represents the coverage of a sequence region along the contig (x-axis). Red line, sequence coverage using the data of PinS; black line, sequence coverage using the data of T1190; green area, part of the contig with 100% sequence identity to the sequence of the plant transformation vector.

Supporting Information 3 Results of BLASTn analyses of contigs that contain sequence fragments, which are identical to the plant transformation vector pHTT602-CaMV35S::BpMADS4.

Supporting Information 4 Contigs underrespresented in sequencing data.
