# Supplementary material for: No Evidence of Unexpected Transgenic Insertions in T1190 – A Transgenic Apple Used in Rapid Cycle Breeding – Following Whole Genome Sequencing
Source: Front Plant Sci. 2021 Aug 11;12:715737. doi: 10.3389/fpls.2021.715737 (PMC8386123; doi:10.3389/fpls.2021.715737)
Supplement: Supporting Information 2 — Coverage plots of all 125 contigs. The y-axis represents the coverage of a sequence region along the contig (x-axis). Red line, sequence coverage using the data of PinS; black line, sequence coverage using the data of T1190; green area, part of the contig with 100% sequence identity to the sequence of the plant transformation vector. [file Data_Sheet_2.pdf]

Supporting information 2. Coverage plots of all 125 contigs. The y-axis represents the coverage of a sequence region along the contig (x-axis). Red line, sequence coverage using the data of PinS; black line, sequence coverage using the data of T1190; green area, part of the contig with 100% sequence identity to the sequence of the plant transformation vector.

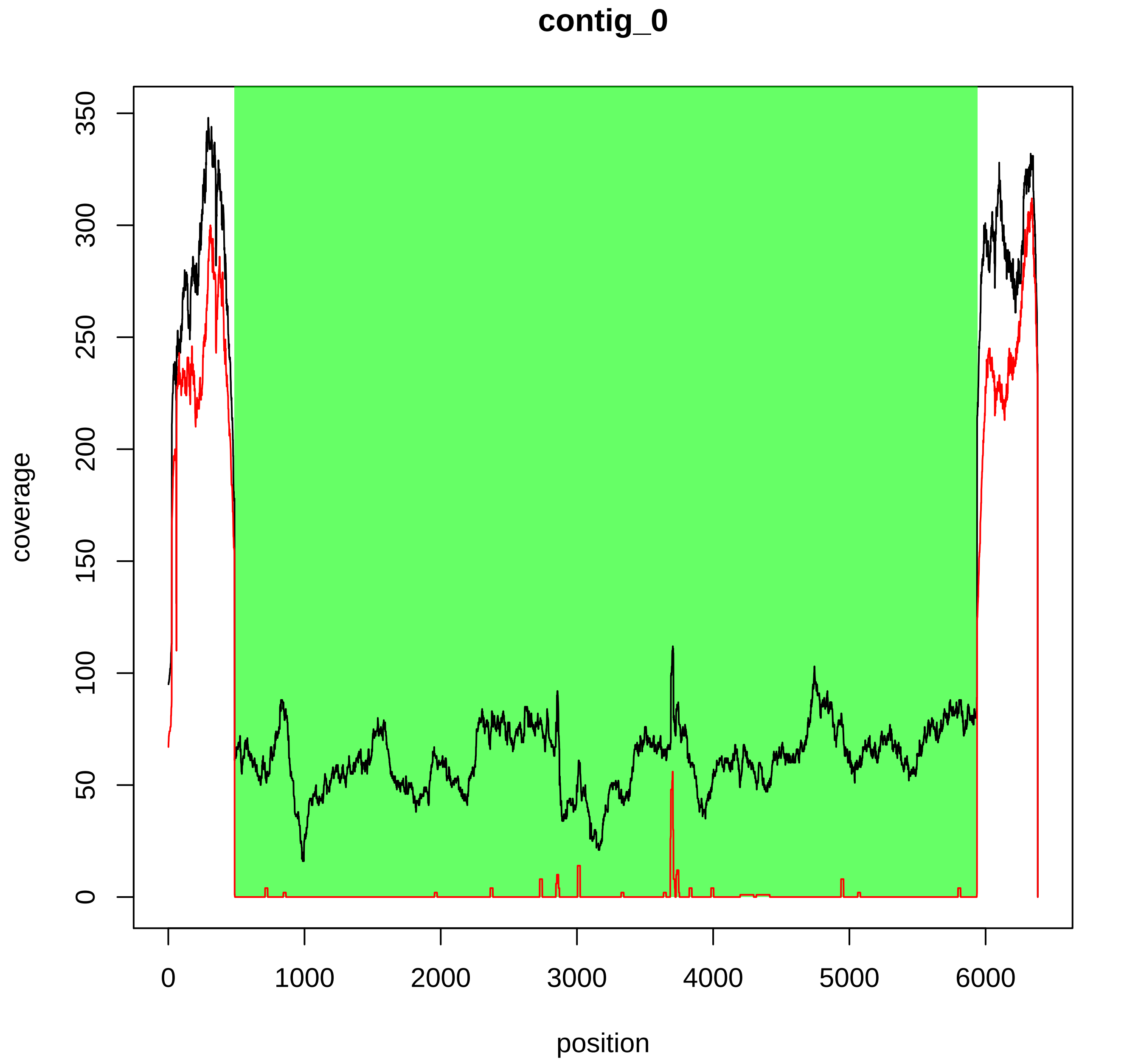
contig_0
coverage
position
0
50
100
150
200
250
300
350
0
1000
2000
3000
4000
5000
6000

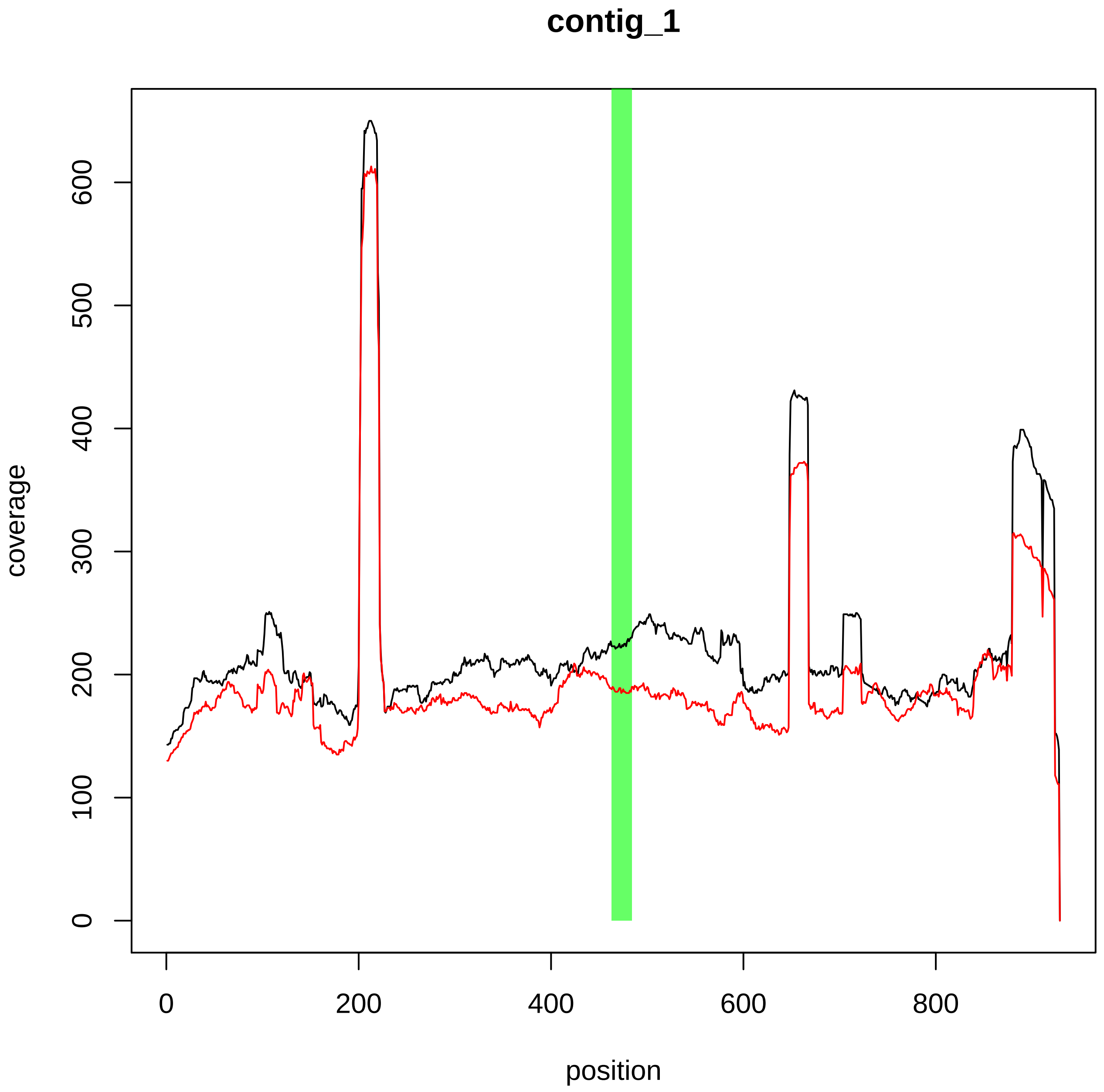
contig_1
coverage
0
100
200
300
400
500
600
position
0
200
400
600
800

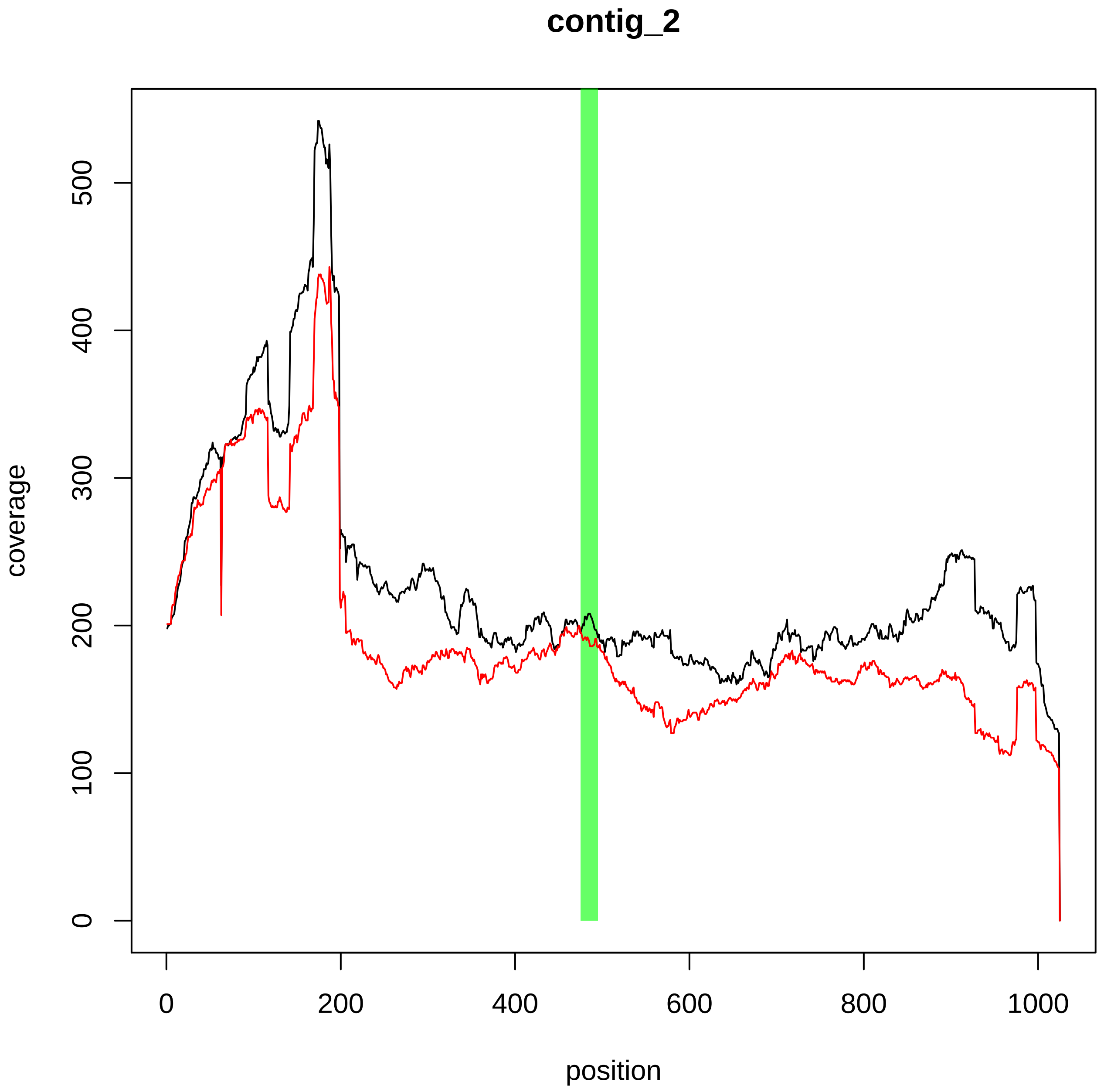
contig_2
coverage
0
100
200
300
400
500
position
0
200
400
600
800
1000

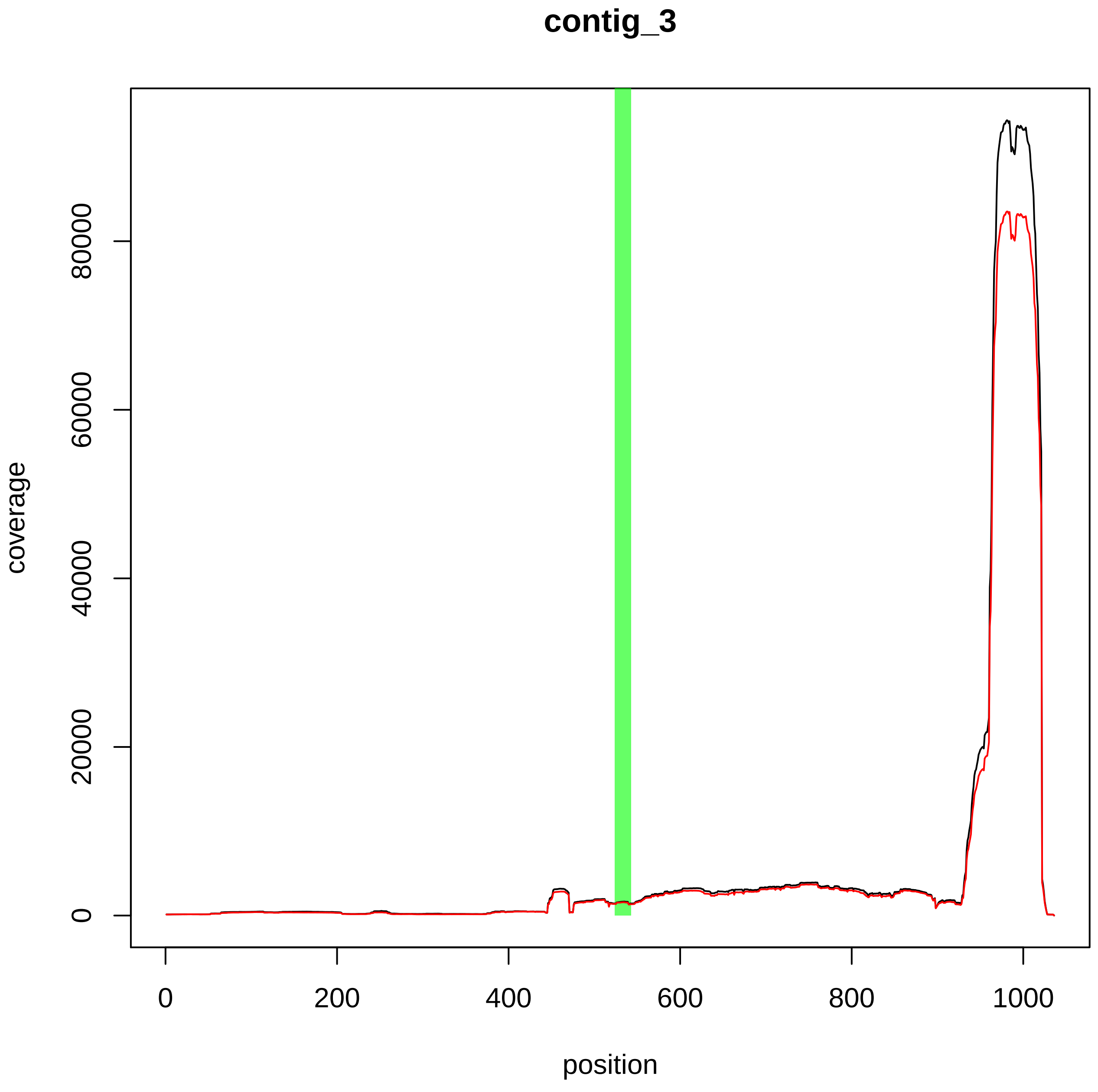
contig_3
coverage
0
20000
40000
60000
80000
0
200
400
600
800
1000
position

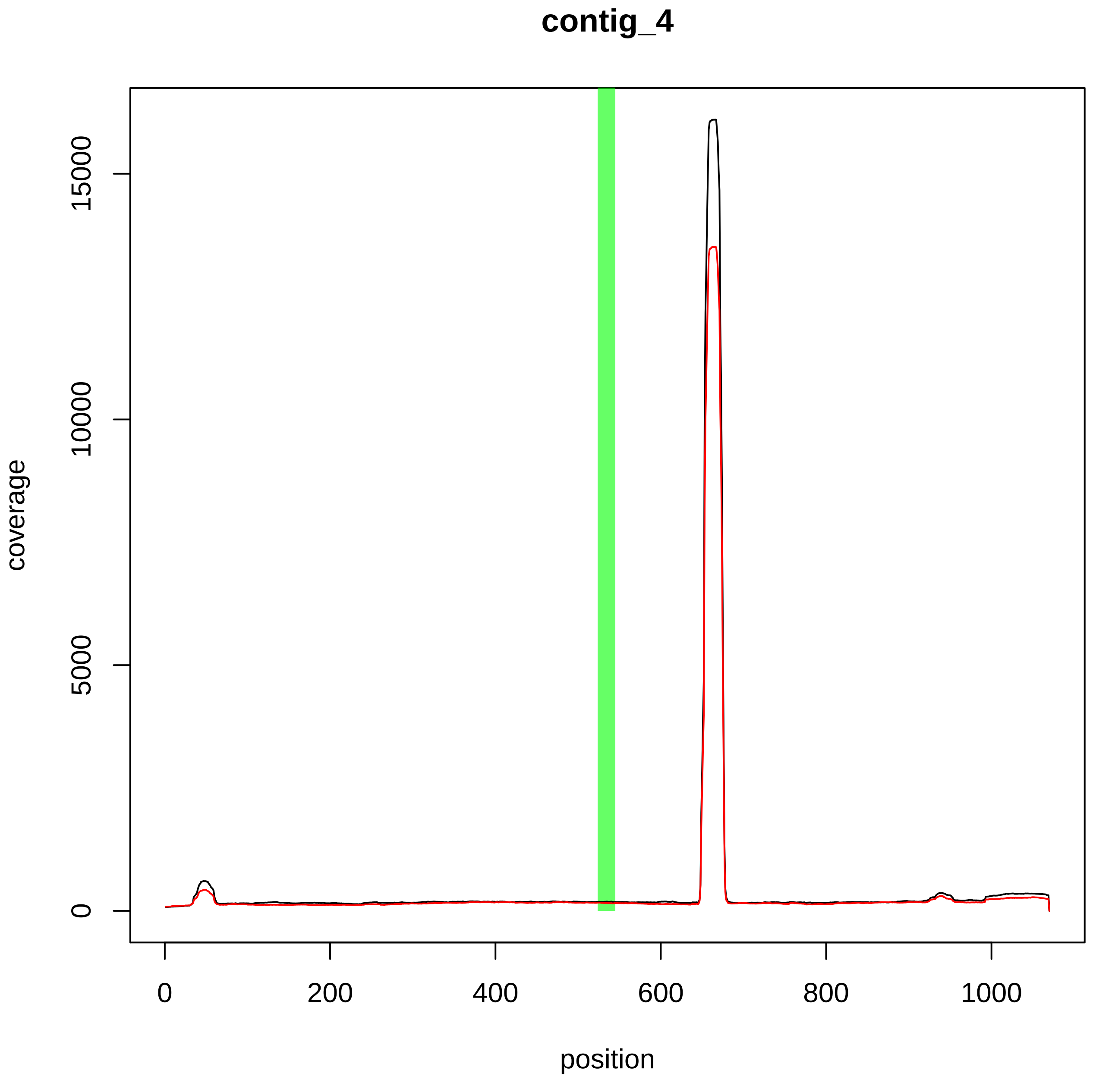
contig_4
coverage
15000
10000
5000
0
0
200
400
600
800
1000
position

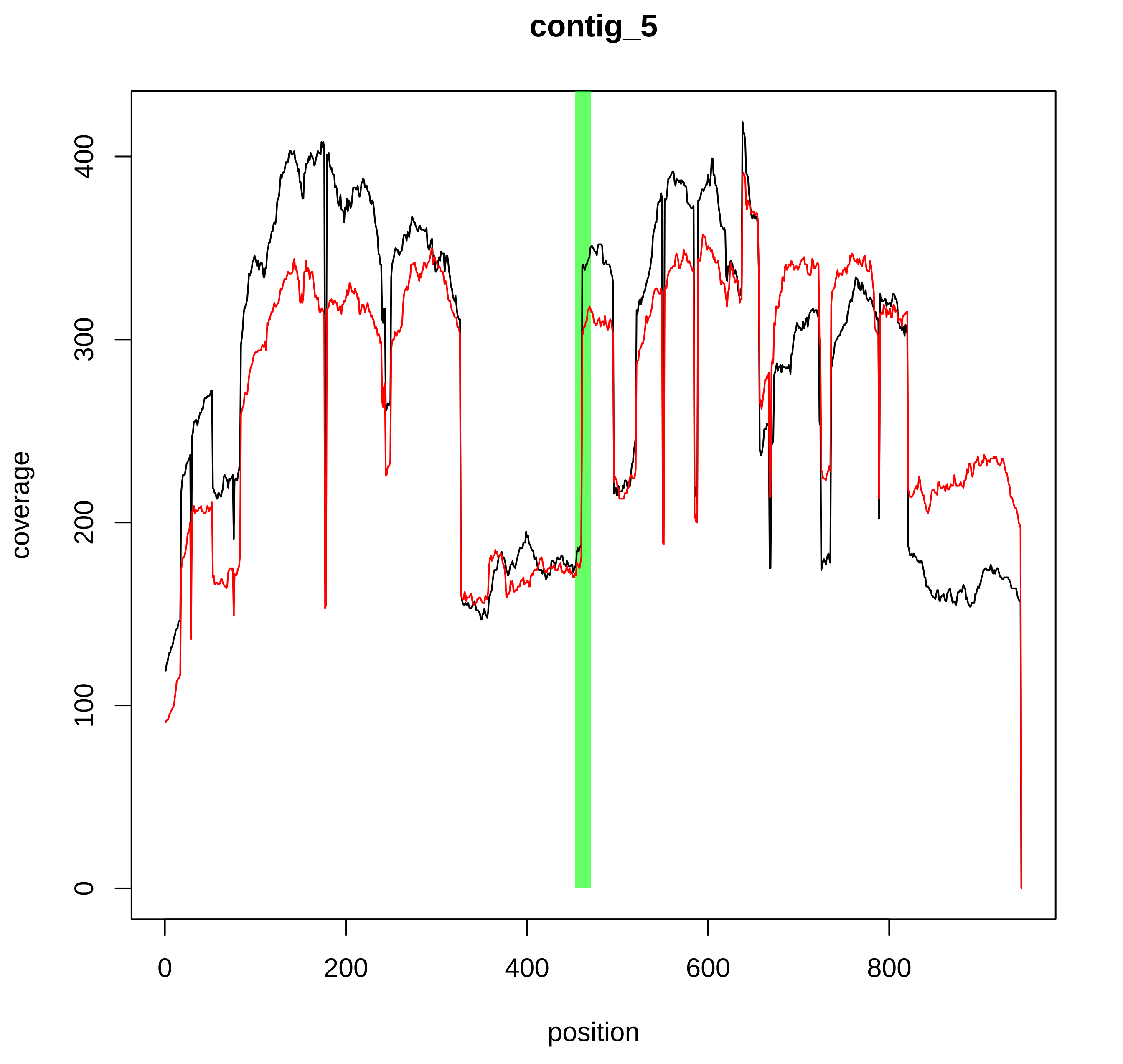

contig_5
coverage
position
0
100
200
300
400
0
200
400
600
800

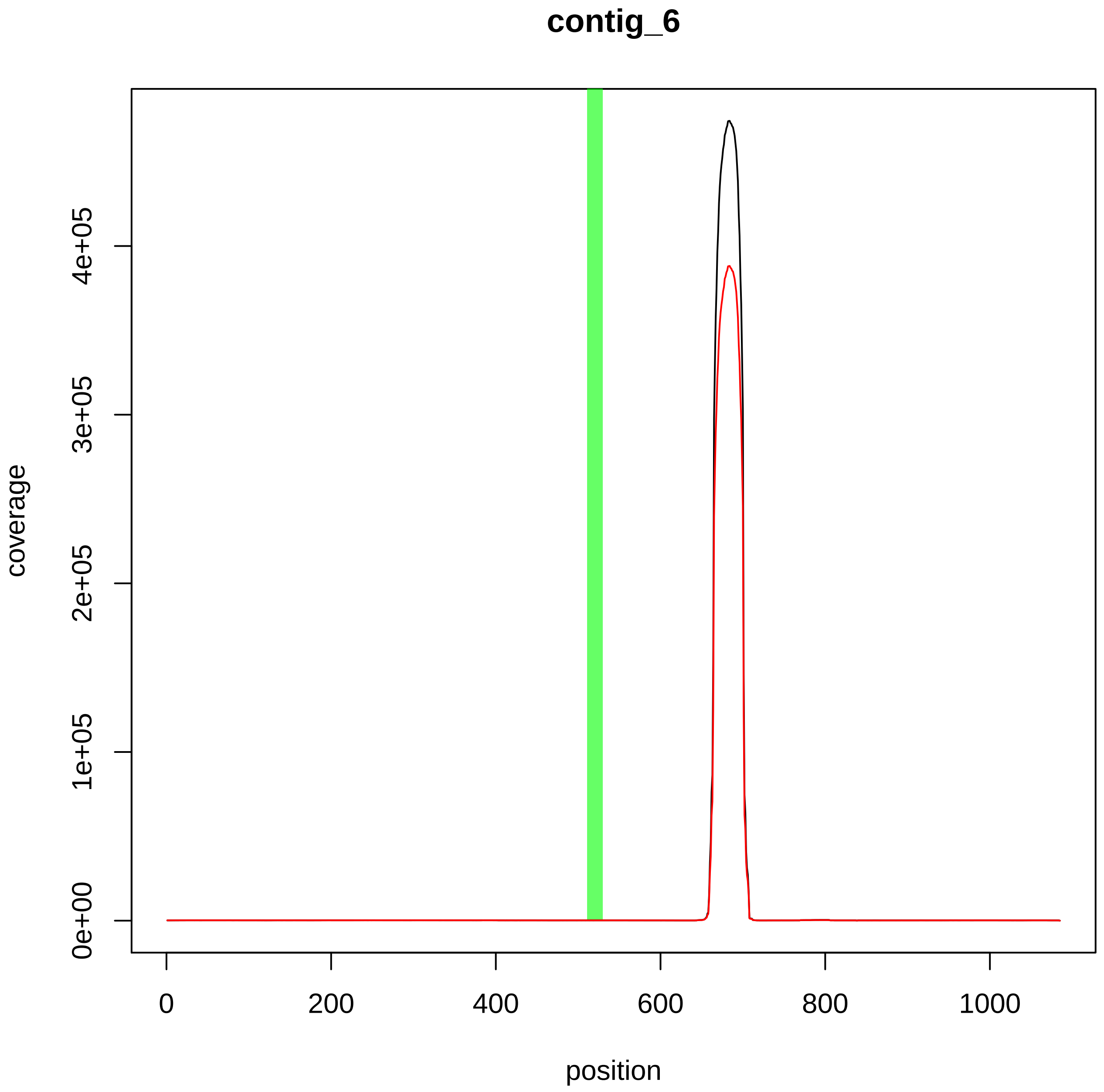

contig_6
coverage
0e+00
1e+05
2e+05
3e+05
4e+05
0
200
400
600
800
1000
position

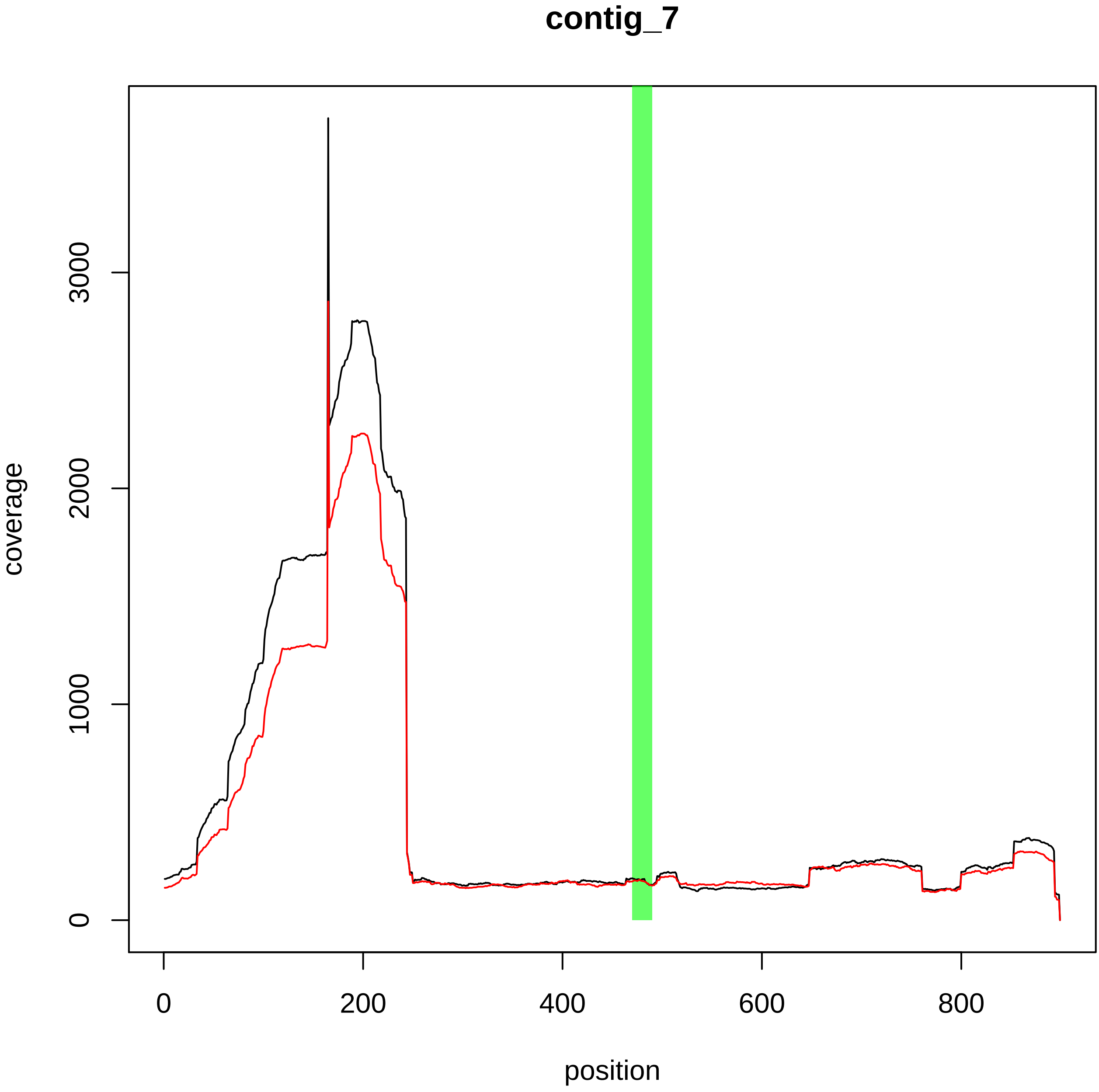
contig_7
coverage
0
1000
2000
3000
0
200
400
600
800
position

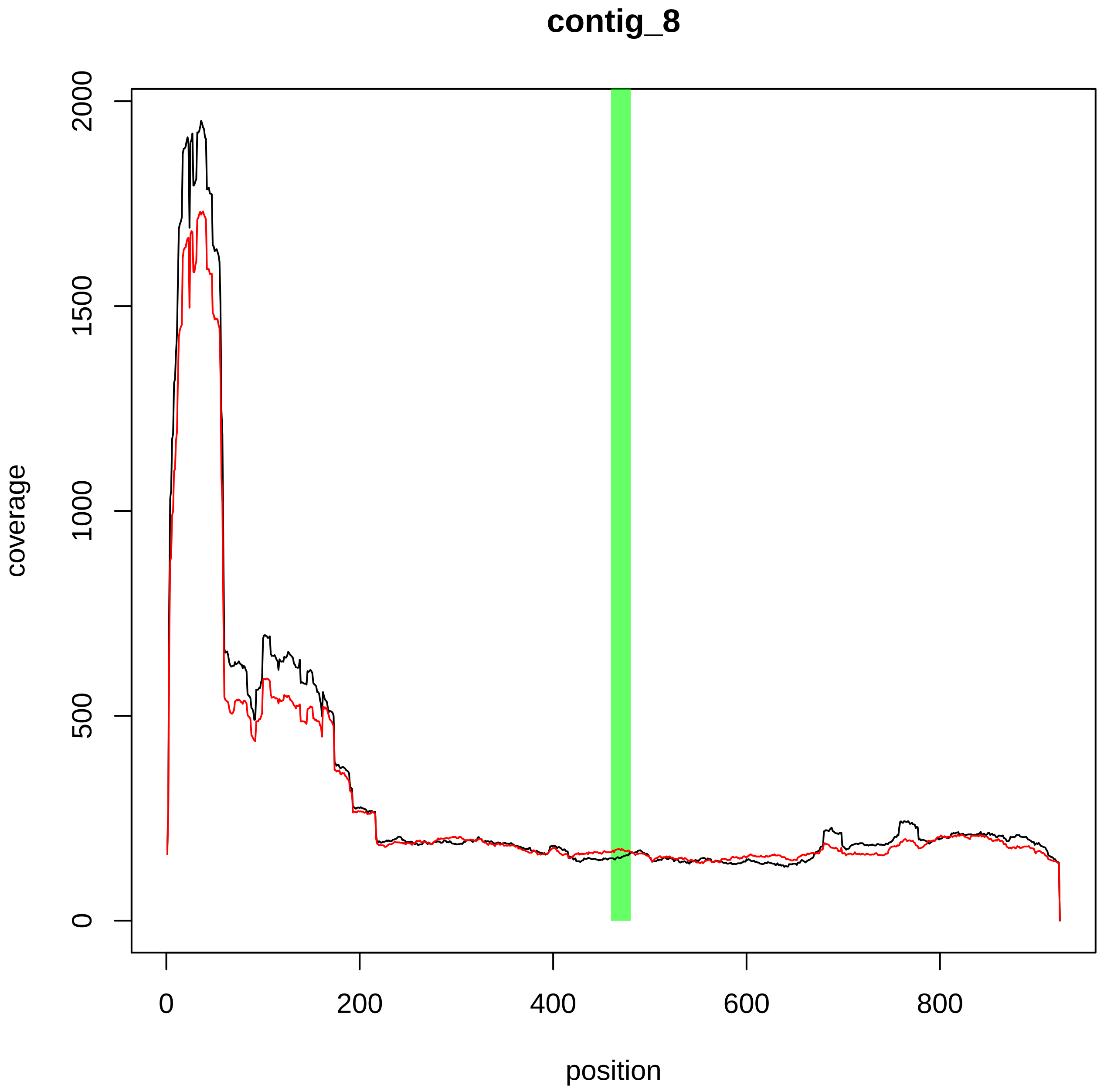

contig_8
coverage
position
0
500
1000
1500
2000
0
200
400
600
800

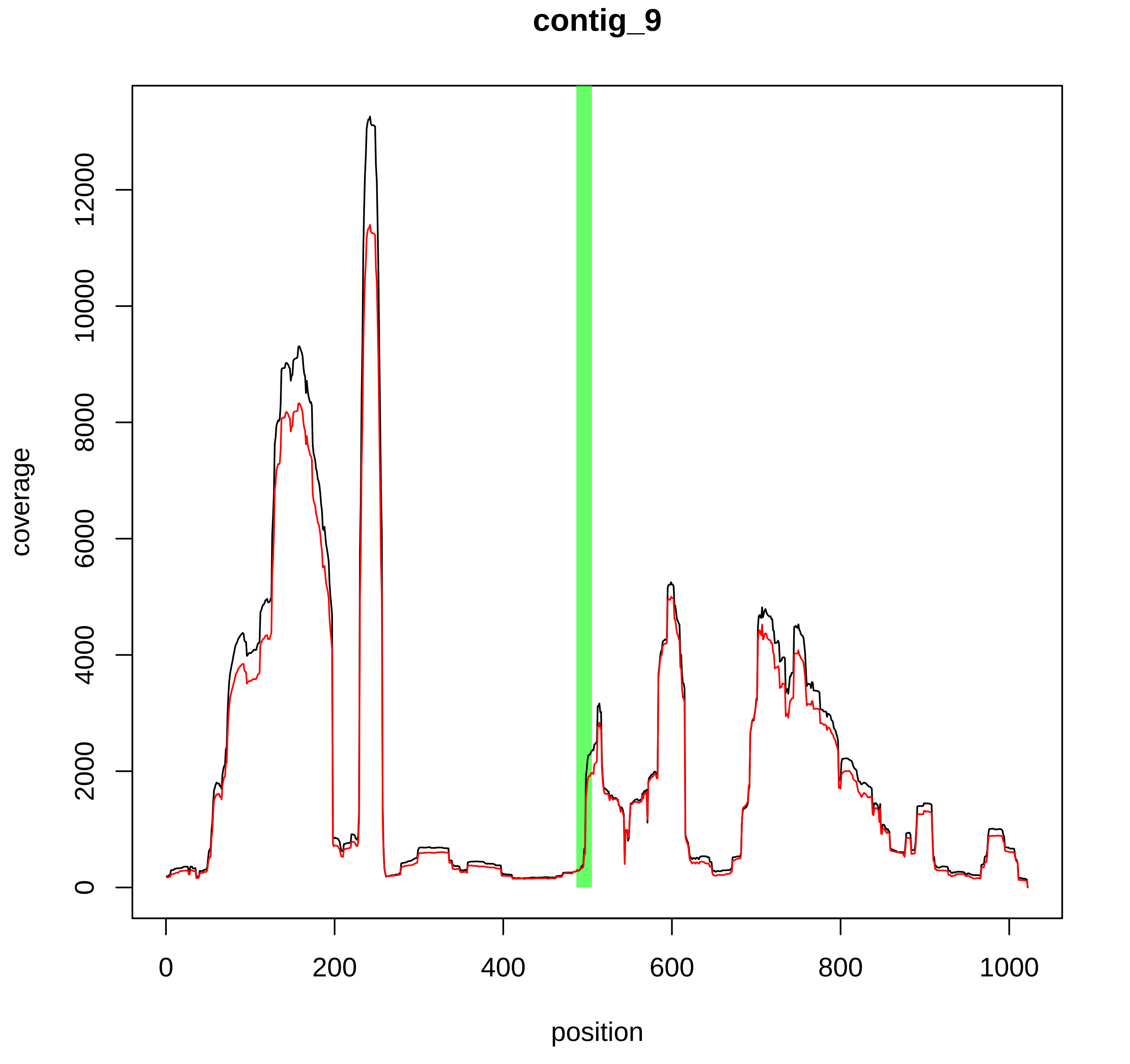
contig_9
coverage
position
0
2000
4000
6000
8000
10000
12000
0
200
400
600
800
1000

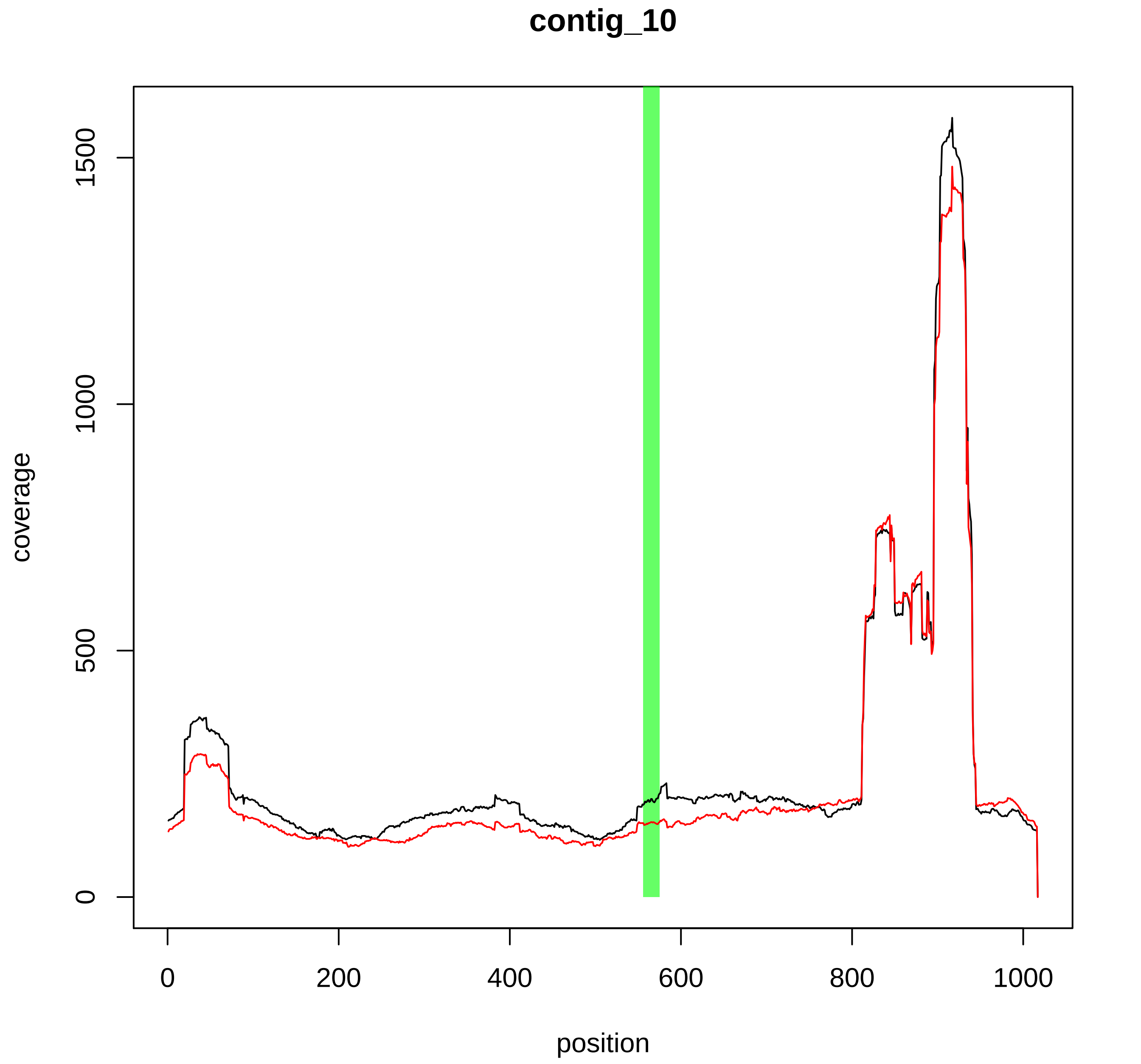
contig_10
coverage
0
500
1000
1500
position
0
200
400
600
800
1000

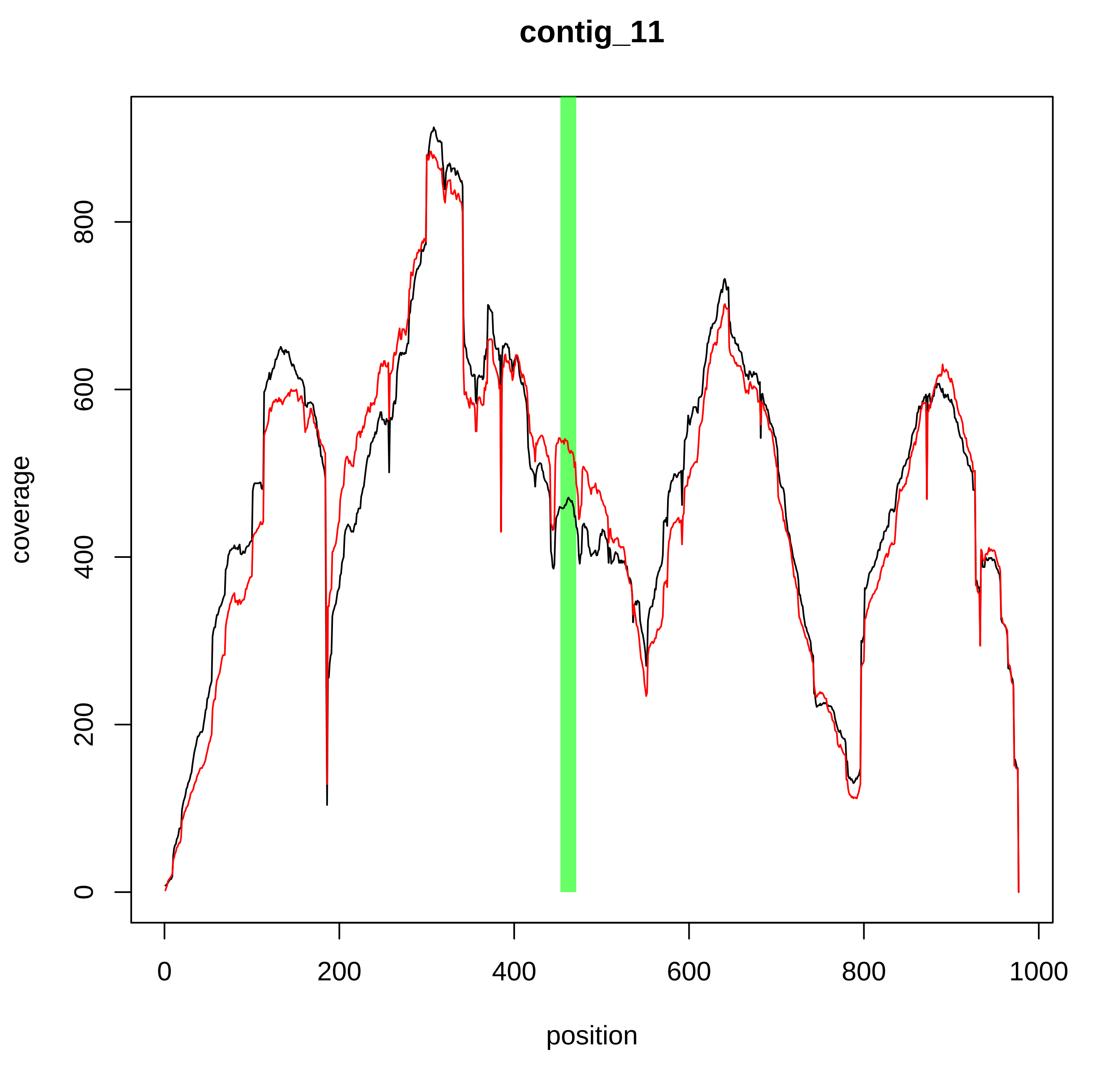
contig_11
coverage
0
200
400
600
800
position
0
200
400
600
800
1000

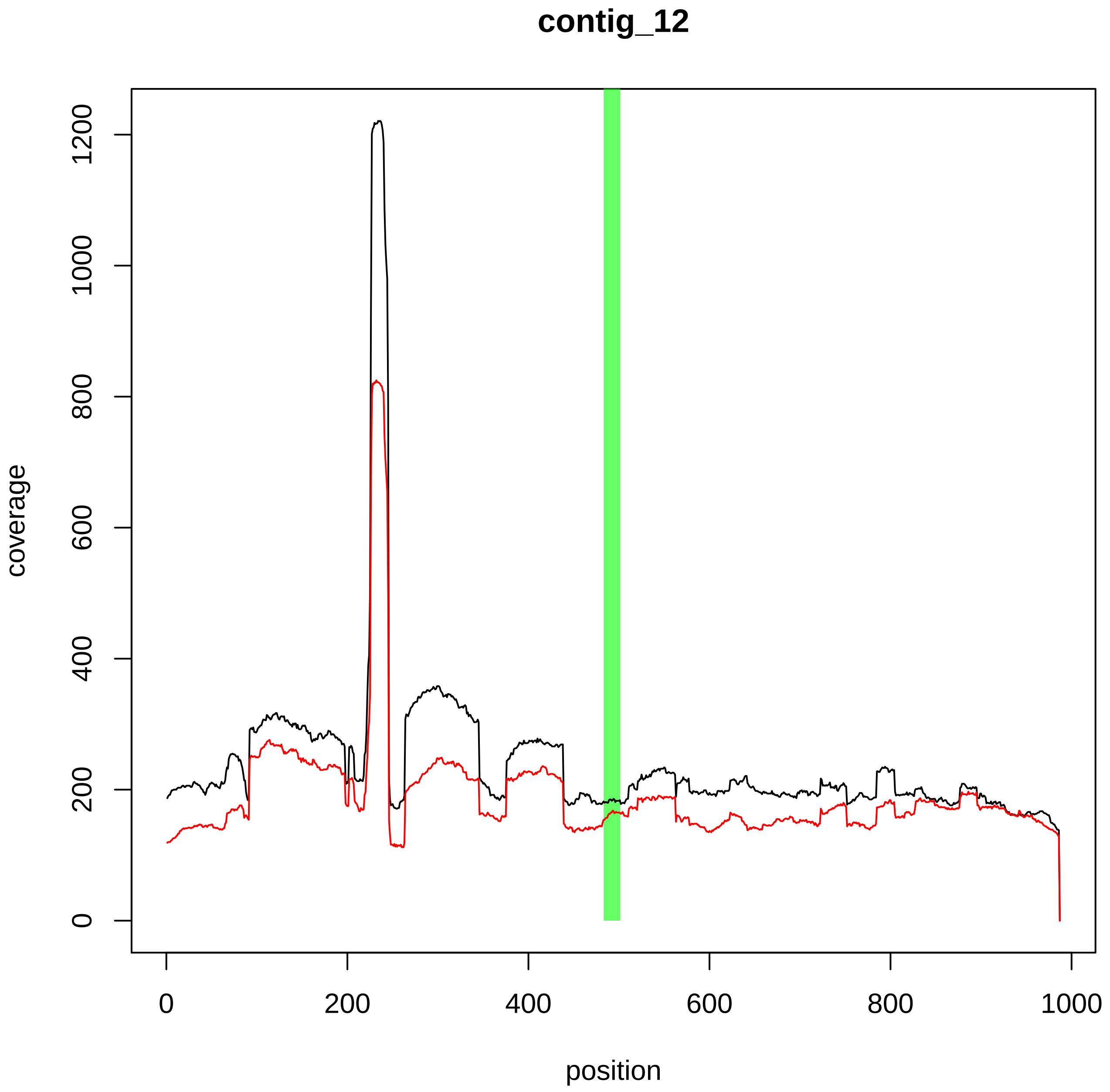
contig_12
coverage
position
0
200
400
600
800
1000
1200

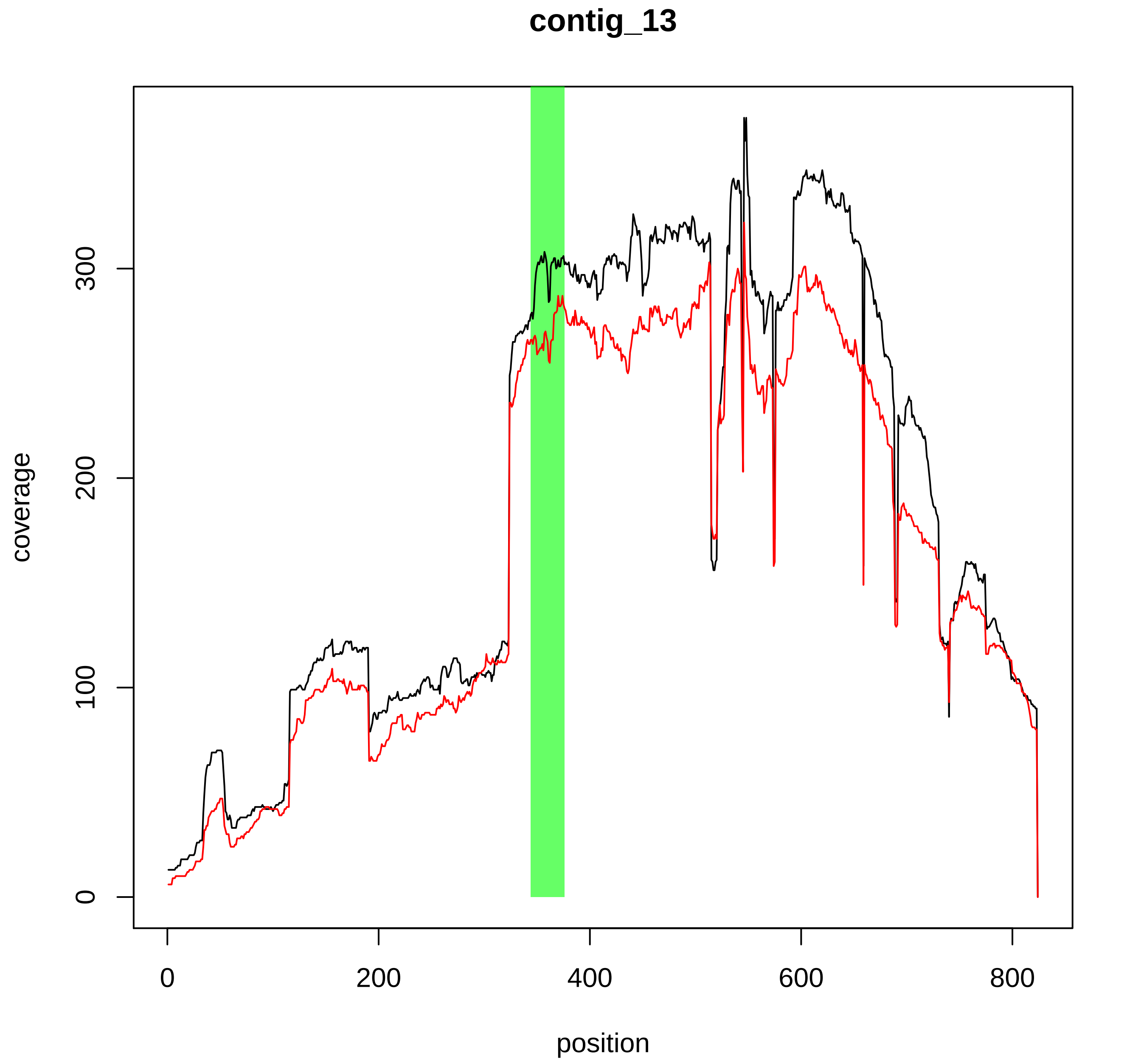

contig_13
coverage
0
100
200
300
position
0
200
400
600
800

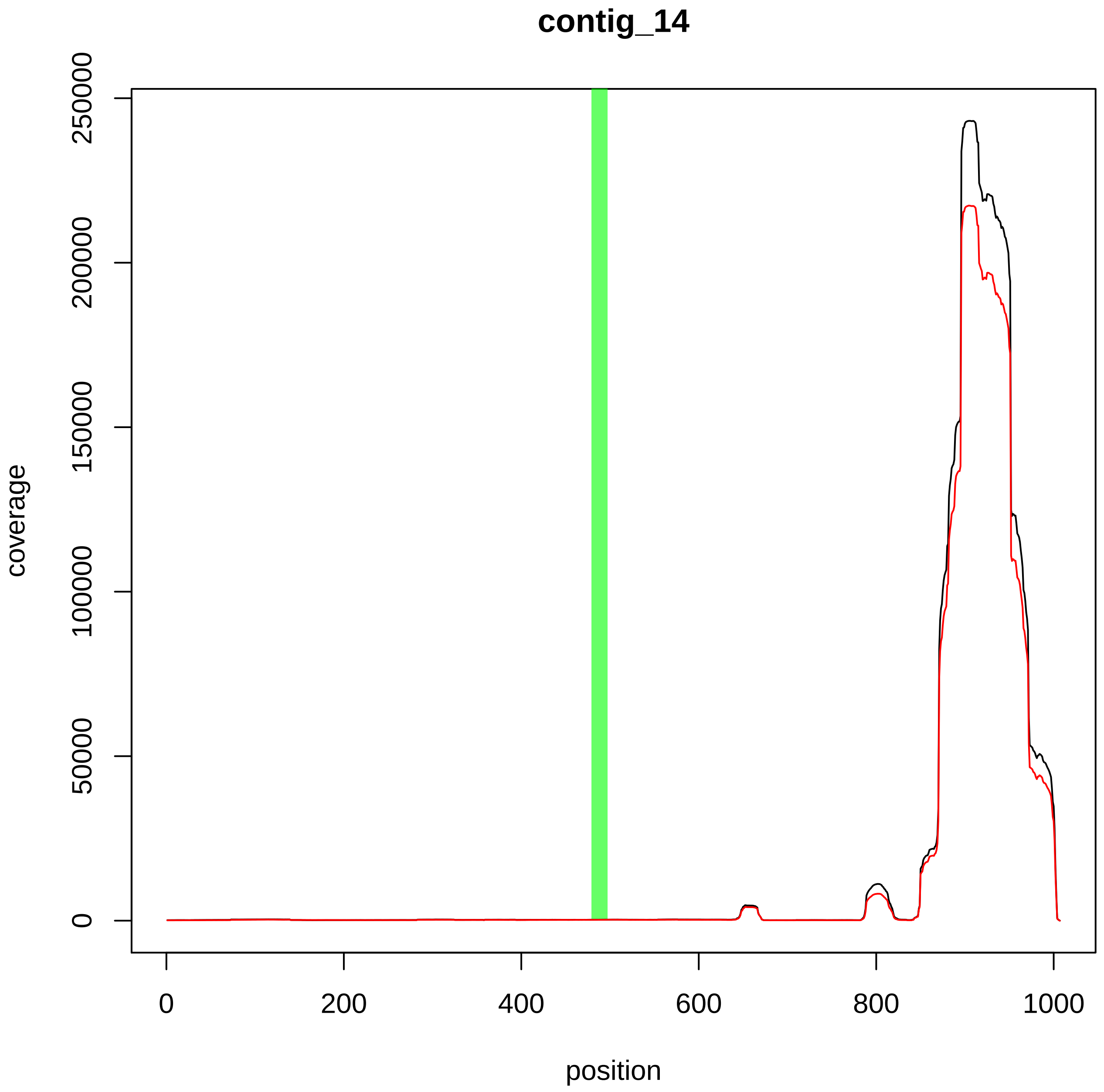
contig_14
coverage
position
0
50000
100000
150000
200000
250000
0
200
400
600
800
1000

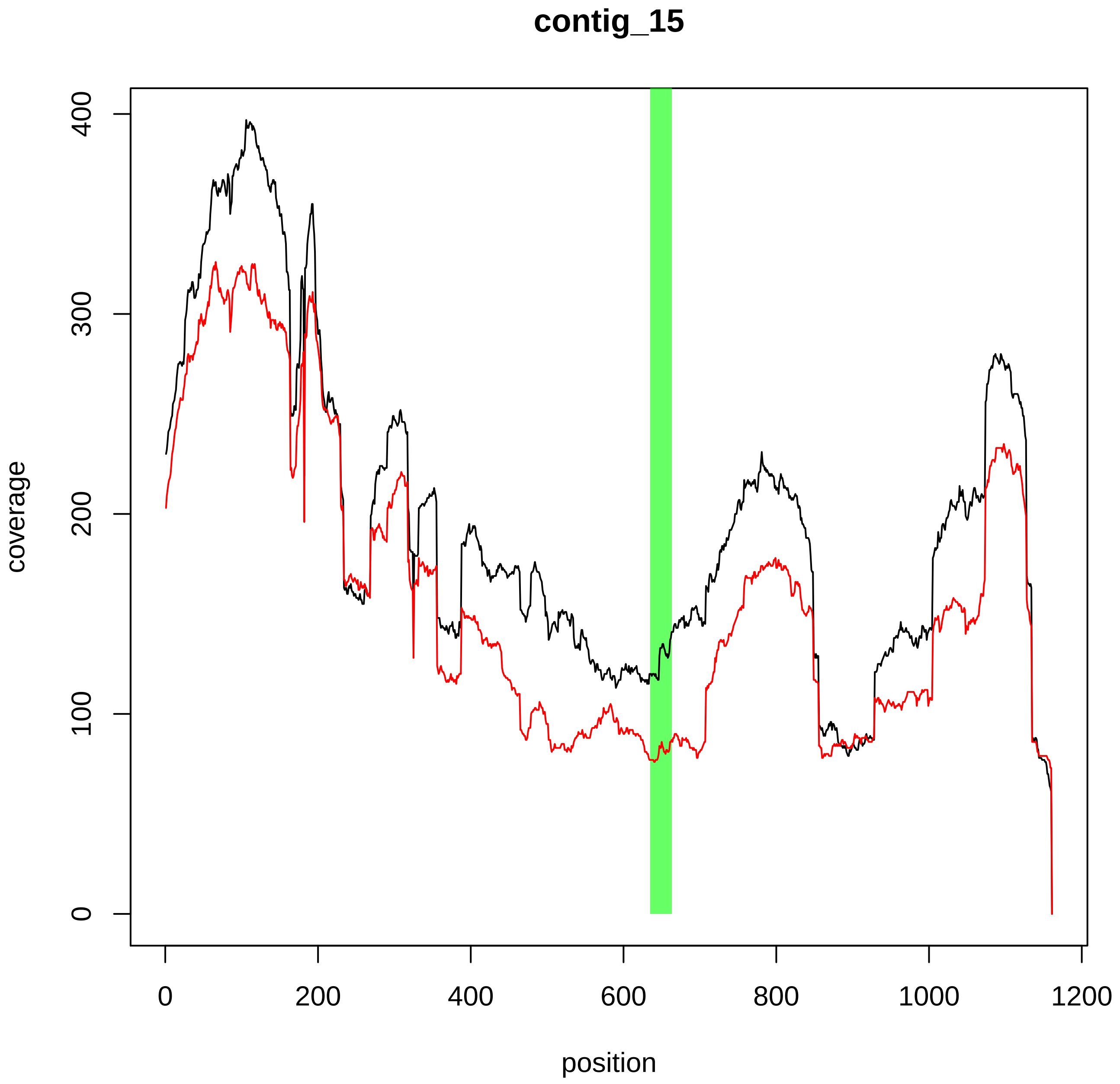

contig_15
coverage
400
300
200
100
0
position
0
200
400
600
800
1000
1200

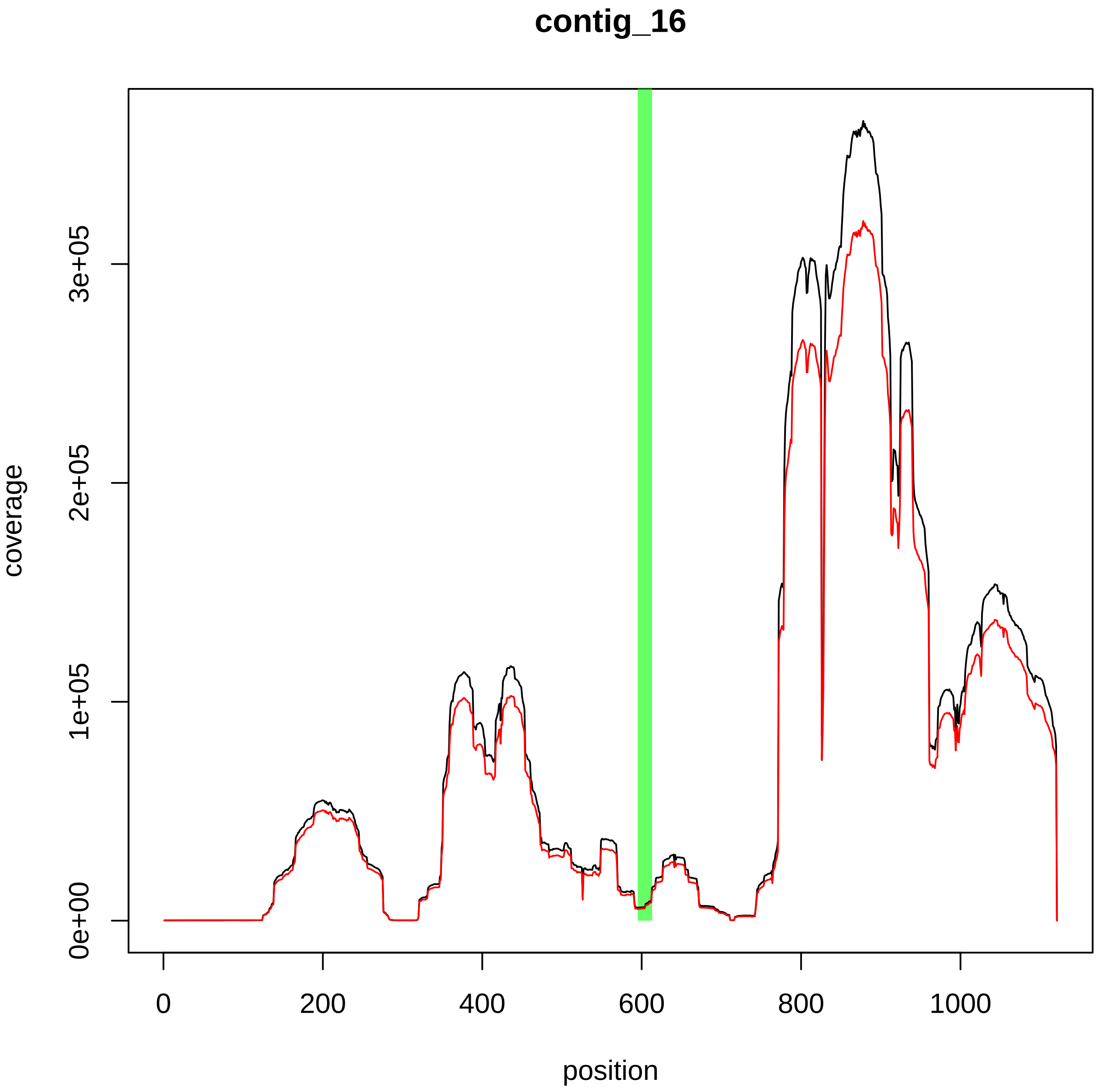
contig_16
coverage
0e+00
1e+05
2e+05
3e+05
position
0
200
400
600
800
1000

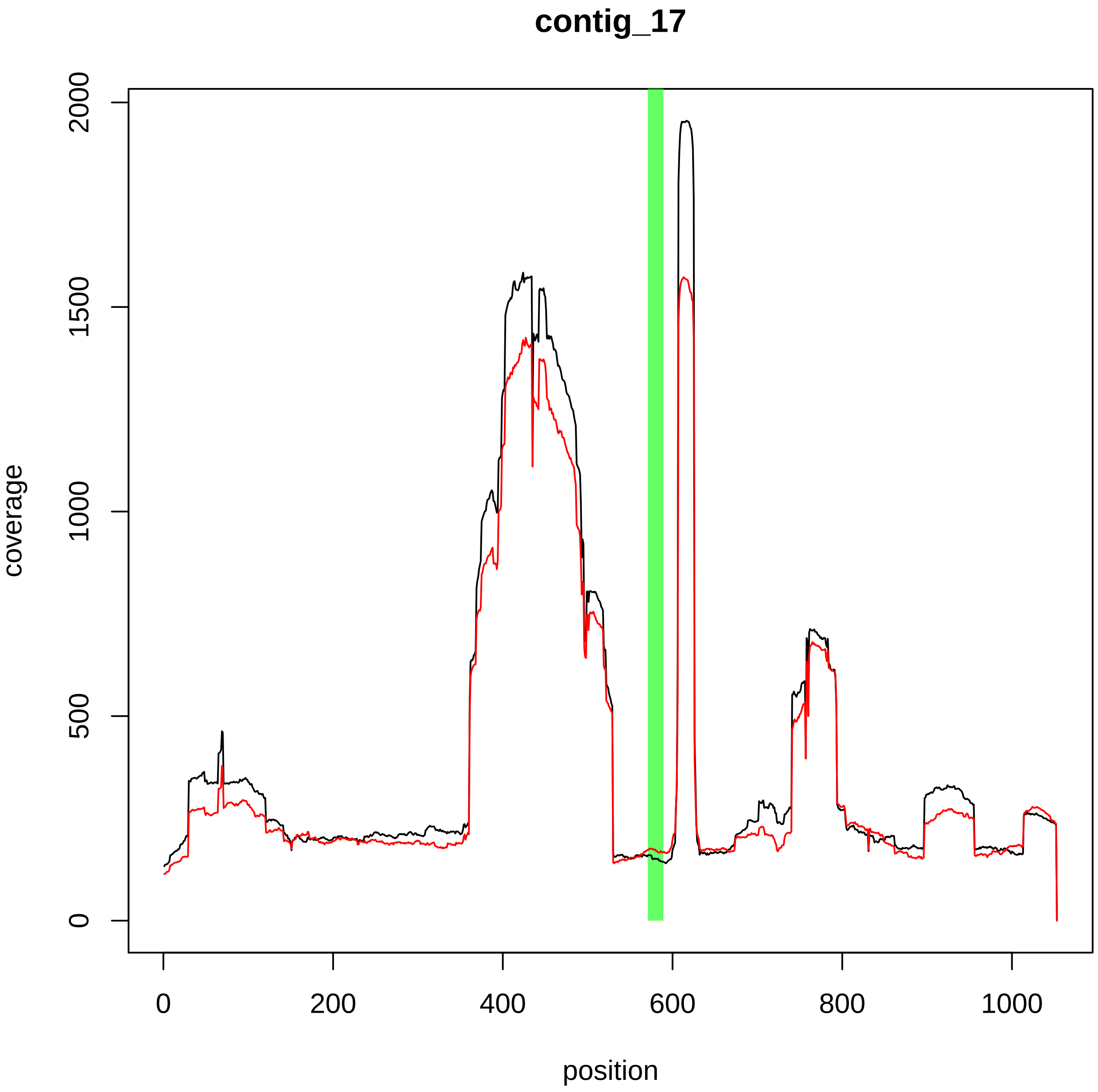
contig_17
coverage
position
0
500
1000
1500
2000
0
200
400
600
800
1000

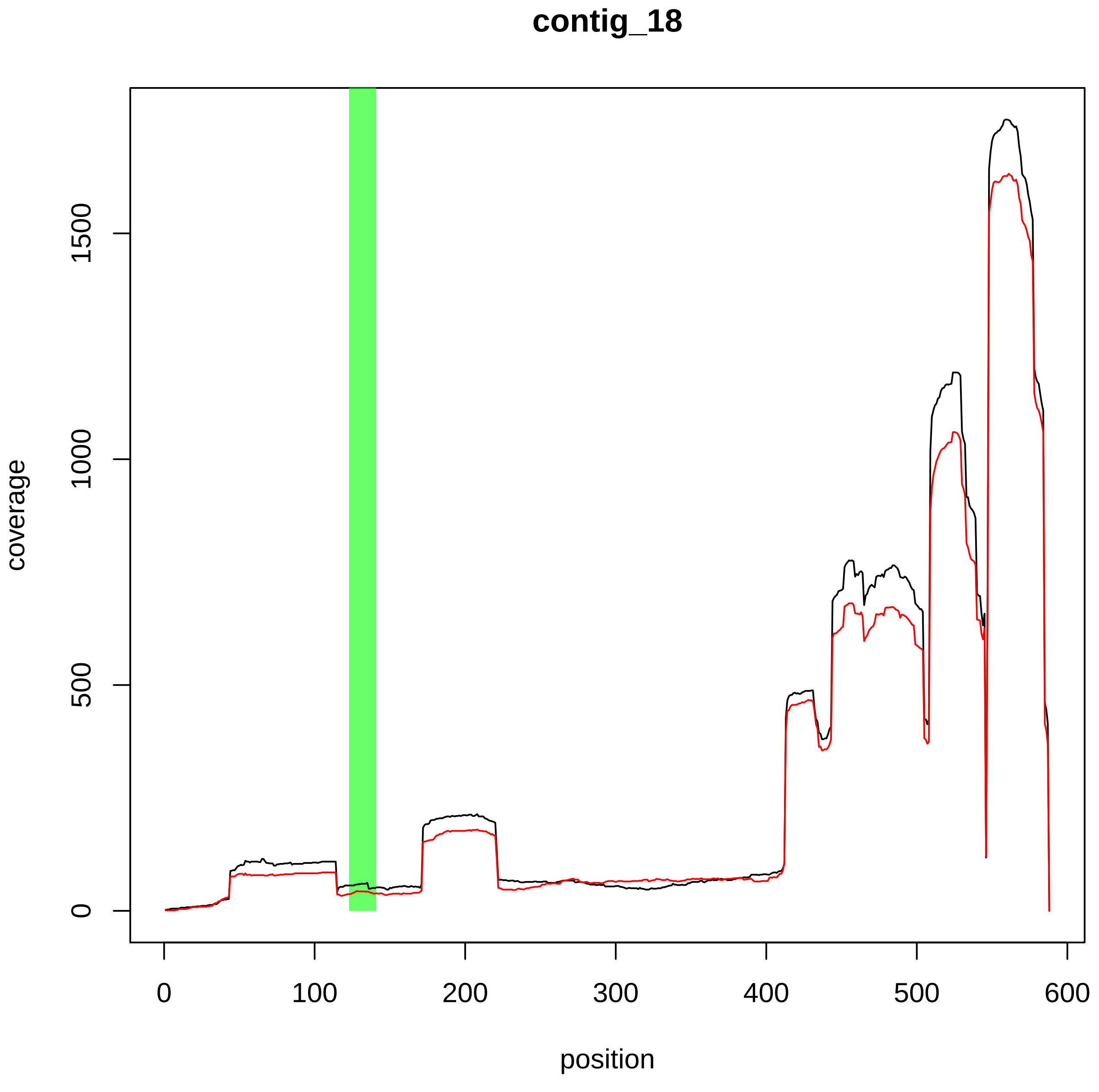

contig_18
coverage
position
0
500
1000
1500
0
100
200
300
400
500
600

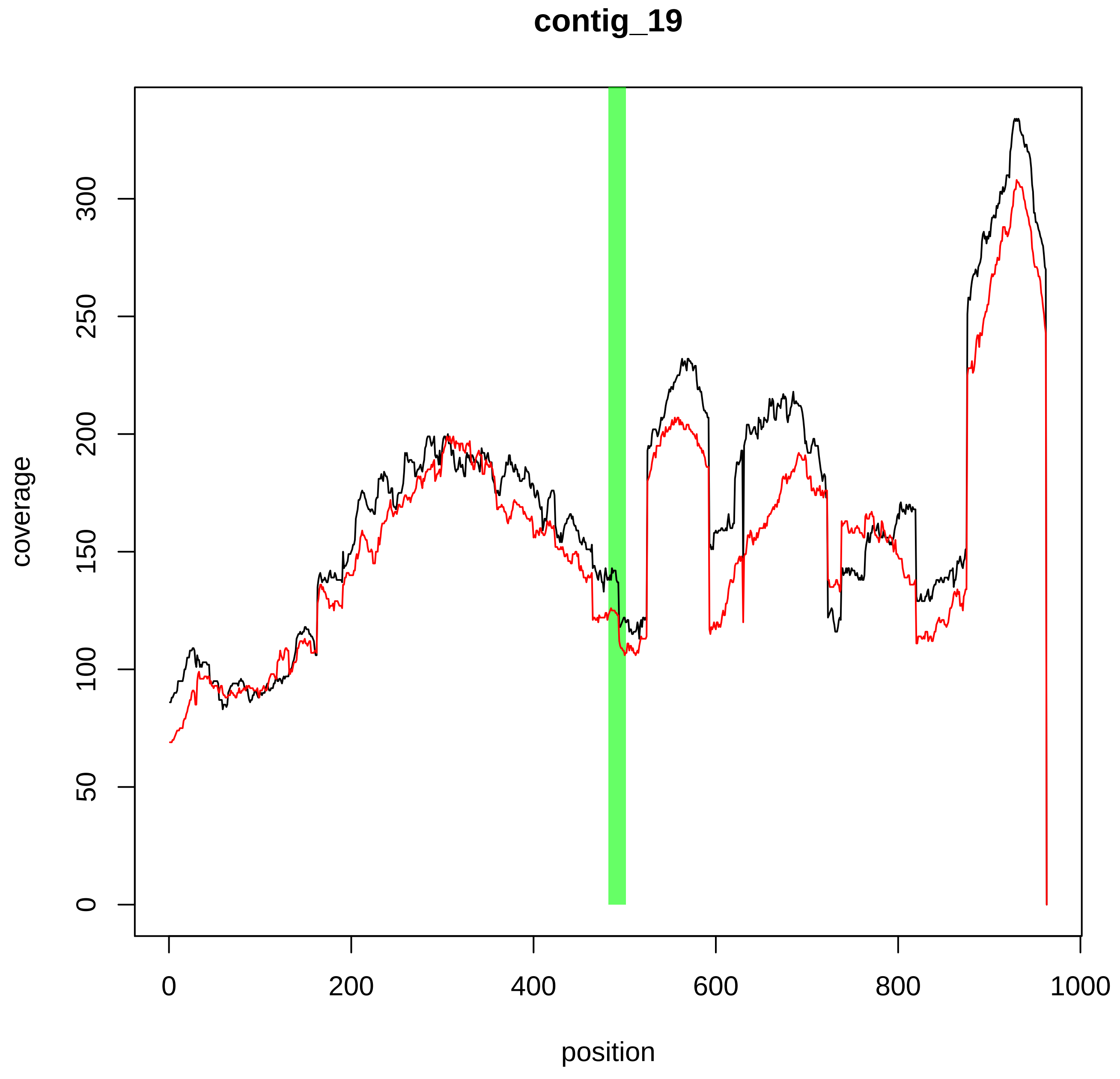
contig_19
coverage
position
0
50
100
150
200
250
300
0
200
400
600
800
1000

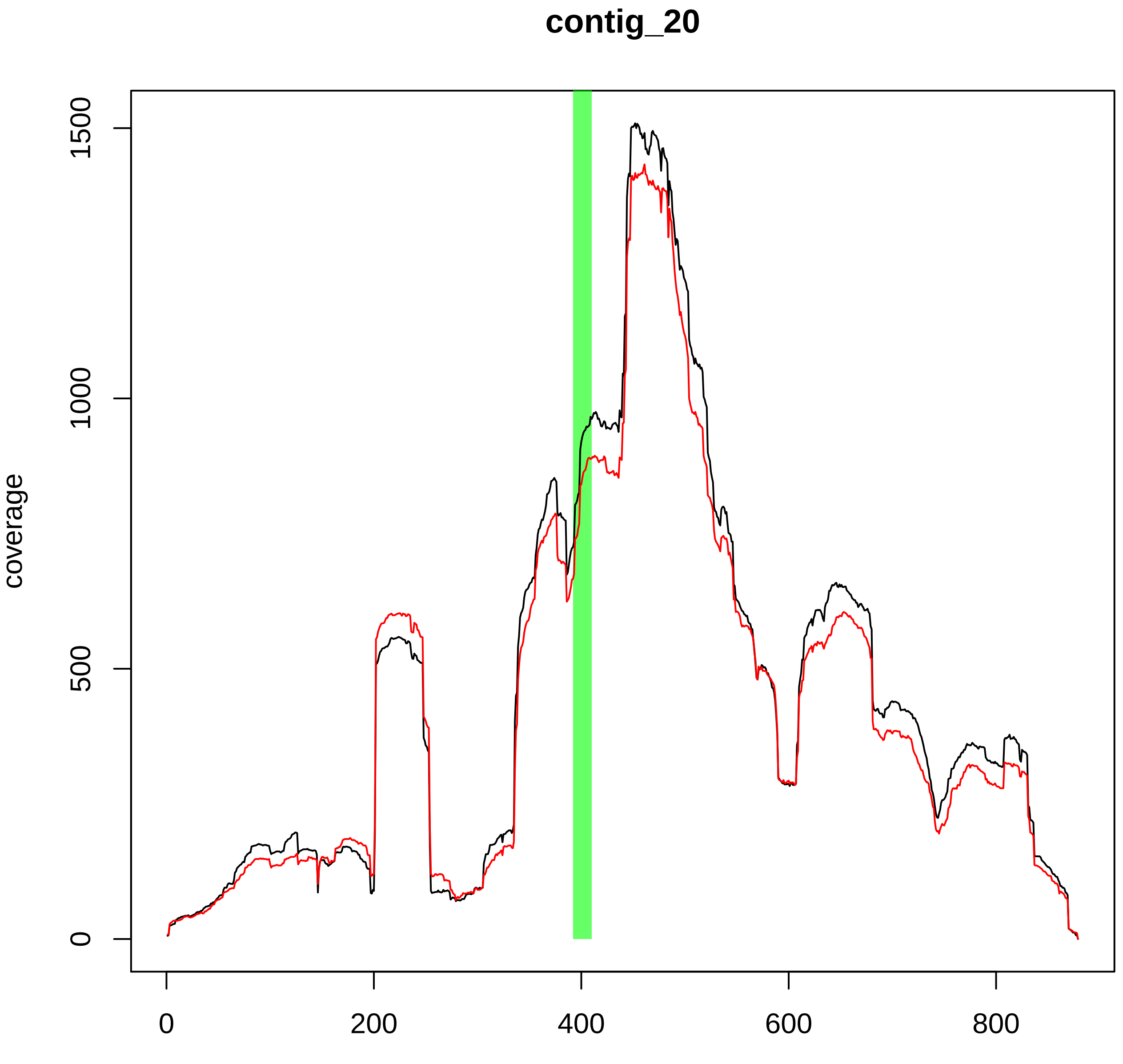
contig_20
coverage
position
0
500
1000
1500
0
200
400
600
800

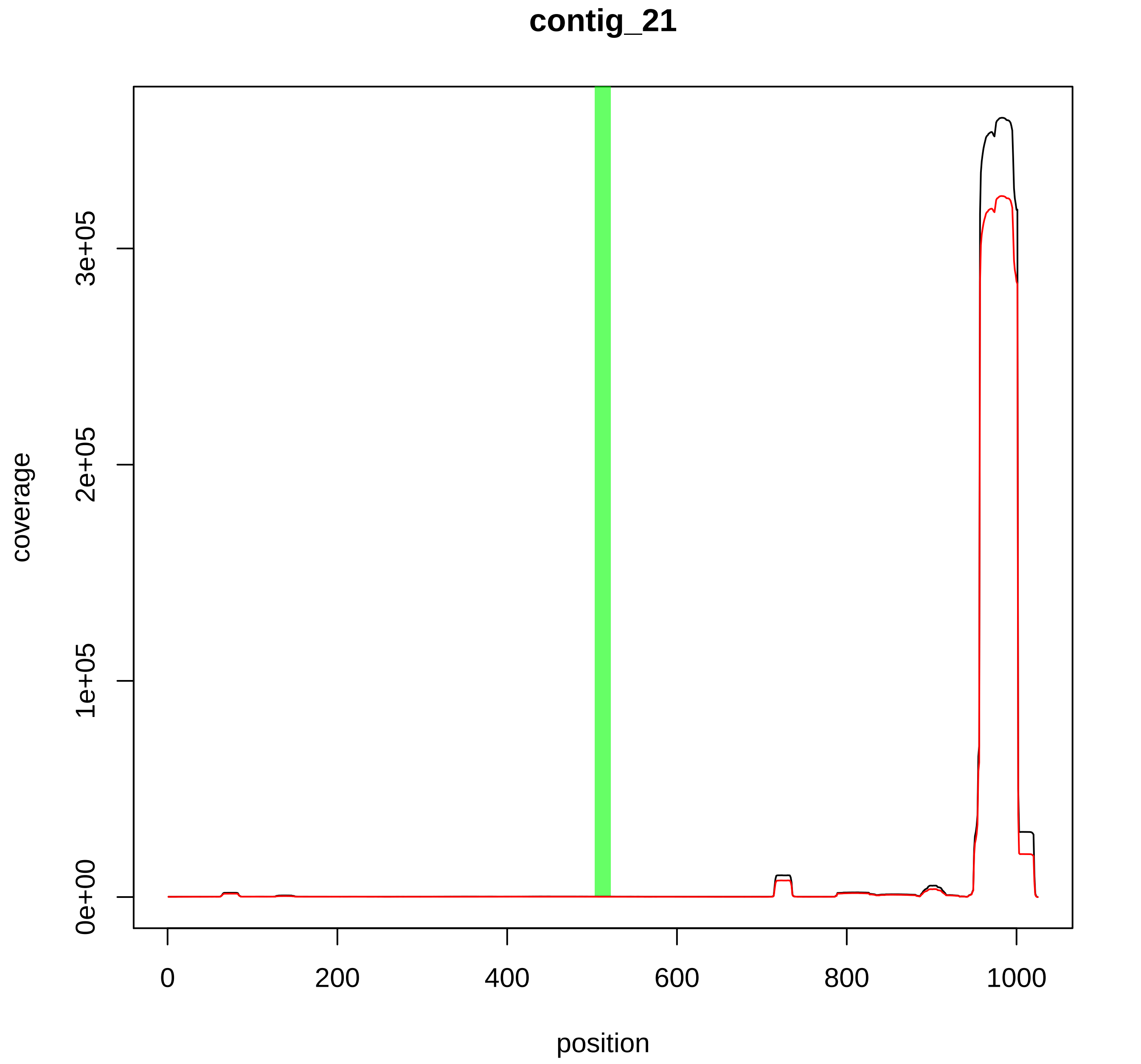
contig_21
coverage
0e+00
1e+05
2e+05
3e+05
0
200
400
600
800
1000
position

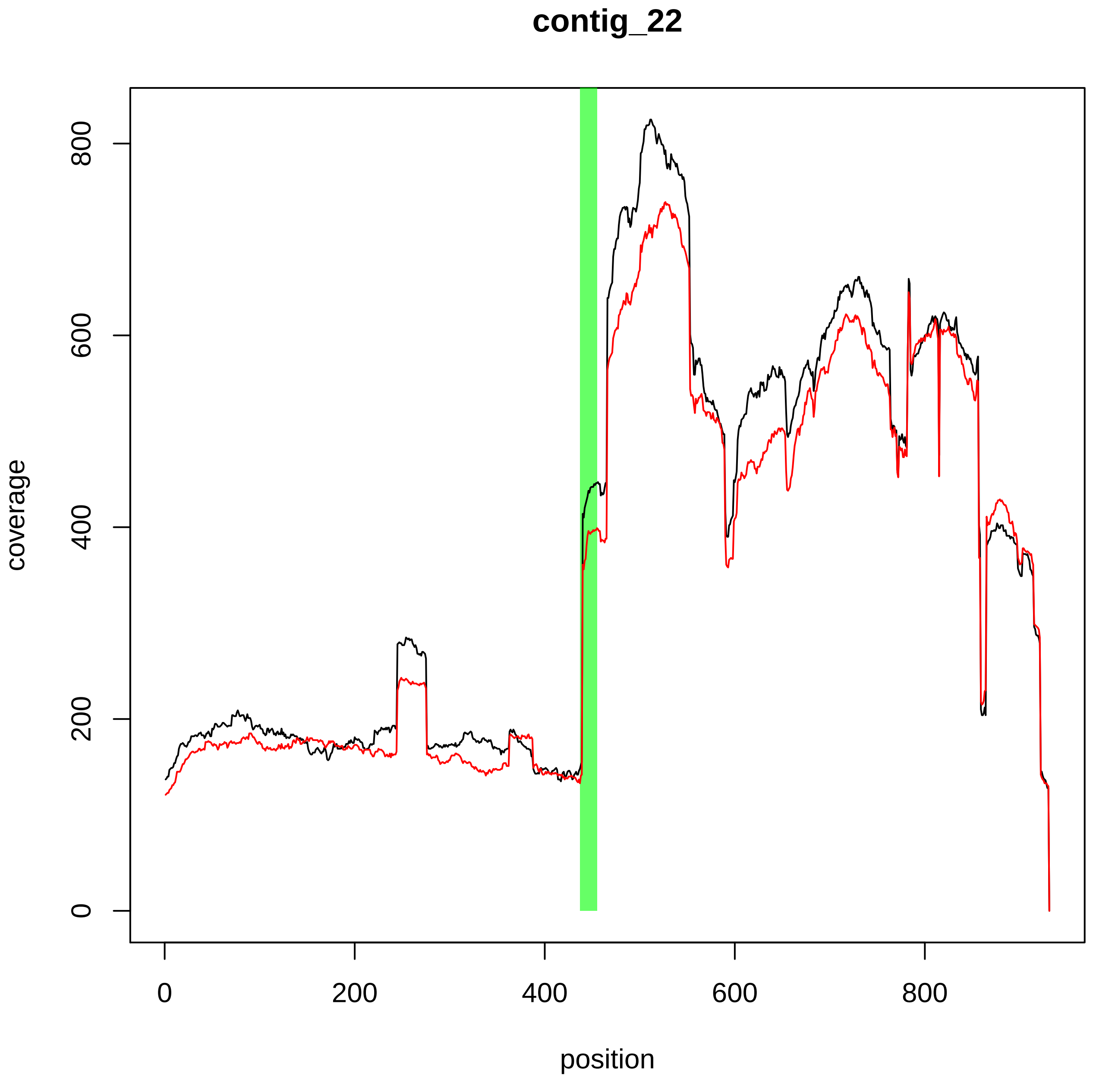
contig_22
coverage
position
0
200
400
600
800
0
200
400
600
800

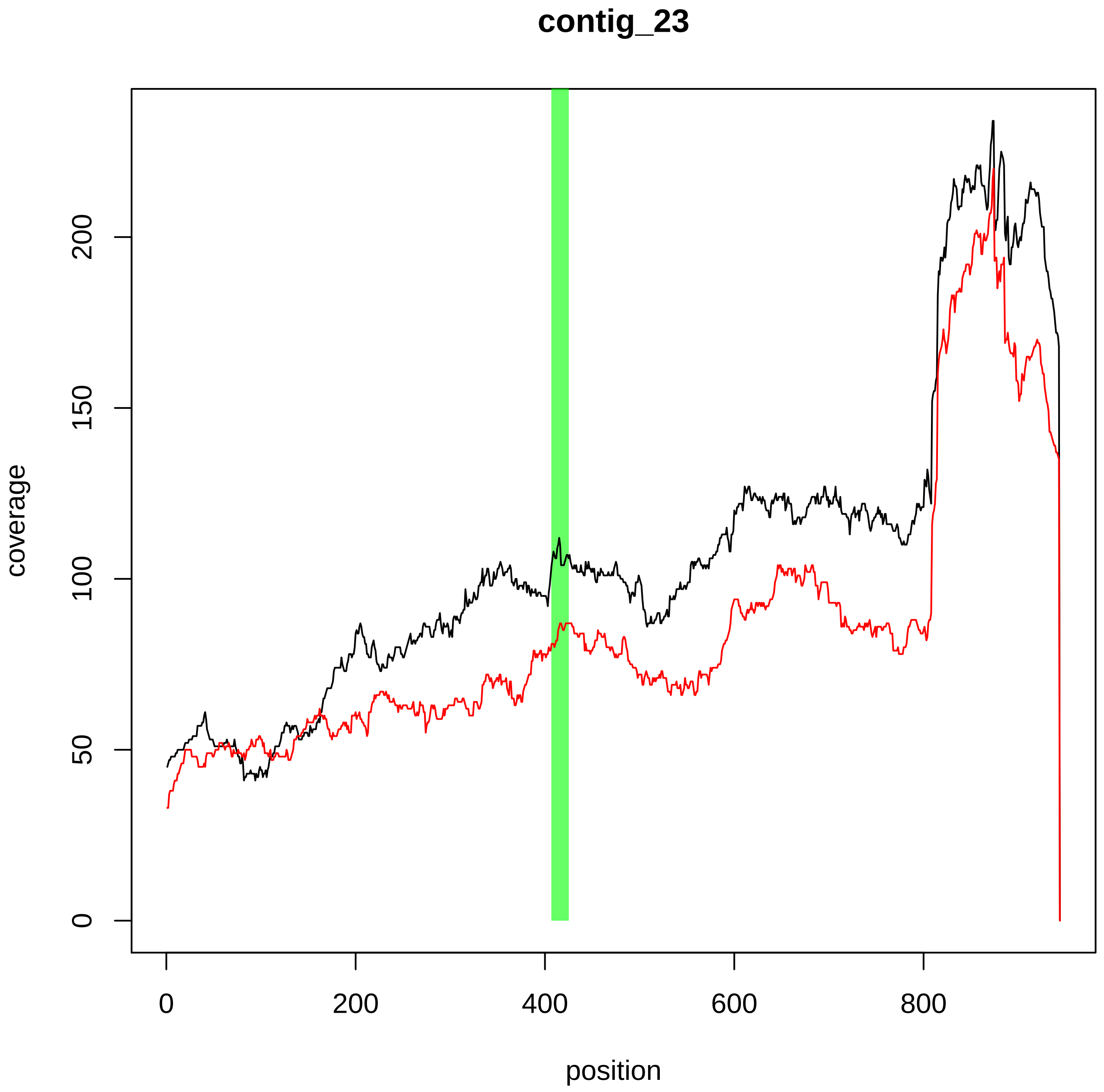
contig_23
coverage
0
50
100
150
200
position
0
200
400
600
800

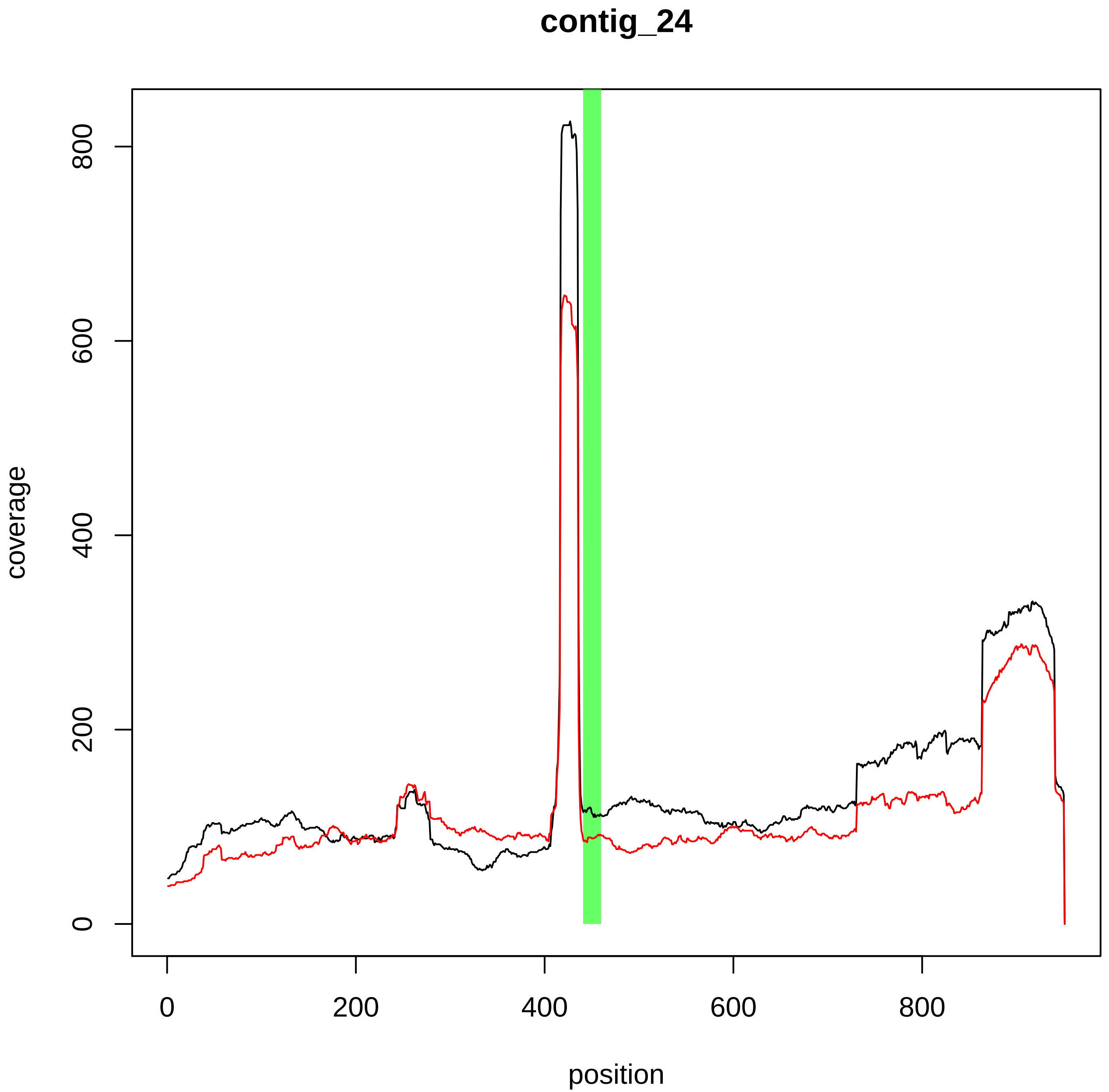
contig_24
coverage
position
0
200
400
600
800
0
200
400
600
800

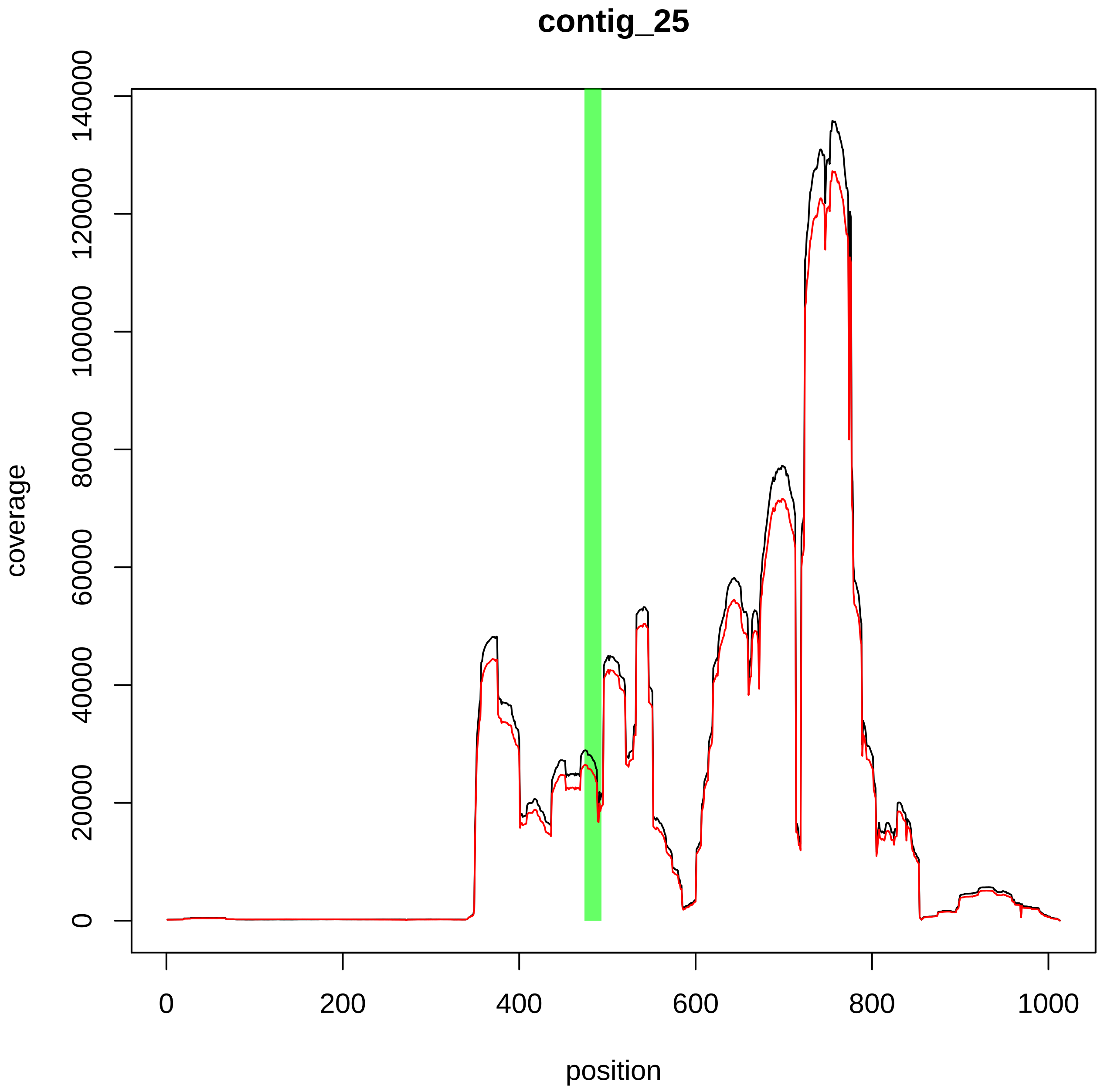

contig_25
coverage
0
20000
40000
60000
80000
100000
120000
140000
0
200
400
600
800
1000
position

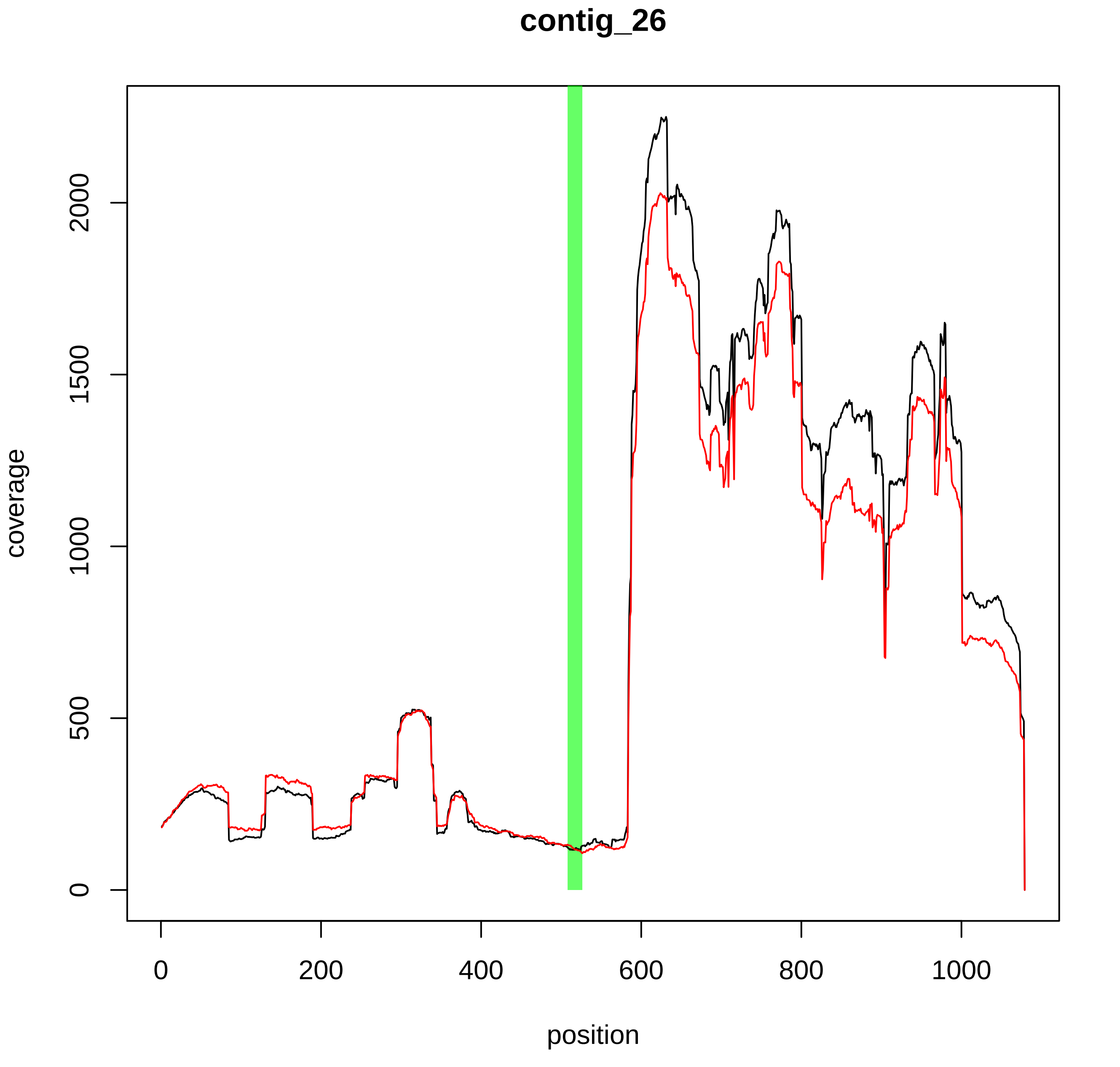
contig_26
coverage
position
0
500
1000
1500
2000
0
200
400
600
800
1000

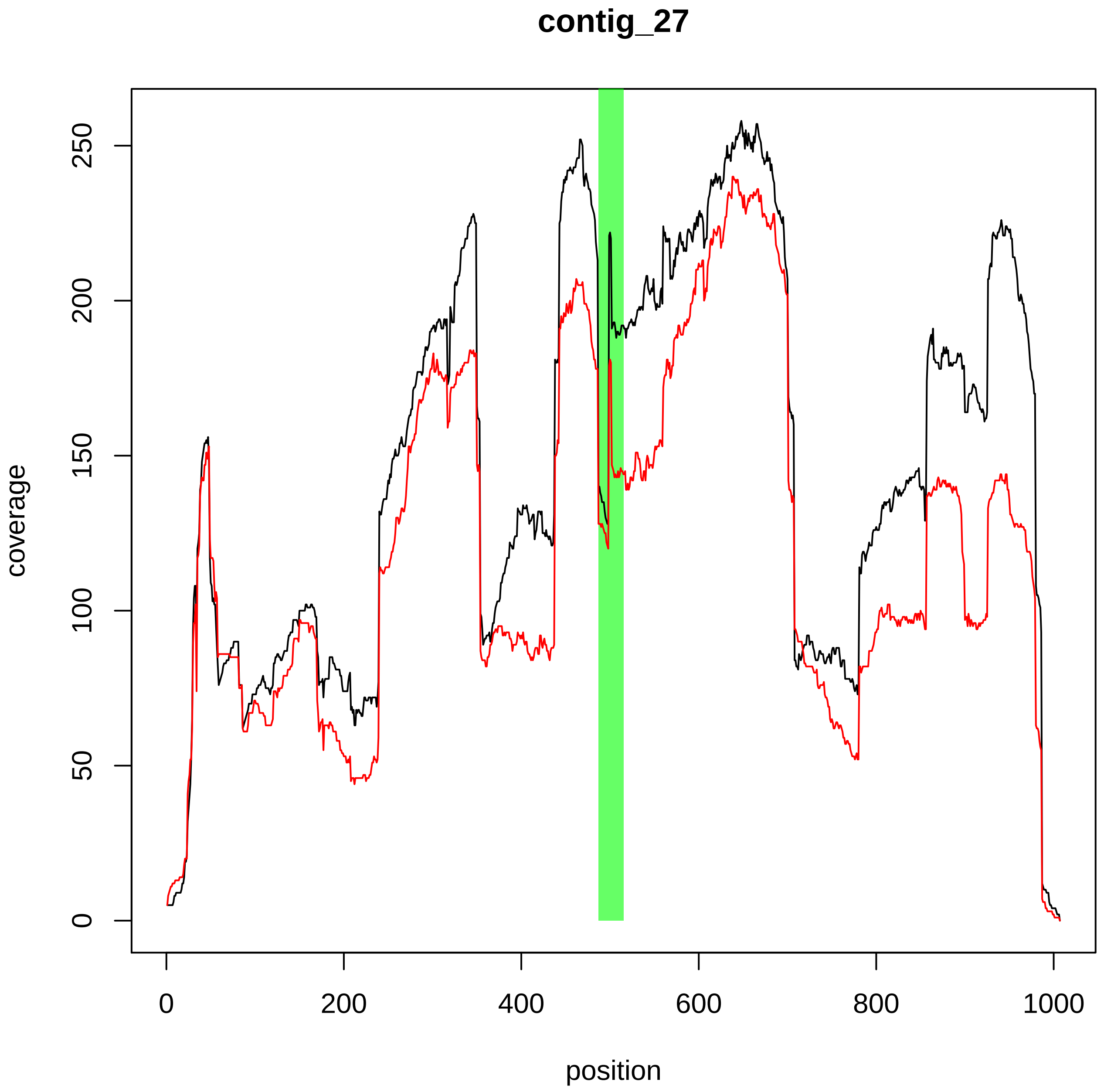

contig_27
coverage
position
0
50
100
150
200
250
0
200
400
600
800
1000

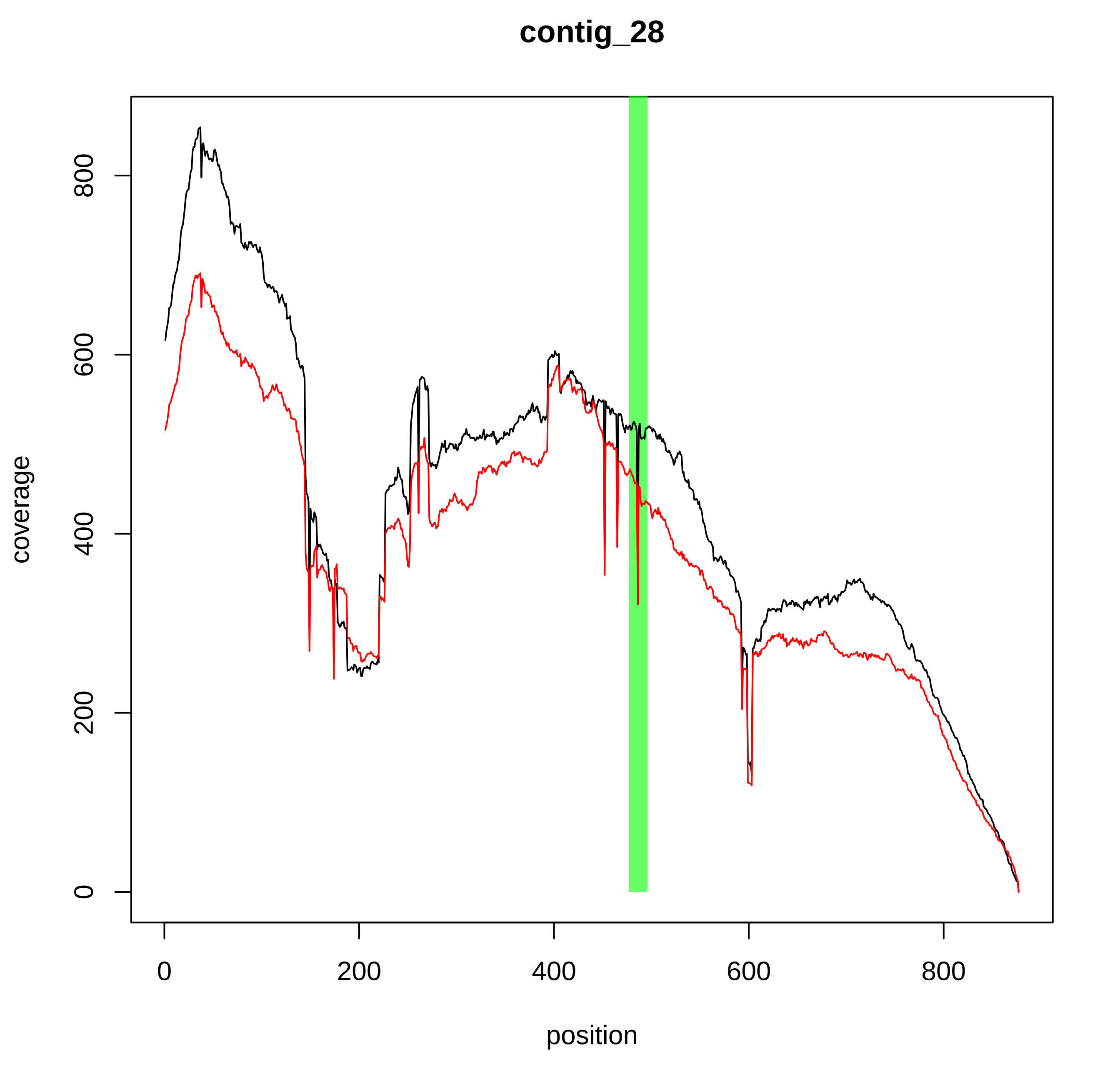
contig_28
coverage
0
200
400
600
800
position
0
200
400
600
800

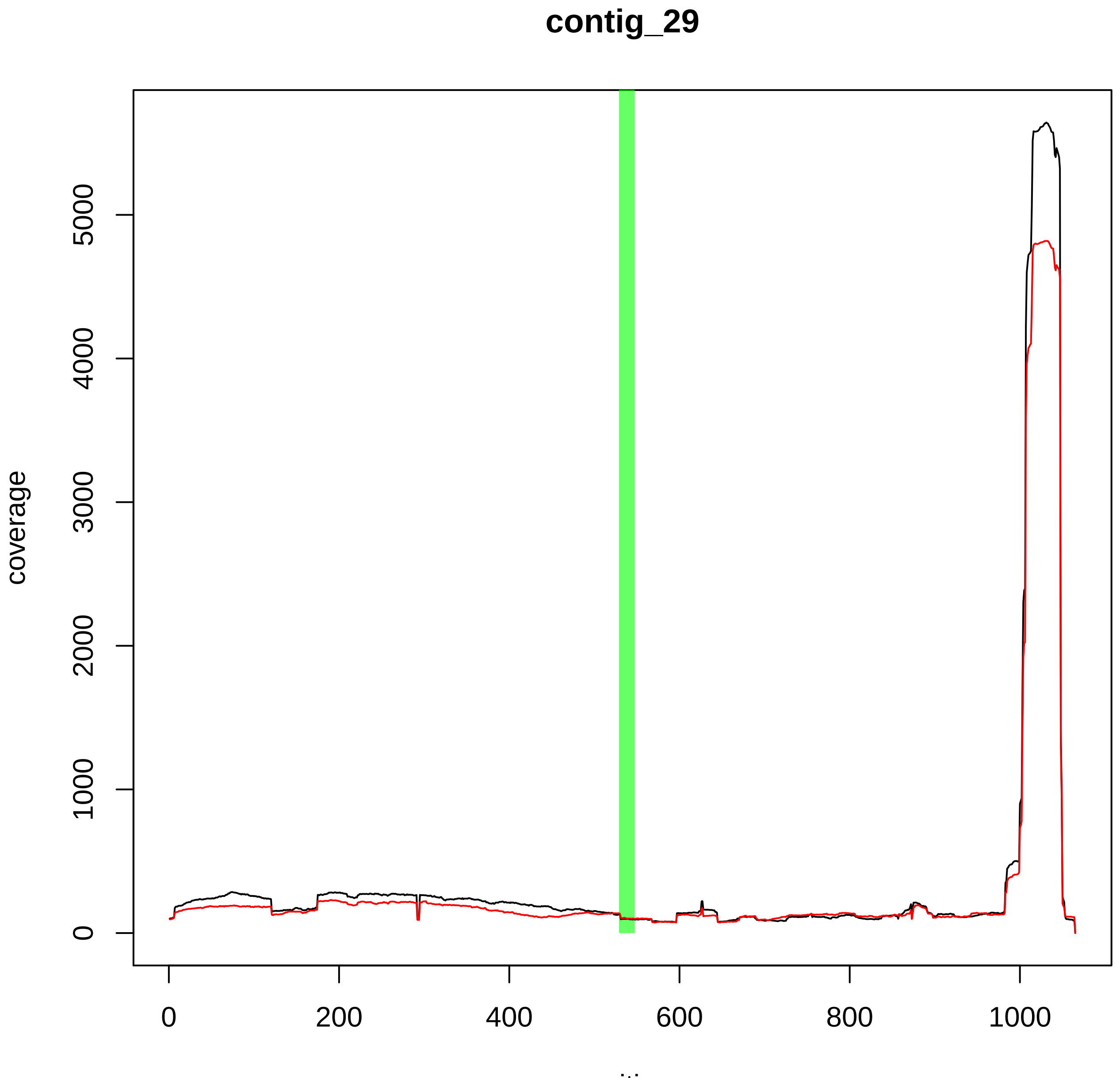
contig_29
coverage
0
1000
2000
3000
4000
5000
0
200
400
600
800
1000
position

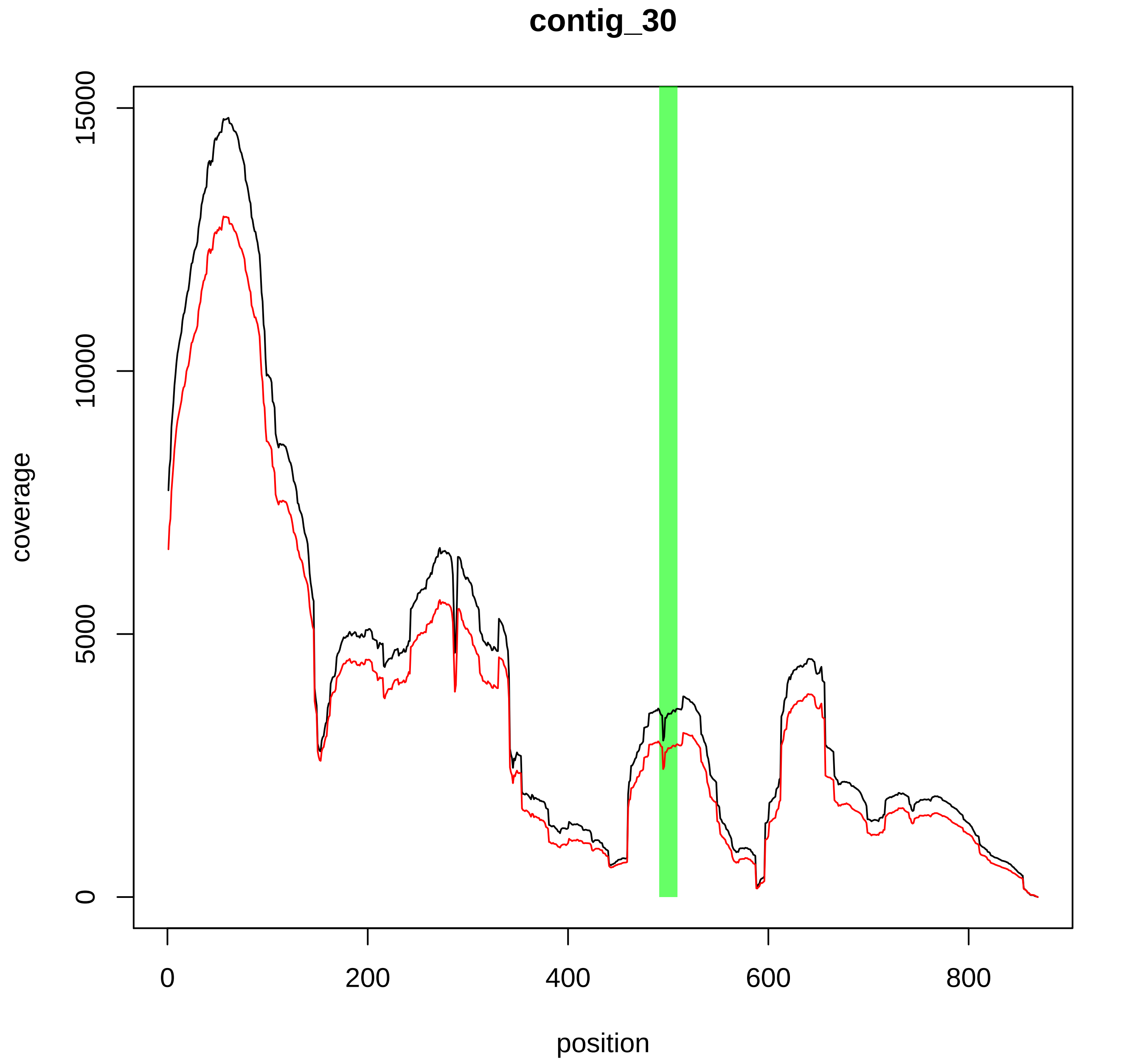
contig_30
coverage
0
5000
10000
15000
position
0
200
400
600
800

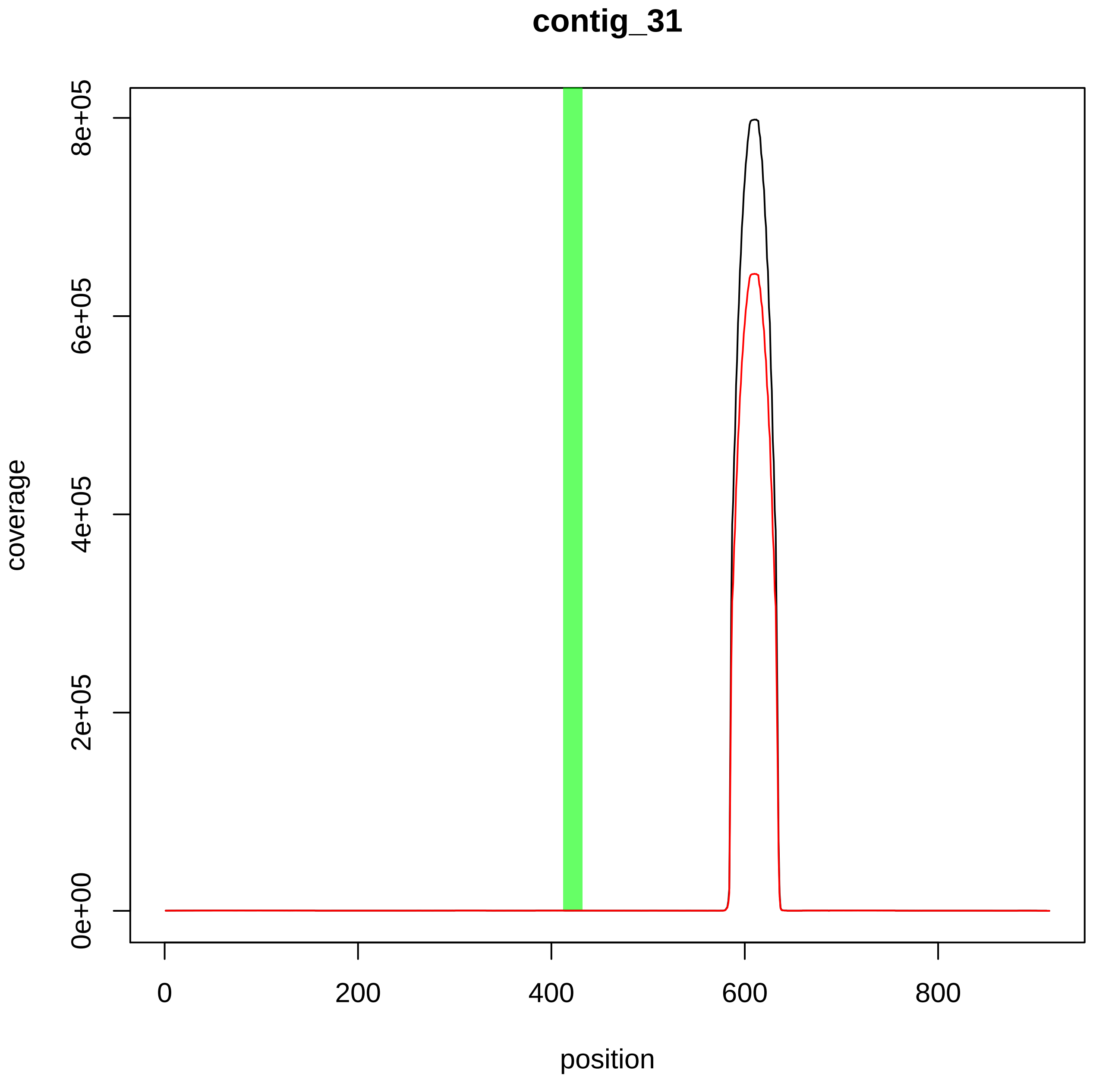

contig_31
coverage
0e+00
2e+05
4e+05
6e+05
8e+05
position
0
200
400
600
800

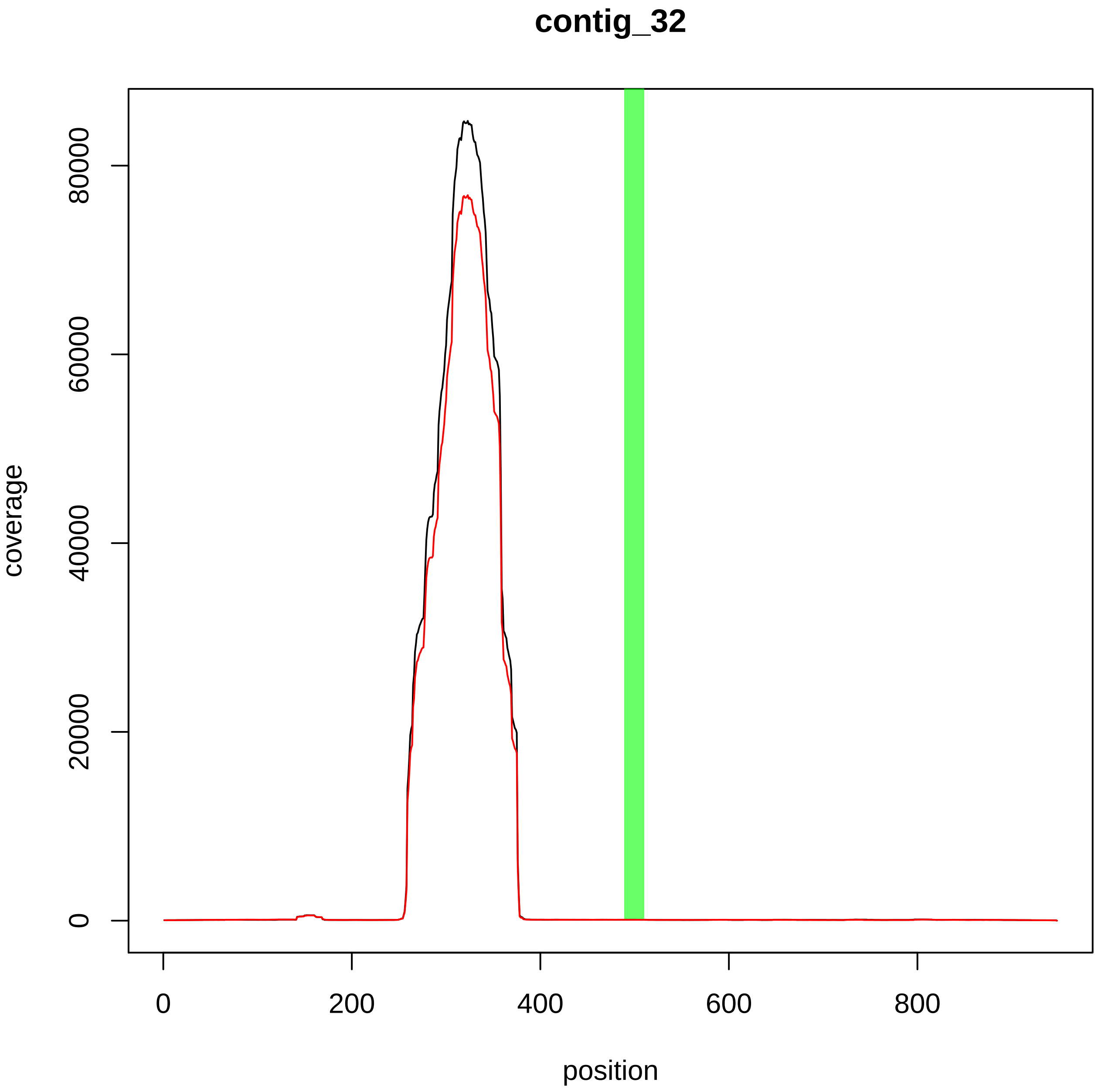

contig_32
coverage
0
20000
40000
60000
80000
position
0
200
400
600
800

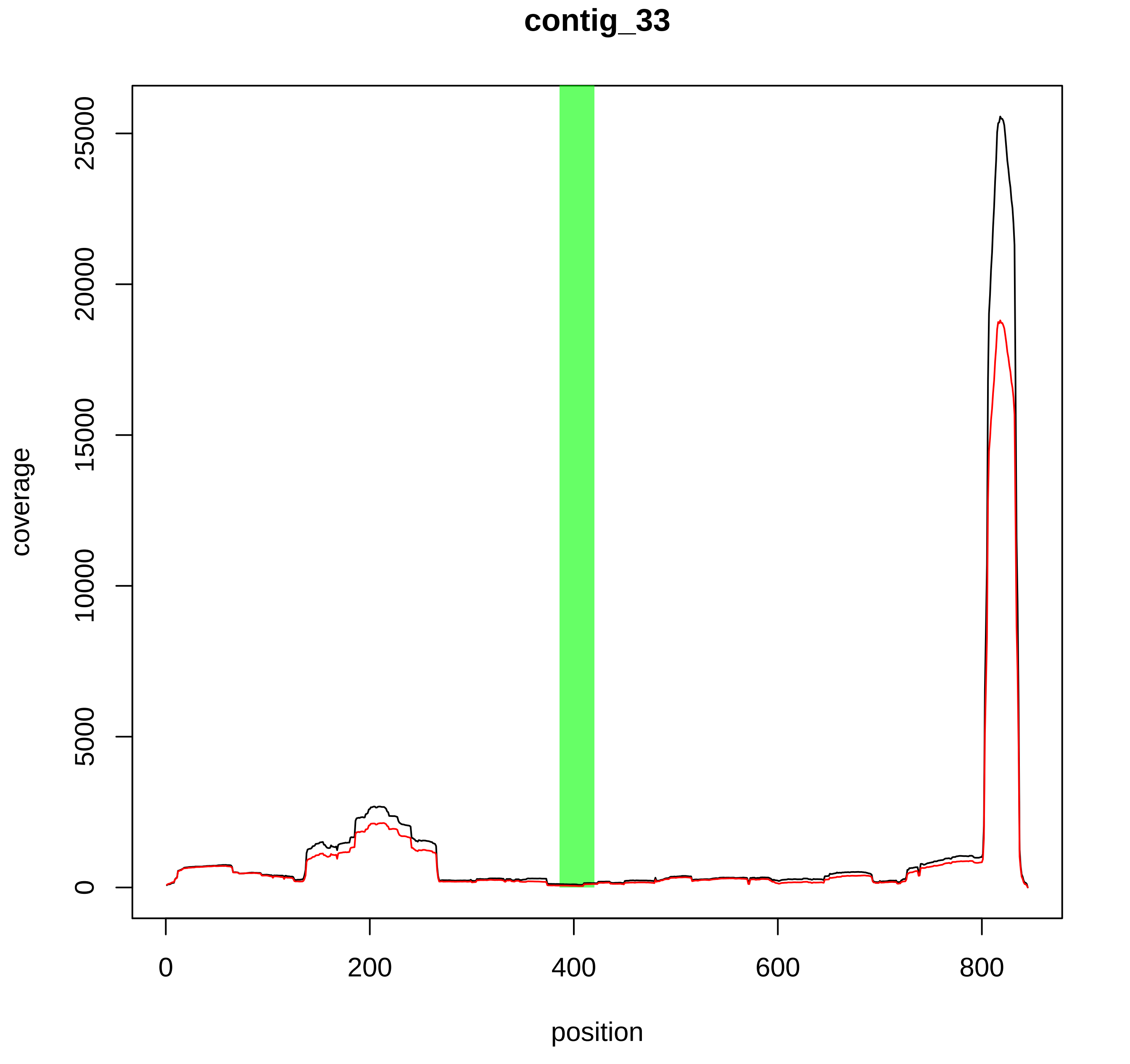
contig_33
coverage
0
5000
10000
15000
20000
25000
0
200
400
600
800
position

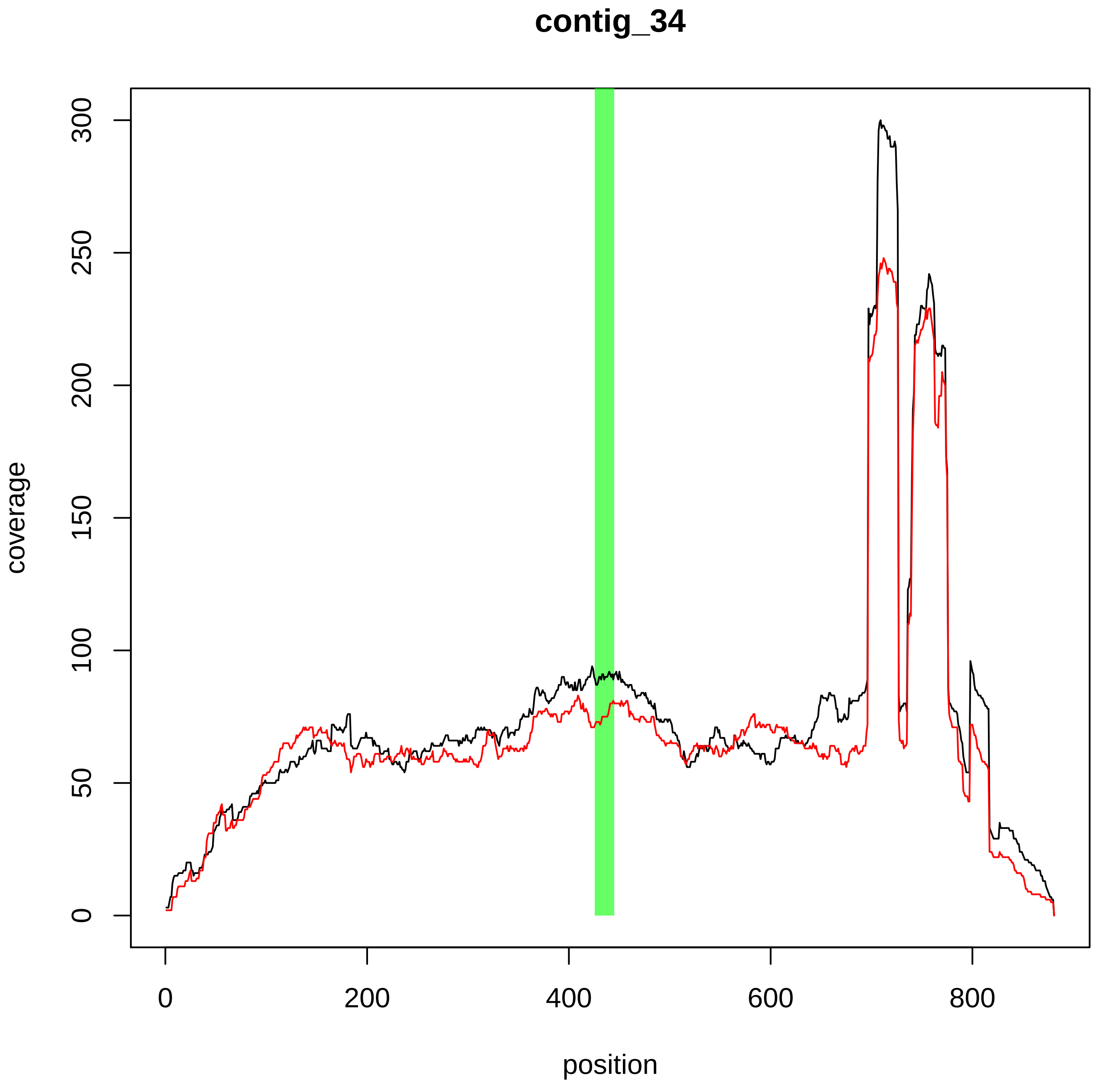
contig_34
coverage
0
50
100
150
200
250
300
position
0
200
400
600
800

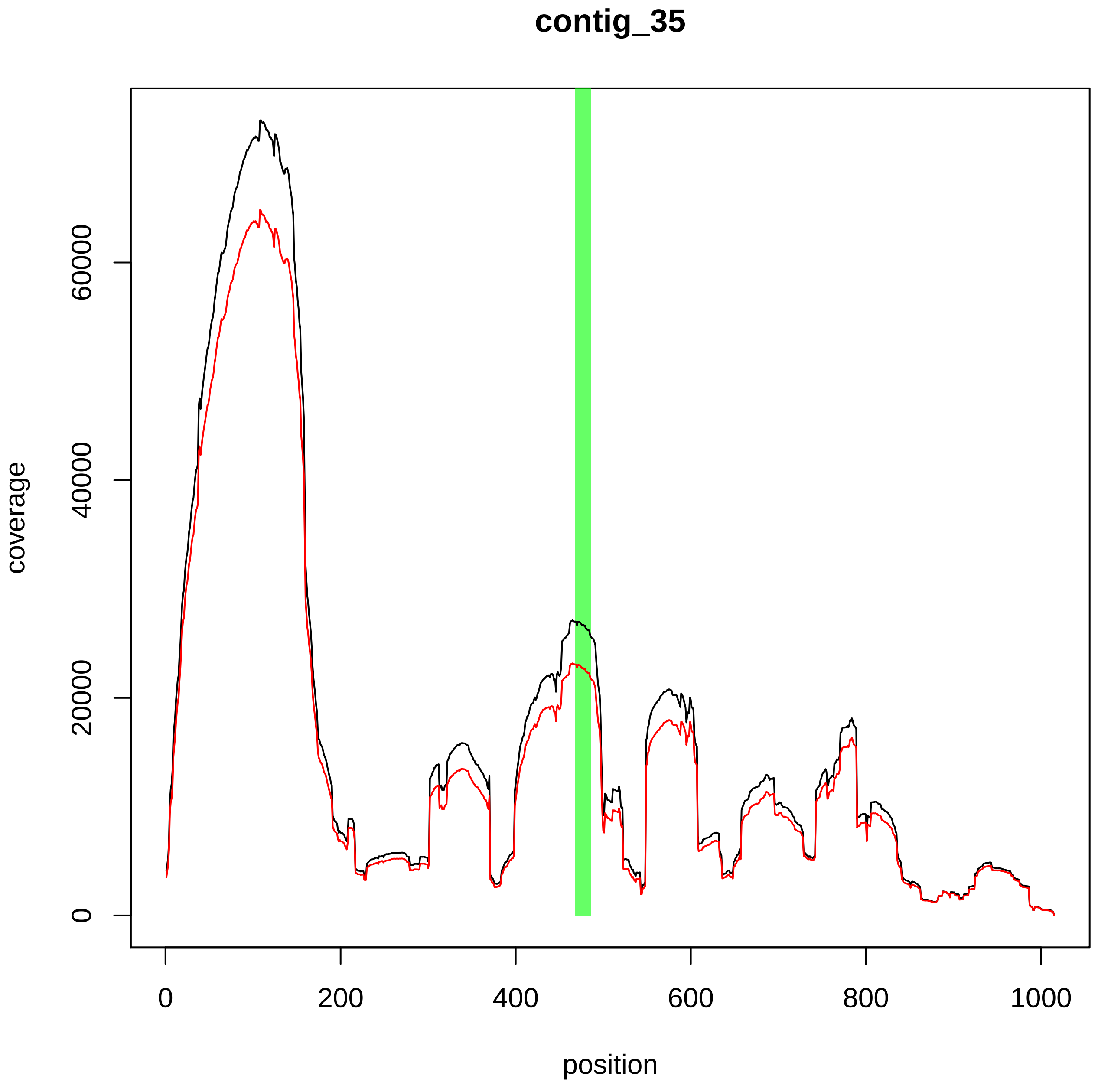

contig_35
coverage
position
0
20000
40000
60000
0
200
400
600
800
1000

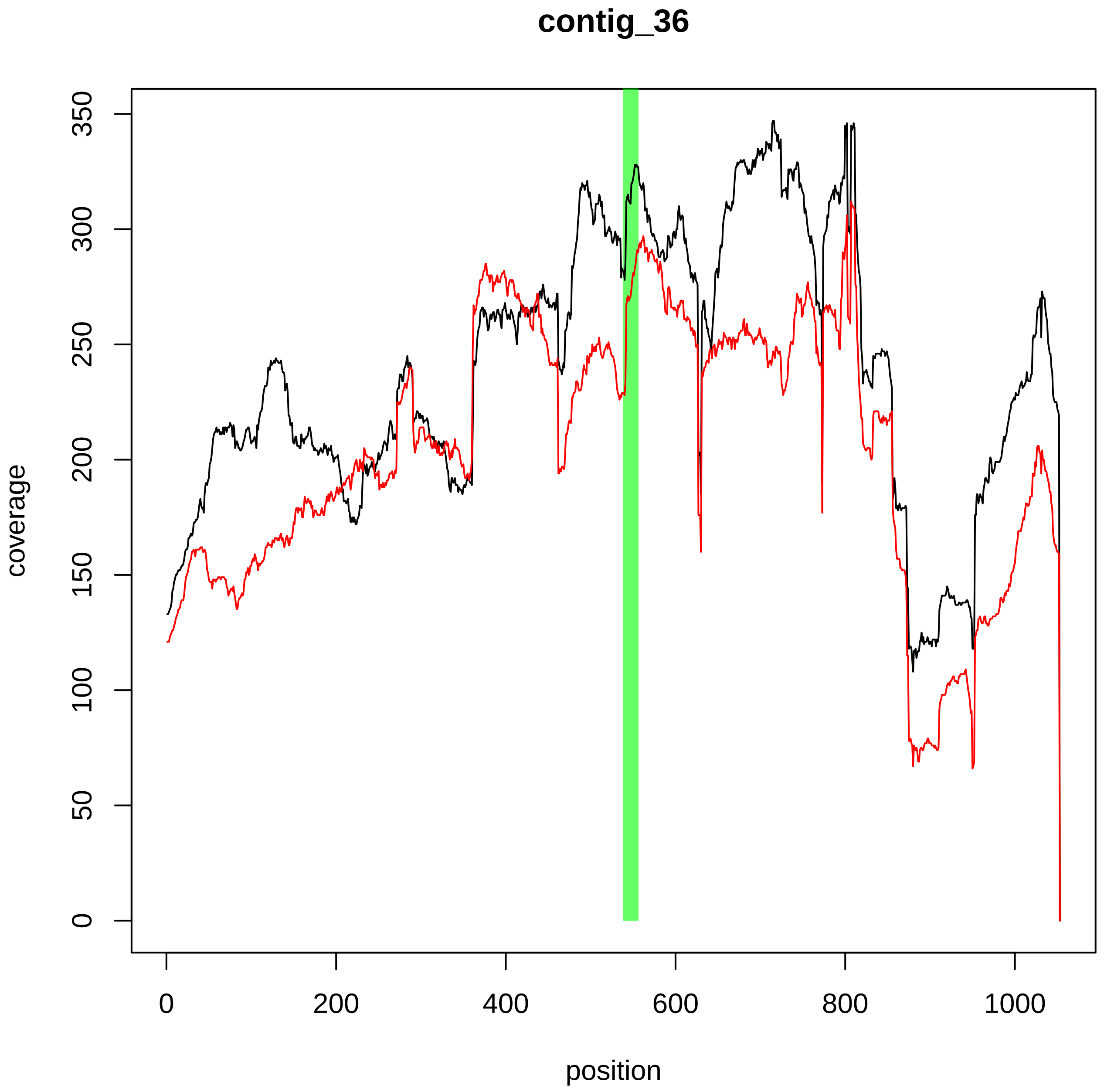

contig_36
coverage
position
0
50
100
150
200
250
300
350
0
200
400
600
800
1000

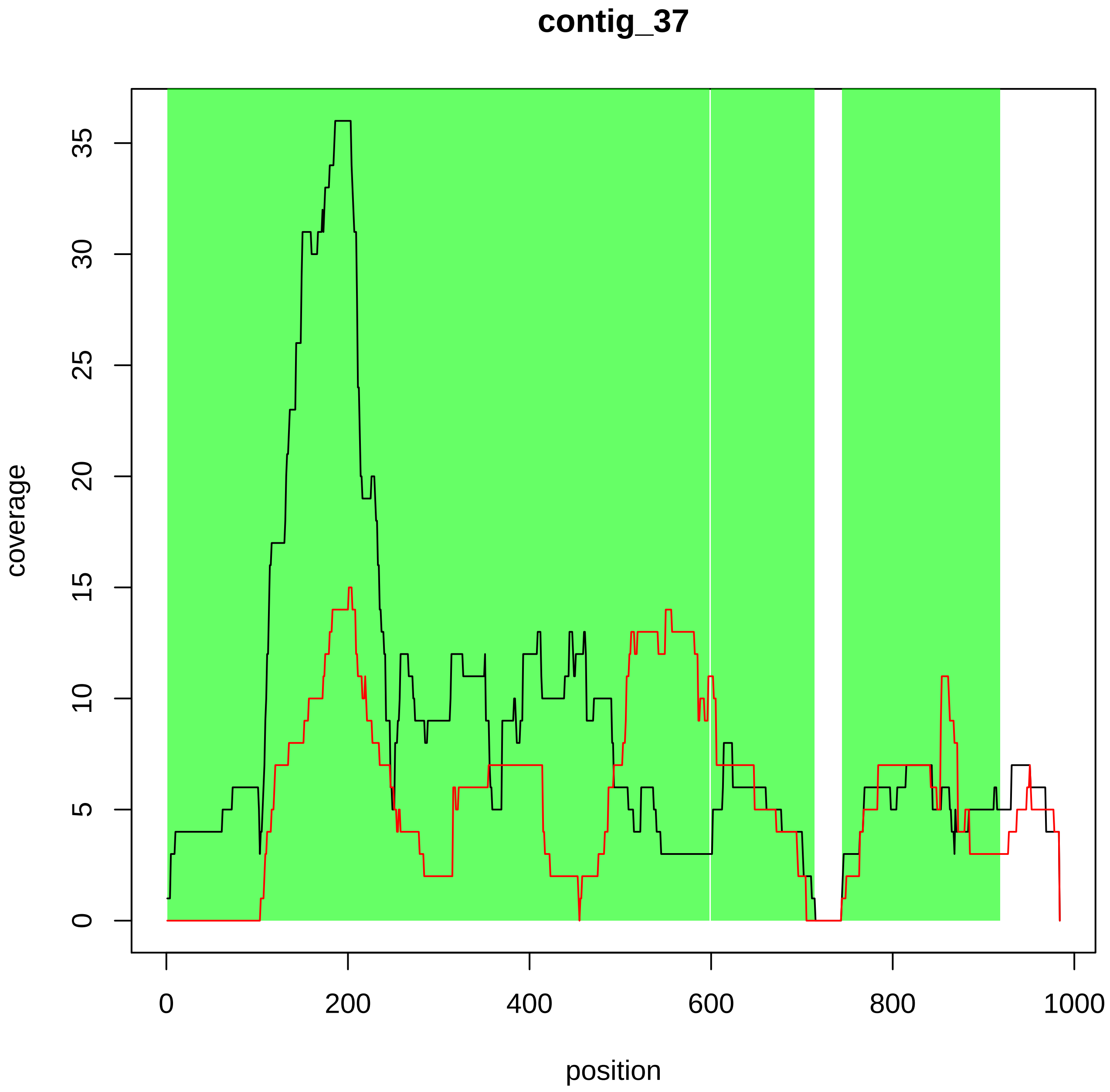
contig_37
coverage
position
0
5
10
15
20
25
30
35
0
200
400
600
800
1000

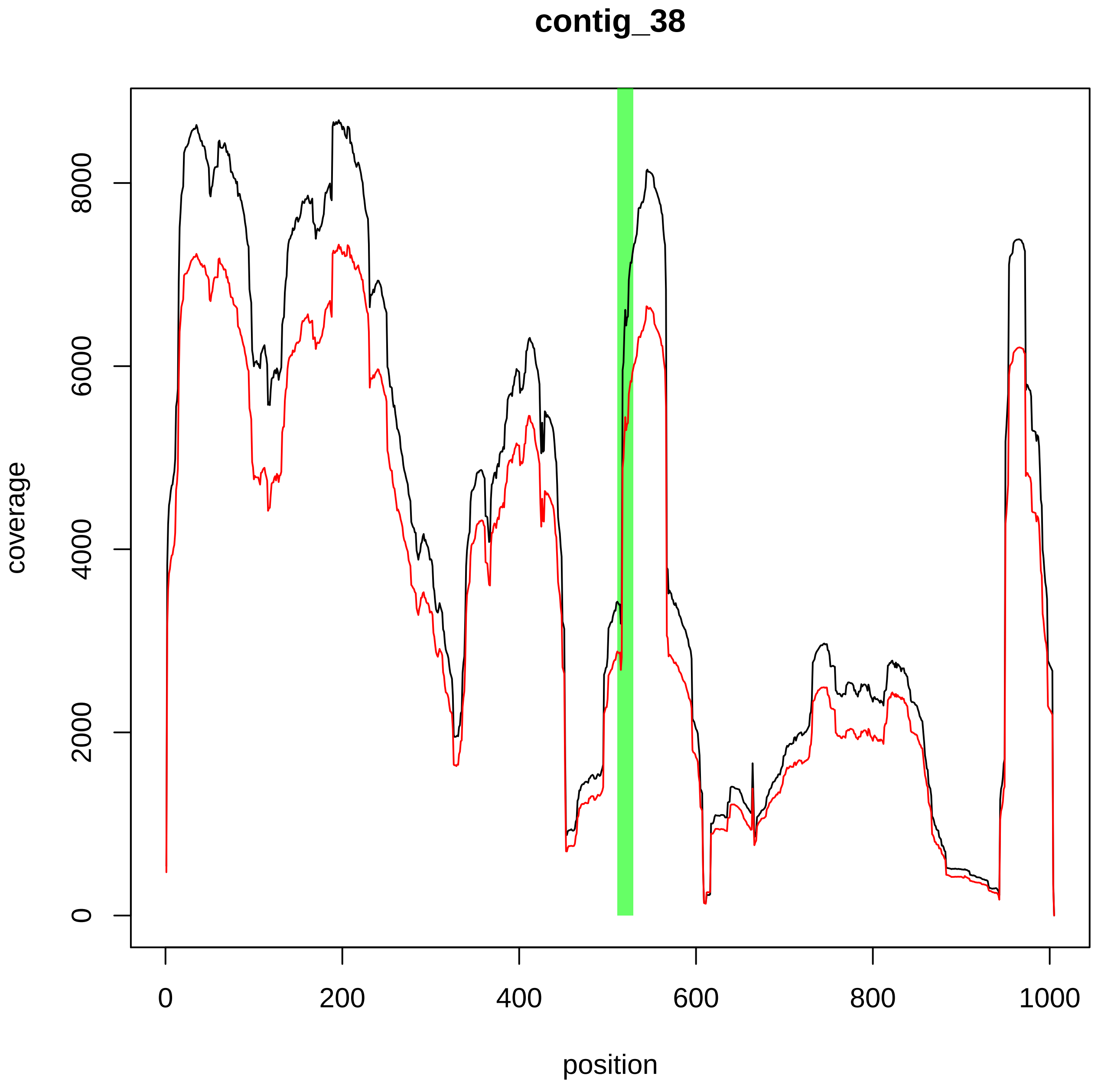
contig_38
coverage
position
0
2000
4000
6000
8000
0
200
400
600
800
1000

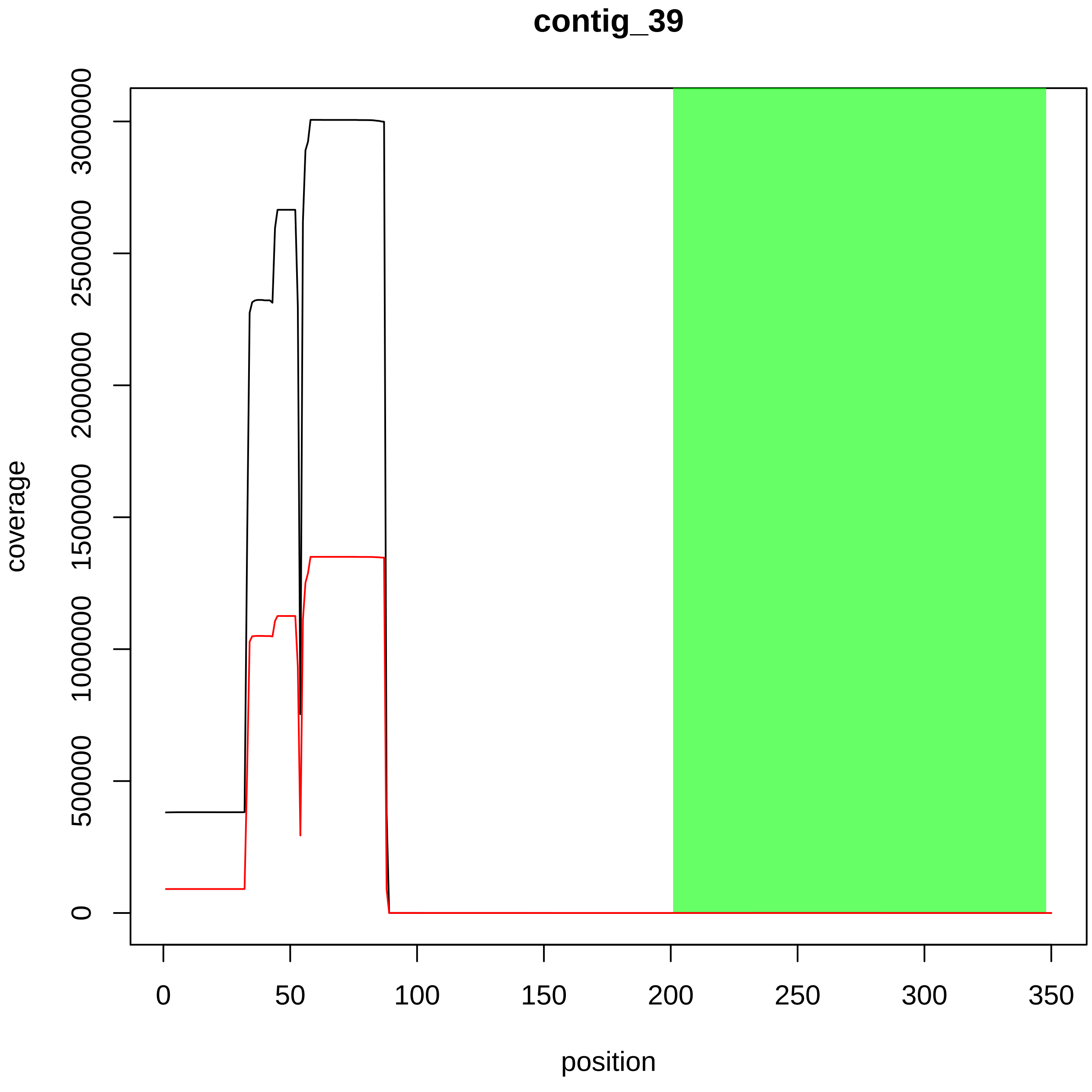

contig_39
coverage
0
500000
1000000
1500000
2000000
2500000
3000000
position
0
50
100
150
200
250
300
350

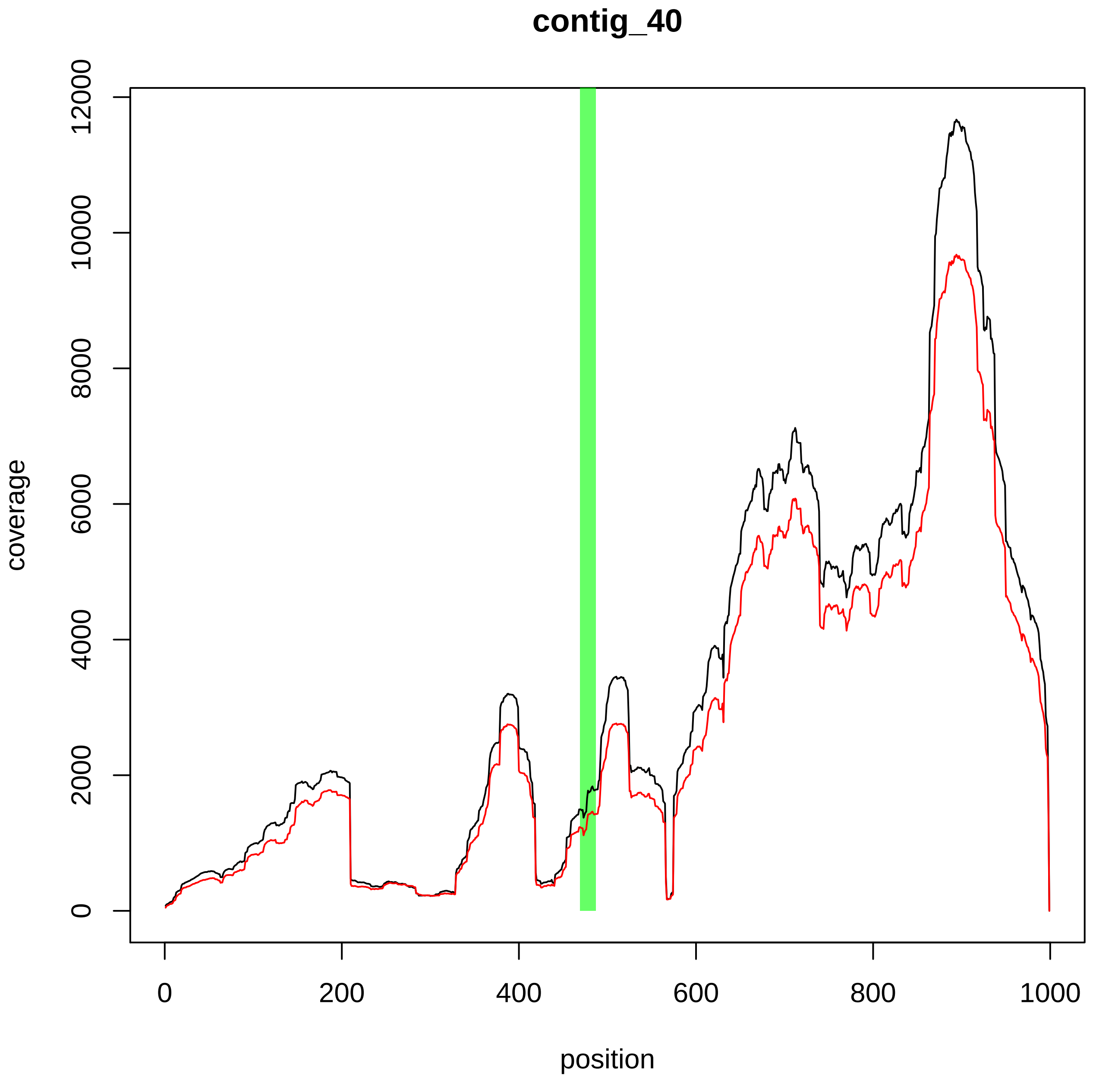

contig_40
coverage
0
2000
4000
6000
8000
10000
12000
position
0
200
400
600
800
1000

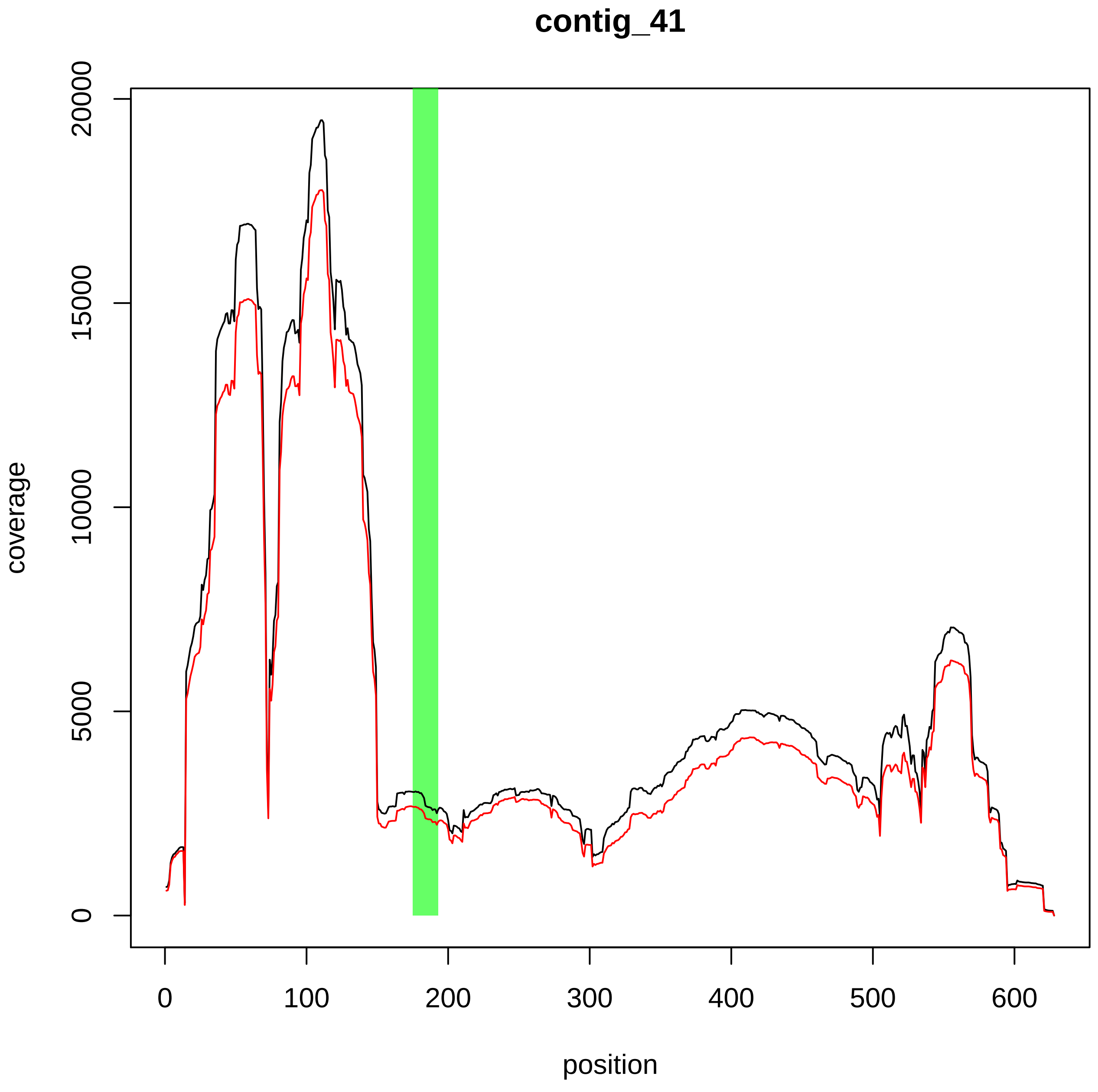
contig_41
coverage
position
0
5000
10000
15000
20000
0
100
200
300
400
500
600

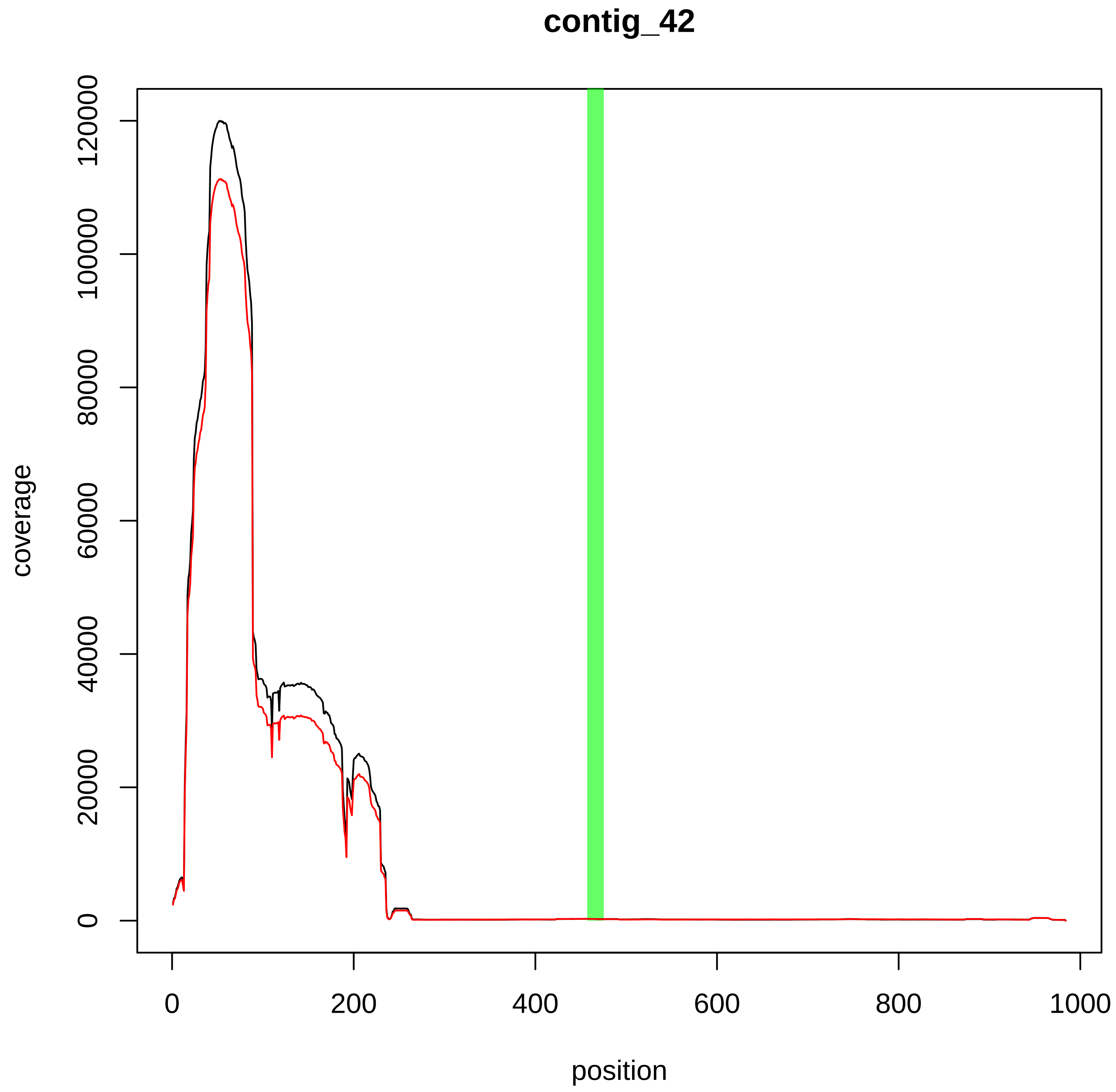

contig_42
coverage
position
0
20000
40000
60000
80000
100000
120000
0
200
400
600
800
1000

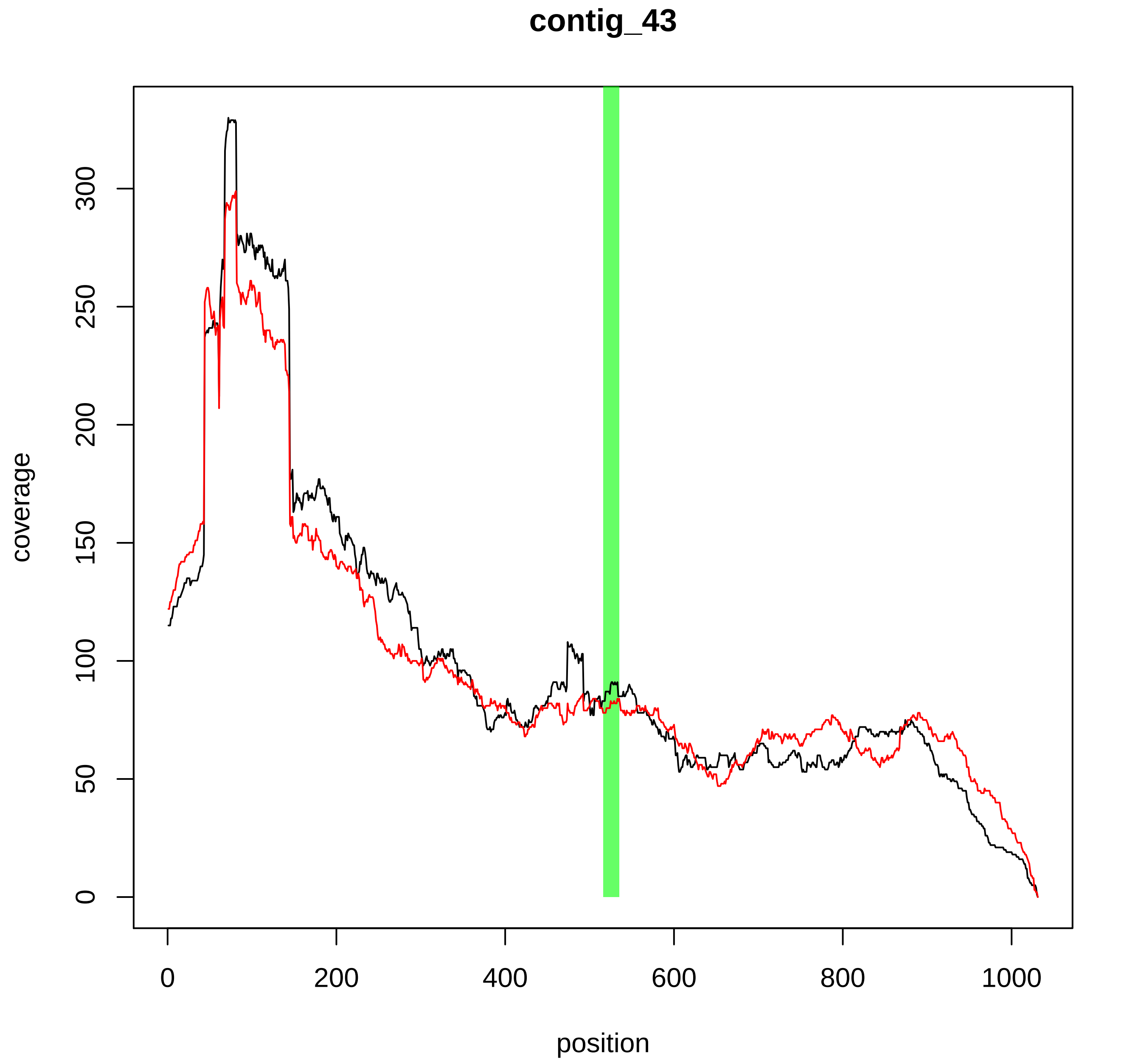
contig_43
coverage
0
50
100
150
200
250
300
position
0
200
400
600
800
1000

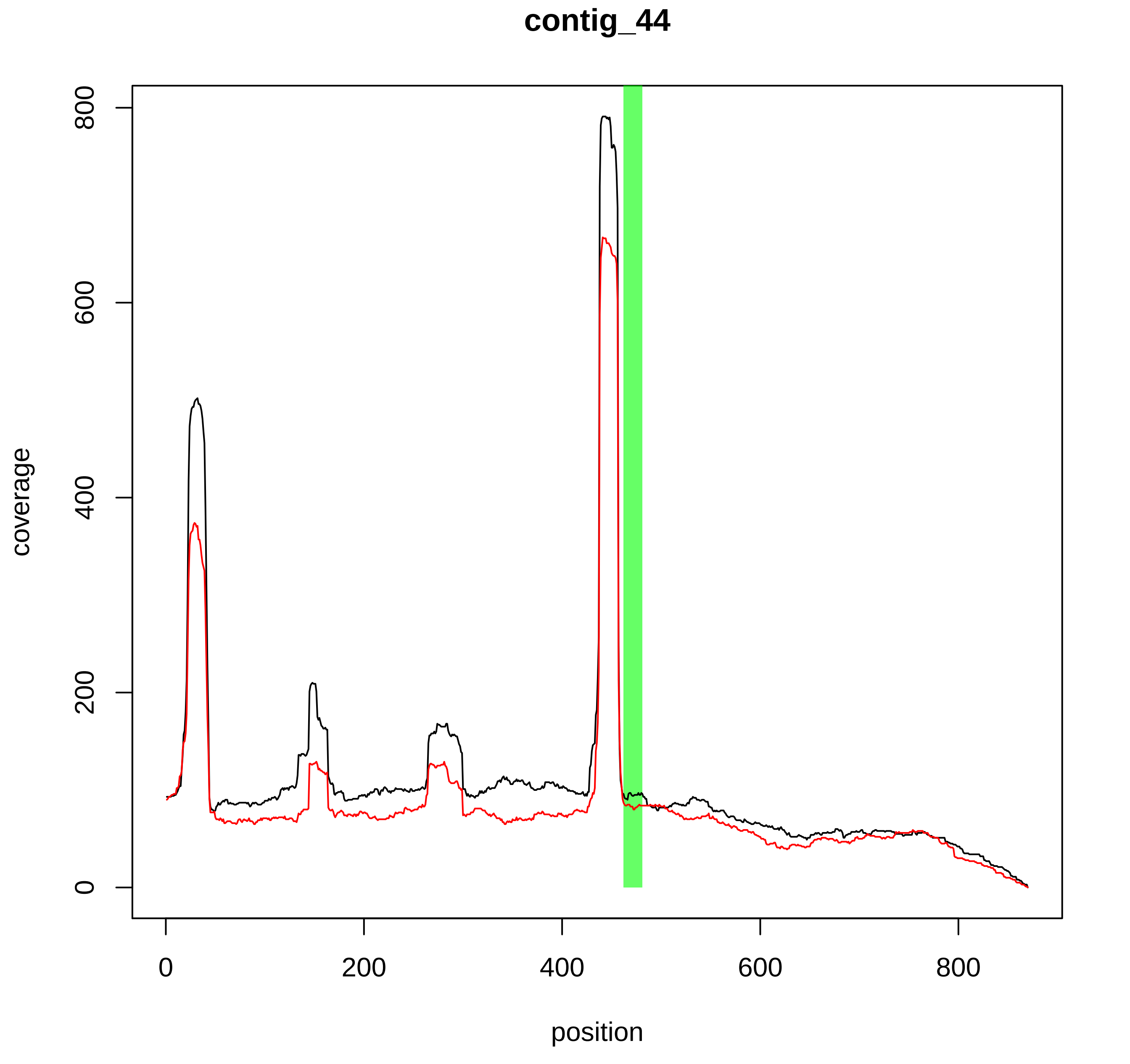

contig_44
coverage
position
0
200
400
600
800
0
200
400
600
800

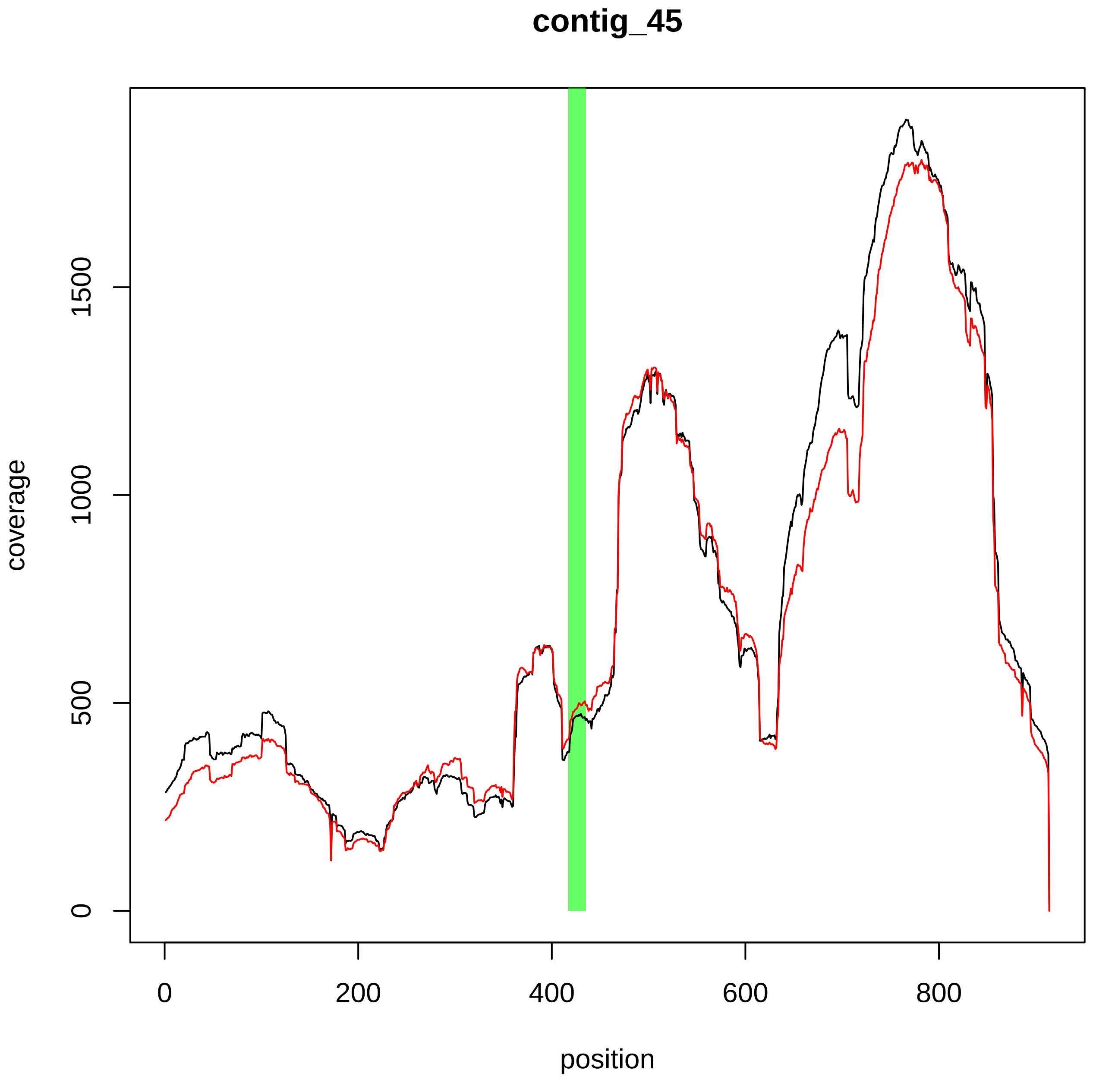

contig_45
coverage
position
0
500
1000
1500
0
200
400
600
800

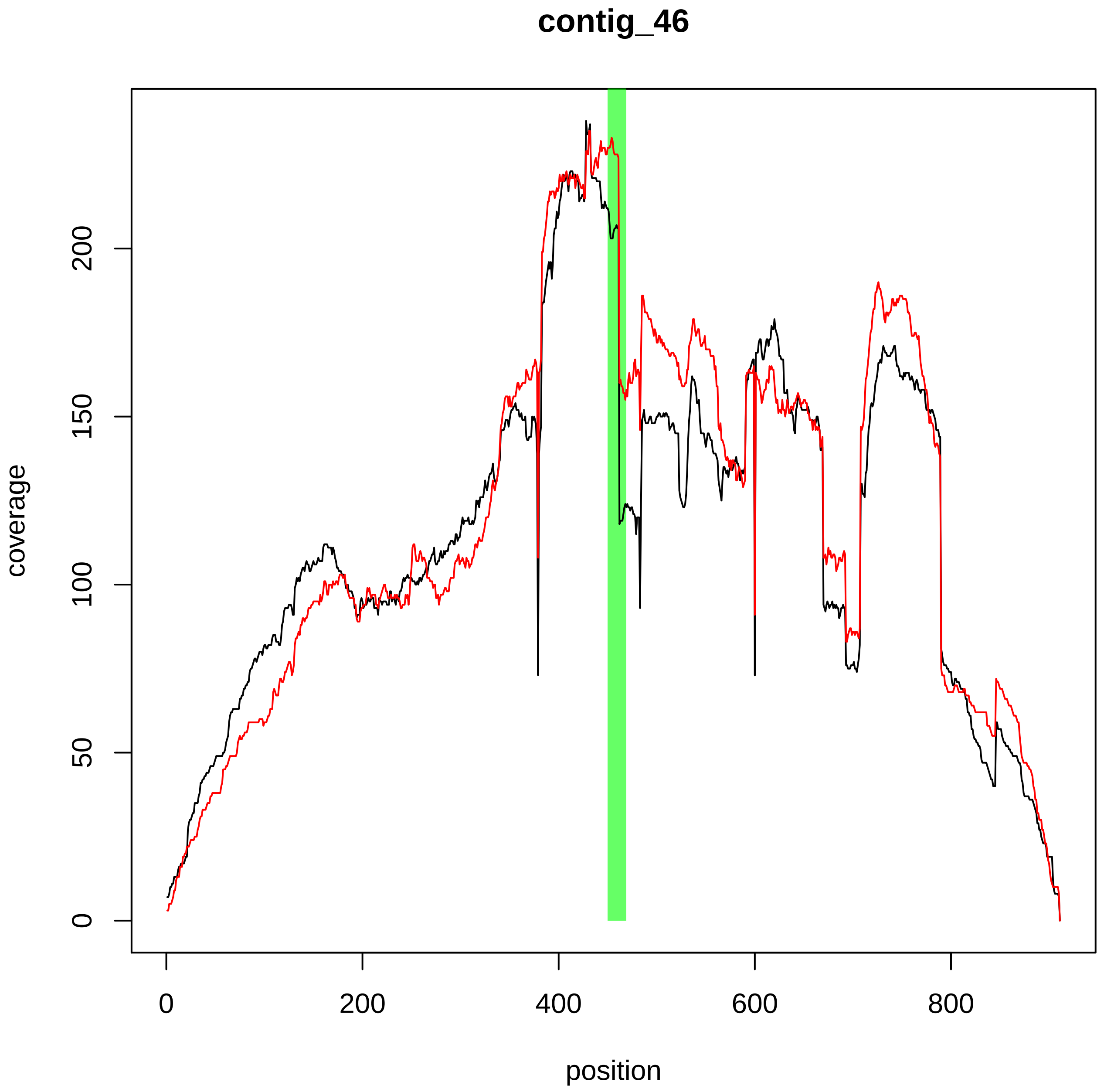
contig_46
coverage
position
0
50
100
150
200
0
200
400
600
800

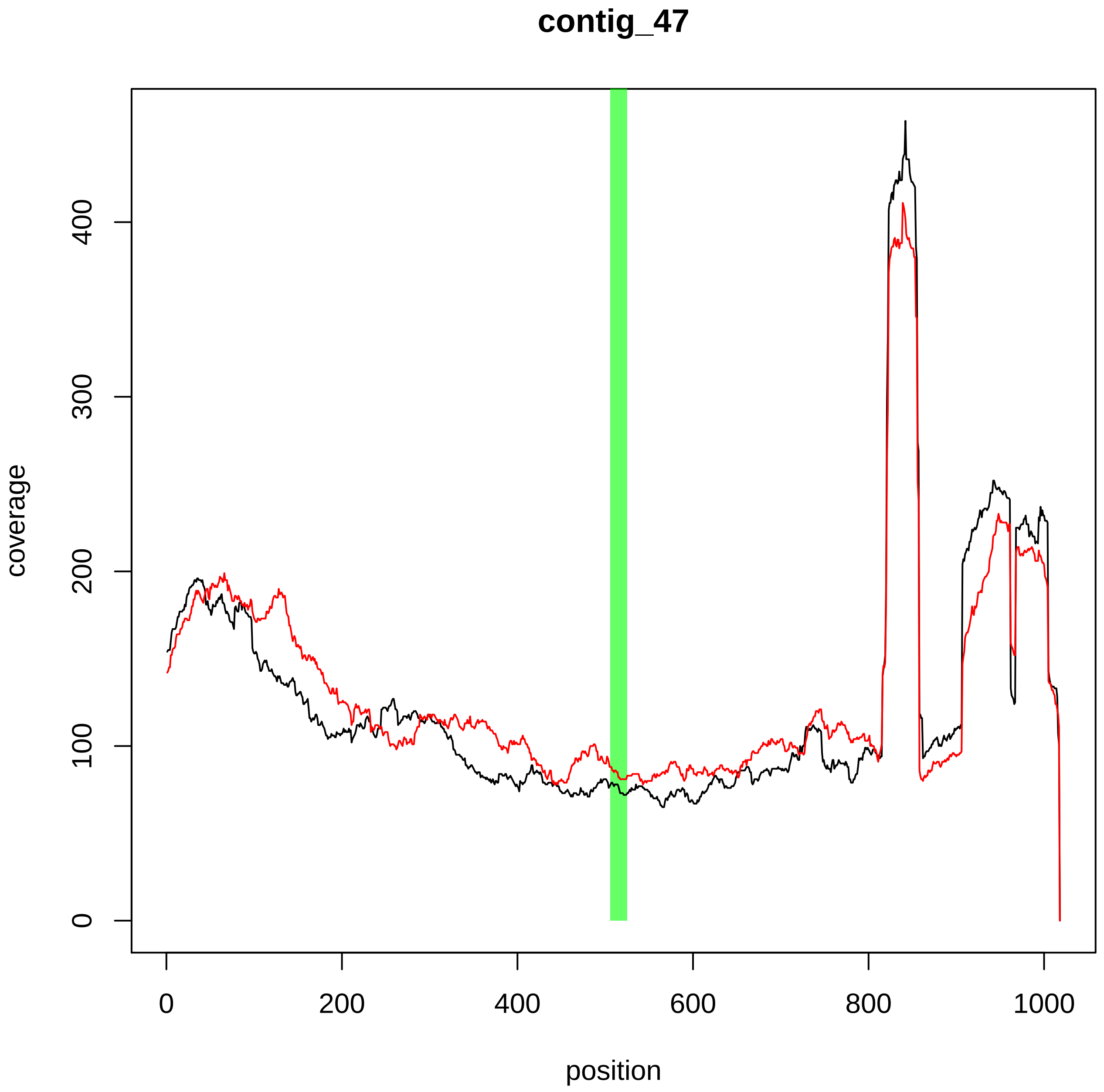
contig_47
coverage
0
100
200
300
400
position
0
200
400
600
800
1000

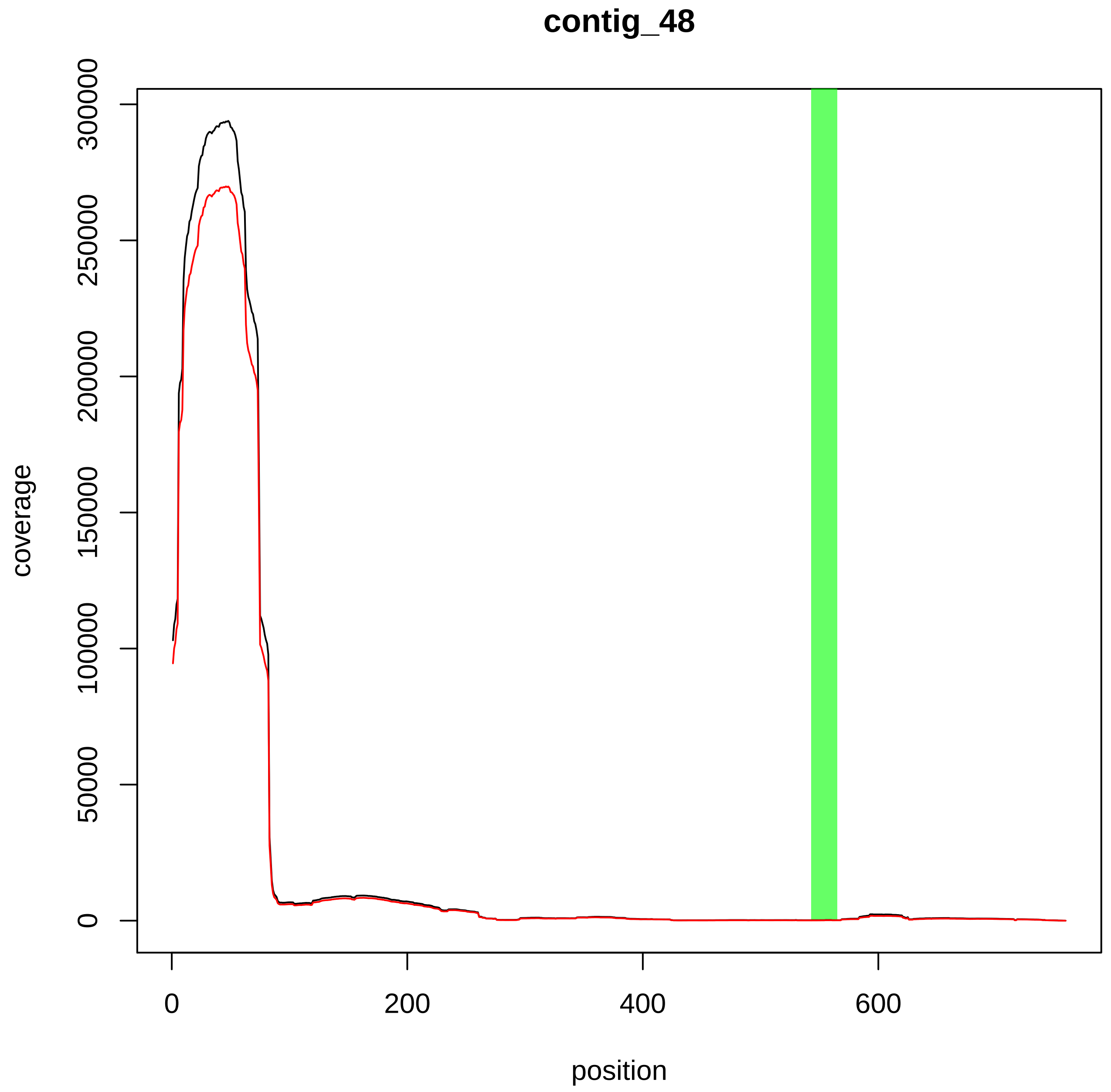
contig_48
coverage
0
50000
100000
150000
200000
250000
300000
0
200
400
600
position

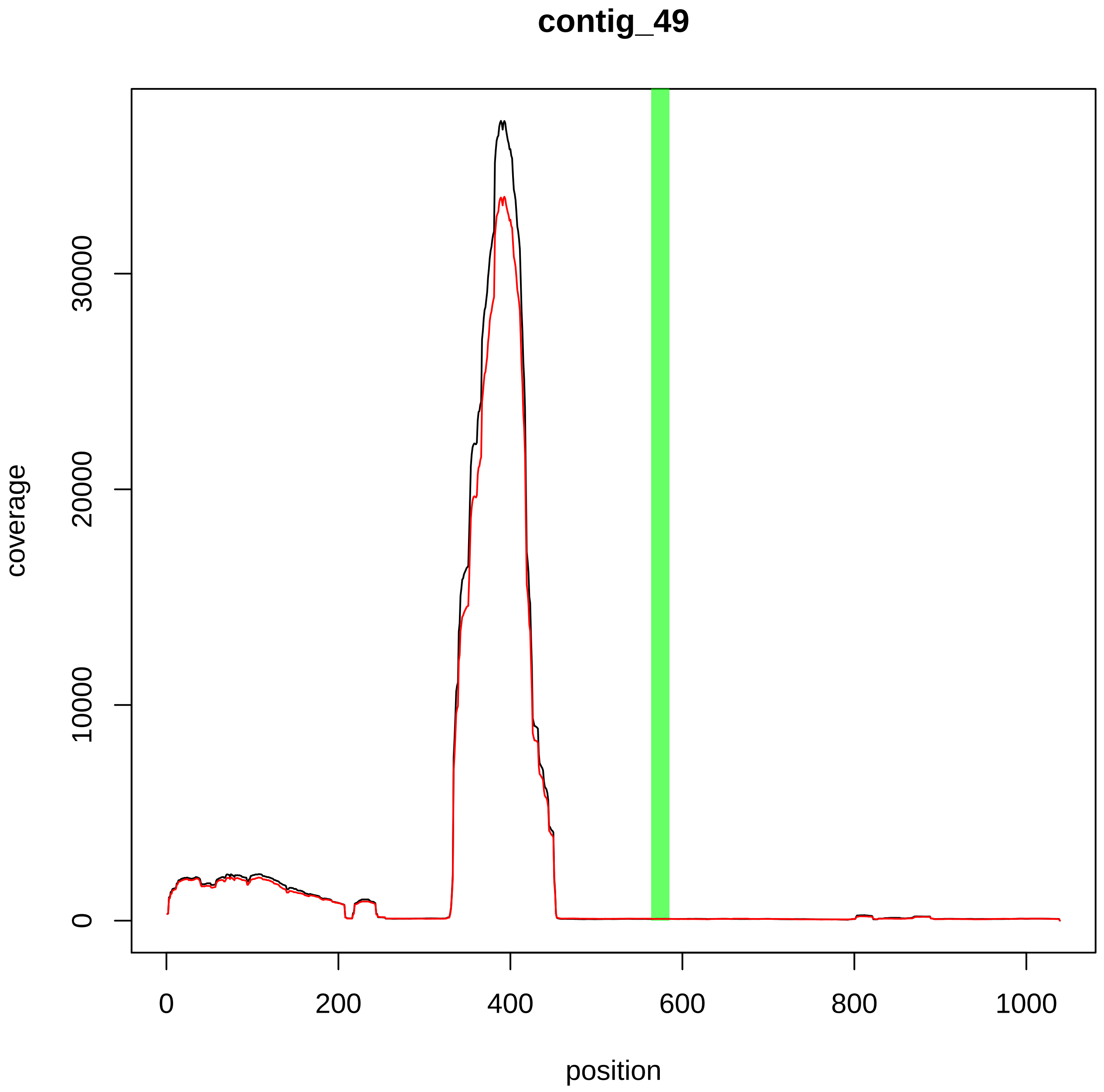

contig_49
coverage
0
10000
20000
30000
position
0
200
400
600
800
1000

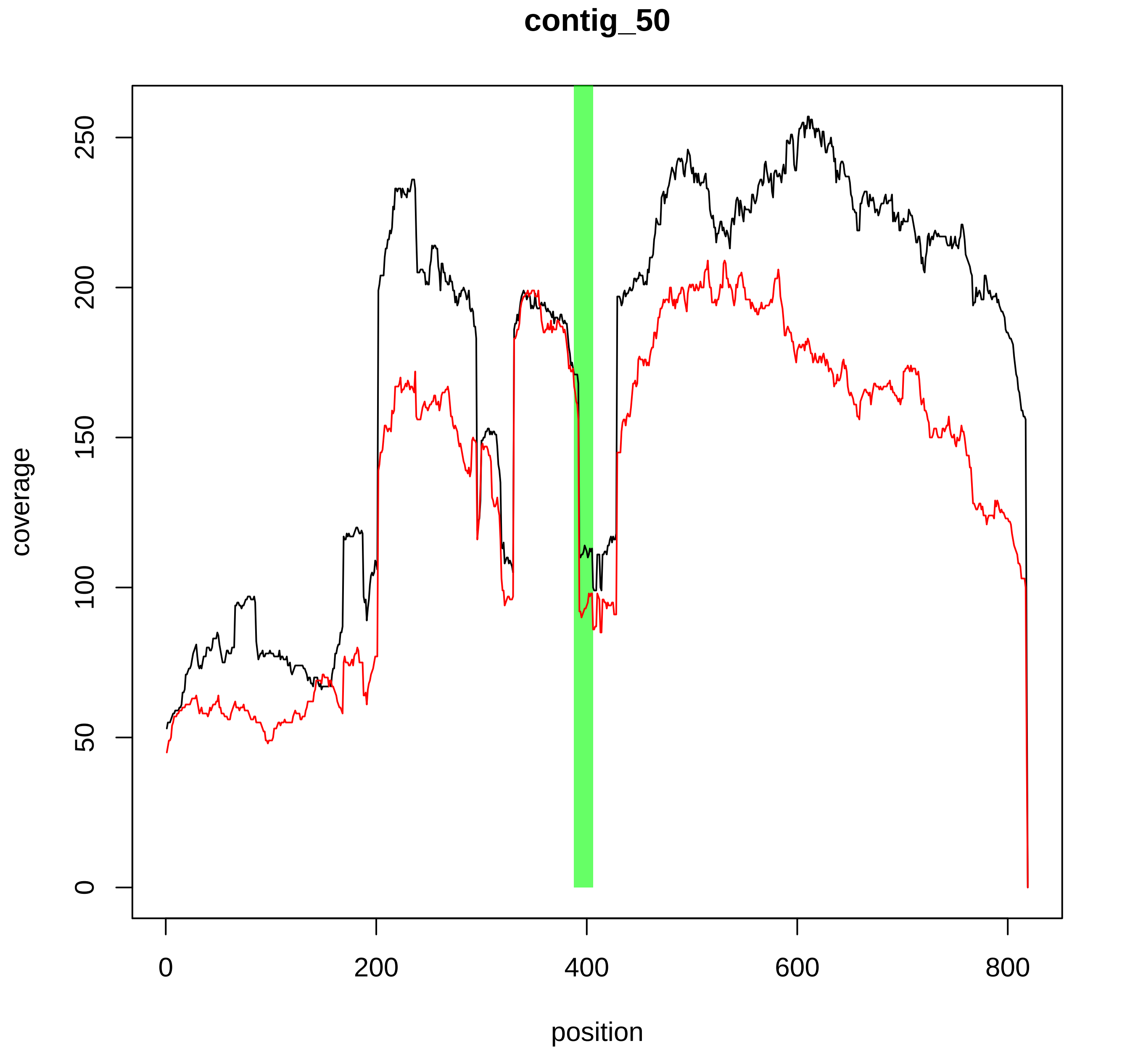
contig_50
coverage
position
0
50
100
150
200
250
0
200
400
600
800

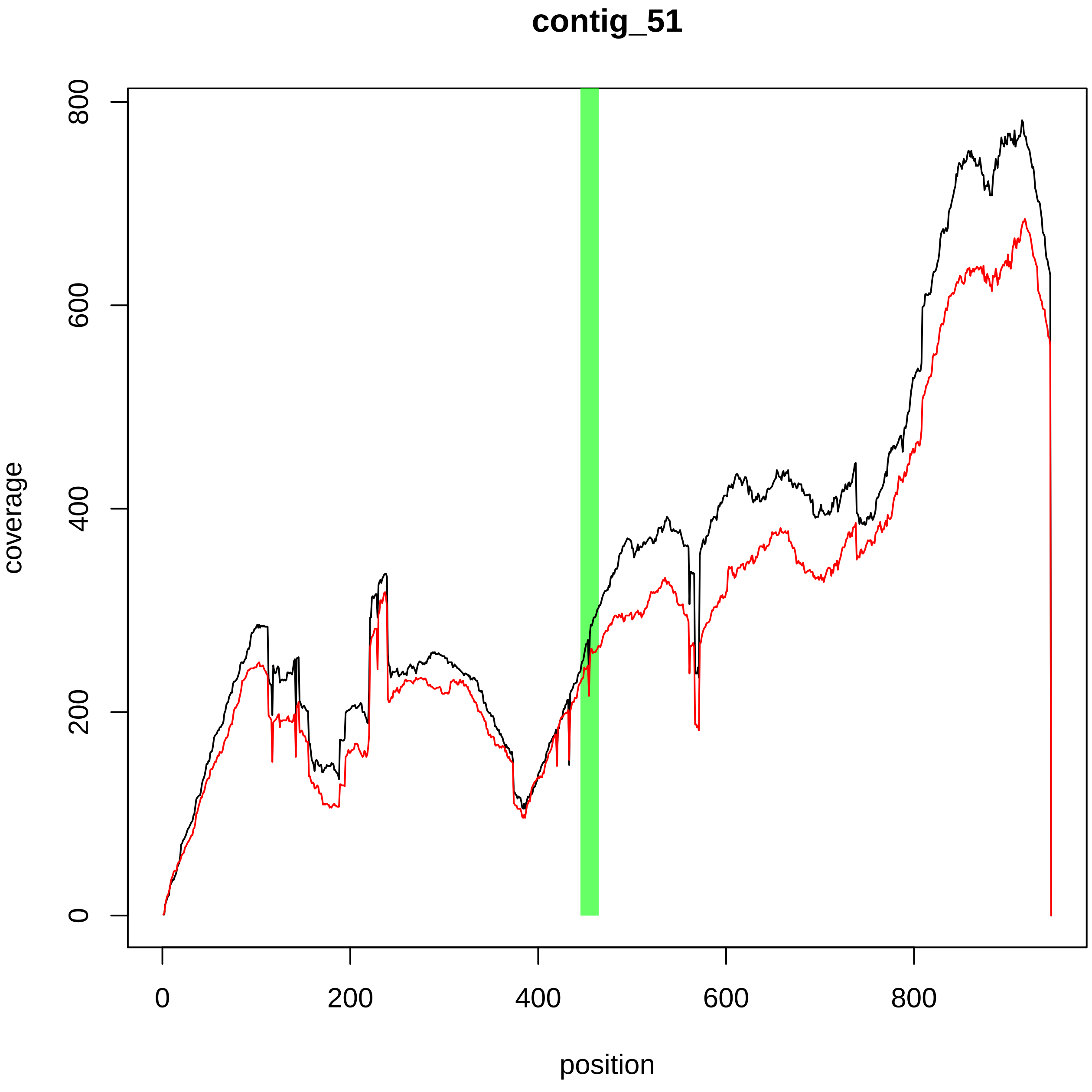
contig_51
coverage
0
200
400
600
800
position
0
200
400
600
800

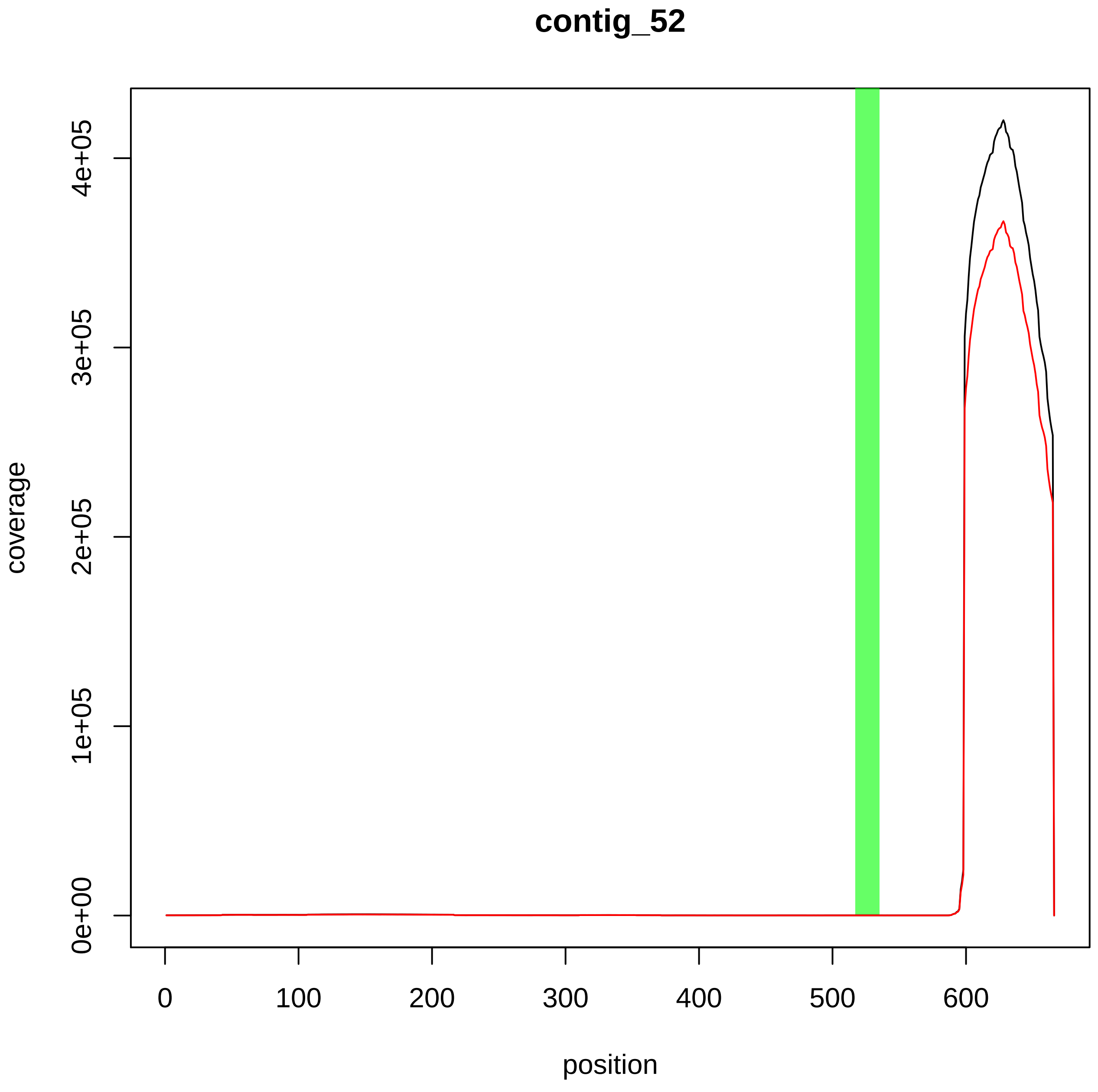

contig_52
coverage
0e+00
1e+05
2e+05
3e+05
4e+05
position
0
100
200
300
400
500
600

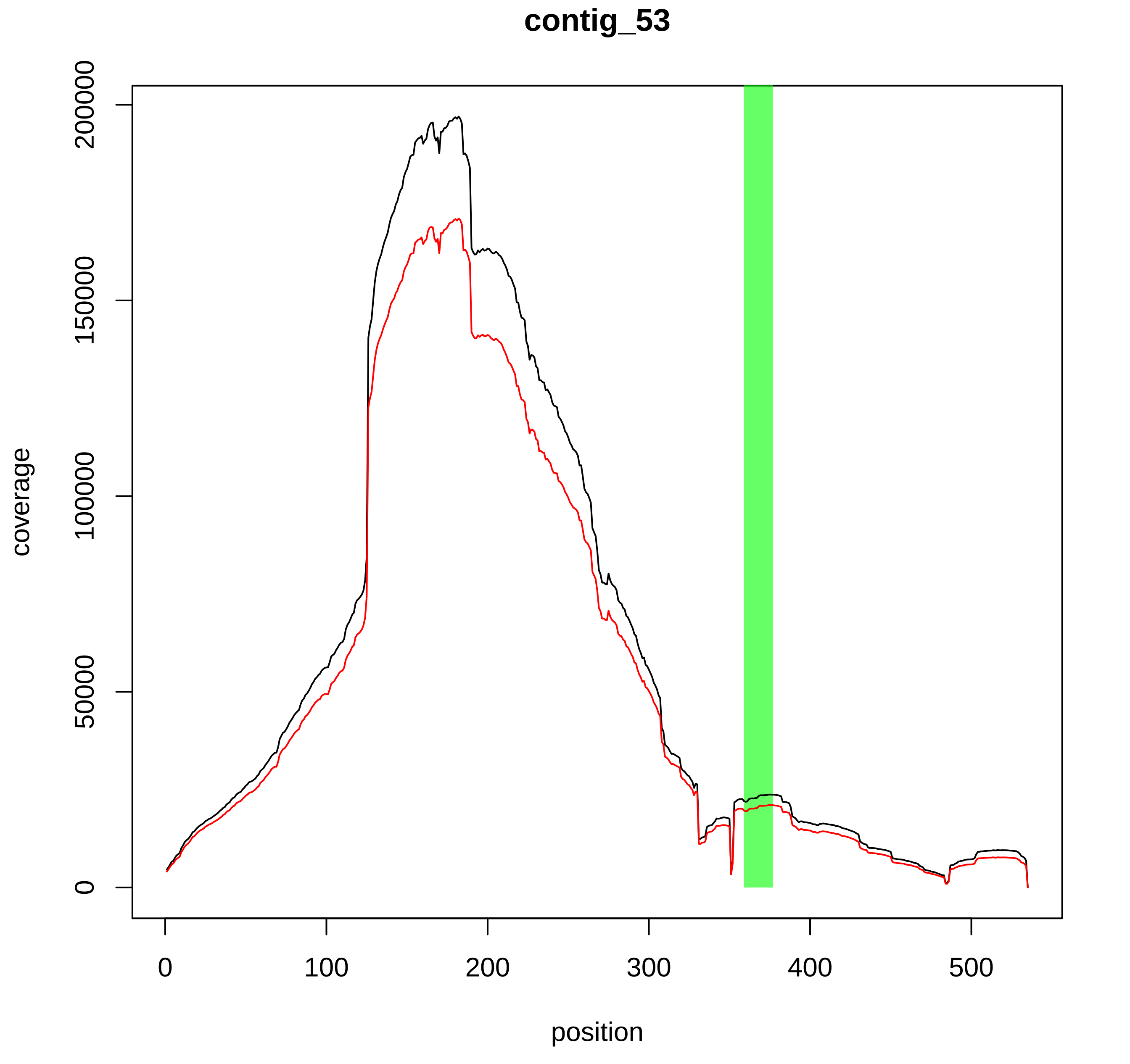
contig_53
coverage
0
50000
100000
150000
200000
position
0
100
200
300
400
500

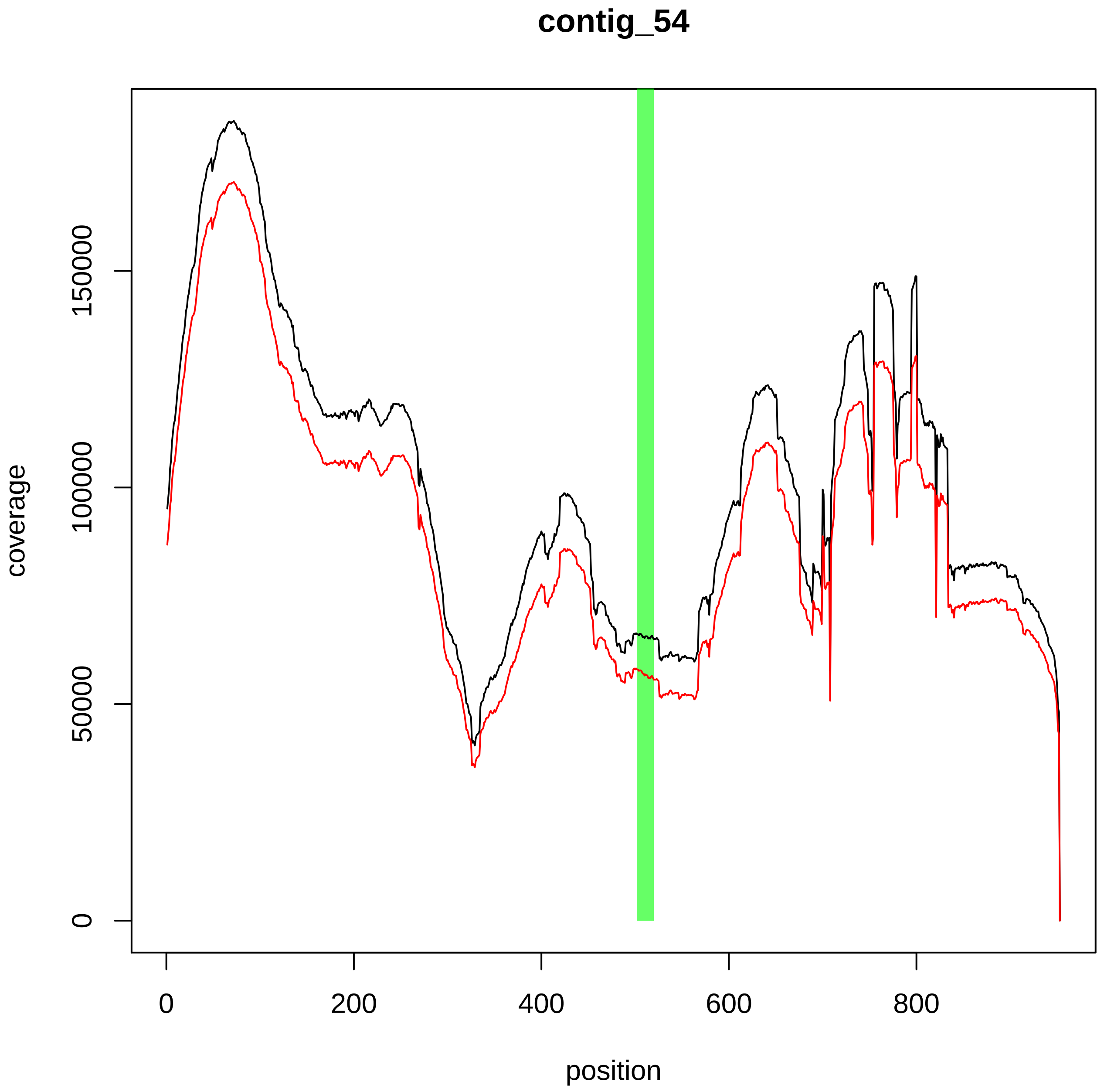
contig_54
coverage
position
0
50000
100000
150000
0
200
400
600
800

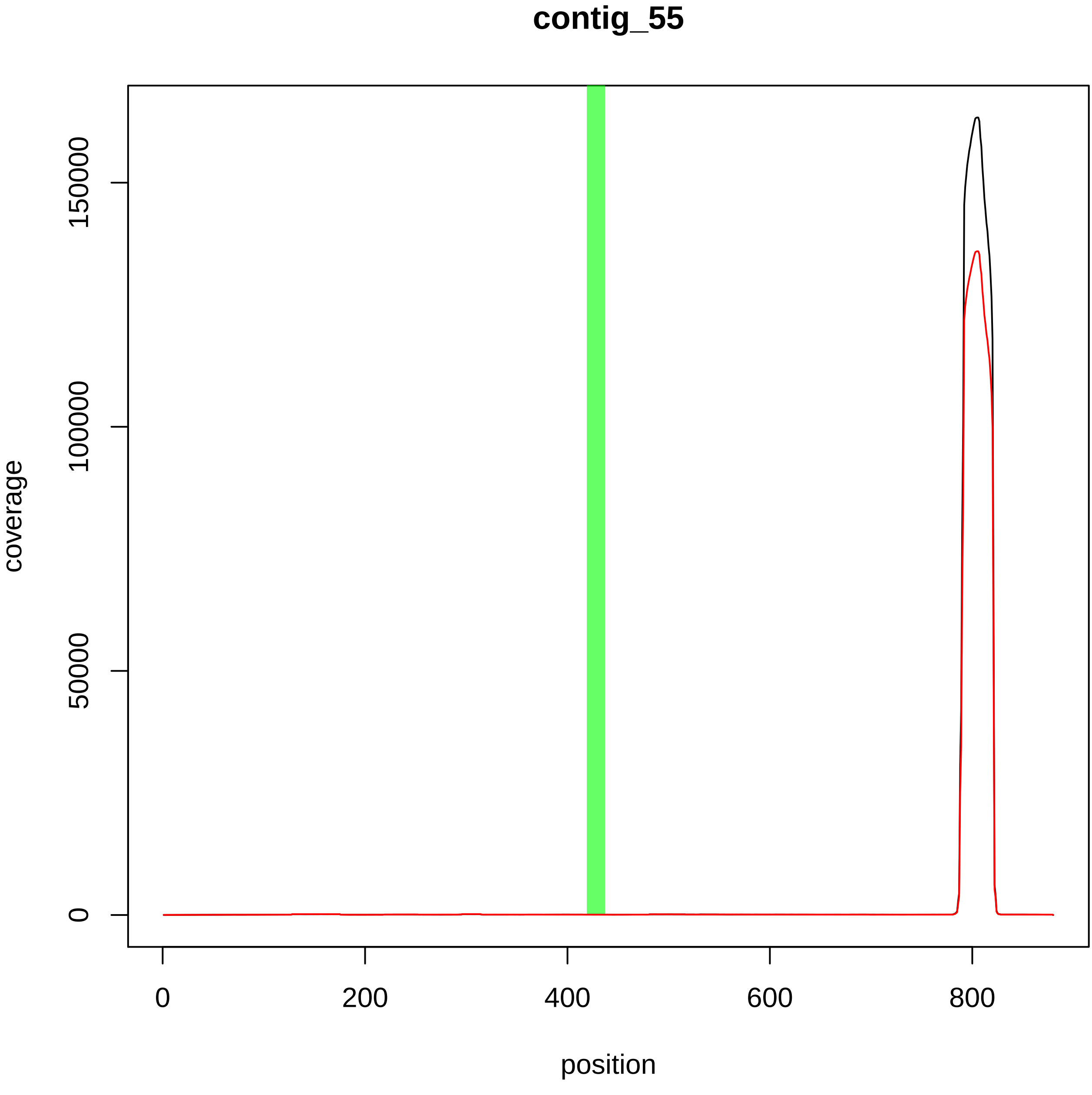

contig_55
coverage
0
50000
100000
150000
position
0
200
400
600
800

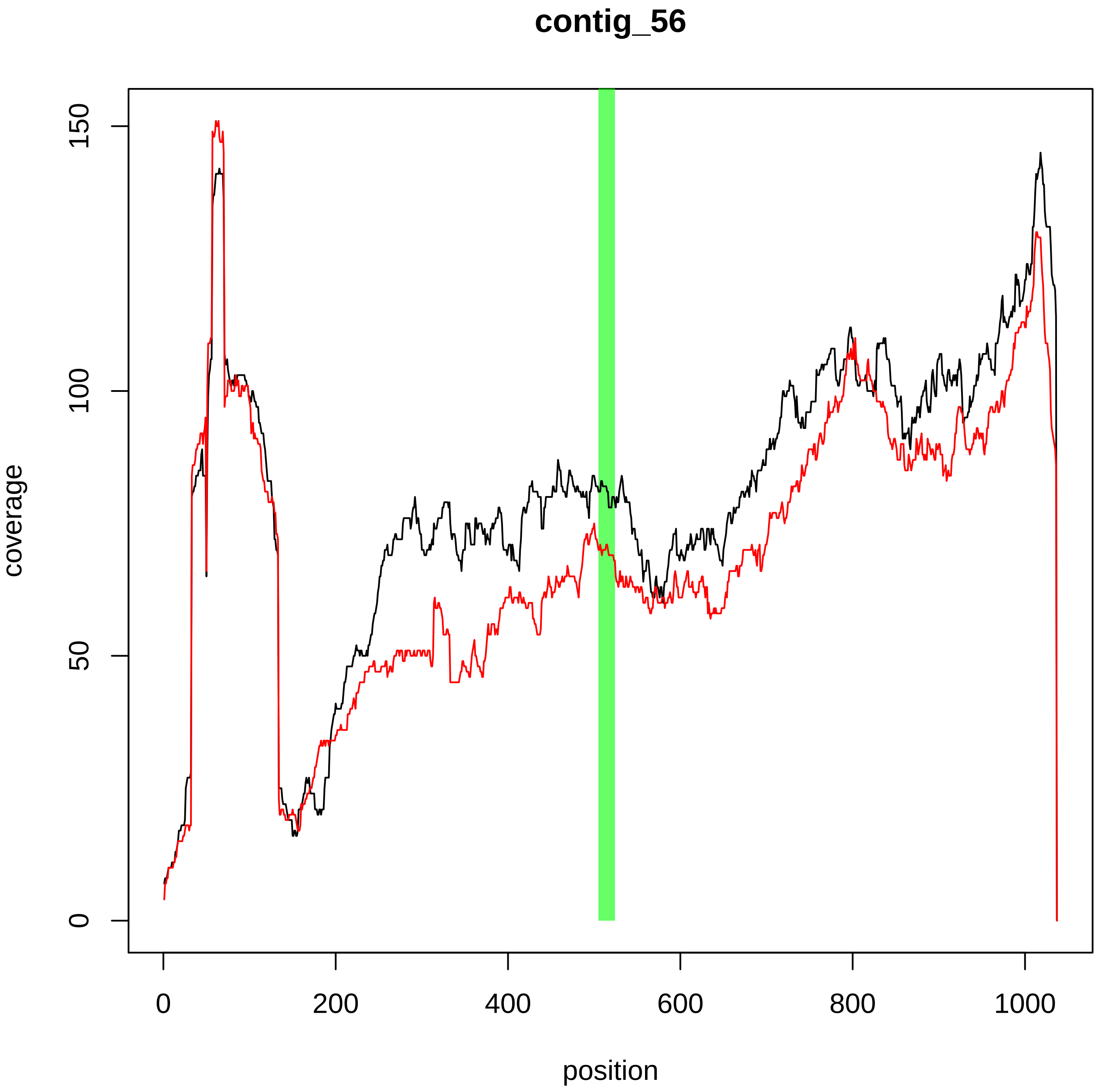
contig_56
coverage
position
0
50
100
150
0
200
400
600
800
1000

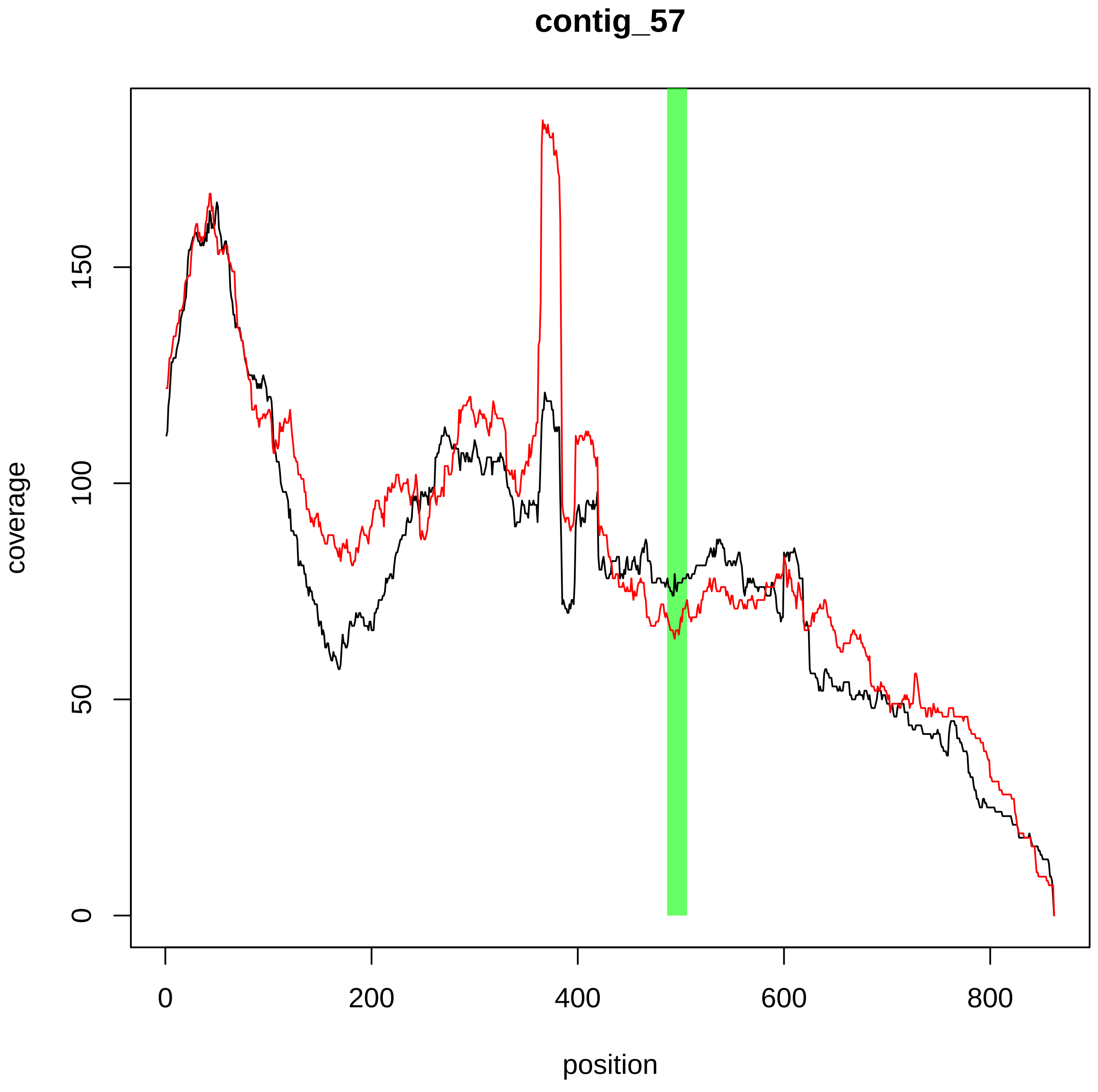
contig_57
coverage
0
50
100
150
position
0
200
400
600
800

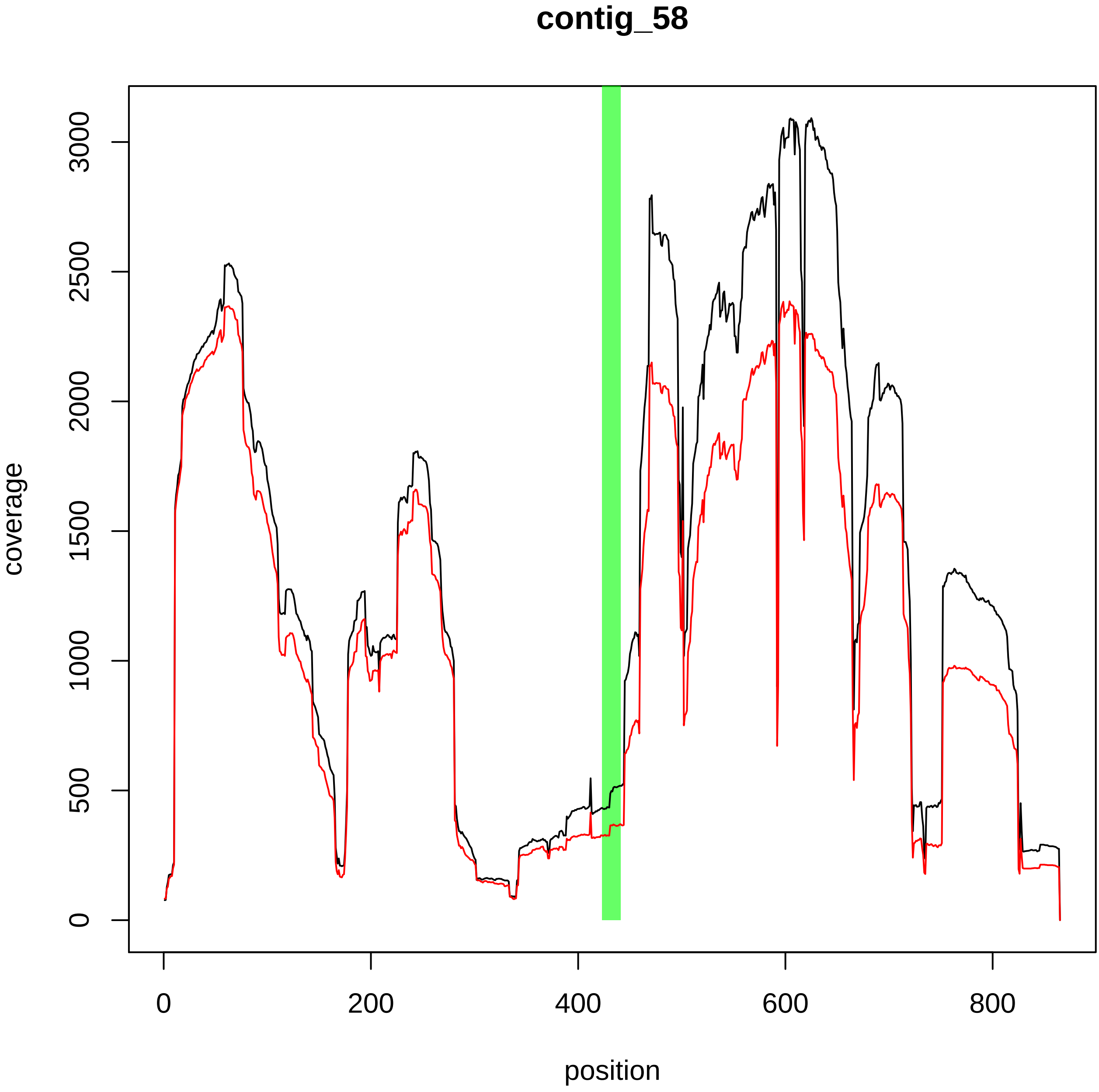
contig_58
coverage
position
0
500
1000
1500
2000
2500
3000
0
200
400
600
800

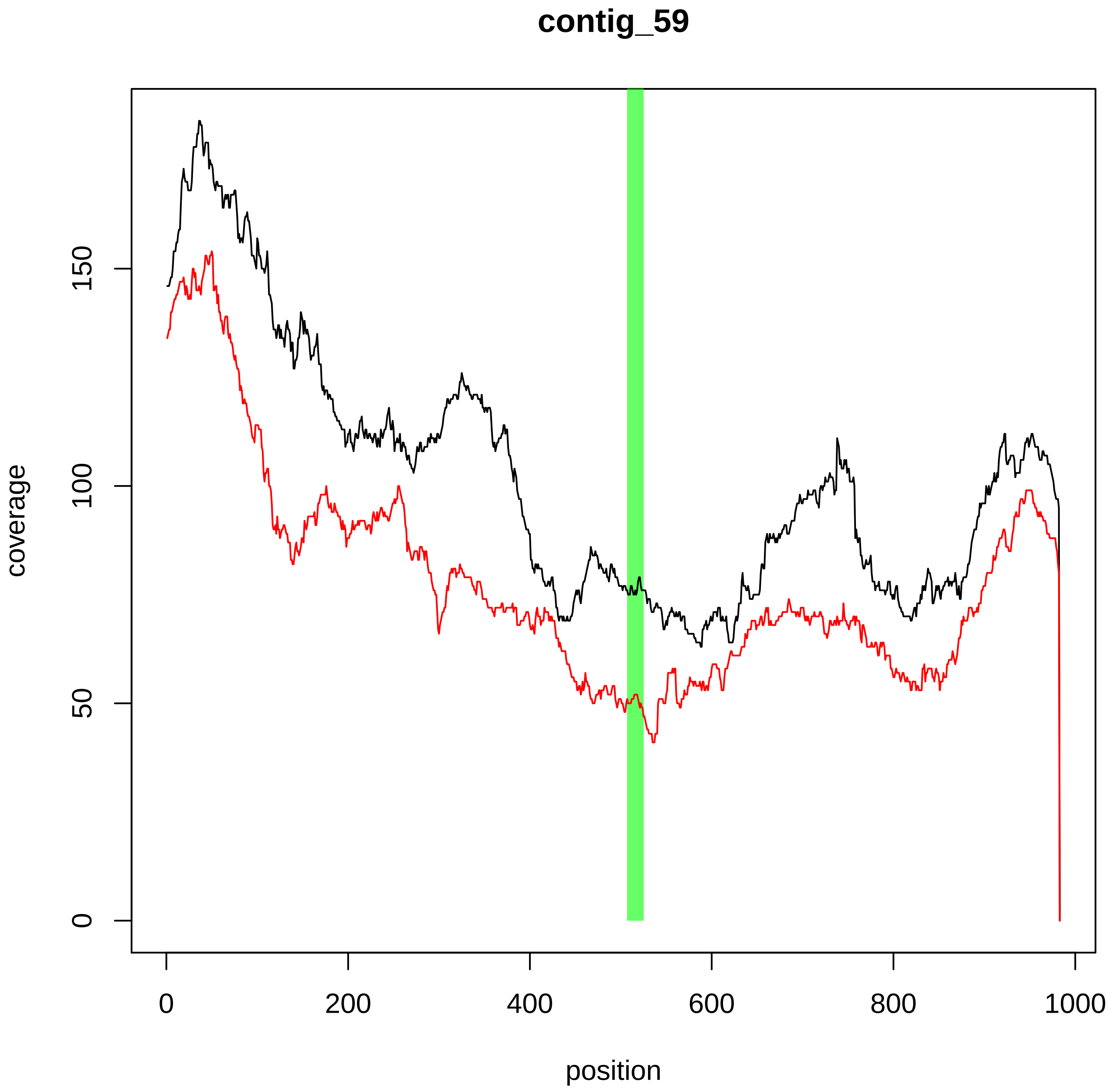

contig_59
coverage
position
0
50
100
150
0
200
400
600
800
1000

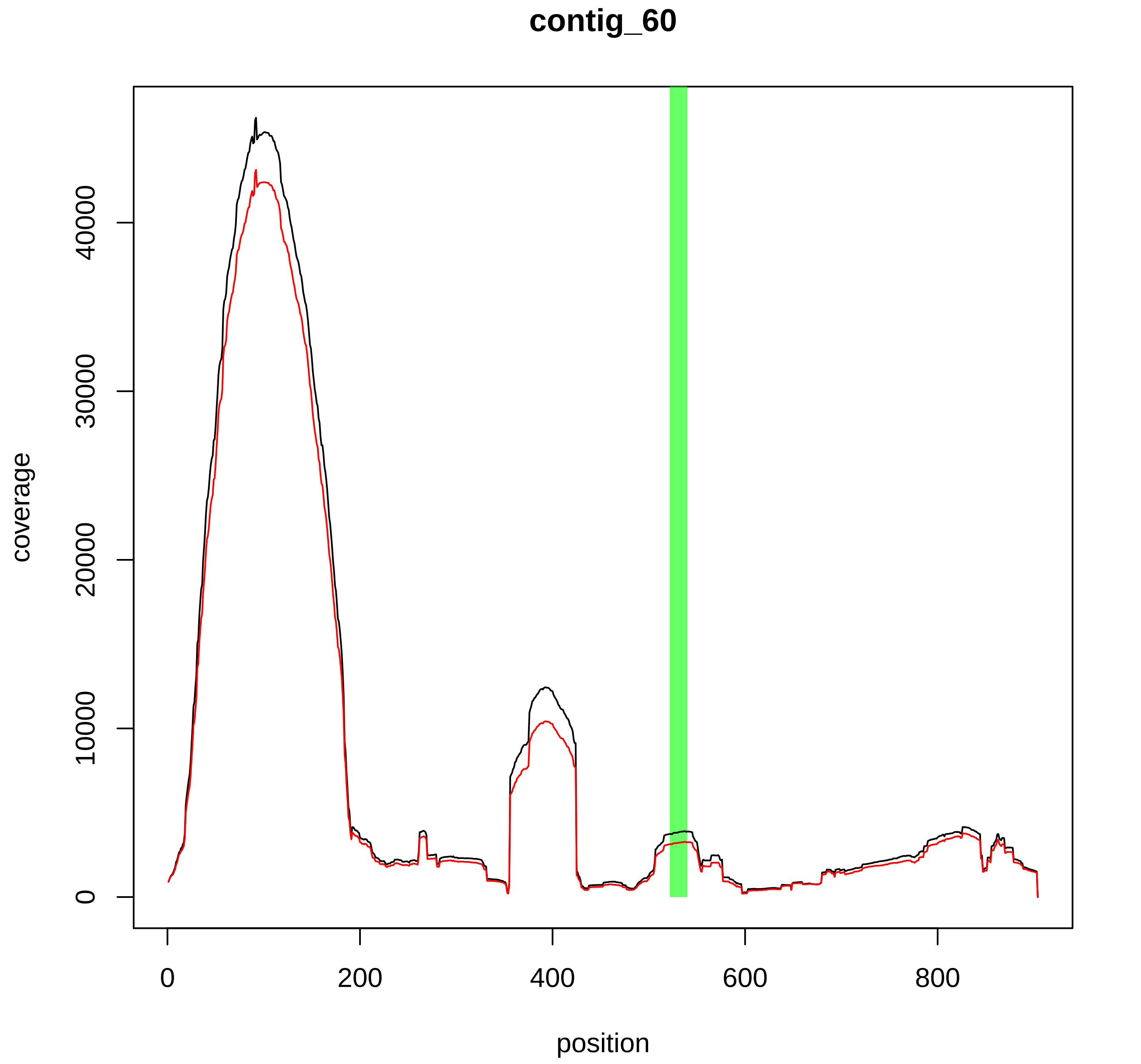
contig_60
coverage
0
10000
20000
30000
40000
position
0
200
400
600
800

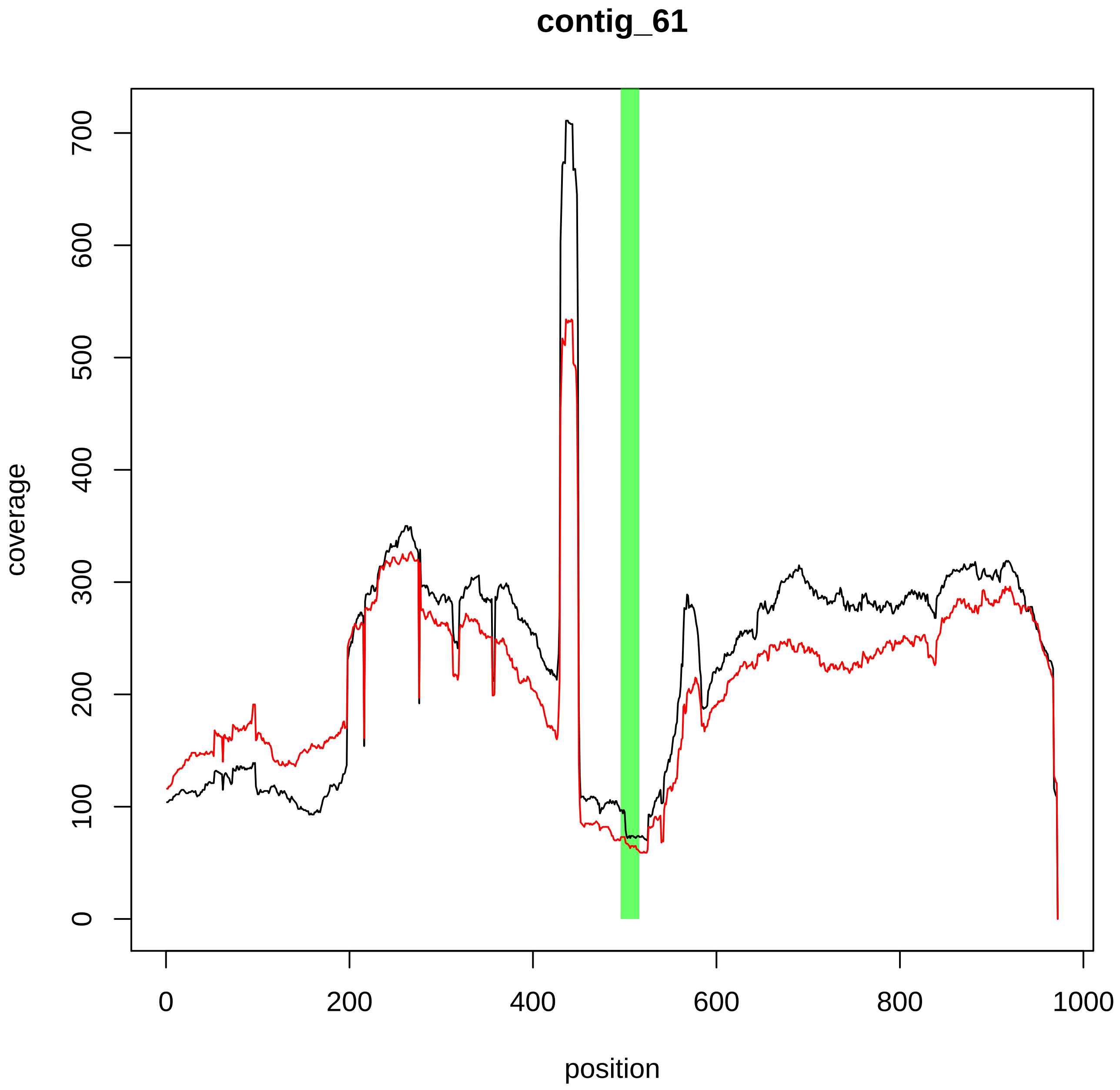
contig_61
coverage
position
0
100
200
300
400
500
600
700
0
200
400
600
800
1000

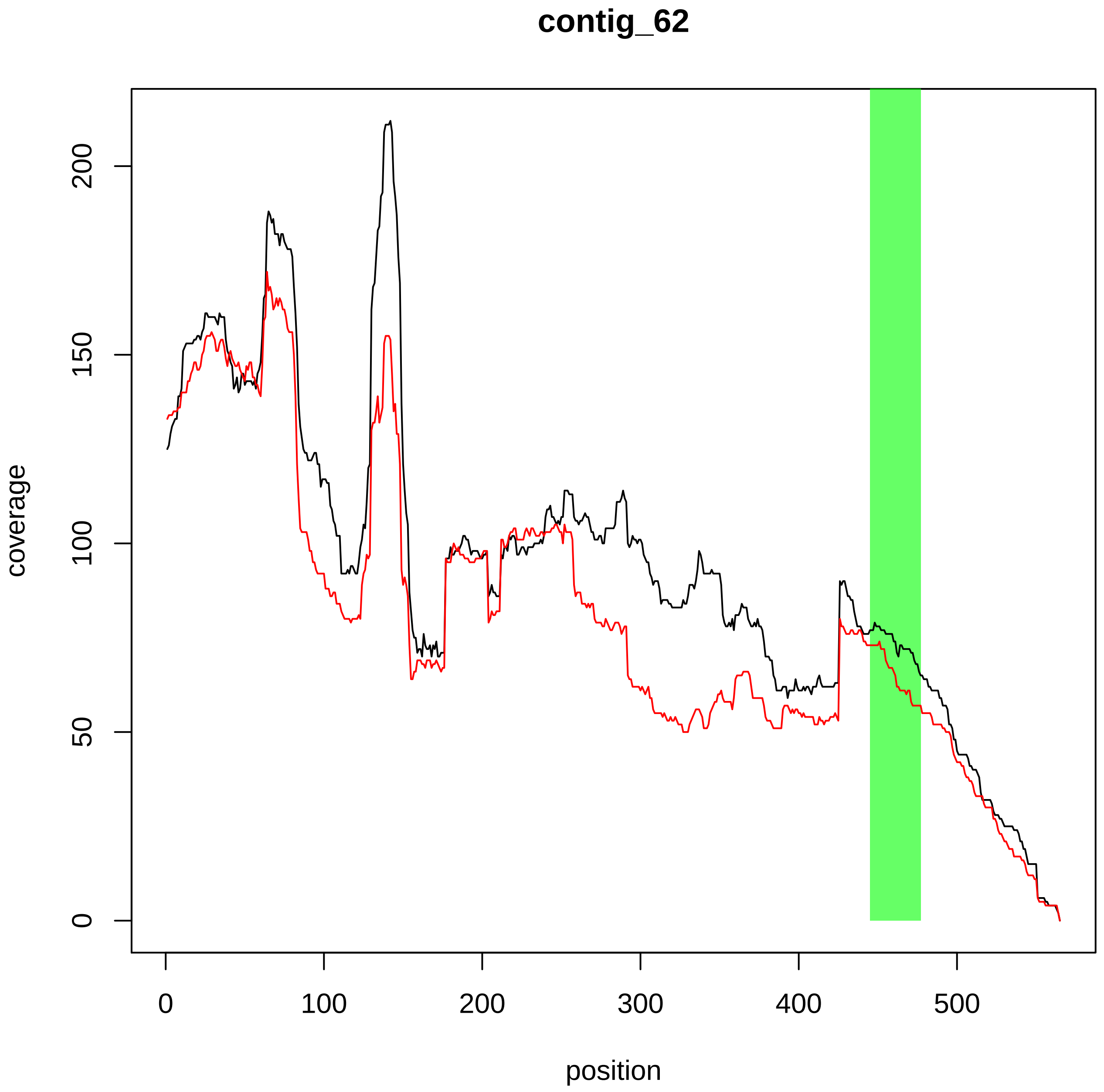

contig_62
coverage
position
0
50
100
150
200
0
100
200
300
400
500

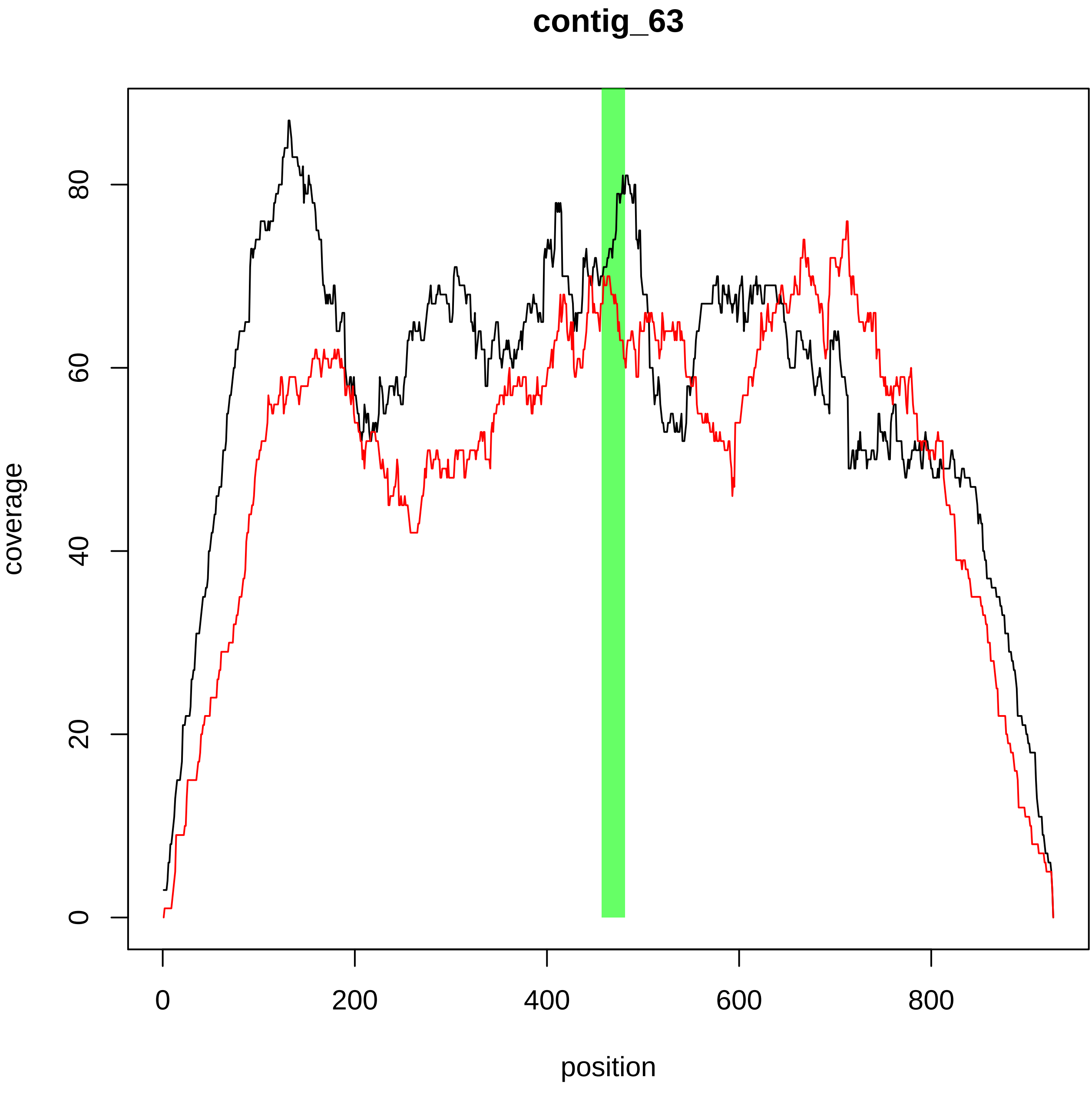
contig_63
coverage
position
0
20
40
60
80
0
200
400
600
800

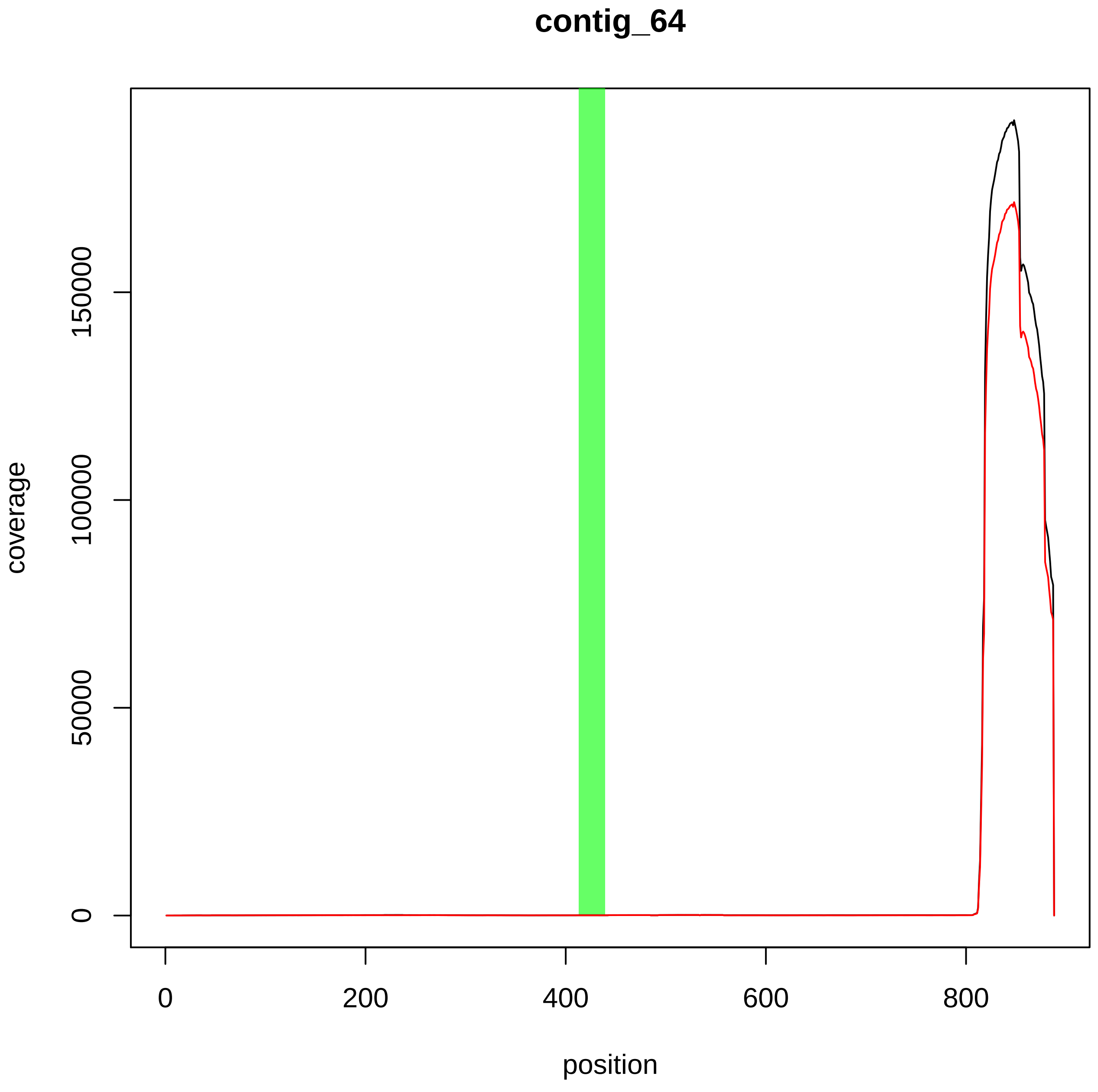

contig_64
coverage
0
50000
100000
150000
0
200
400
600
800
position

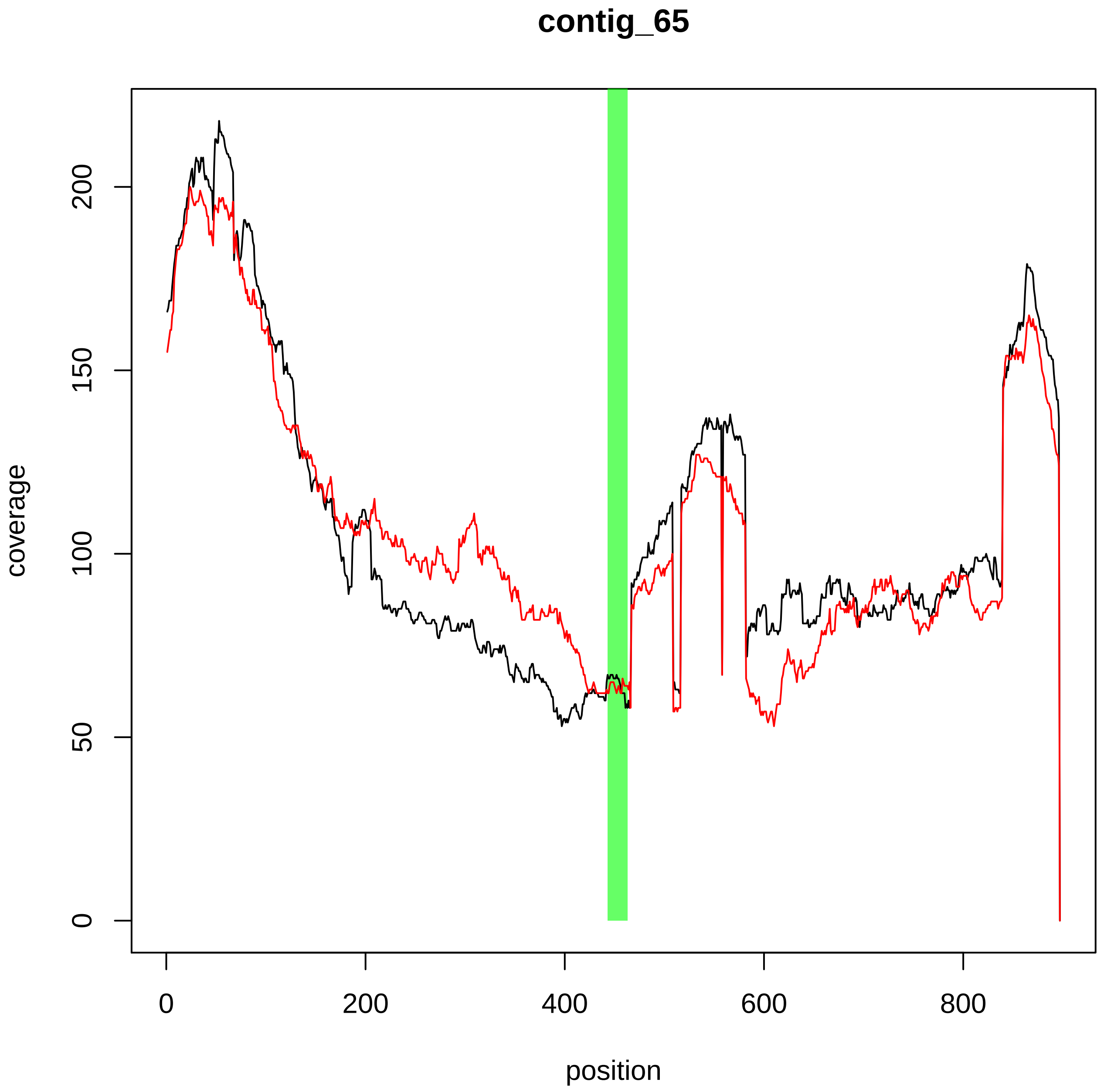
contig_65
coverage
0
50
100
150
200
position
0
200
400
600
800

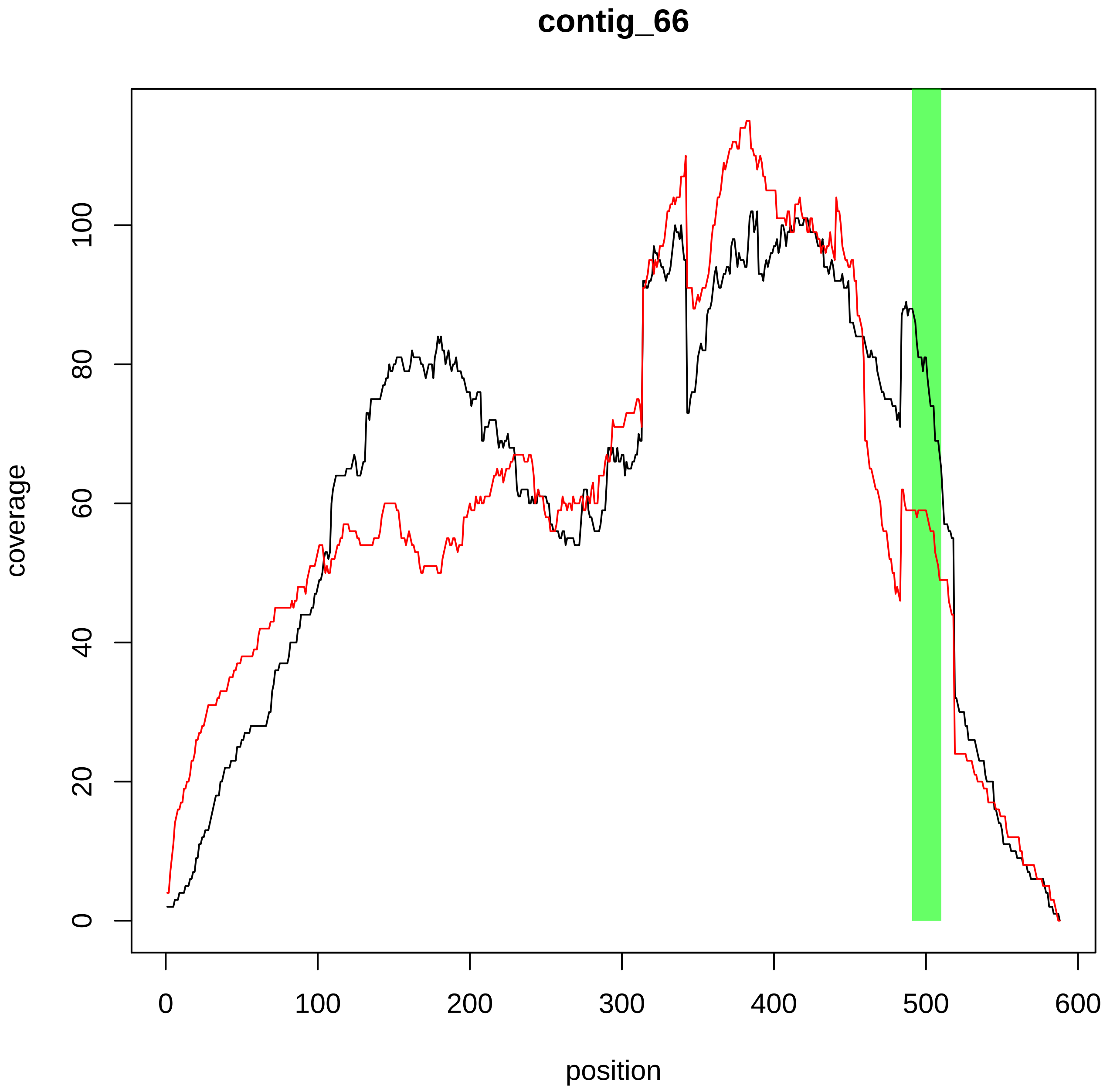
contig_66
coverage
0
20
40
60
80
100
position
0
100
200
300
400
500
600

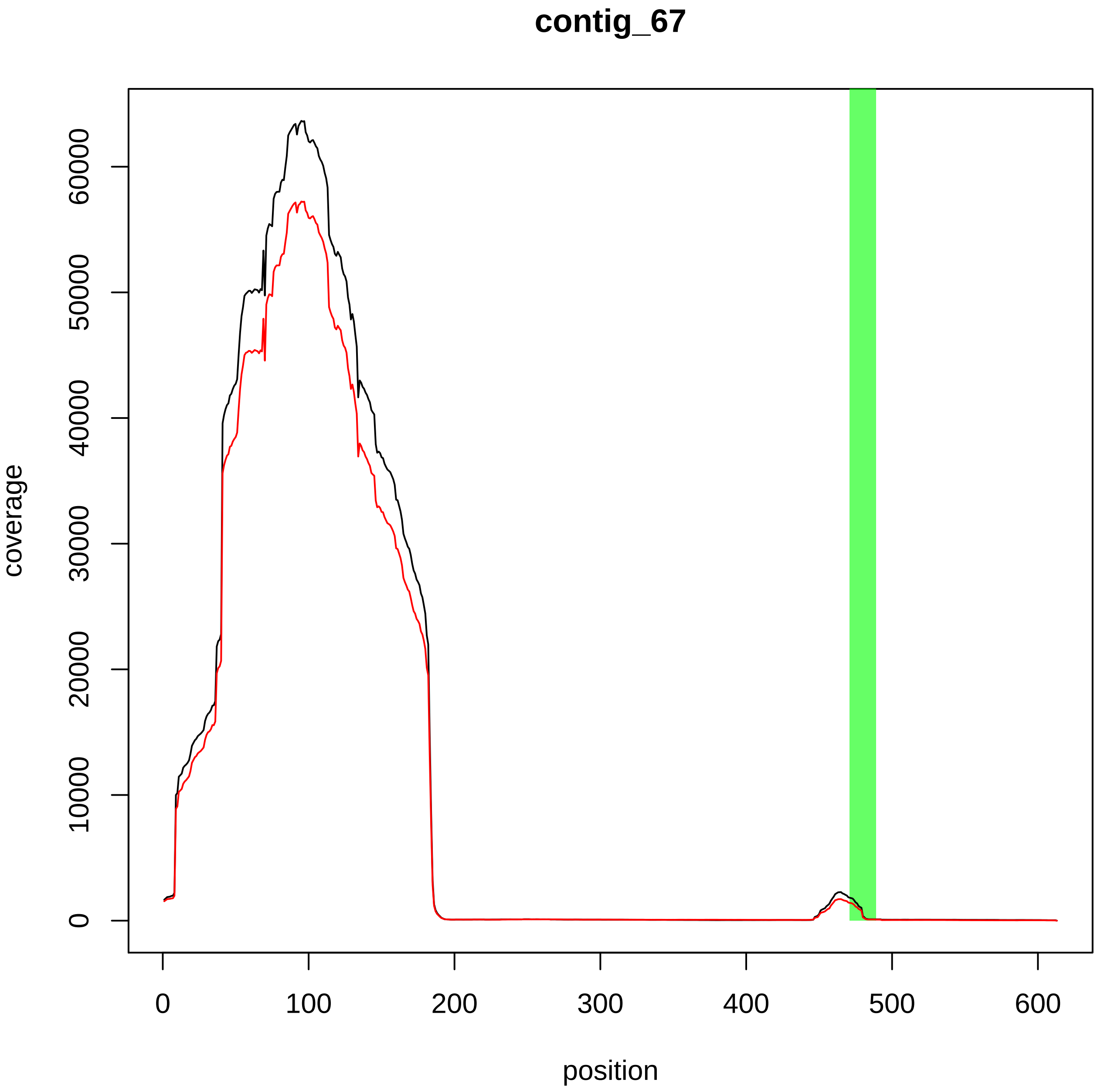

contig_67
coverage
0
10000
20000
30000
40000
50000
60000
position
0
100
200
300
400
500
600

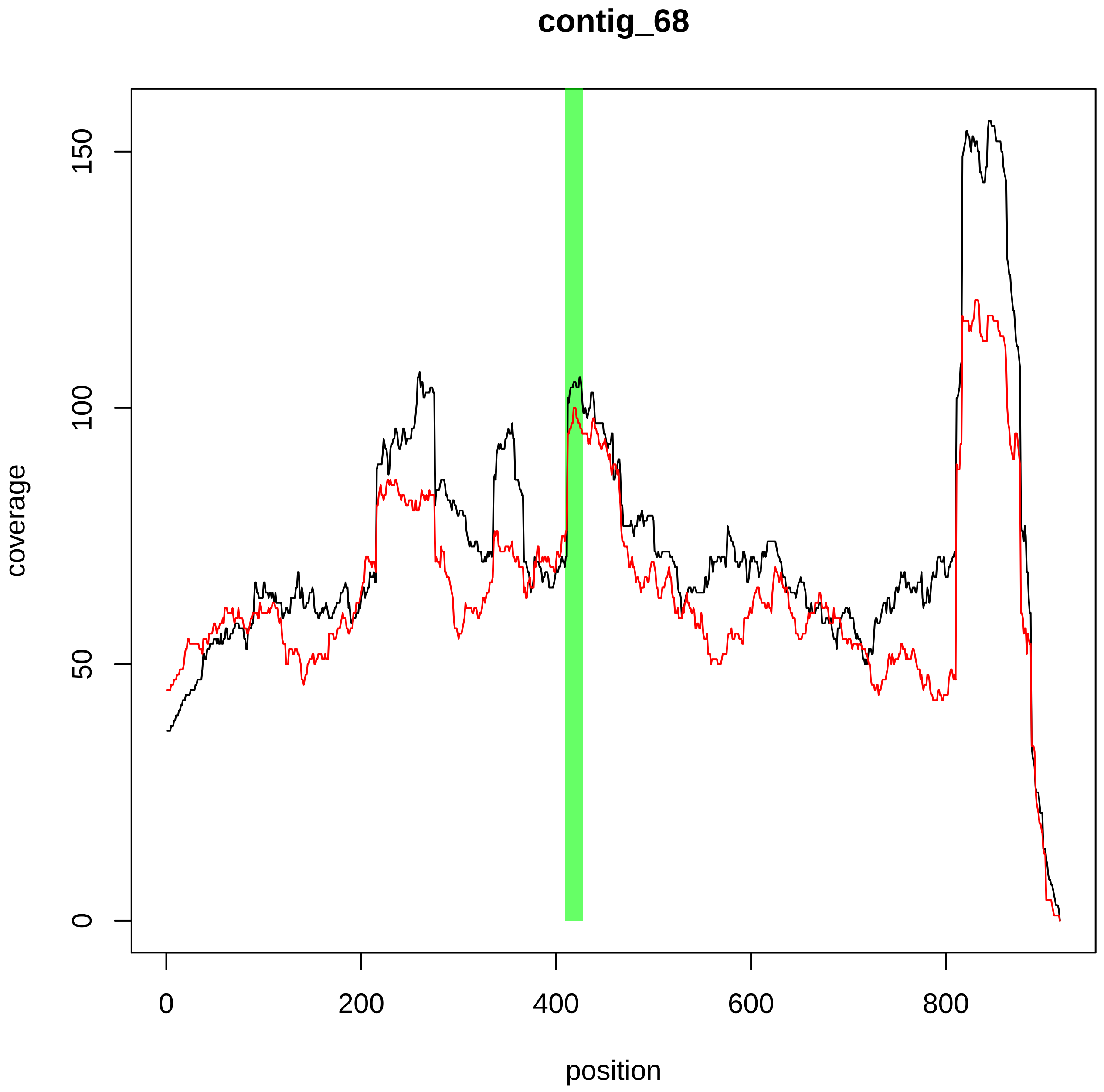
contig_68
coverage
position
0
50
100
150
0
200
400
600
800

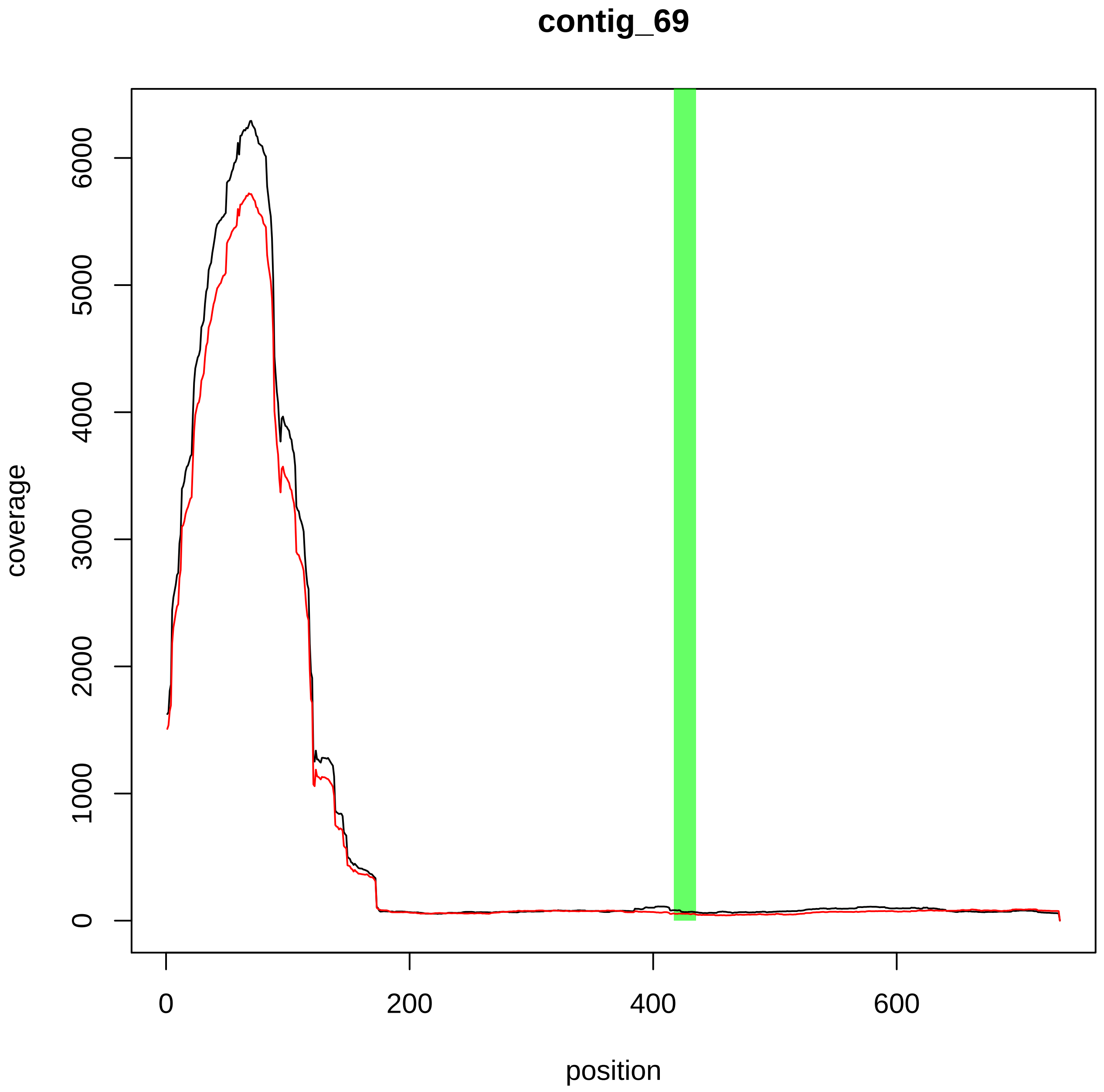
contig_69
coverage
position
0
1000
2000
3000
4000
5000
6000
0
200
400
600

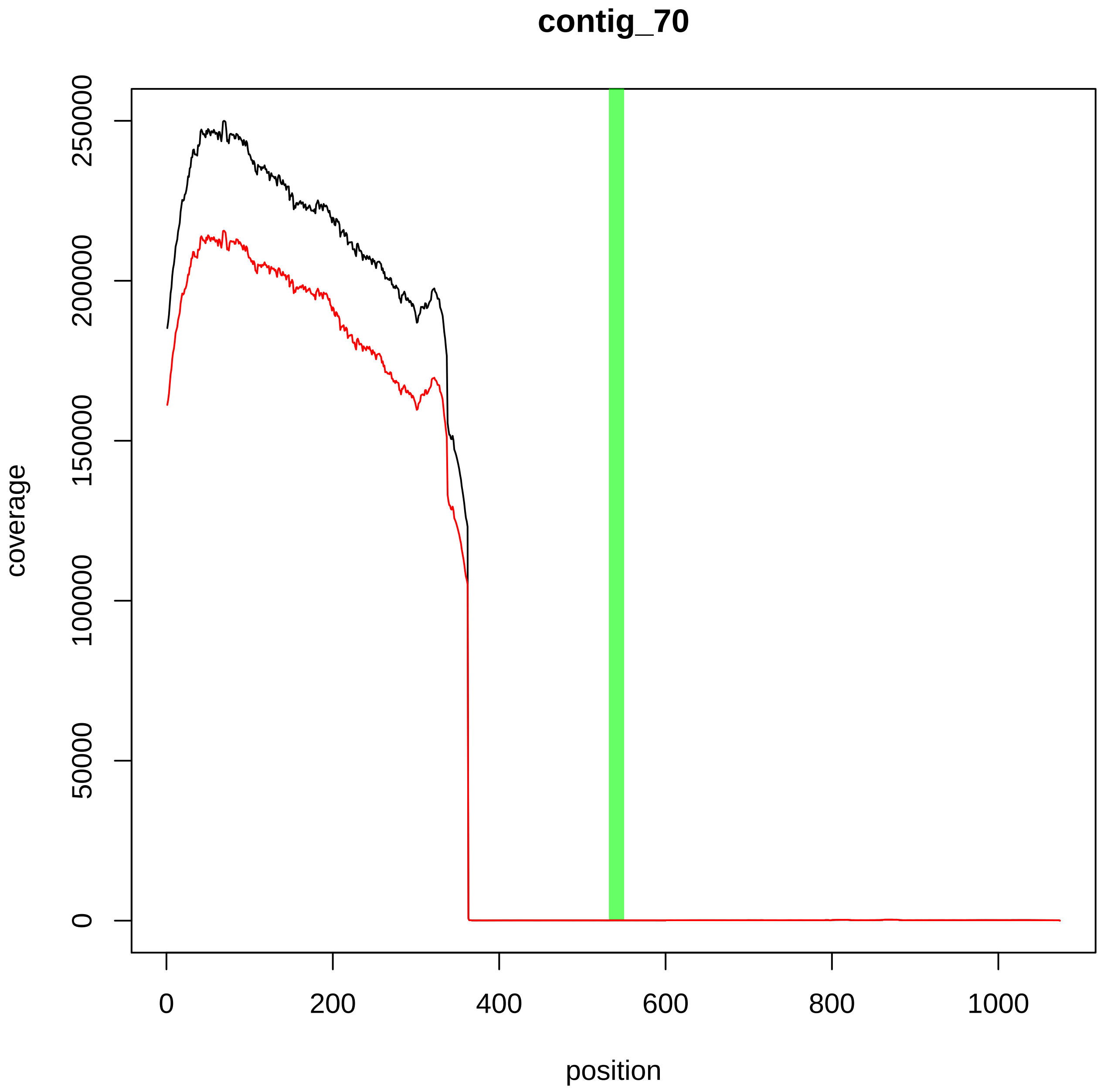

contig_70
coverage
0
50000
100000
150000
200000
250000
position
0
200
400
600
800
1000

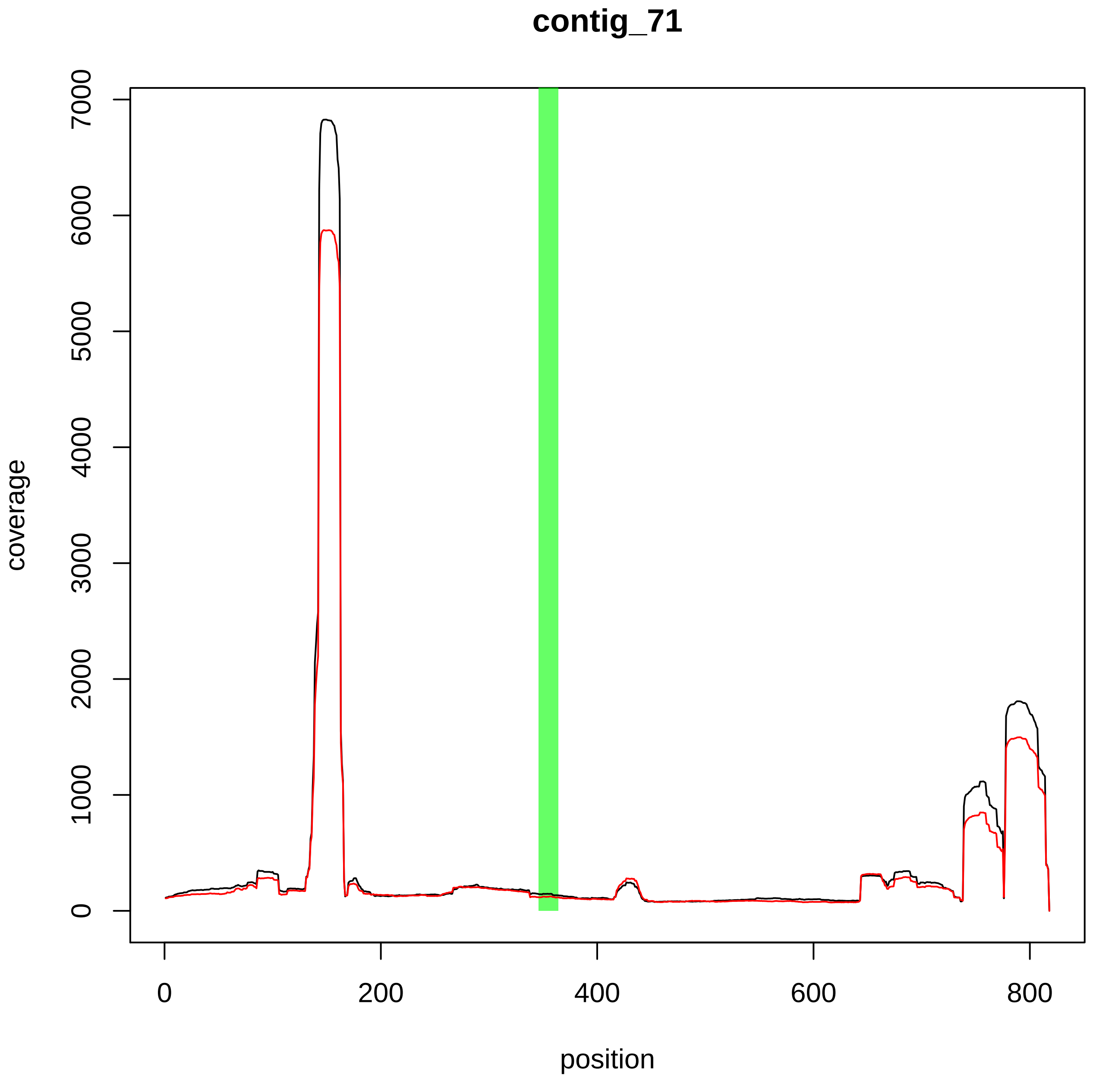
contig_71
coverage
0
1000
2000
3000
4000
5000
6000
7000
position
0
200
400
600
800

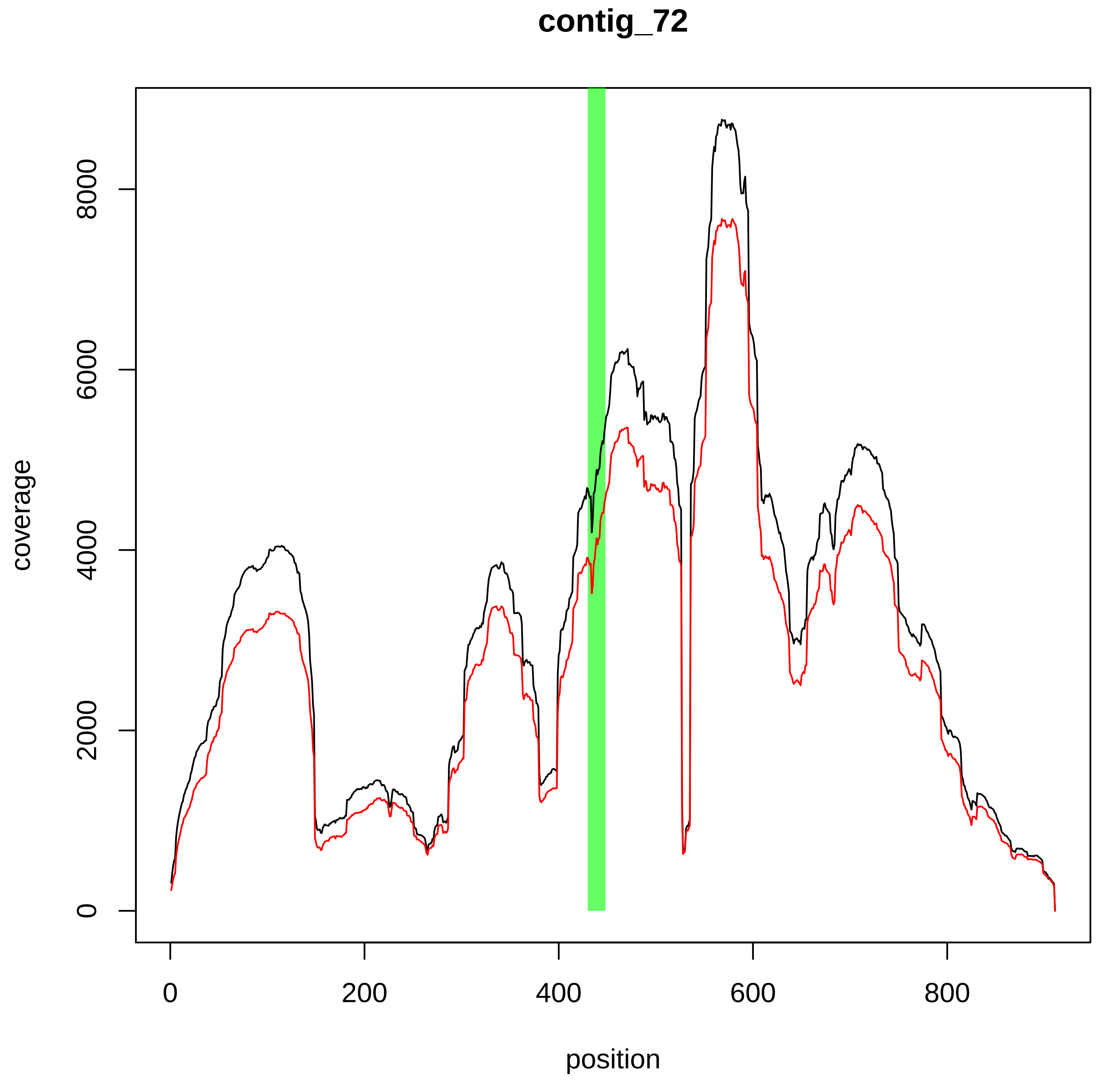

contig_72
coverage
position
0
2000
4000
6000
8000
0
200
400
600
800

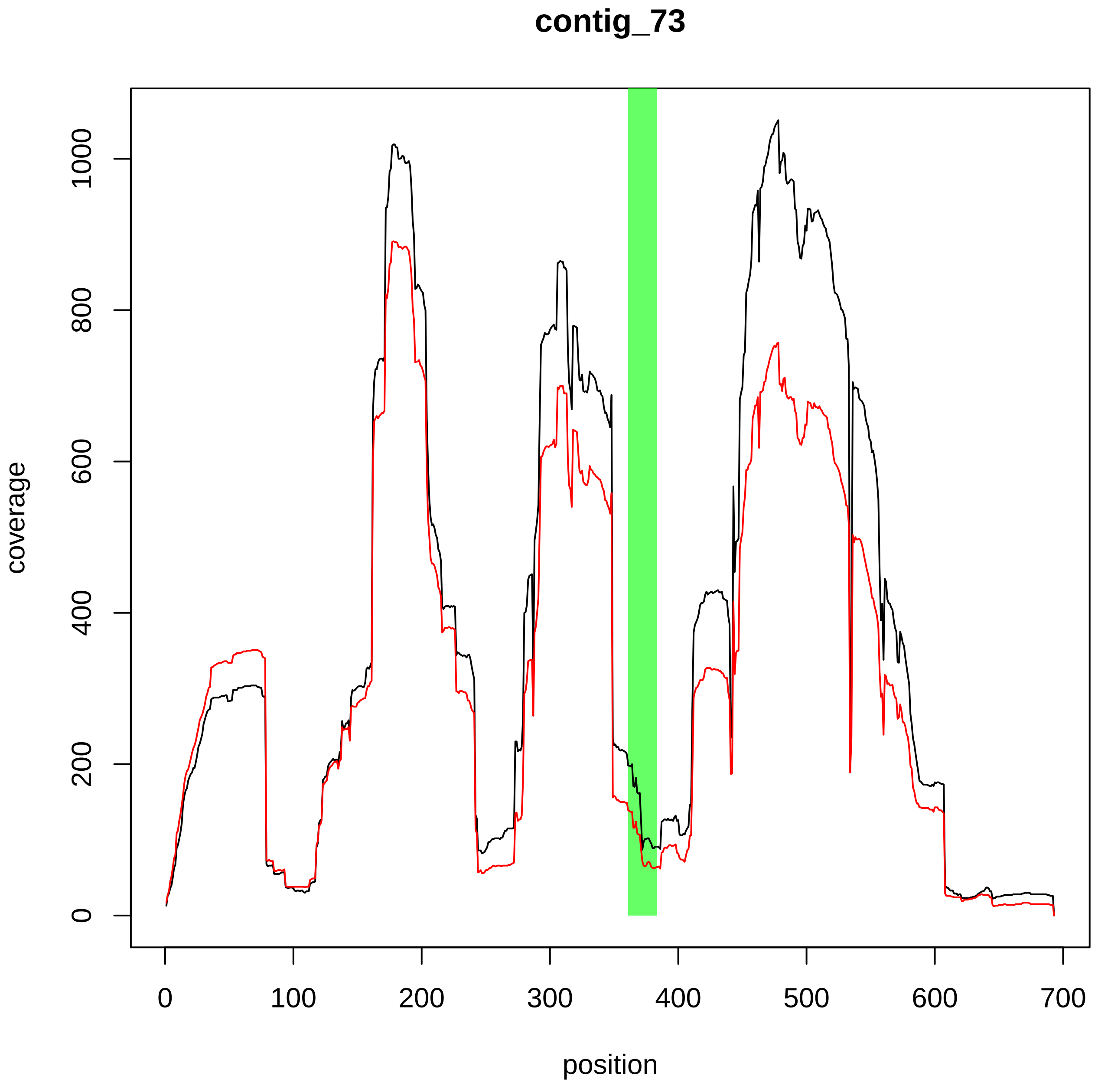
contig_73
coverage
position
0
200
400
600
800
1000
0
100
200
300
400
500
600
700

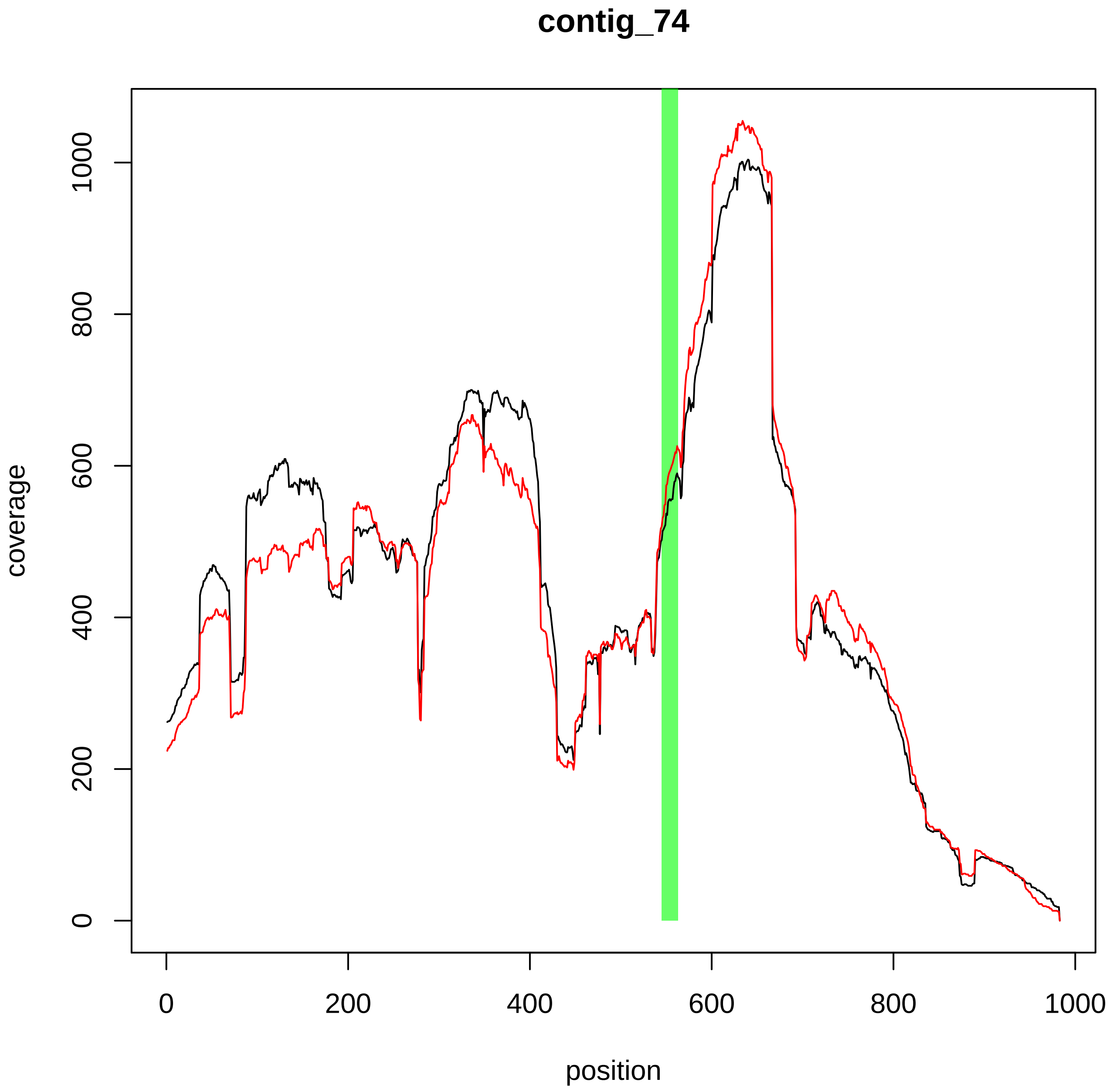

contig_74
coverage
position
0
200
400
600
800
1000
0
200
400
600
800
1000

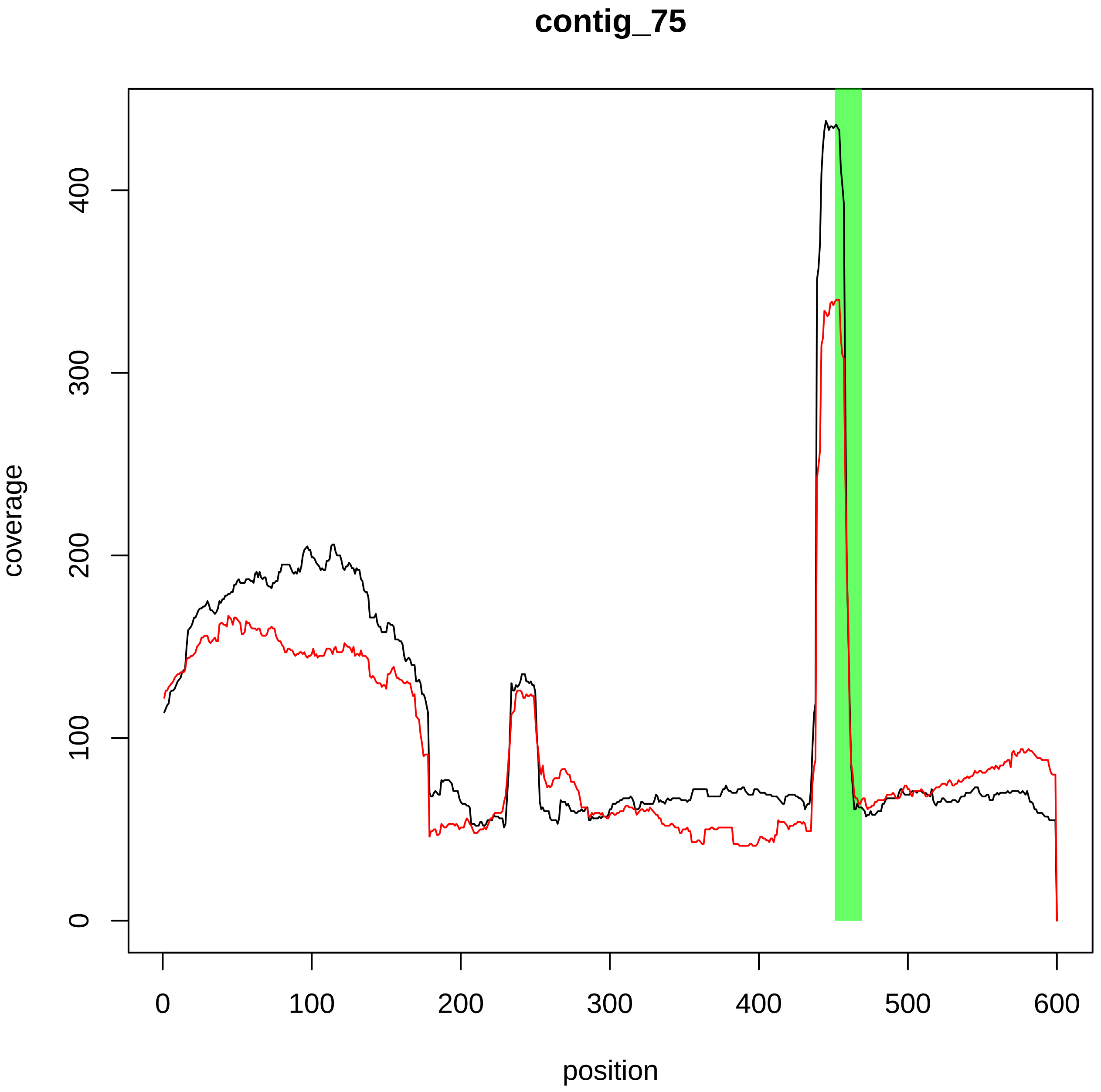

contig_75
coverage
position
0
100
200
300
400
0
100
200
300
400
500
600

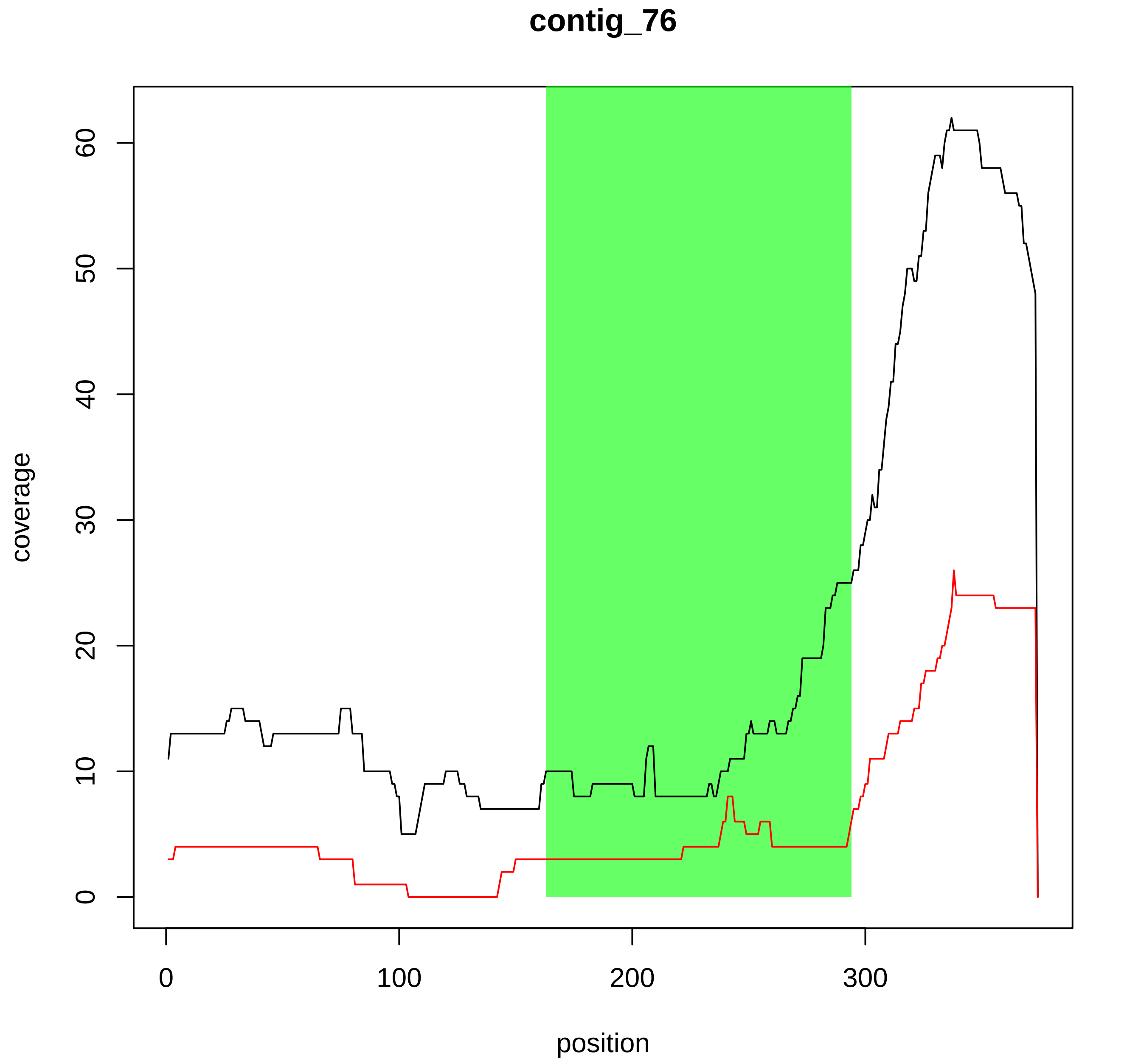

contig_76
coverage
position
0
10
20
30
40
50
60
0
100
200
300

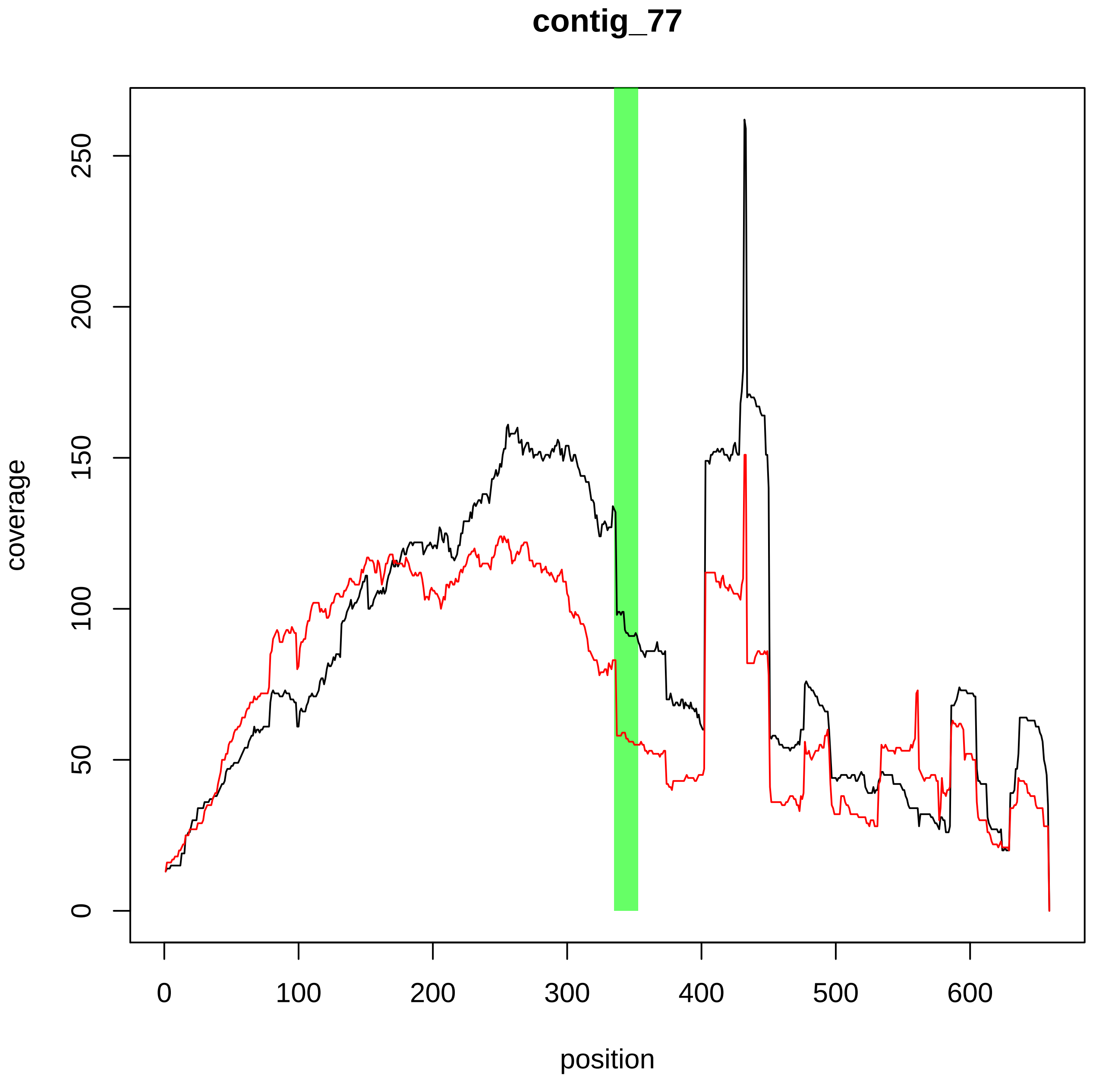

contig_77
coverage
0
50
100
150
200
250
position
0
100
200
300
400
500
600

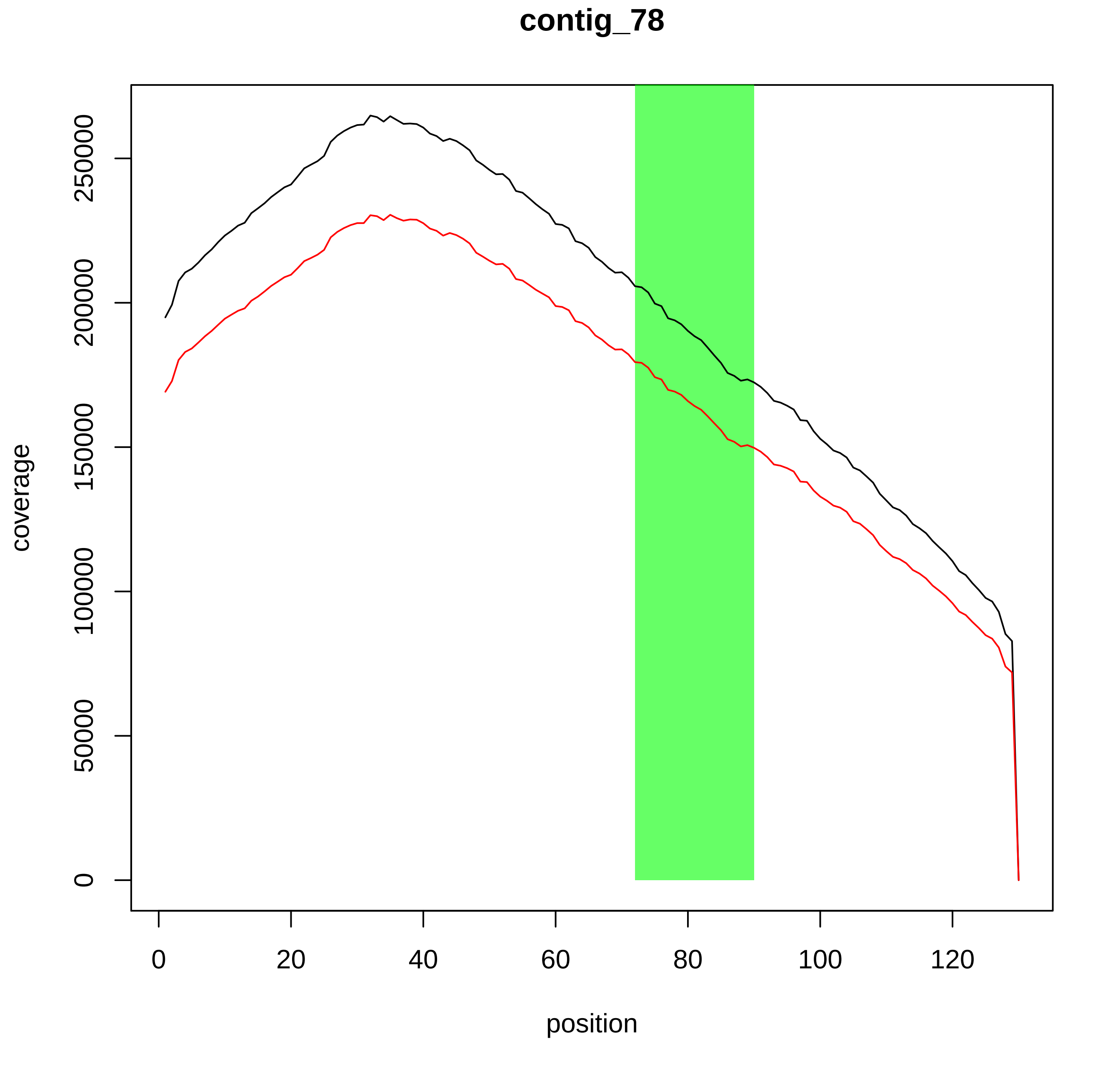
contig_78
coverage
0
50000
100000
150000
200000
250000
0
20
40
60
80
100
120
position

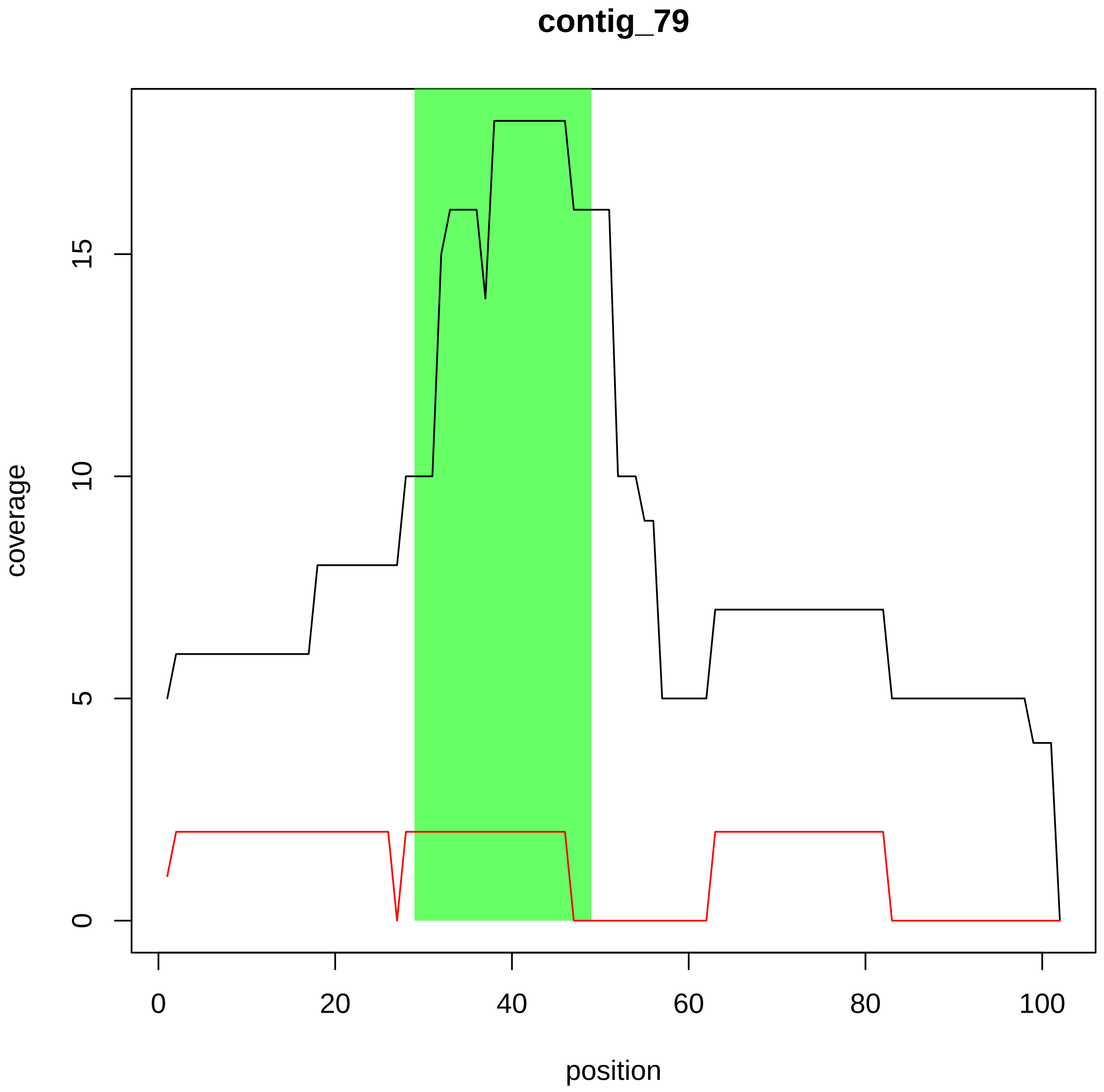
contig_79
coverage
0
5
10
15
position
0
20
40
60
80
100

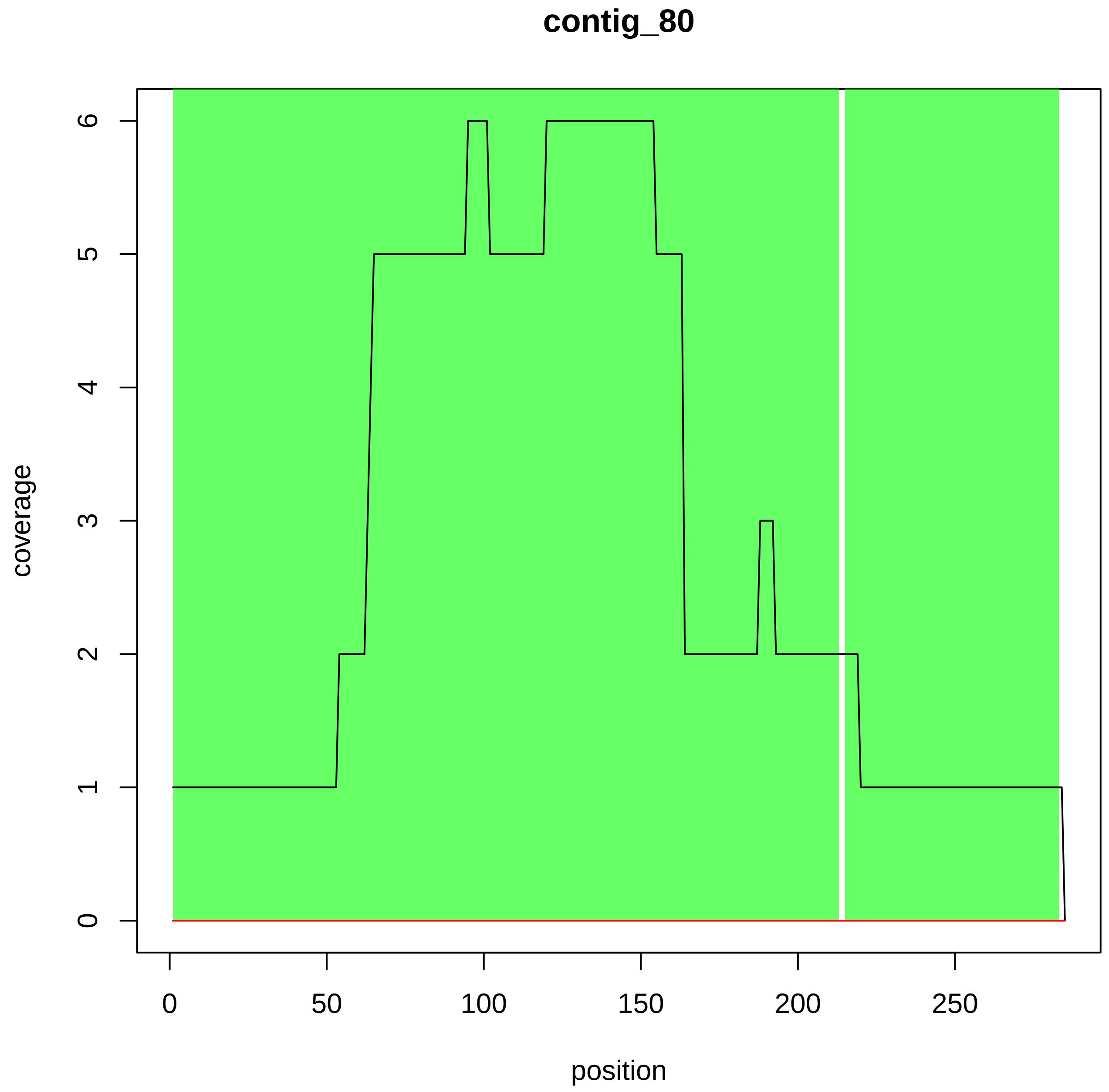
contig_80
coverage
0
1
2
3
4
5
6
0
50
100
150
200
250
position

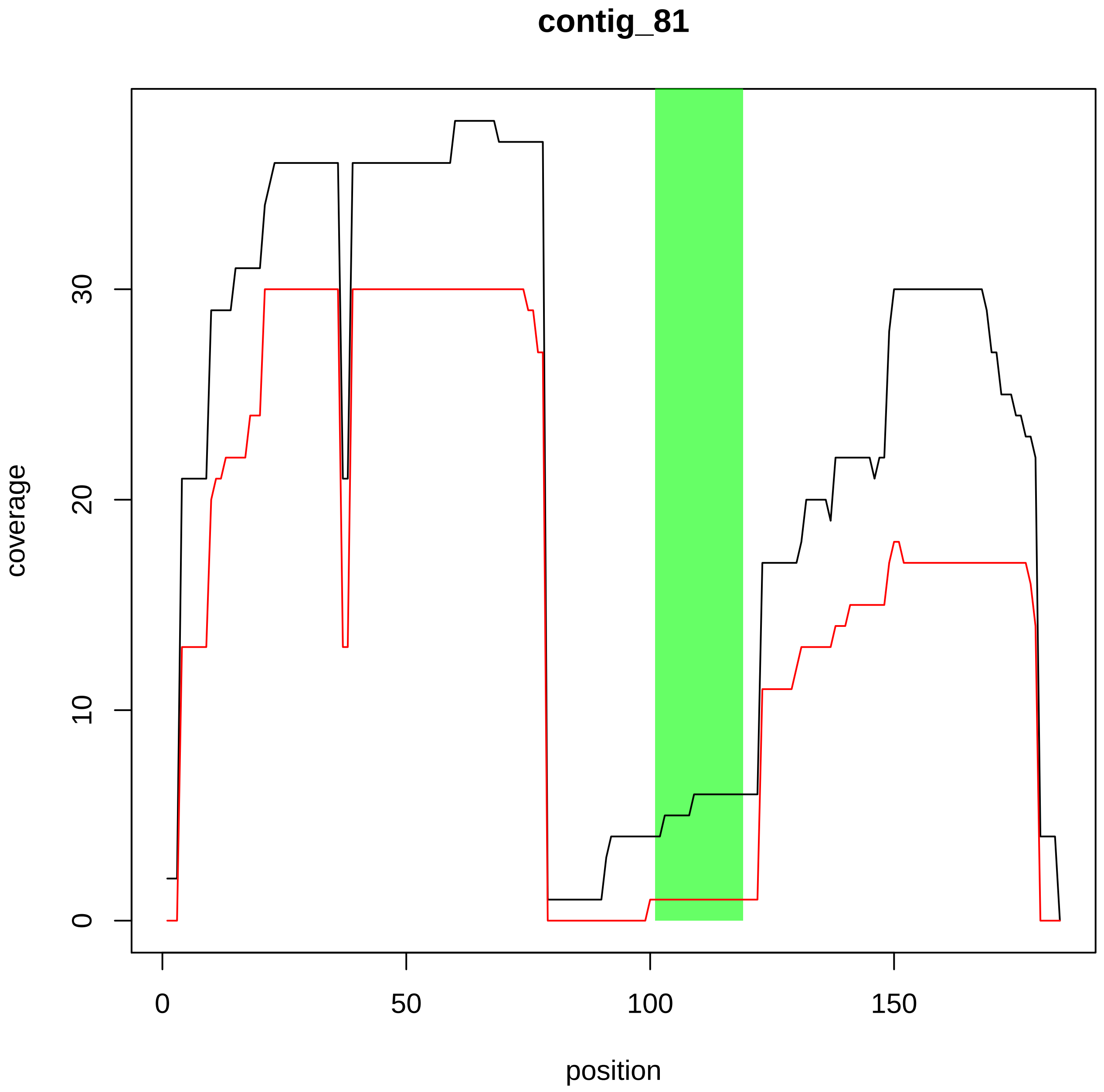
contig_81
coverage
0
10
20
30
position
0
50
100
150

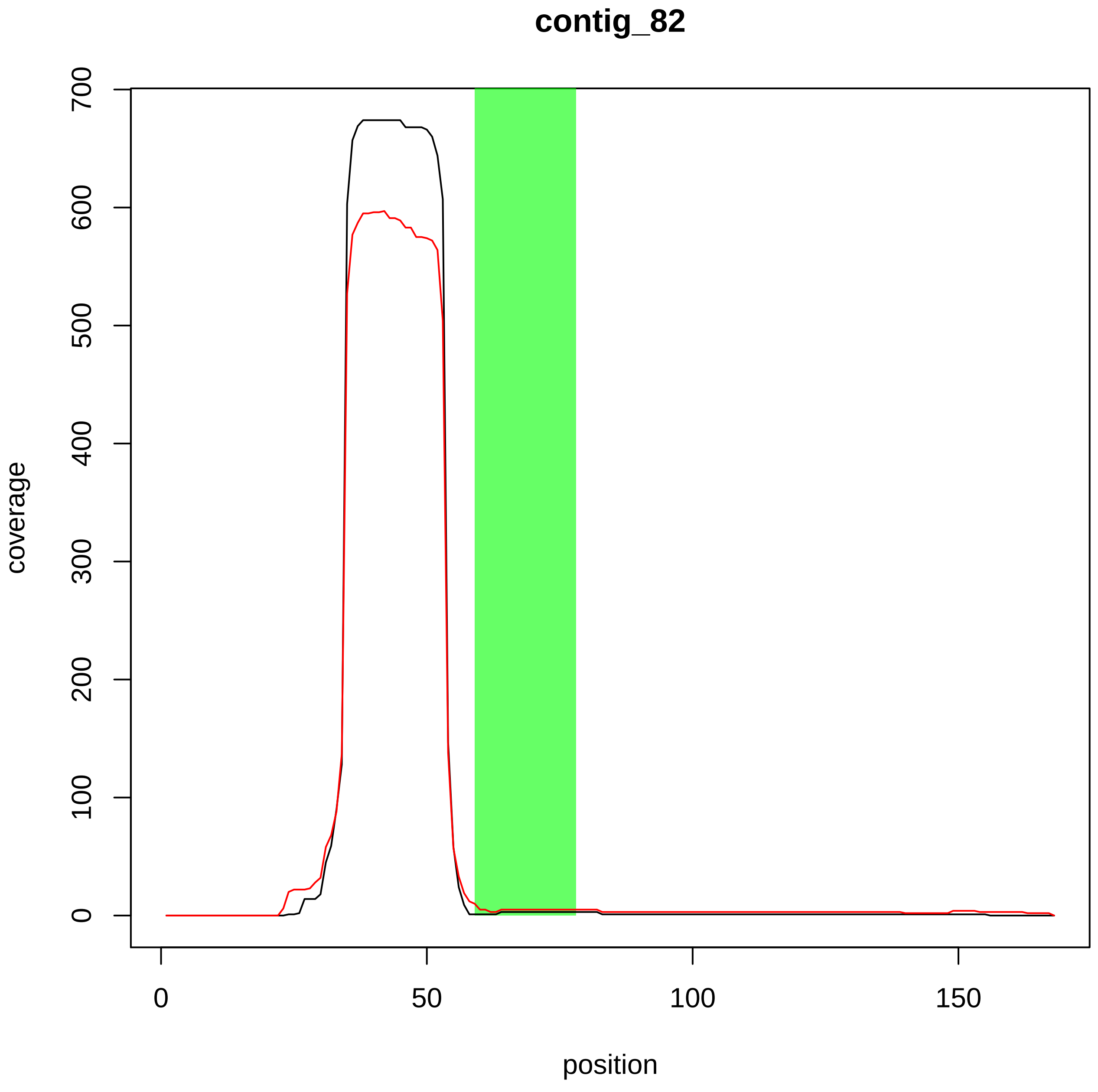
contig_82
coverage
0
100
200
300
400
500
600
700
position
0
50
100
150

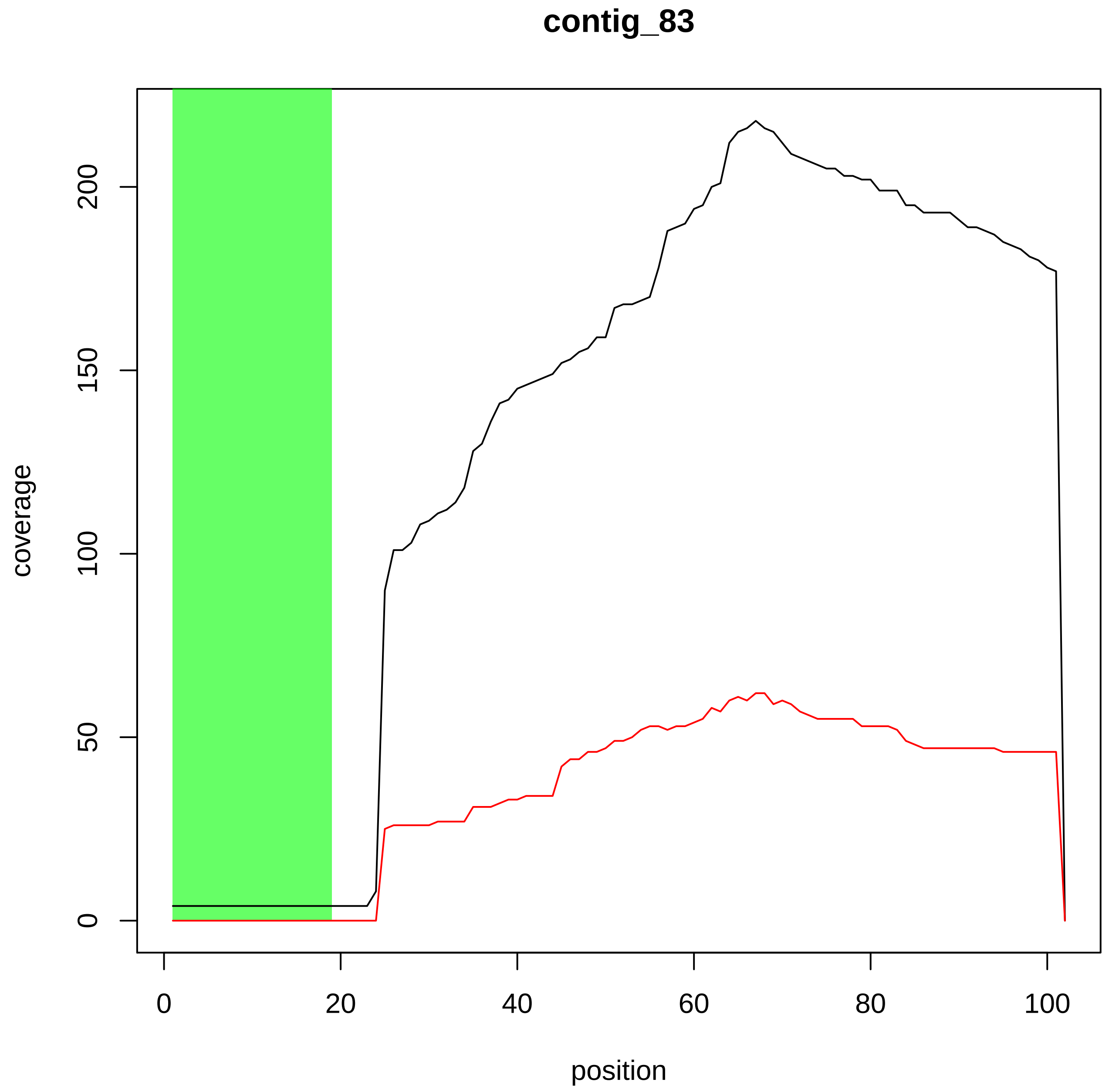

contig_83
coverage
position
0
50
100
150
200
0
20
40
60
80
100

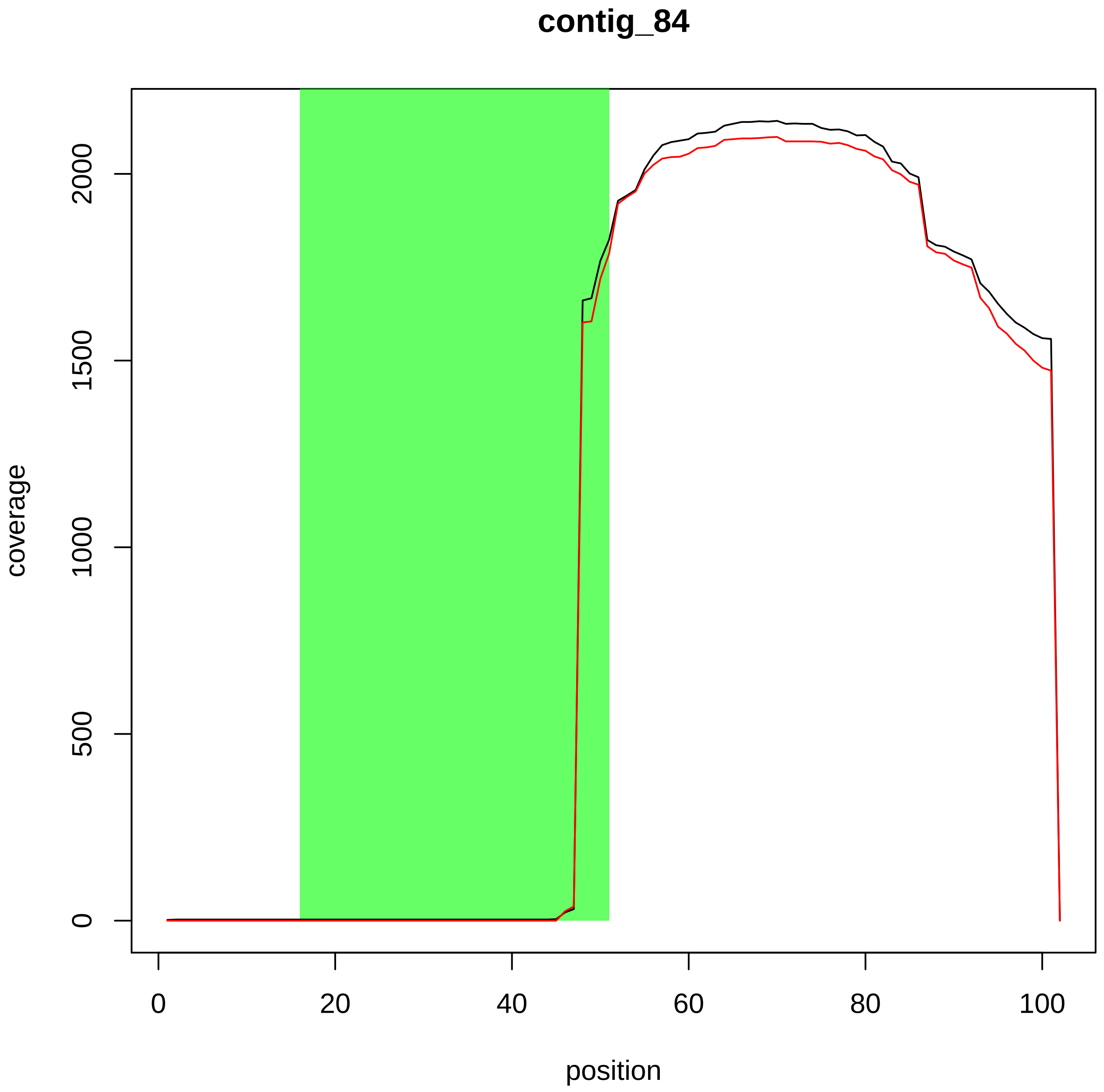
contig_84
coverage
0
500
1000
1500
2000
position
0
20
40
60
80
100

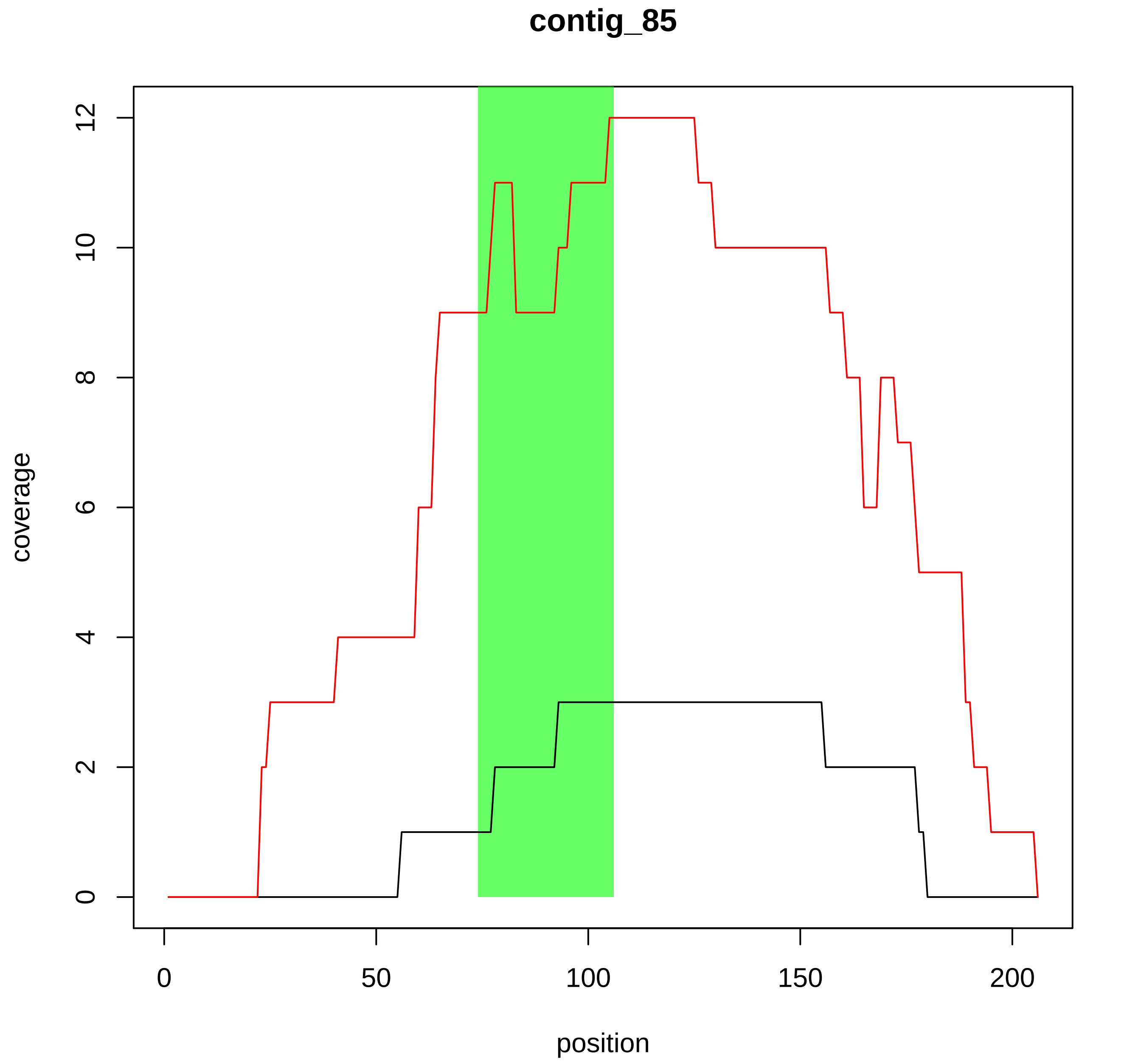
contig_85
coverage
position
0
2
4
6
8
10
12
0
50
100
150
200

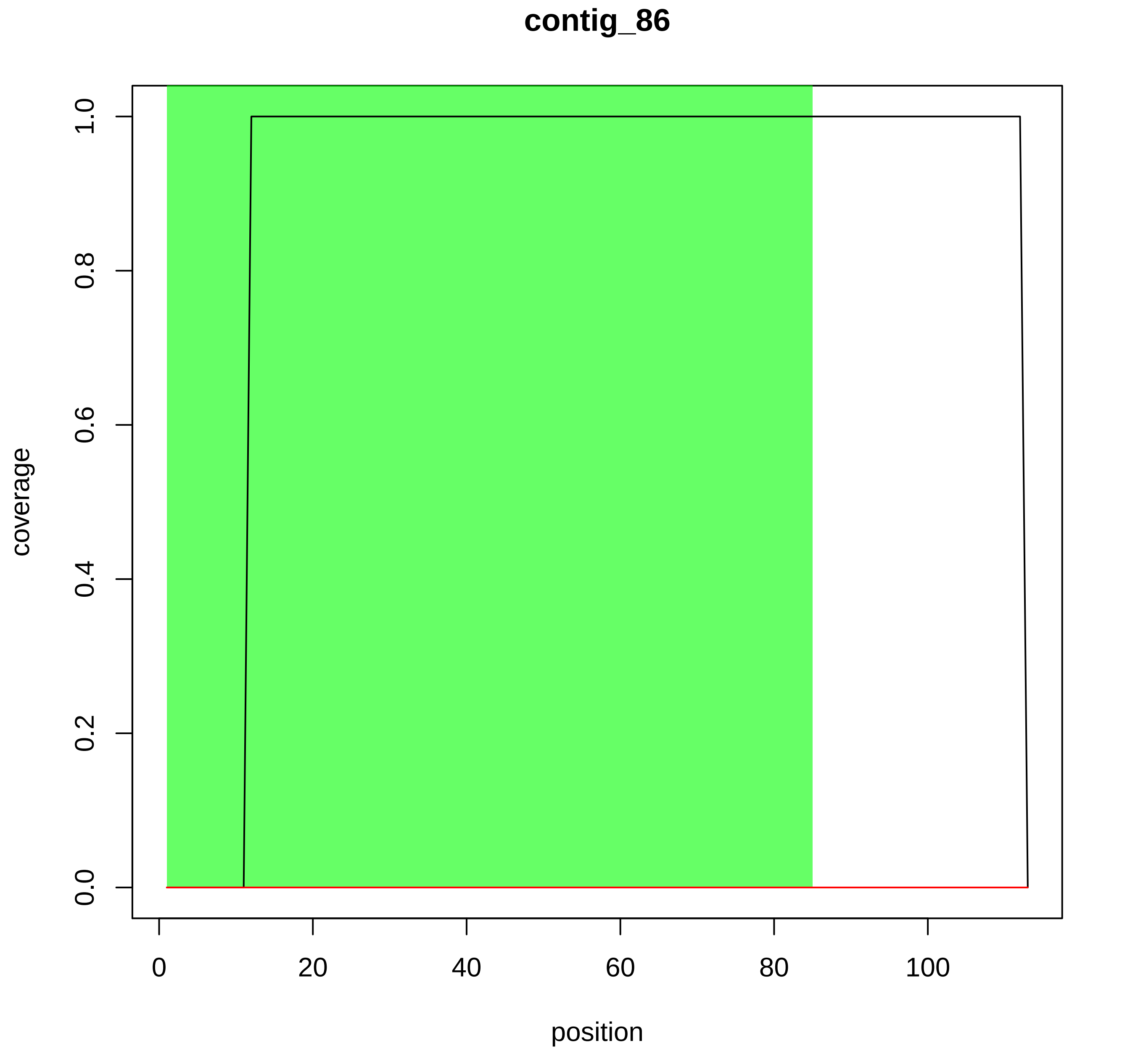
contig_86
coverage
position
0.0
0.2
0.4
0.6
0.8
1.0
0
20
40
60
80
100

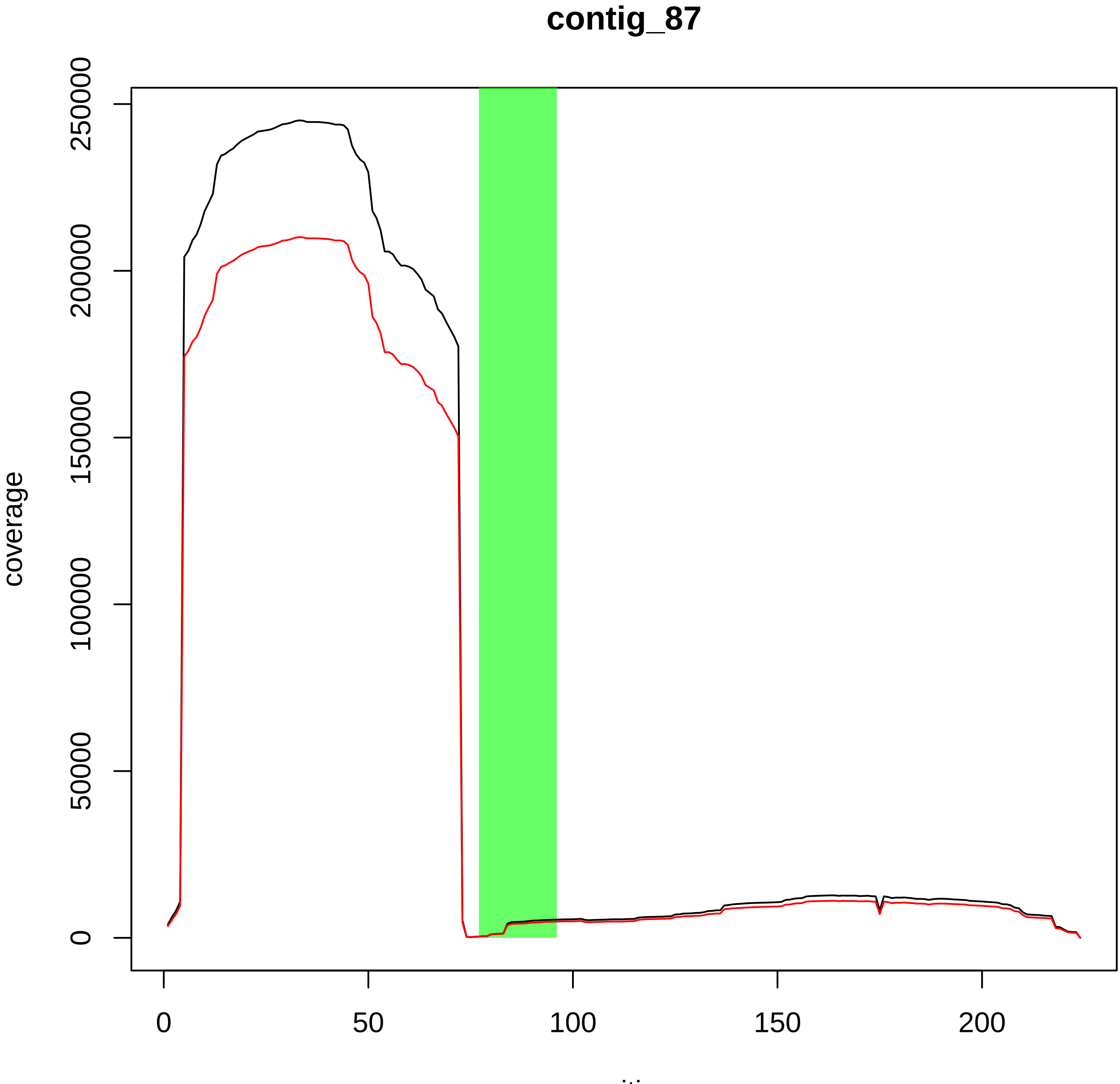
contig_87
coverage
0
50000
100000
150000
200000
250000
0
50
100
150
200
position

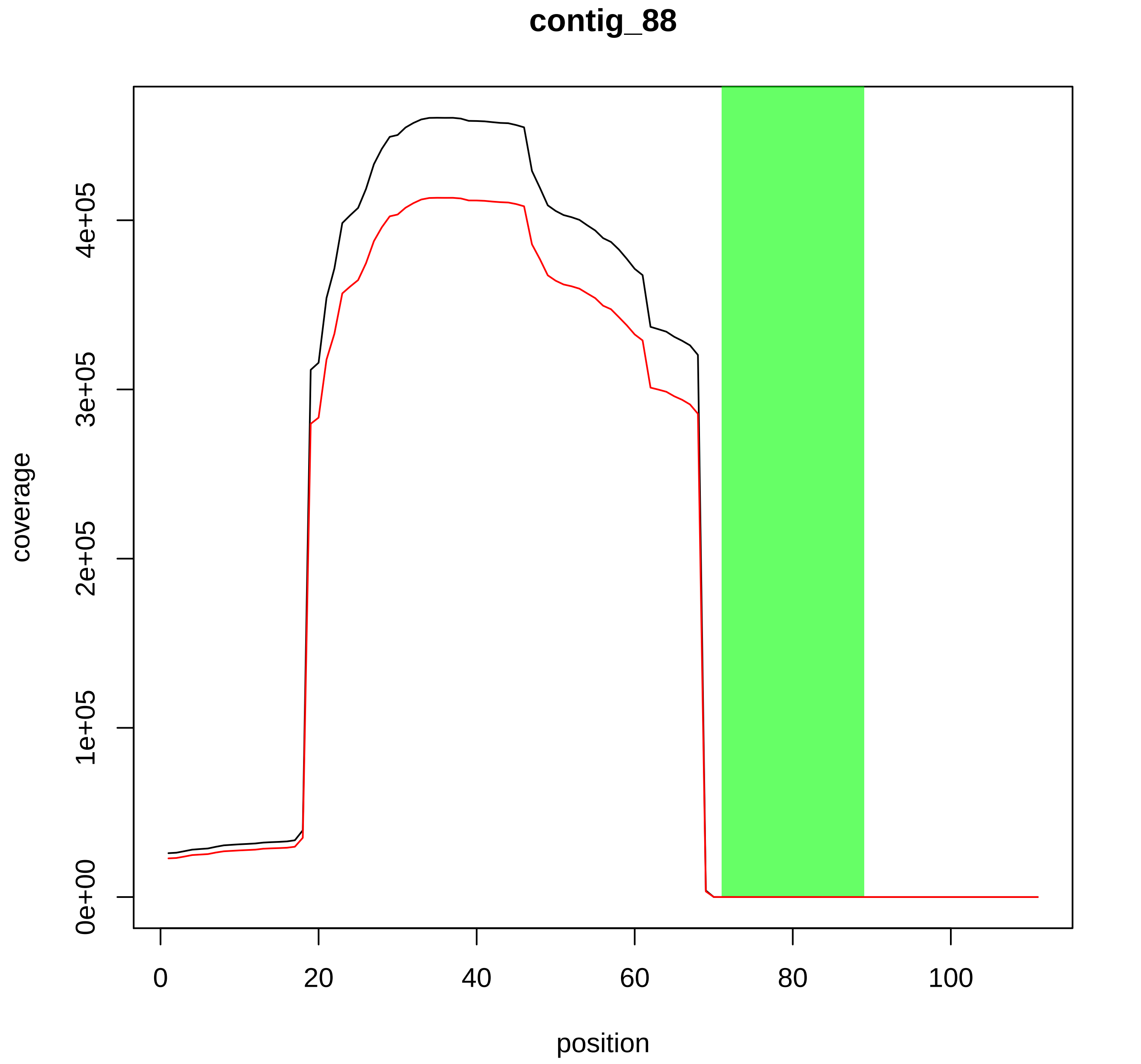

contig_88
coverage
0e+00
1e+05
2e+05
3e+05
4e+05
position
0
20
40
60
80
100

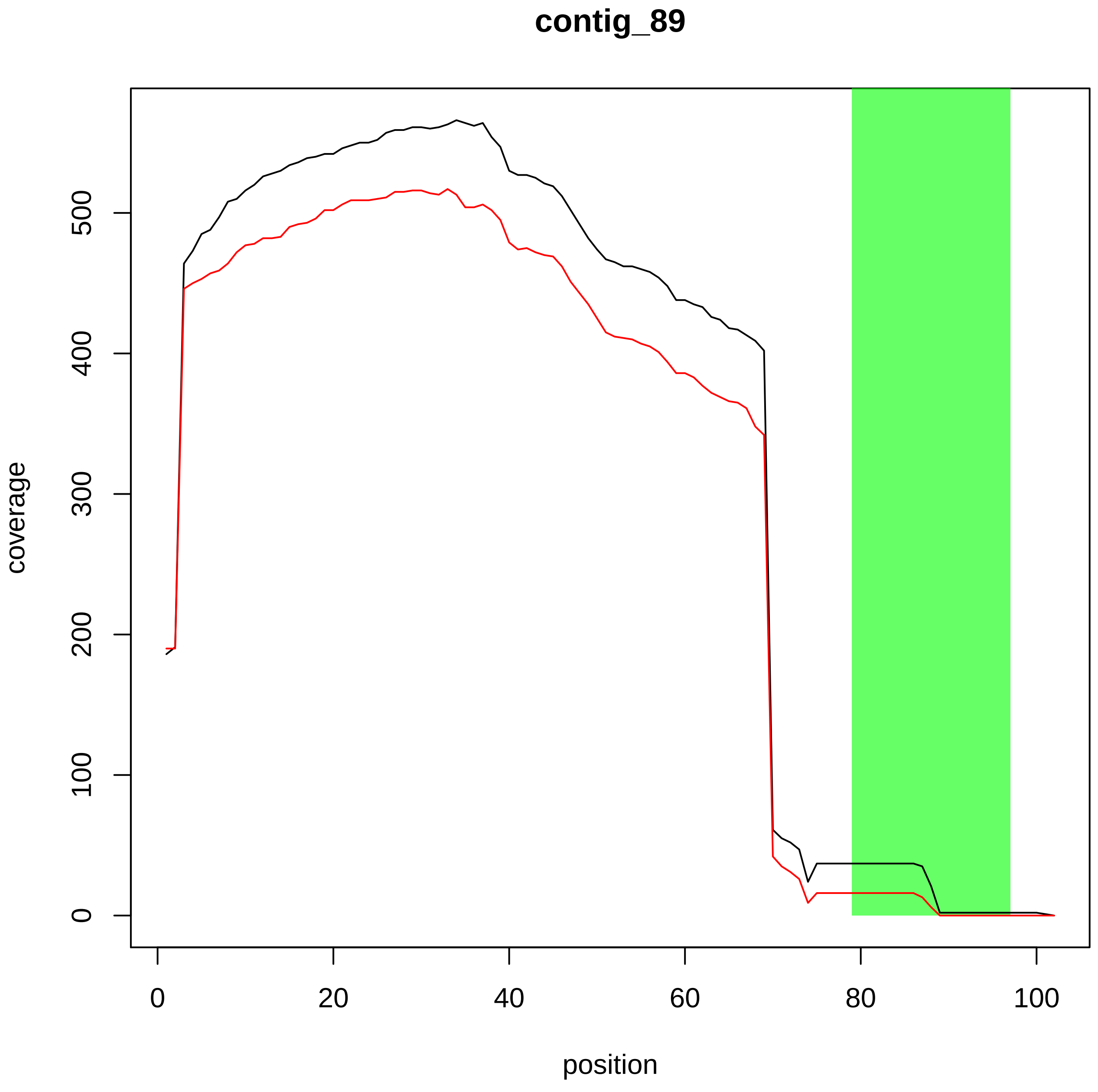
contig_89
coverage
0
100
200
300
400
500
position
0
20
40
60
80
100

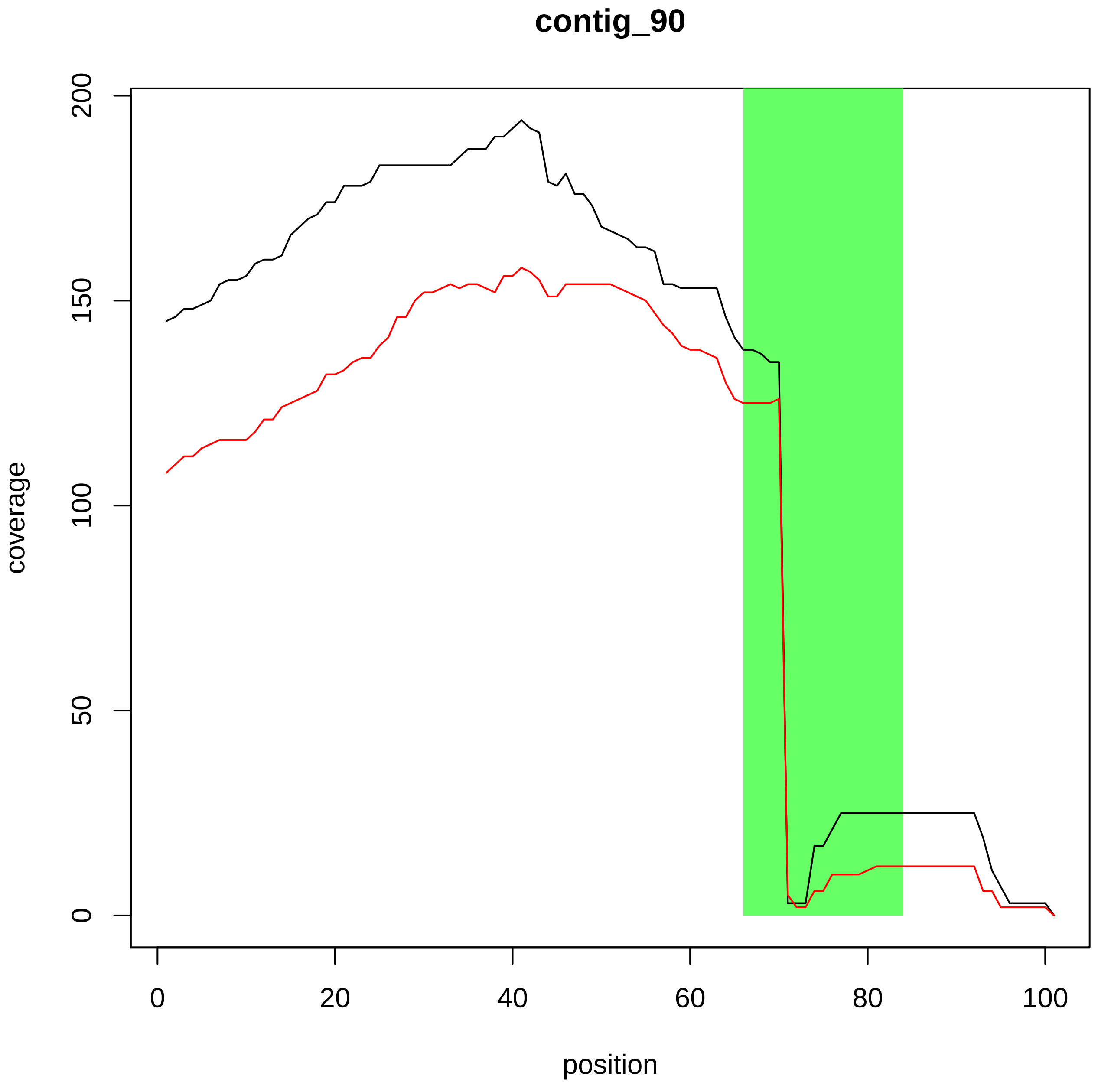
contig_90
coverage
0
50
100
150
200
position
0
20
40
60
80
100

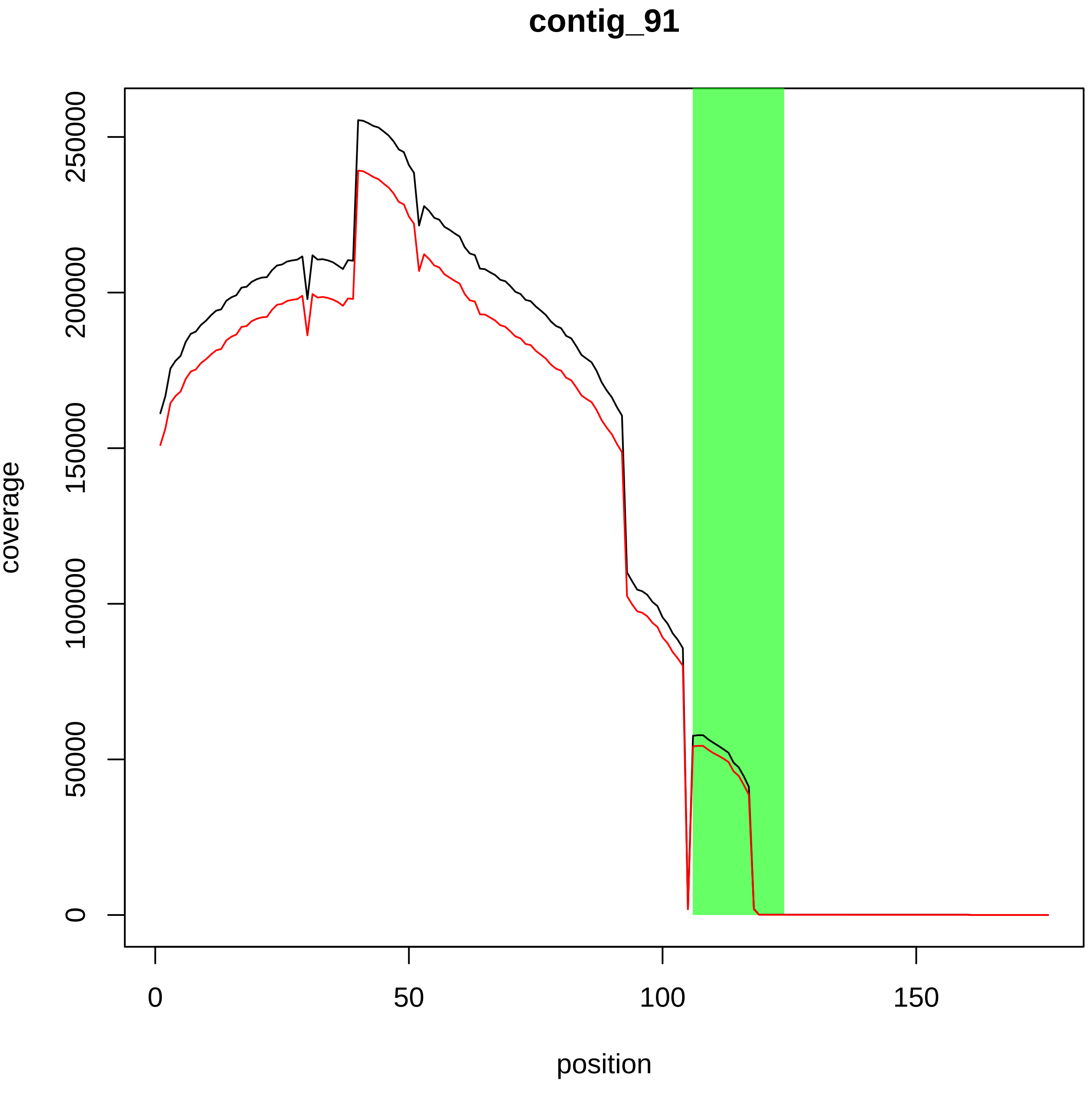

contig_91
coverage
position
0
50000
100000
150000
200000
250000
0
50
100
150

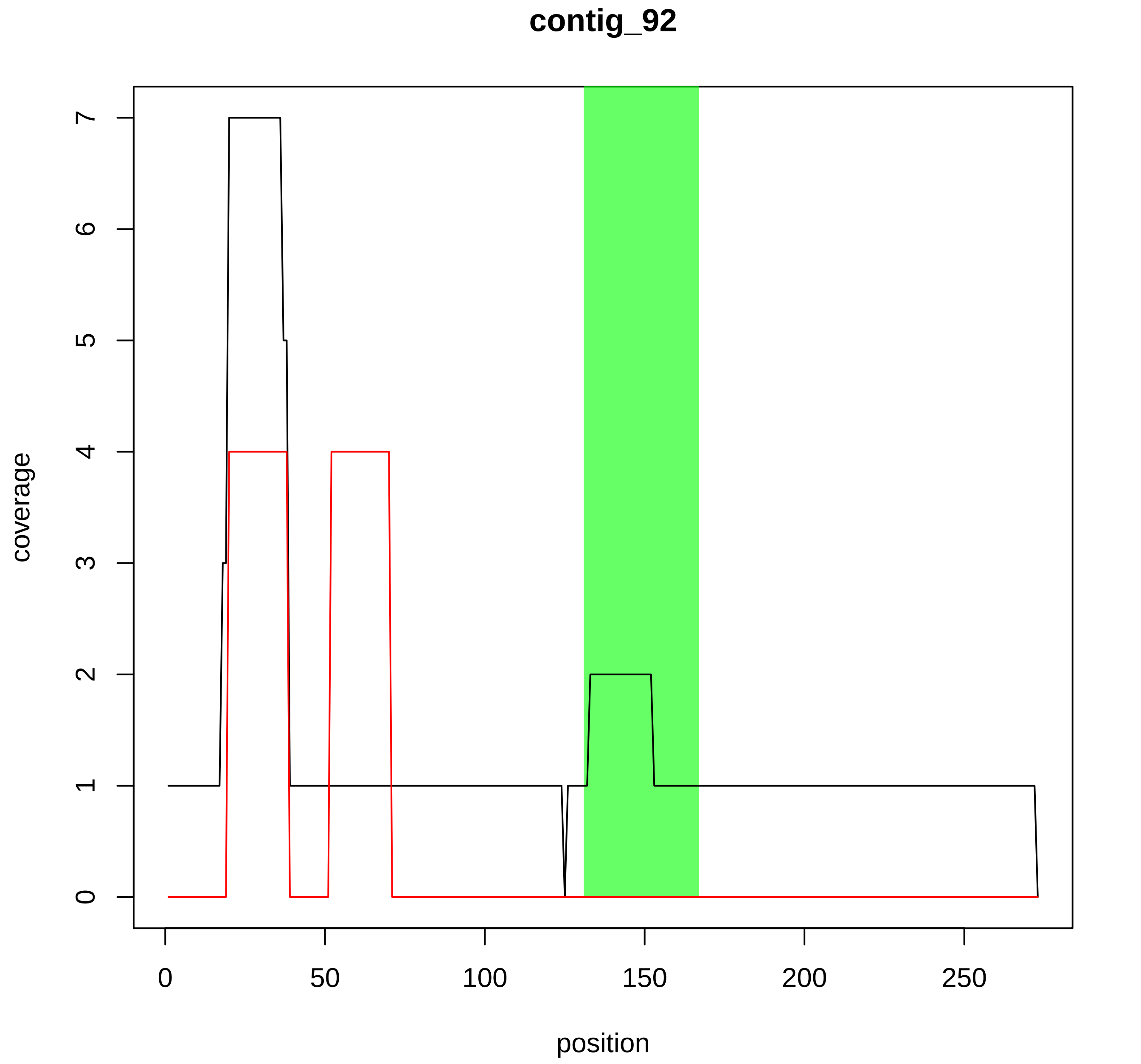
contig_92
coverage
position
0
1
2
3
4
5
6
7
0
50
100
150
200
250

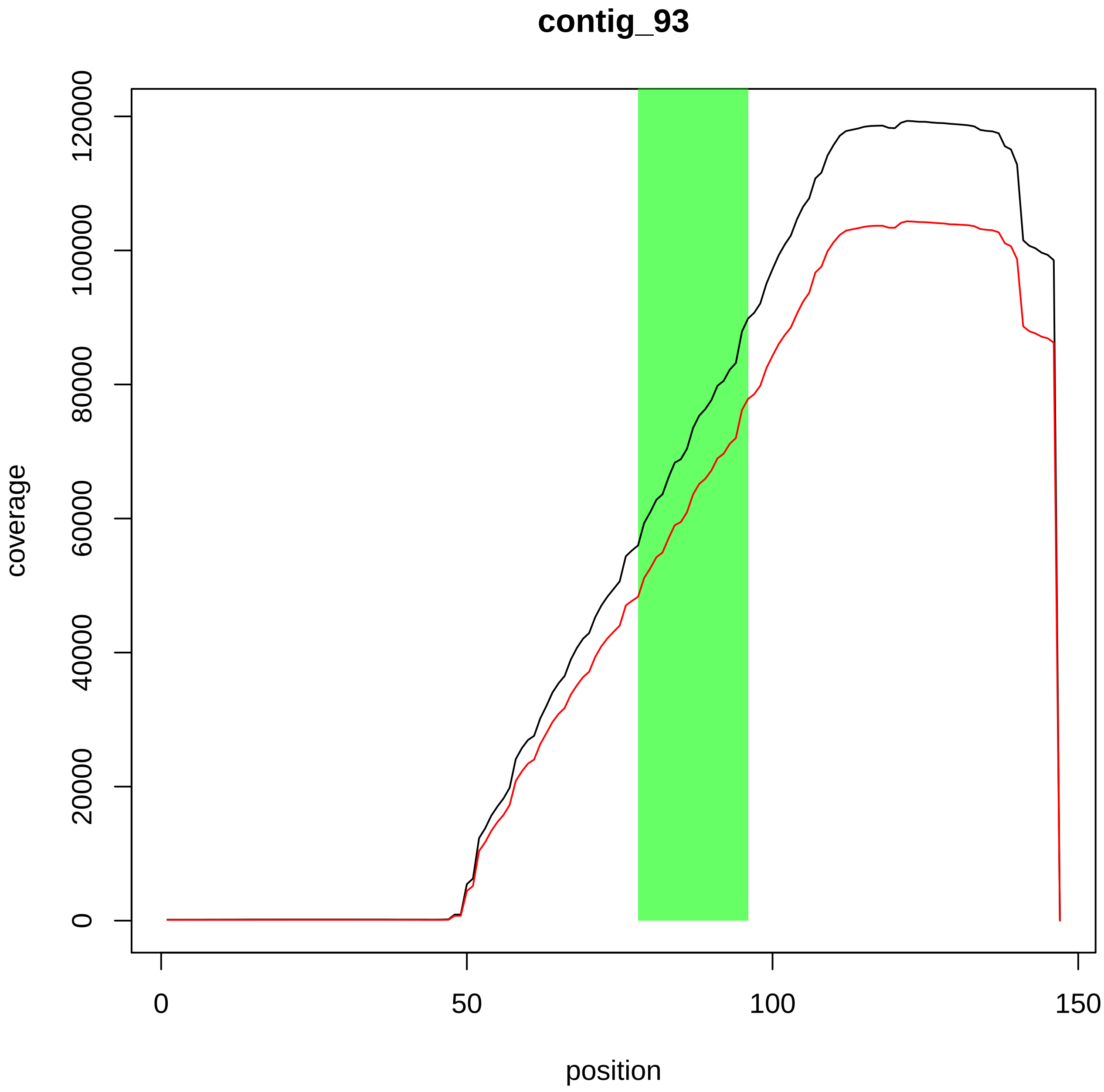

contig_93
coverage
position
0
20000
40000
60000
80000
100000
120000
0
50
100
150

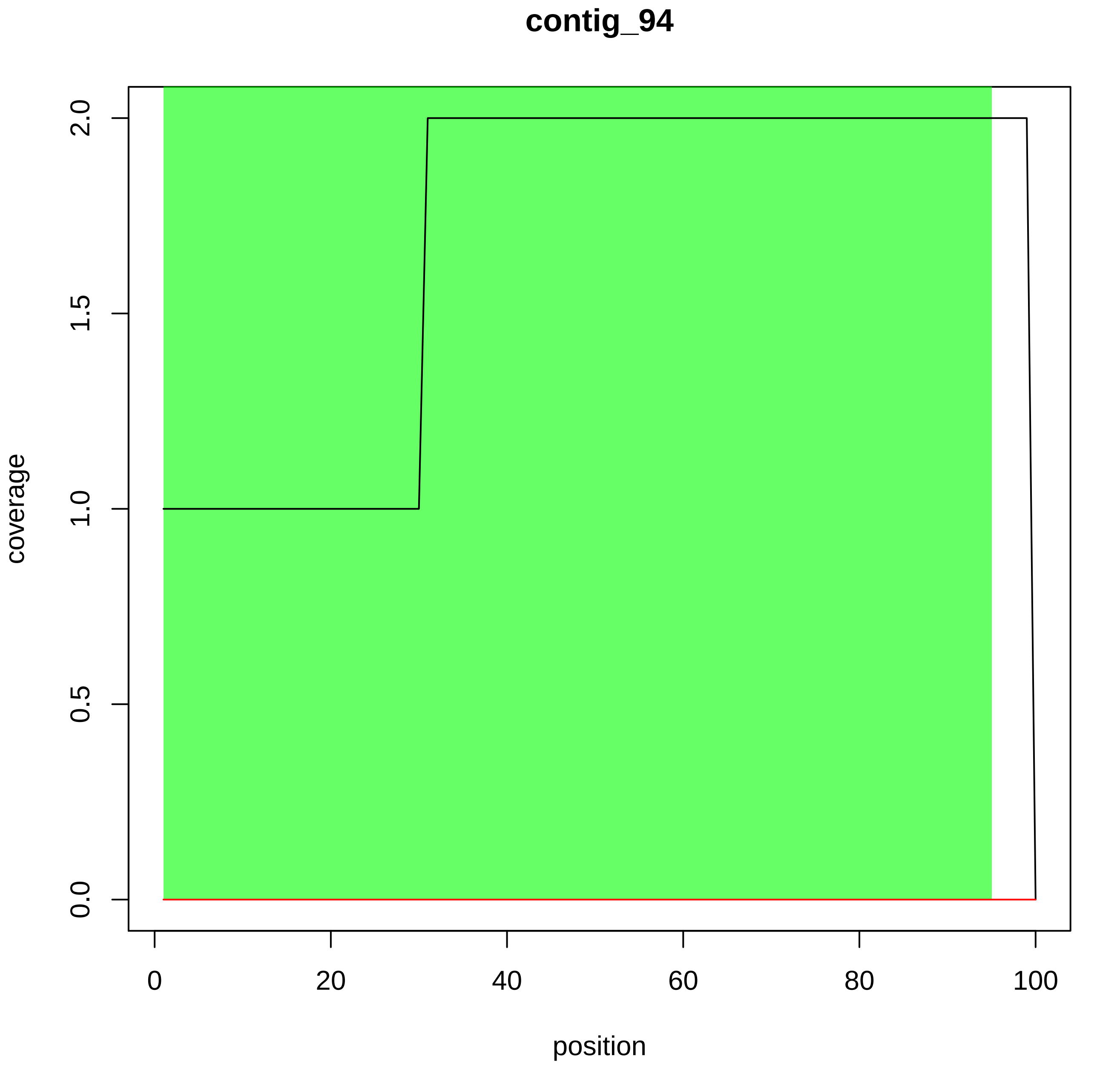
contig_94
coverage
2.0
1.5
1.0
0.5
0.0
0
20
40
60
80
100
position

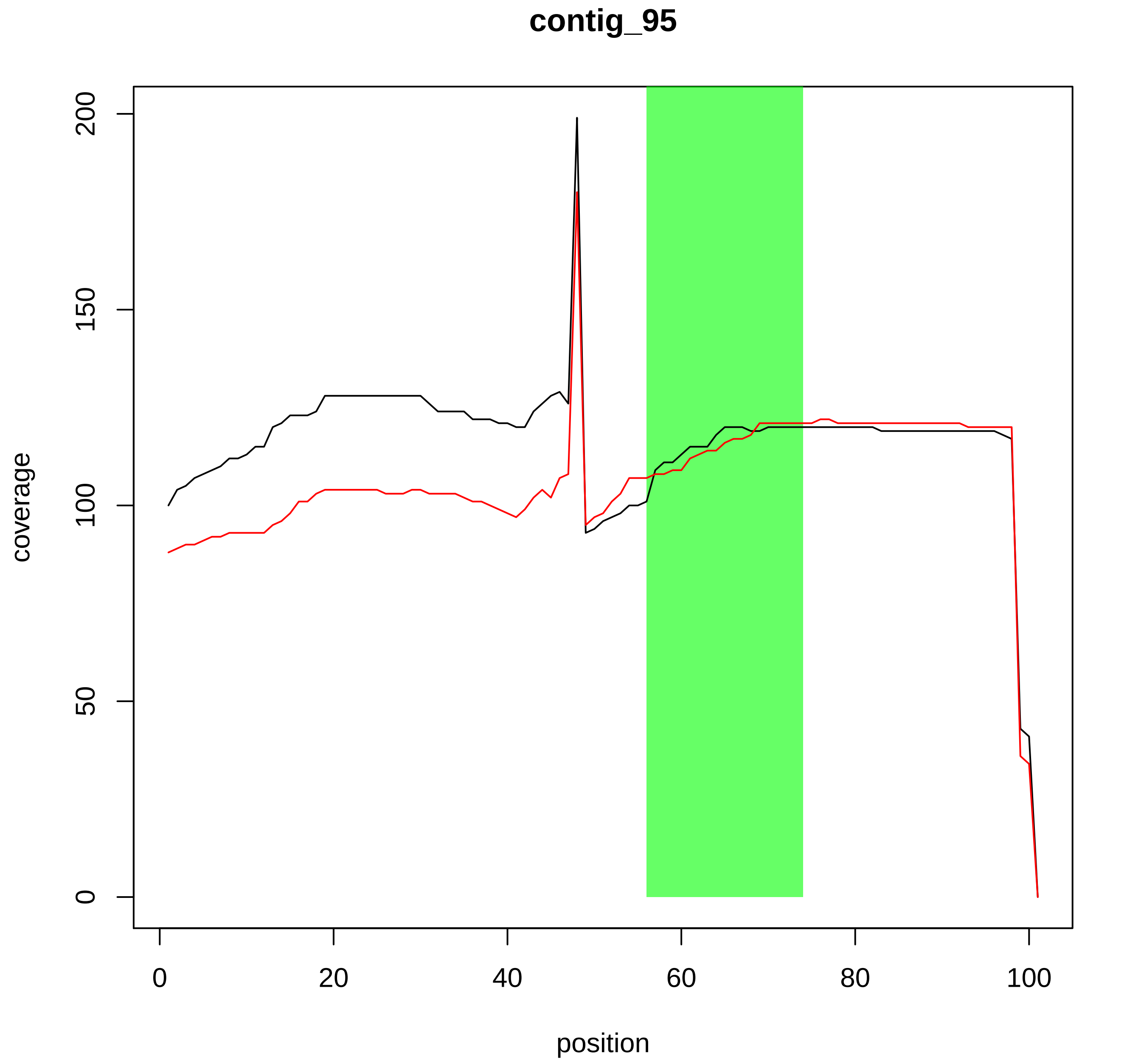
contig_95
coverage
position
0
50
100
150
200
0
20
40
60
80
100

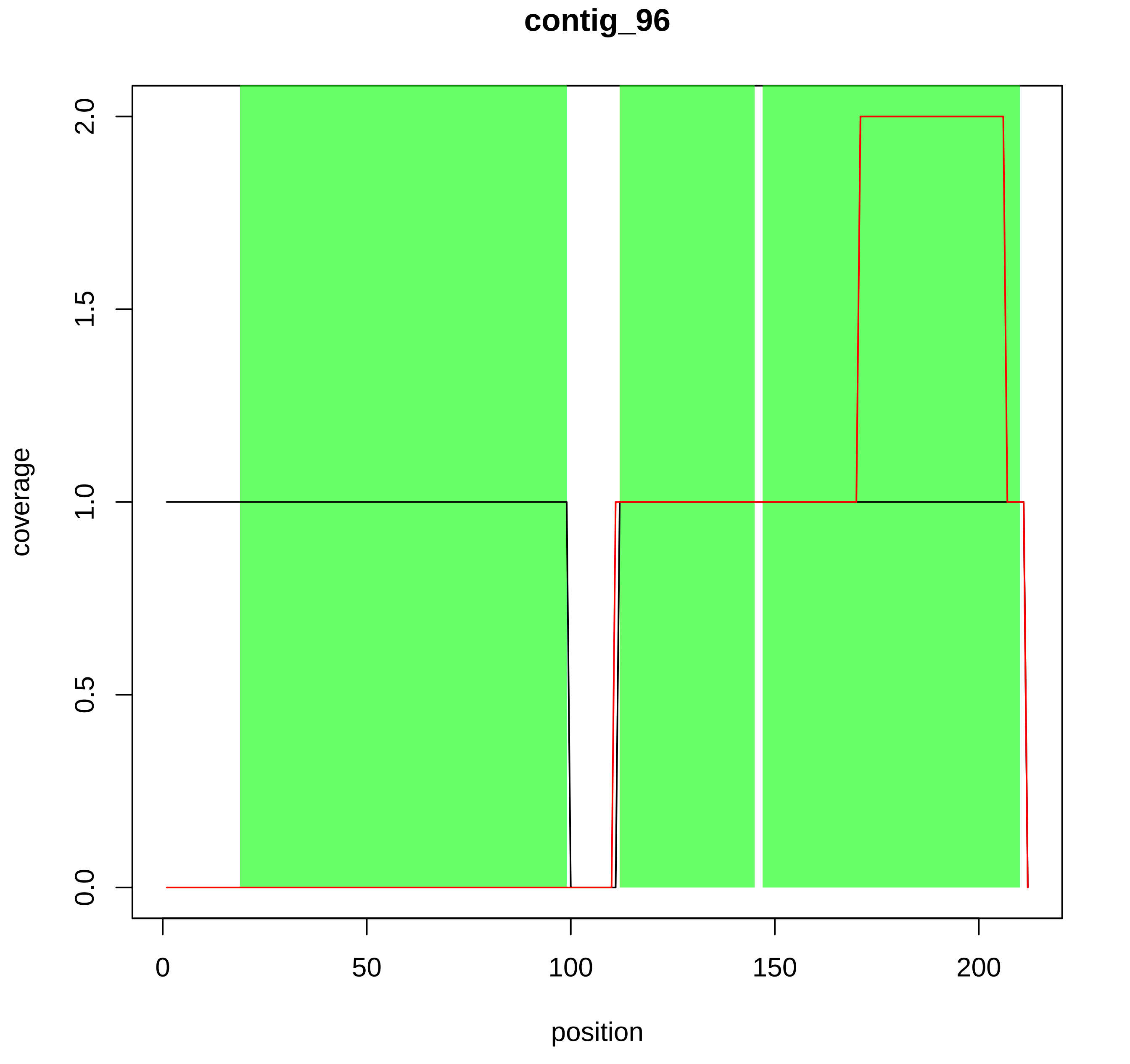
contig_96
coverage
0.0
0.5
1.0
1.5
2.0
0
50
100
150
200
position

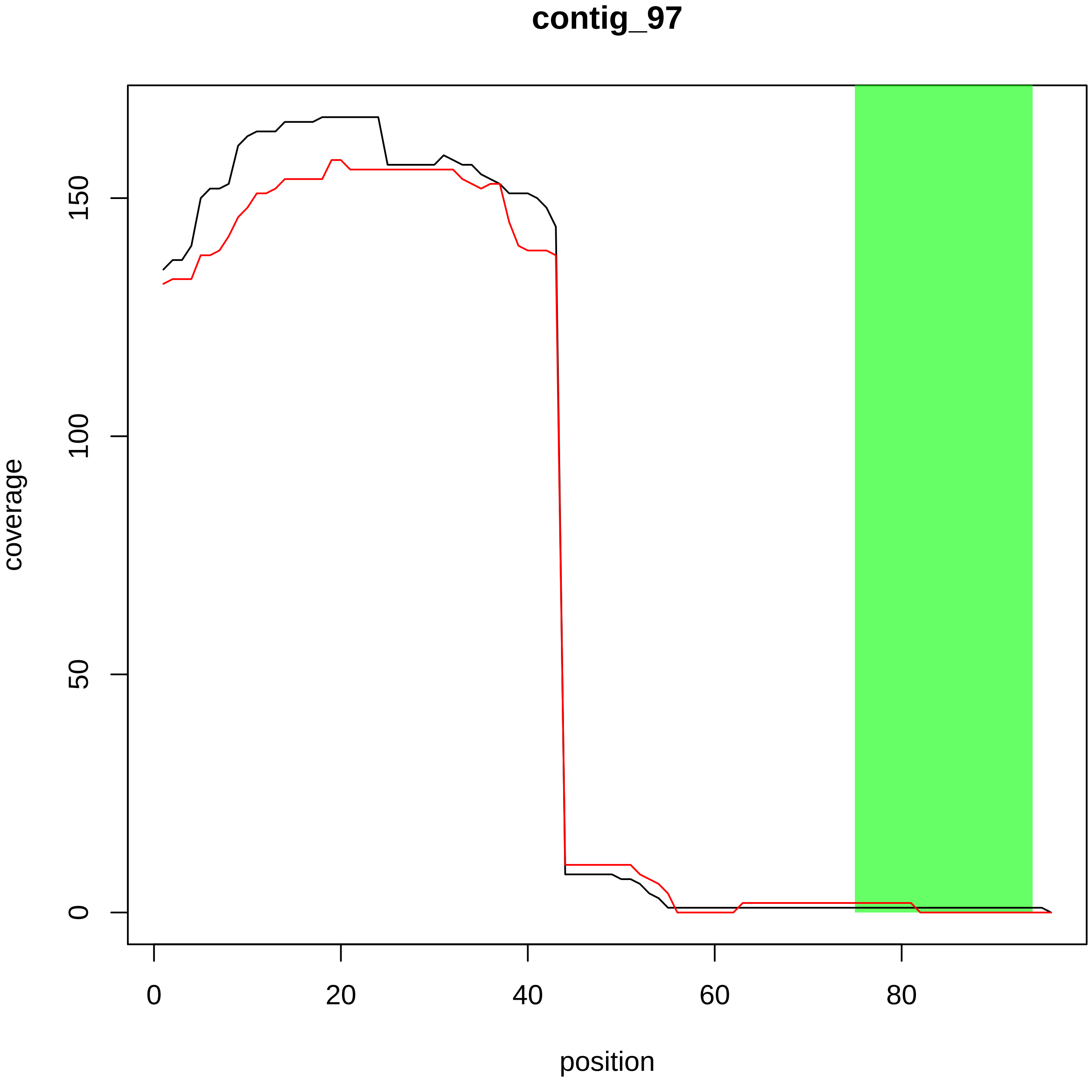
contig_97
coverage
0
50
100
150
position
0
20
40
60
80

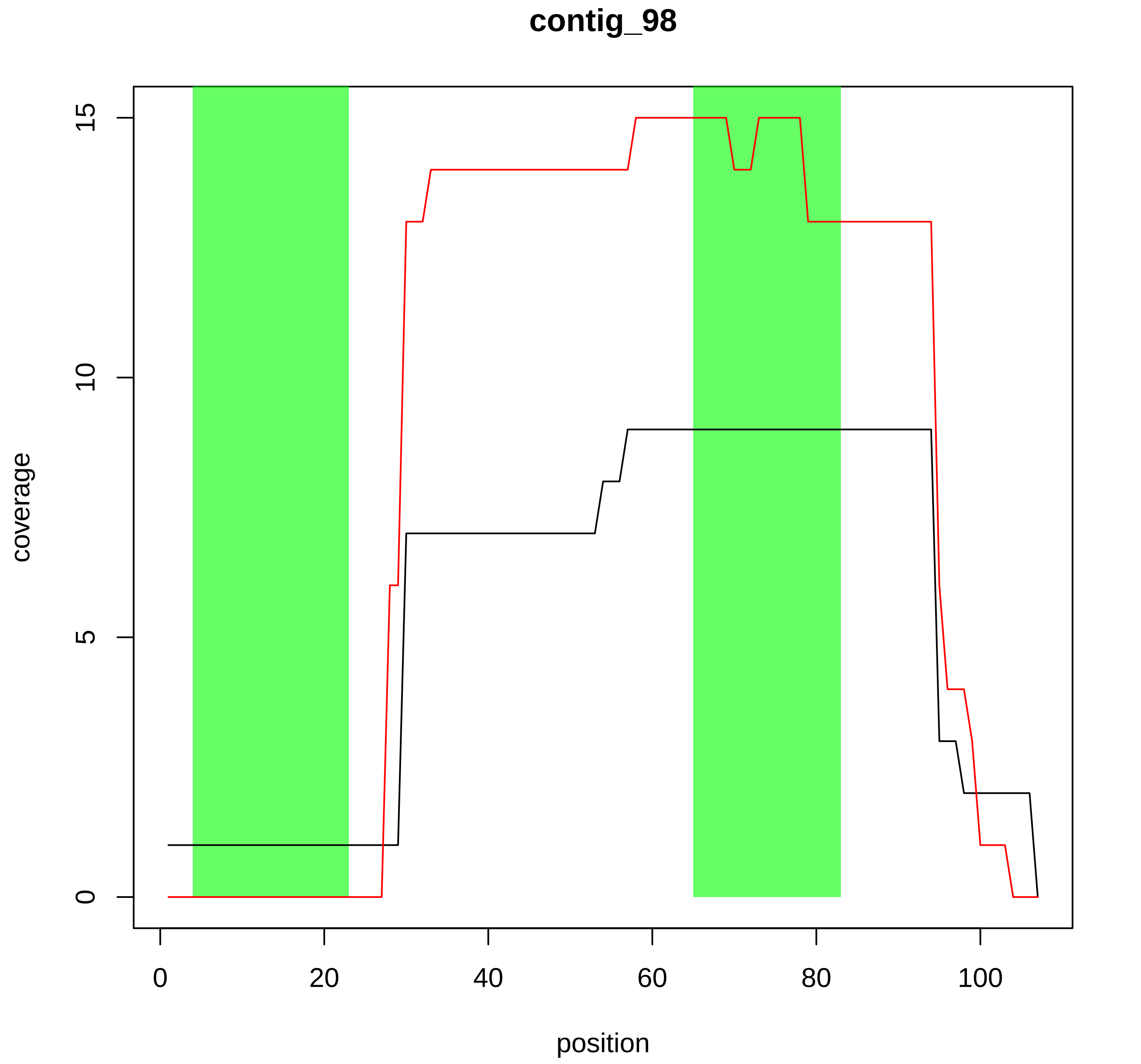
contig_98
coverage
position
0
5
10
15
0
20
40
60
80
100

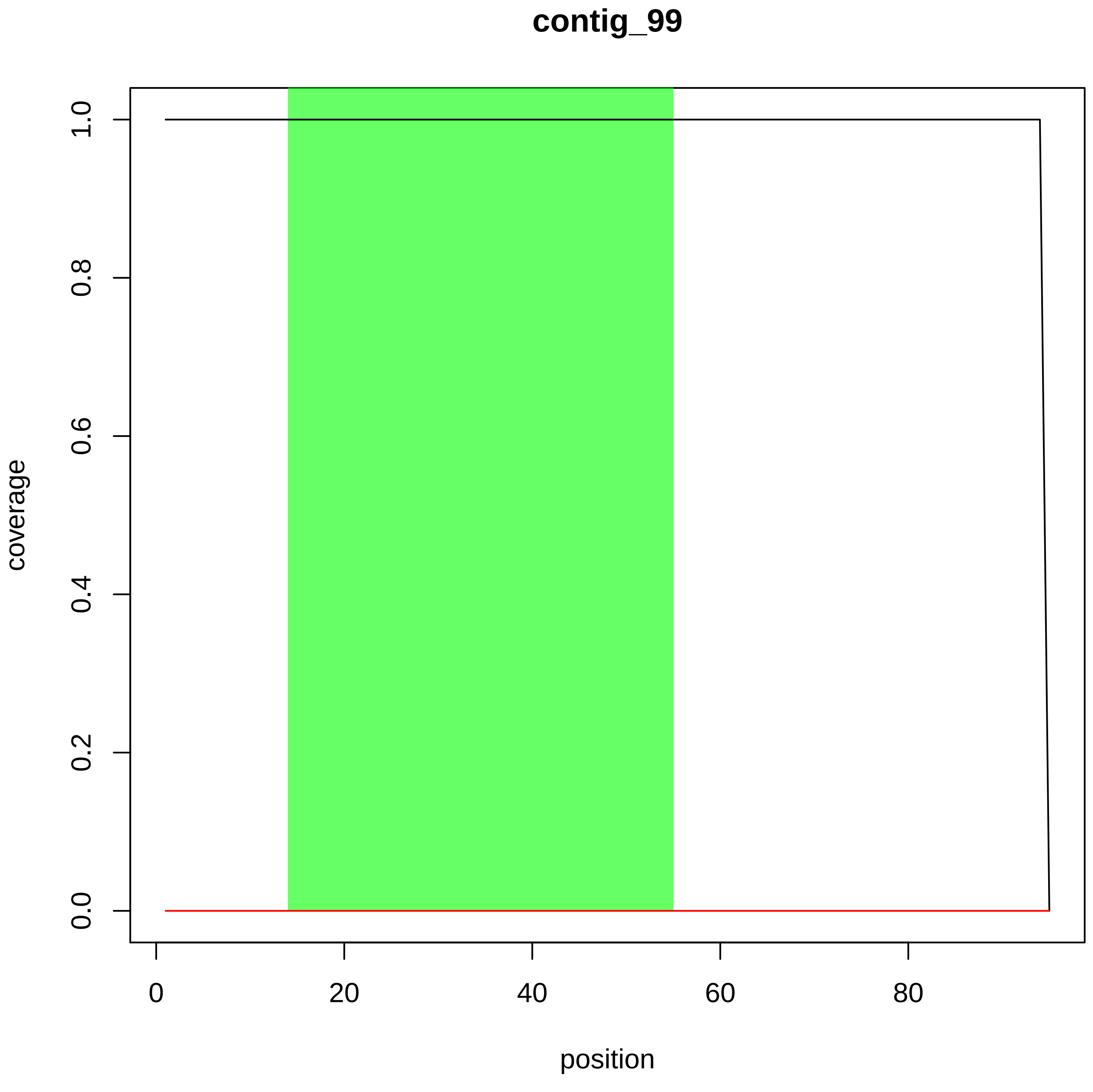
contig_99
coverage
0.0
0.2
0.4
0.6
0.8
1.0
0
20
40
60
80
position

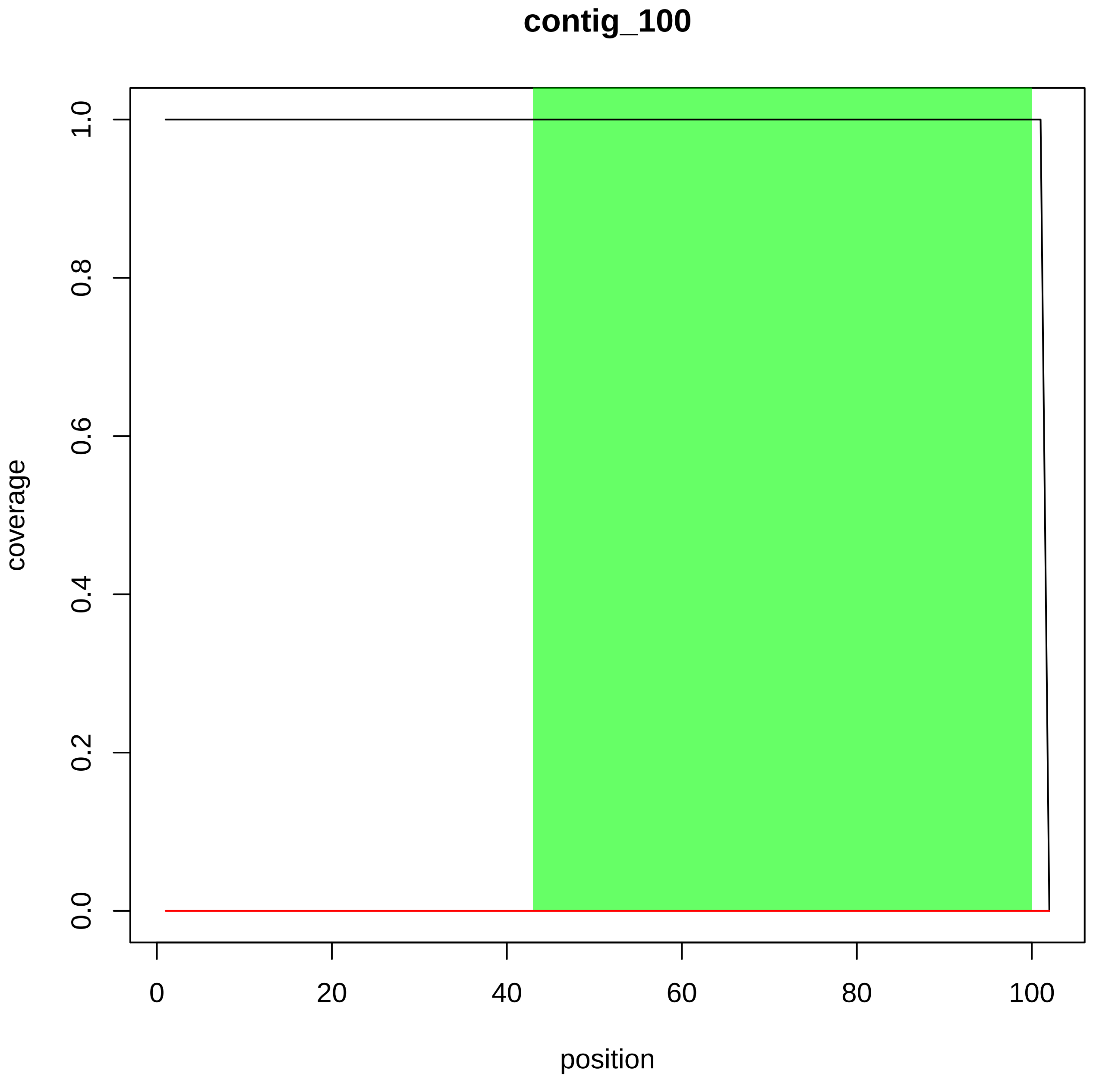

contig_100
coverage
1.0
0.8
0.6
0.4
0.2
0.0
0
20
40
60
80
100
position

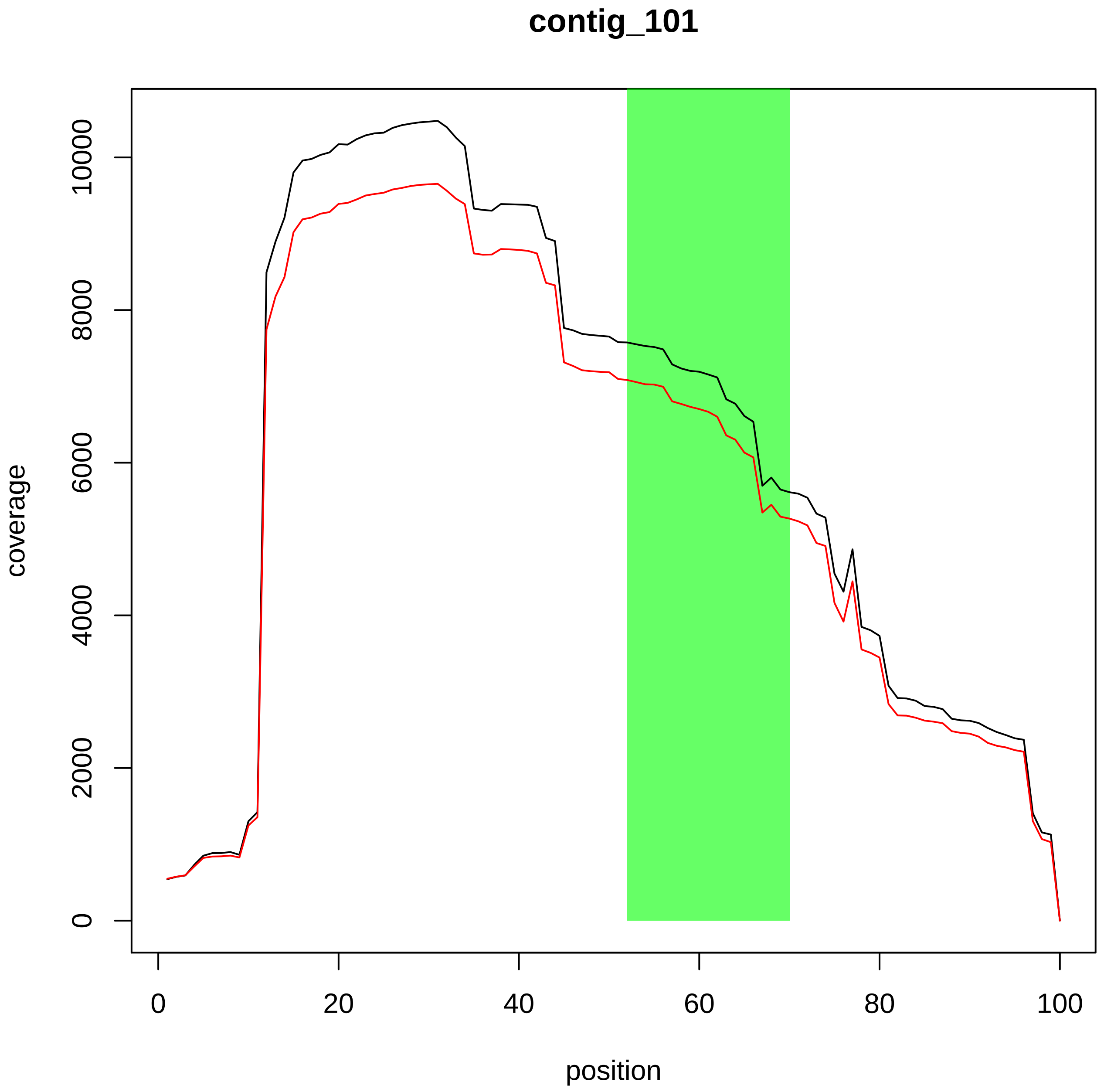
contig_101
coverage
0
2000
4000
6000
8000
10000
position
0
20
40
60
80
100

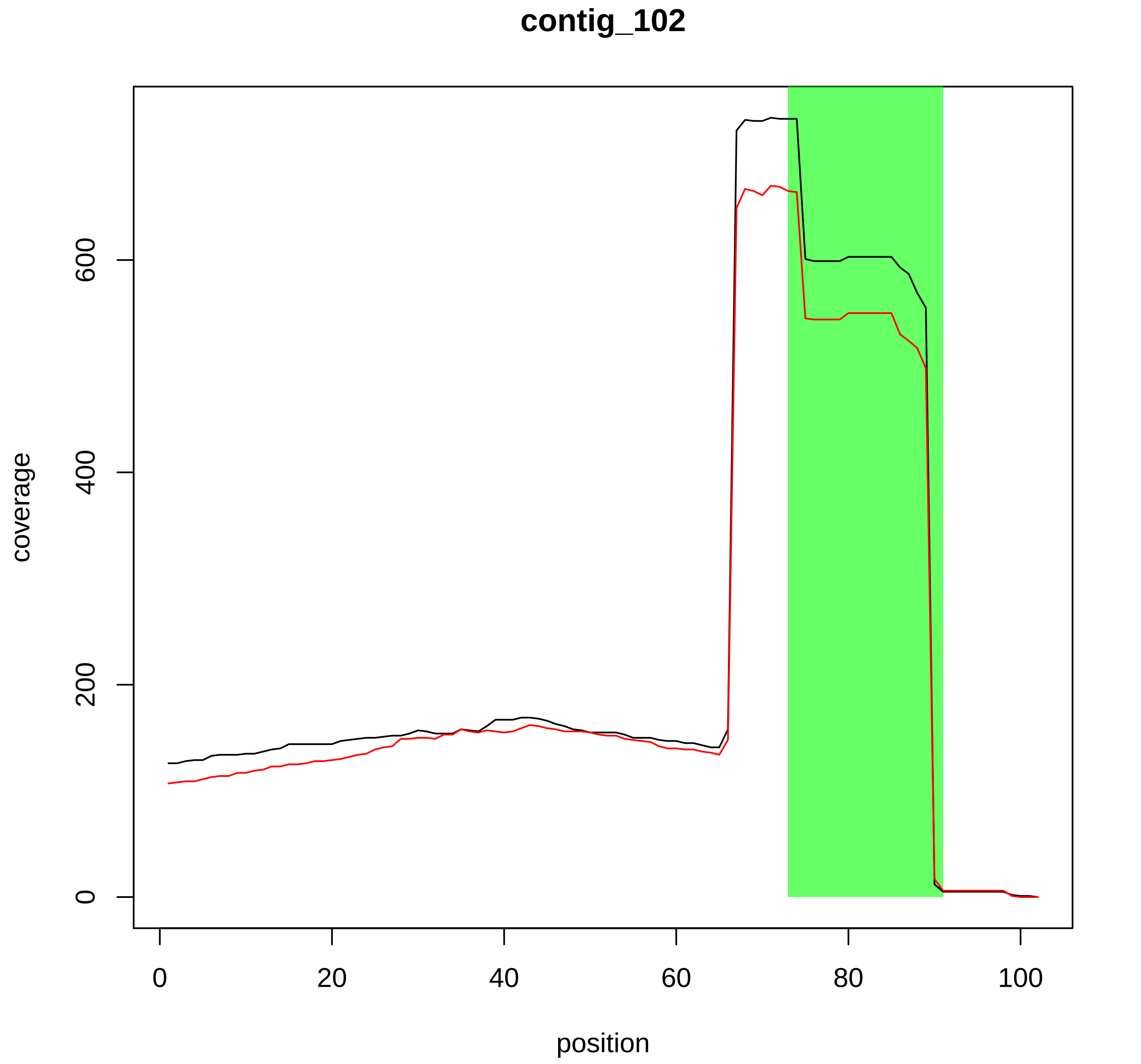
contig_102
coverage
600
400
200
0
0
20
40
60
80
100
position

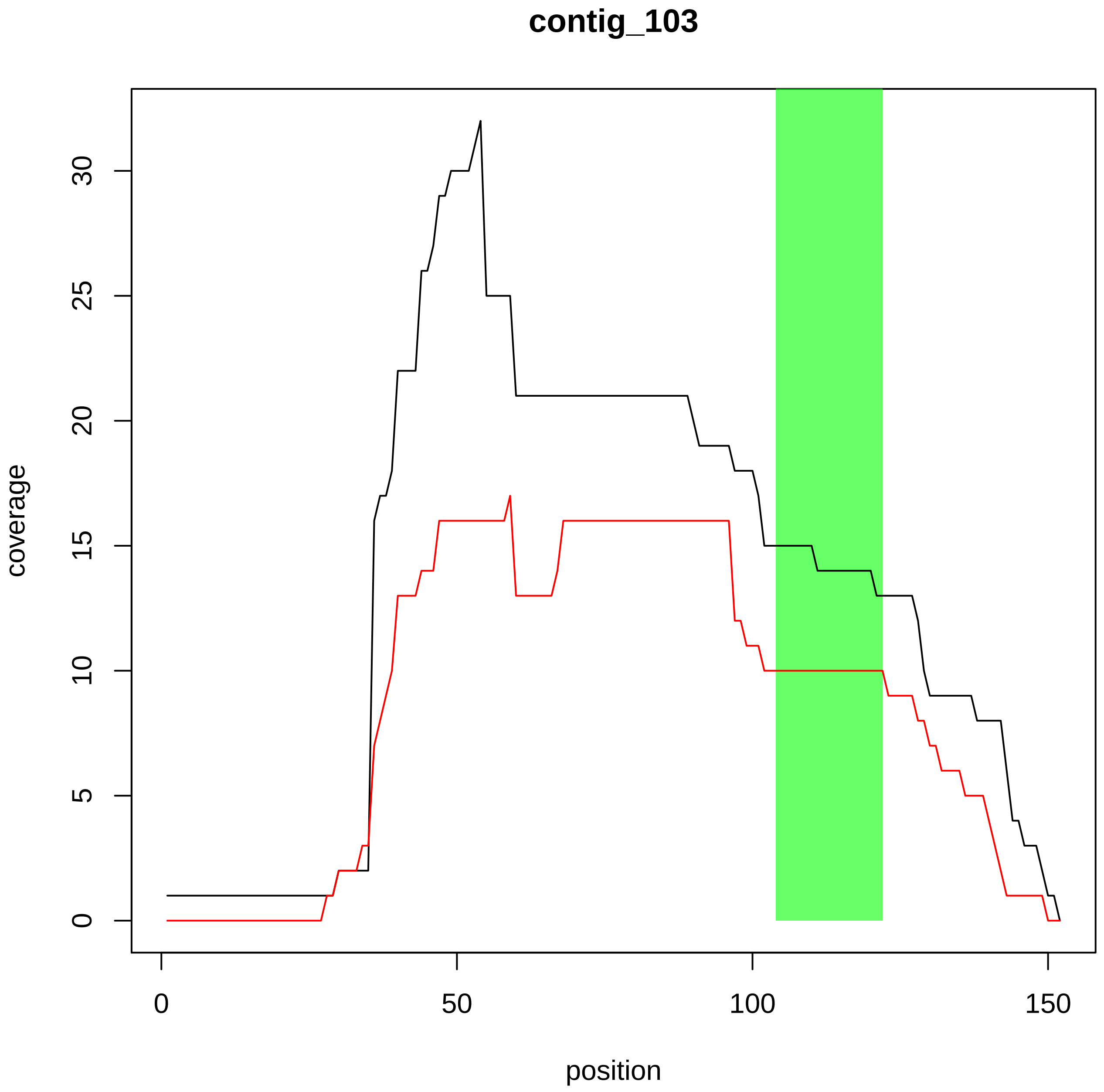

contig_103
coverage
position
0
5
10
15
20
25
30
0
50
100
150

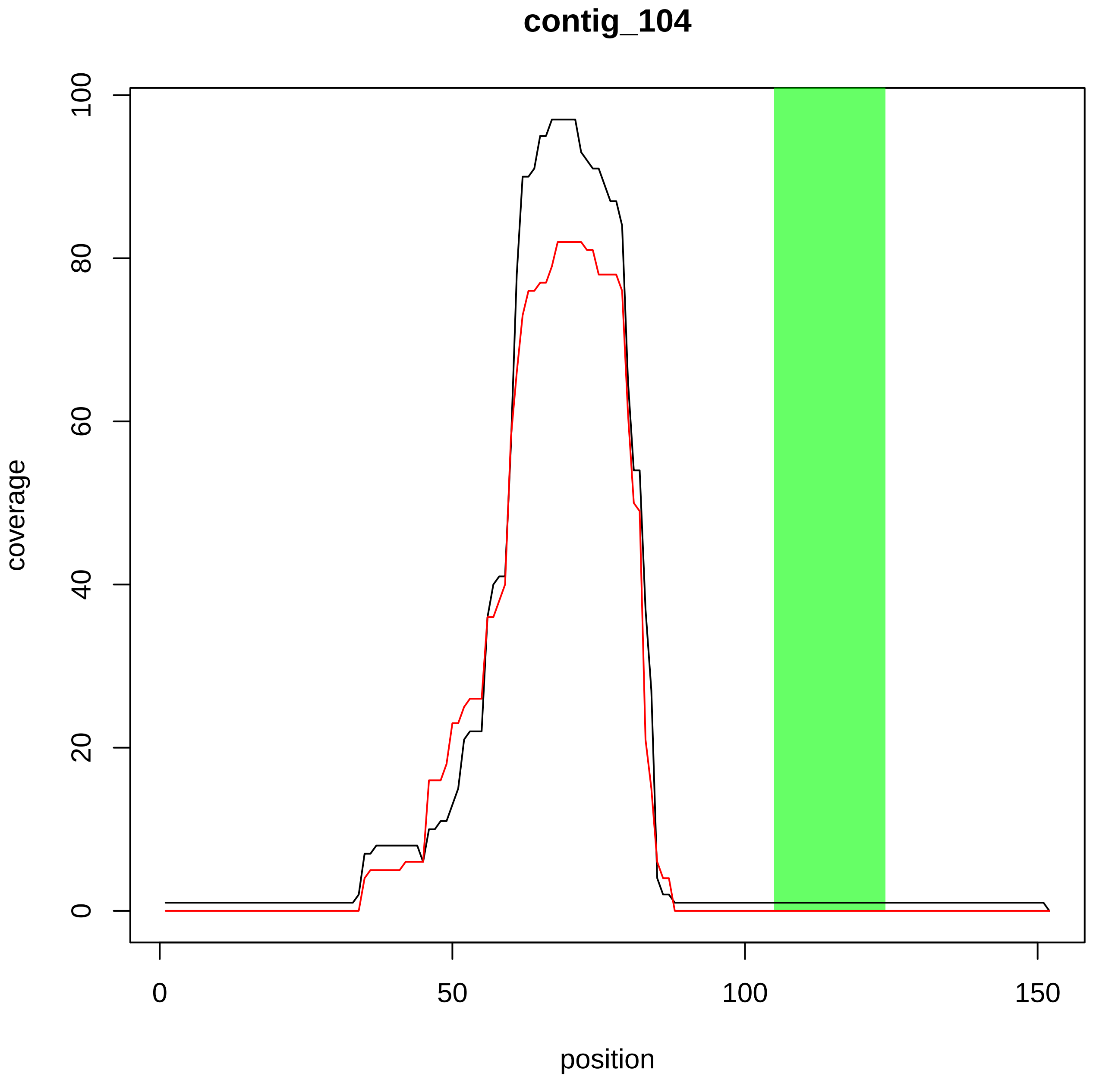
contig_104
coverage
position
0
20
40
60
80
100
0
50
100
150

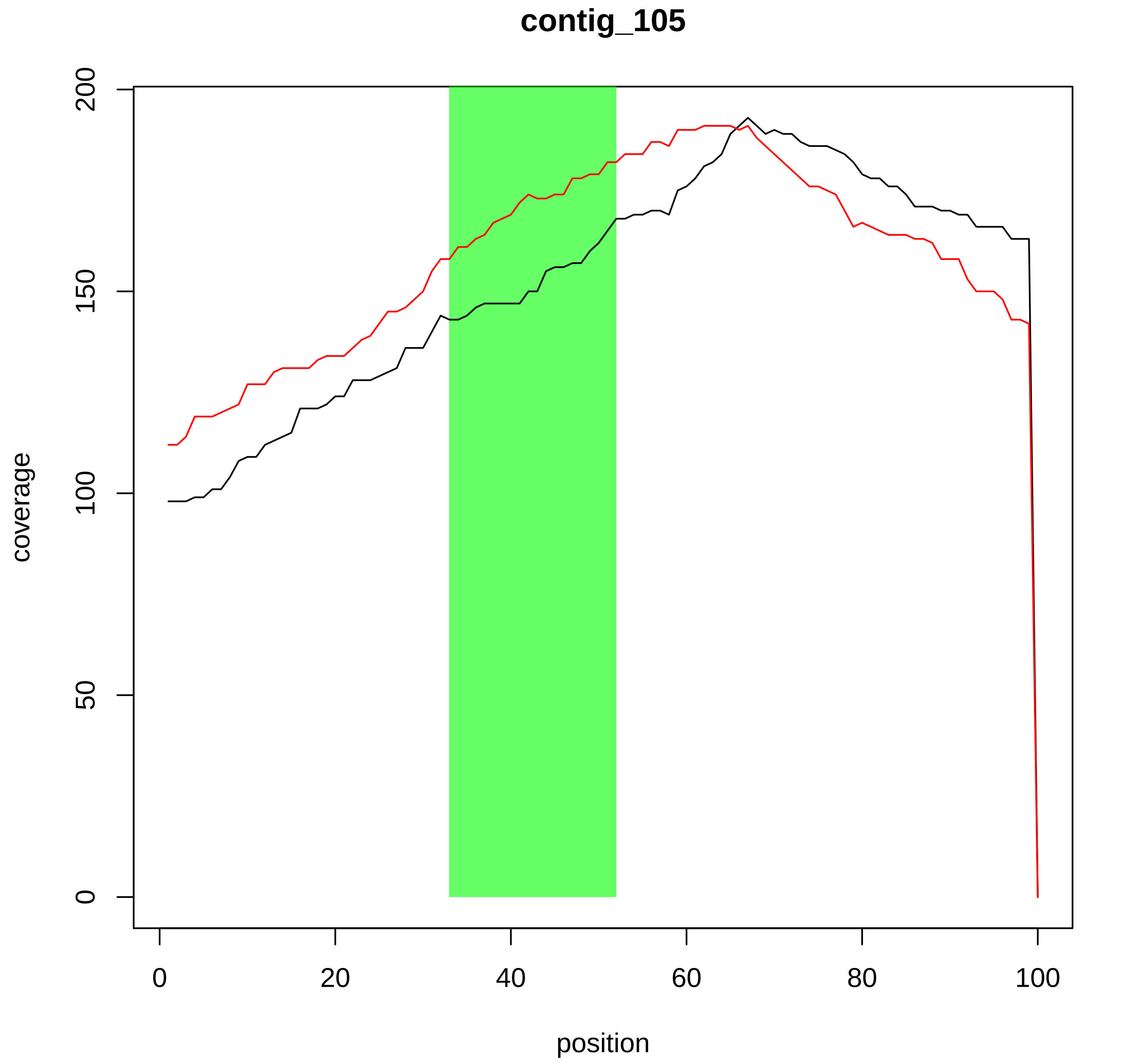
contig_105
coverage
position
0
50
100
150
200
0
20
40
60
80
100

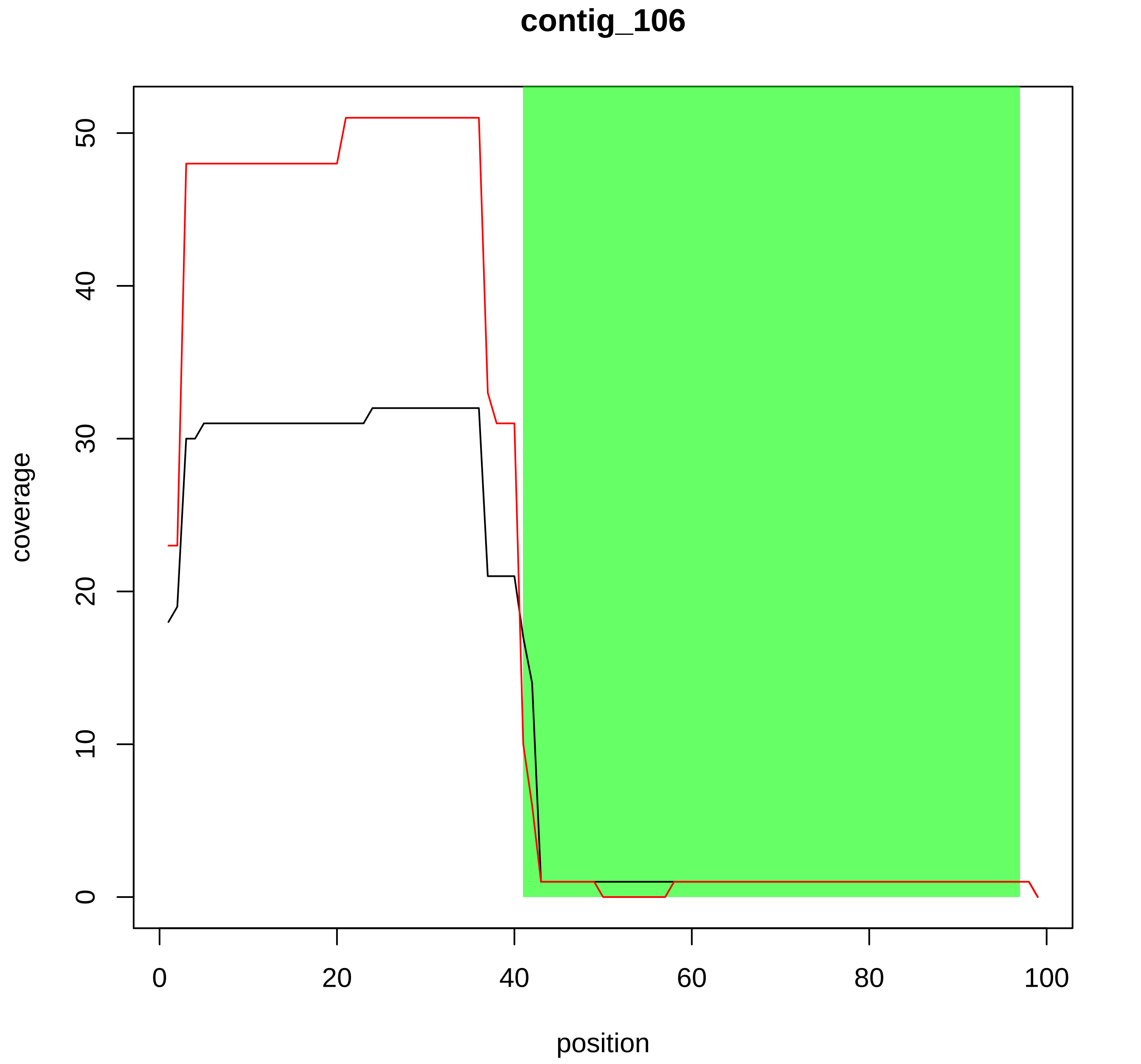
contig_106
coverage
position
0
10
20
30
40
50
0
20
40
60
80
100

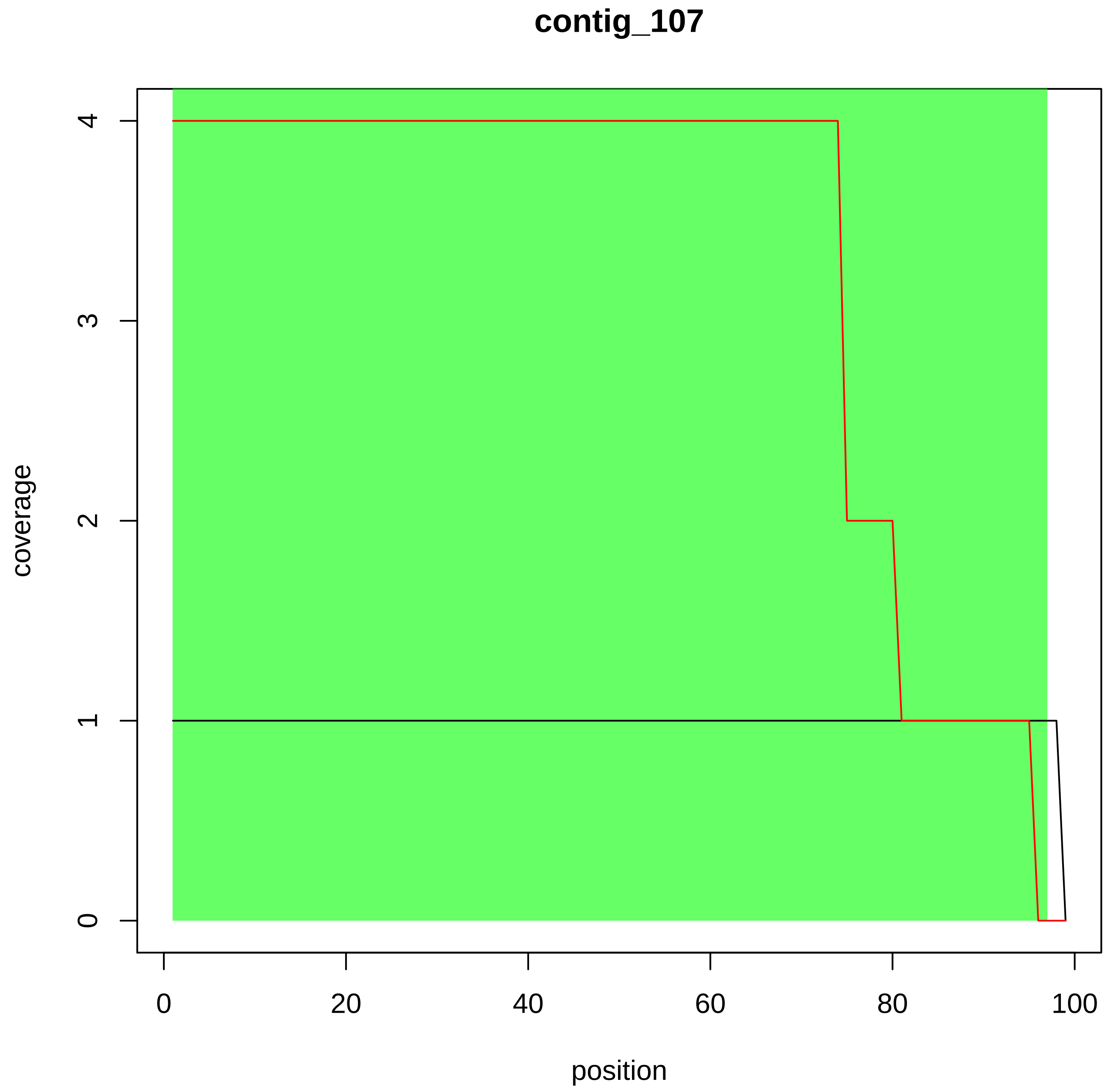
contig_107
coverage
position
0
1
2
3
4
0
20
40
60
80
100

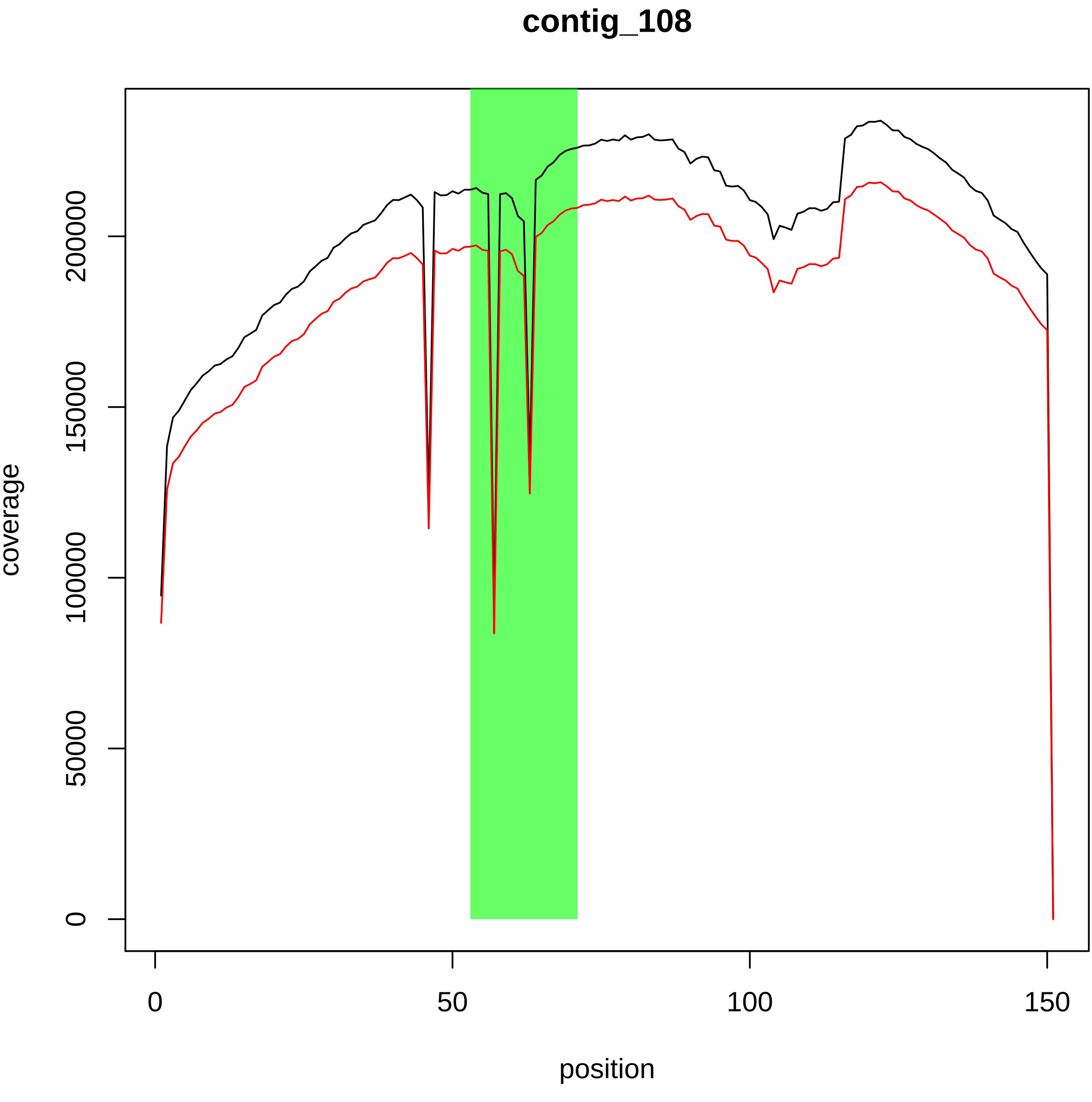

contig_108
coverage
0
50000
100000
150000
200000
position
0
50
100
150

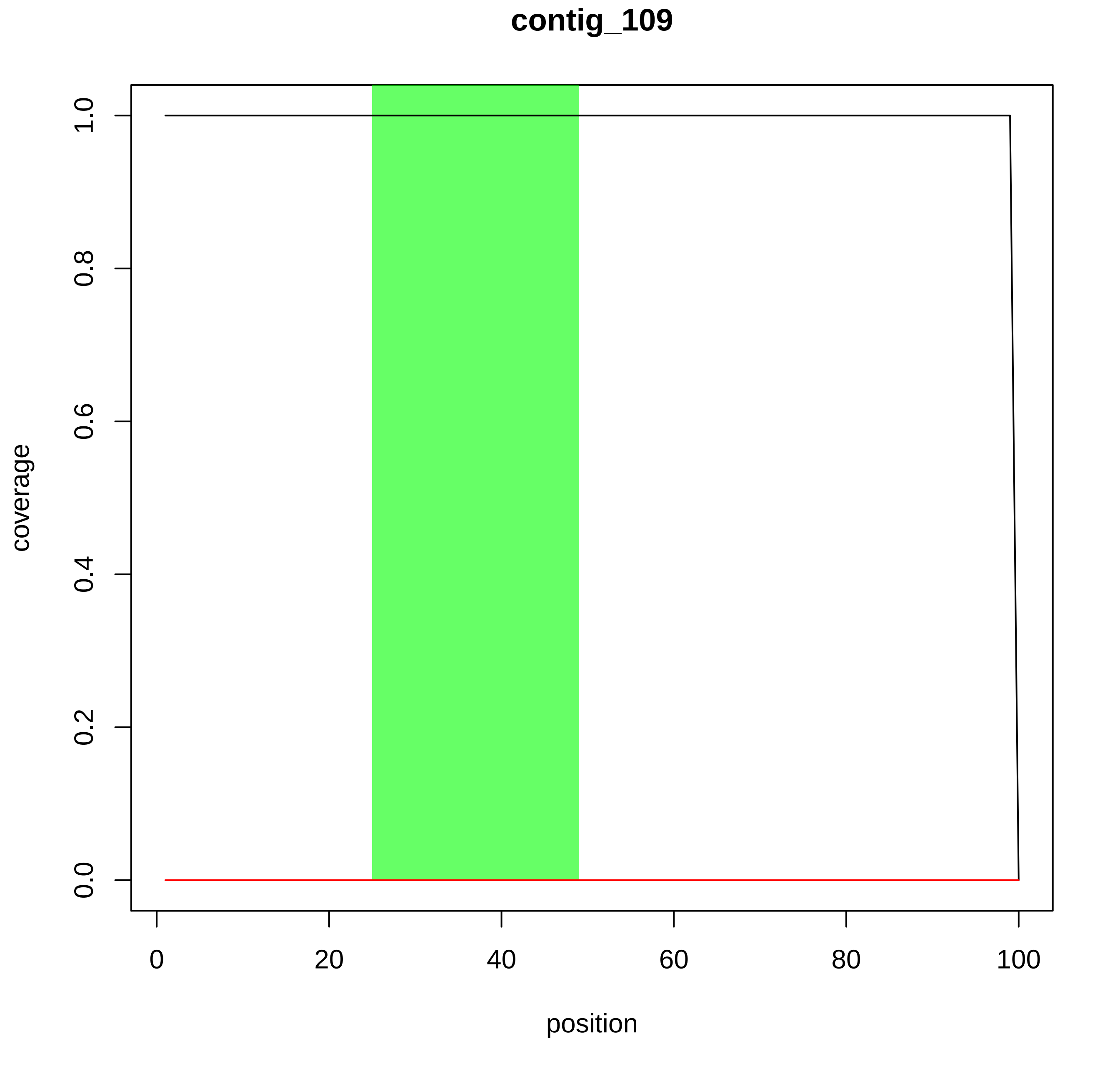
contig_109
coverage
1.0
0.8
0.6
0.4
0.2
0.0
0
20
40
60
80
100
position

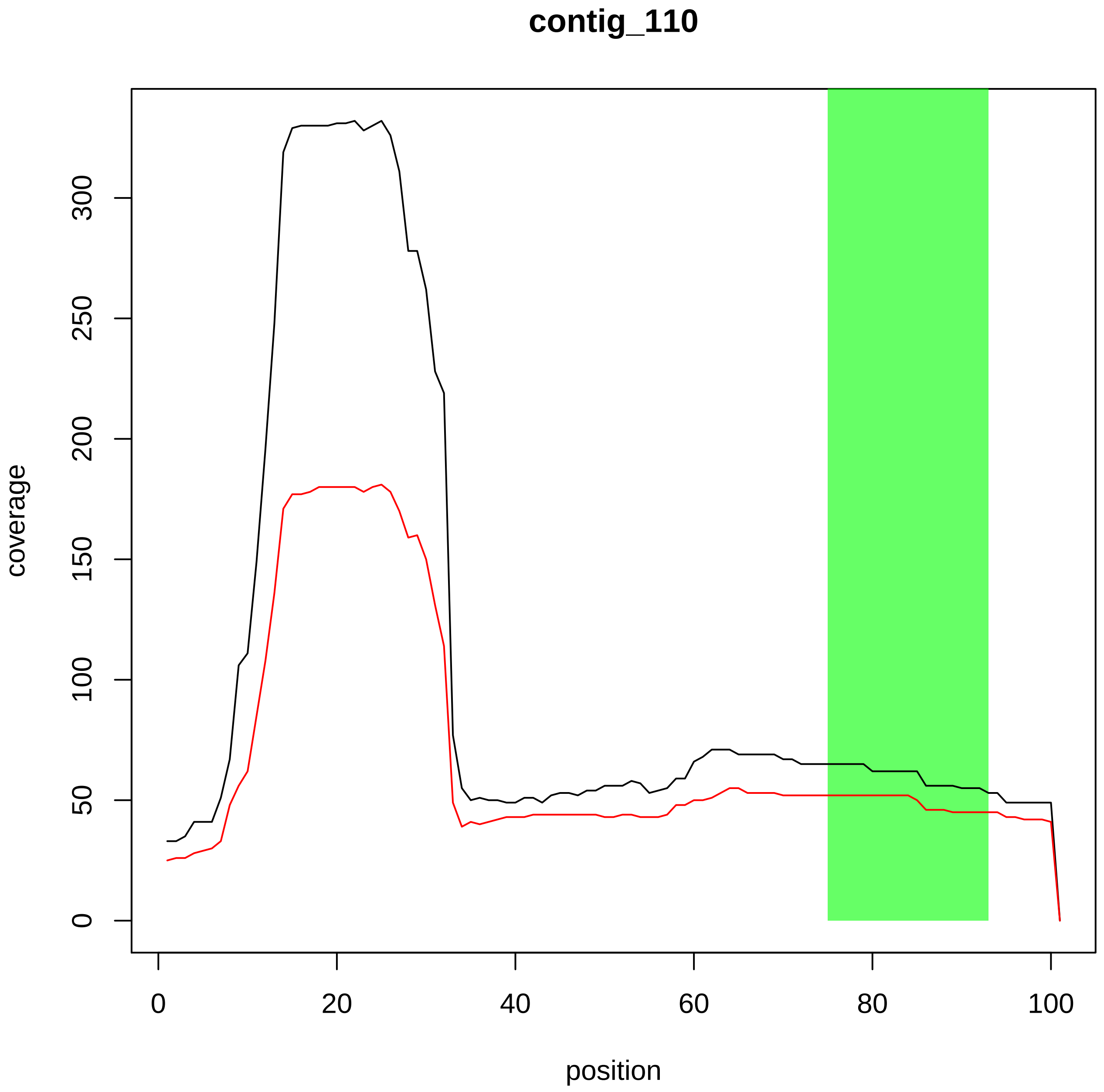
contig_110
coverage
0
50
100
150
200
250
300
position
0
20
40
60
80
100

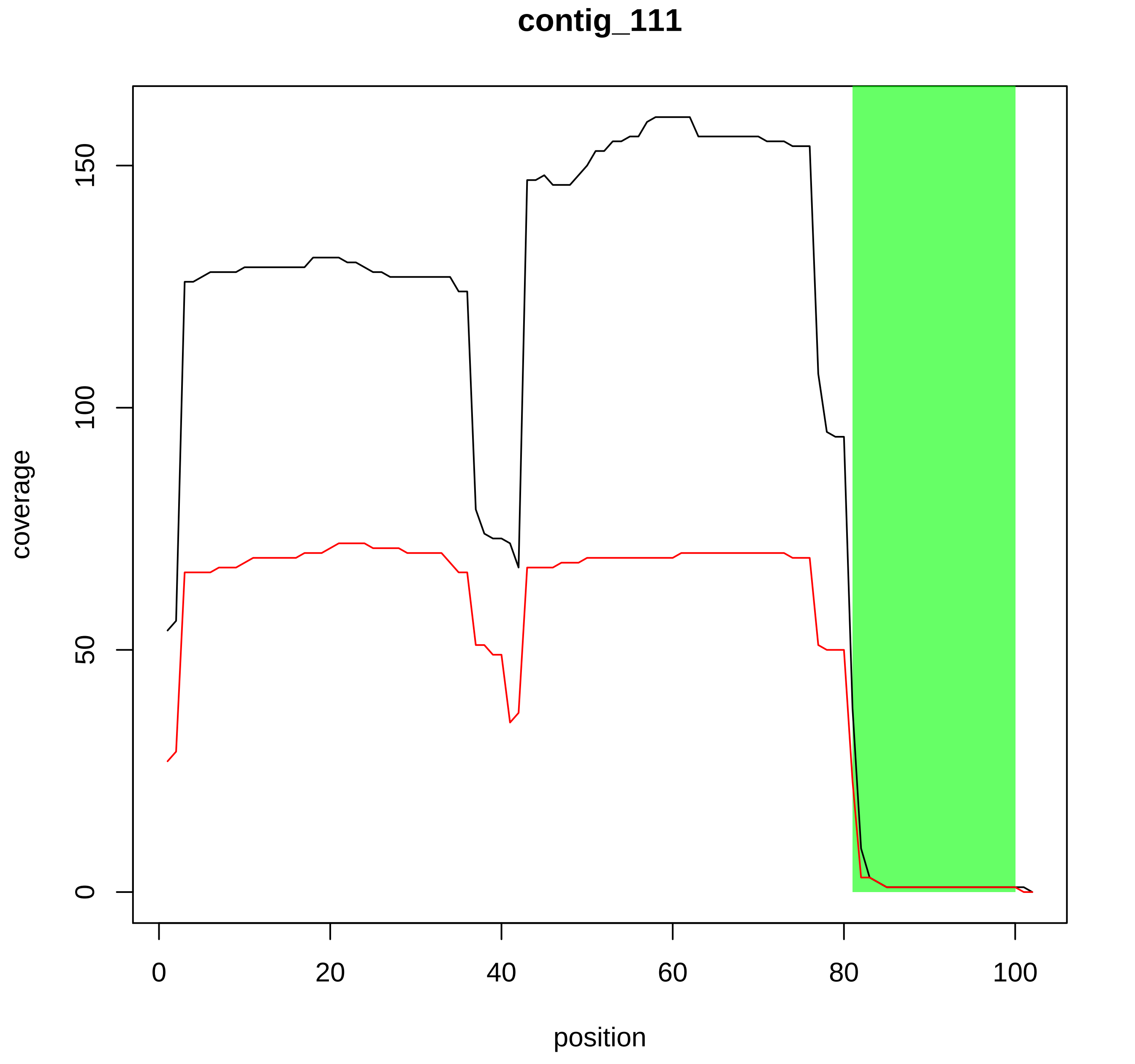

contig_111
coverage
position
0
50
100
150
0
20
40
60
80
100

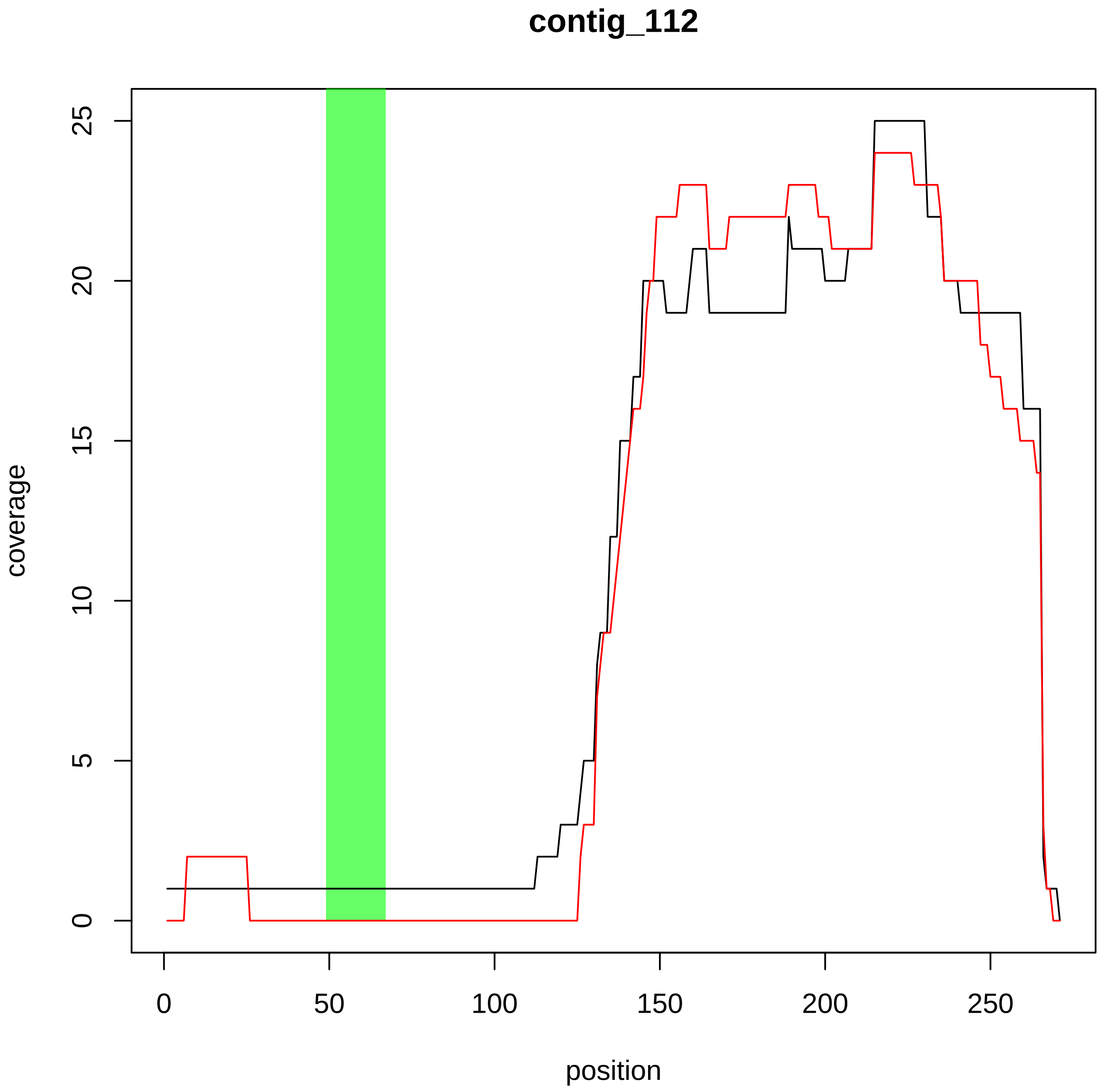
contig_112
coverage
position
0
5
10
15
20
25
0
50
100
150
200
250

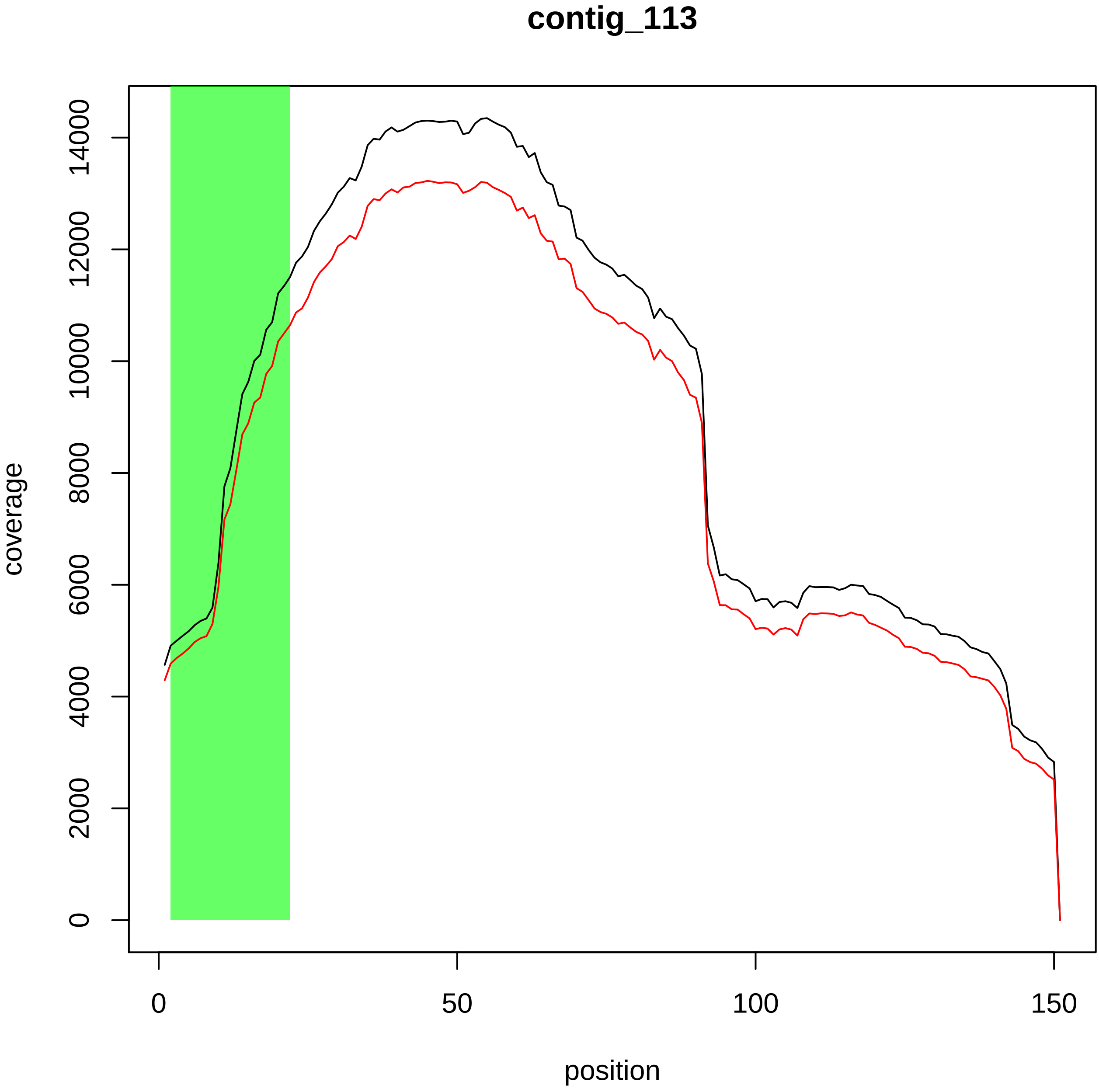
contig_113
coverage
position
0
2000
4000
6000
8000
10000
12000
14000
0
50
100
150

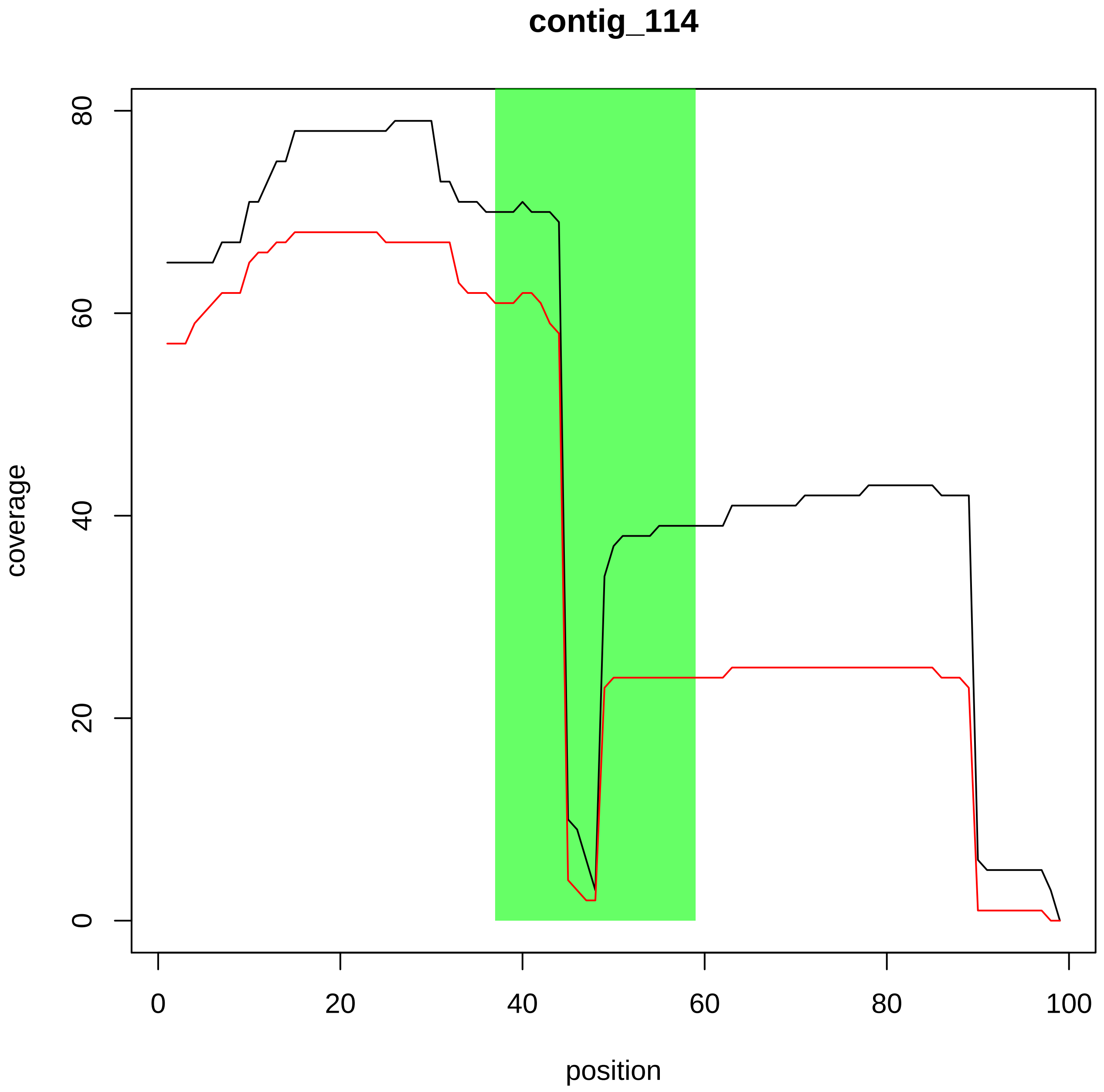
contig_114
coverage
0
20
40
60
80
0
20
40
60
80
100
position

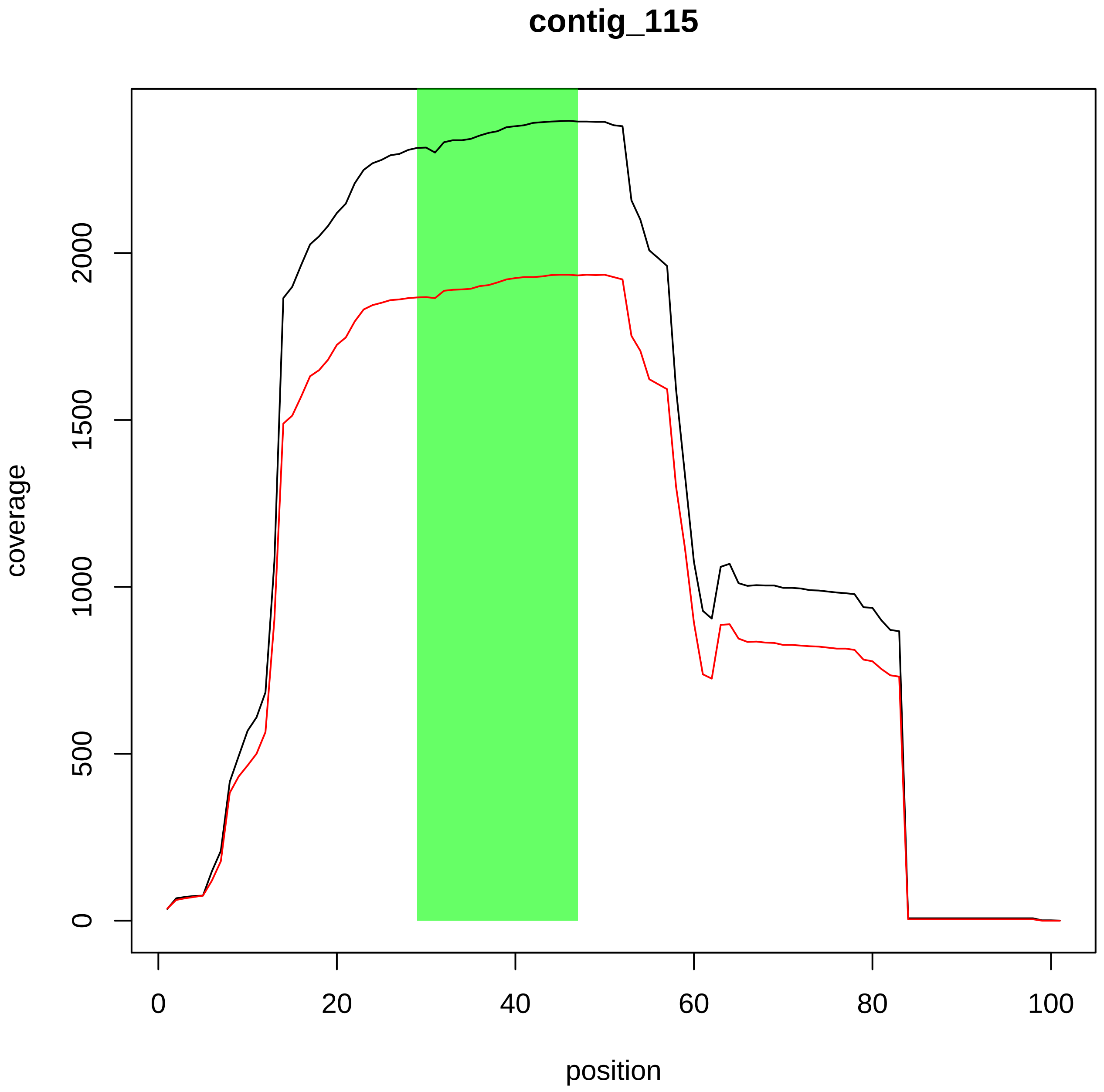

contig_115
coverage
position
0
500
1000
1500
2000
0
20
40
60
80
100

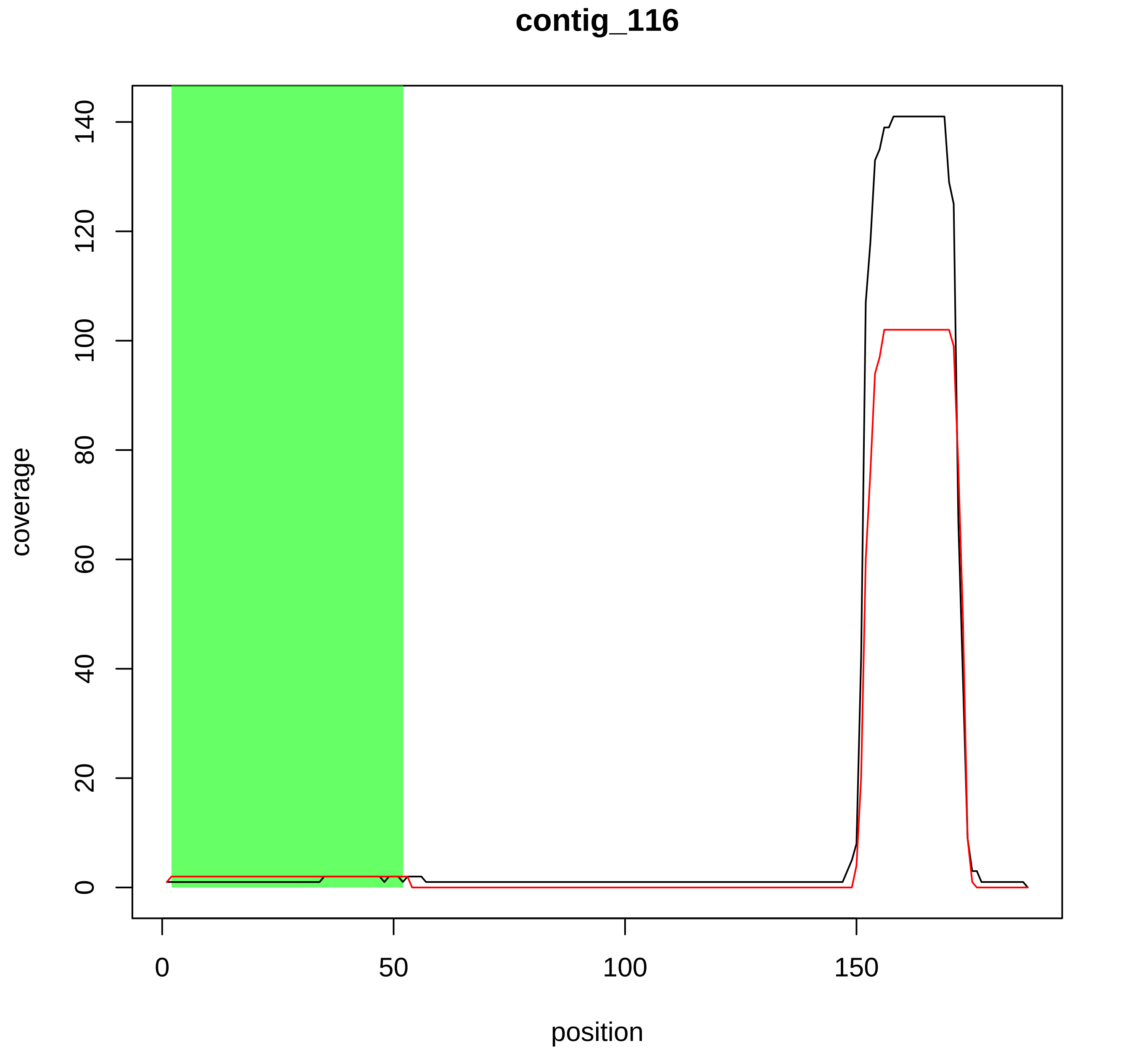
contig_116
coverage
0
20
40
60
80
100
120
140
0
50
100
150
position

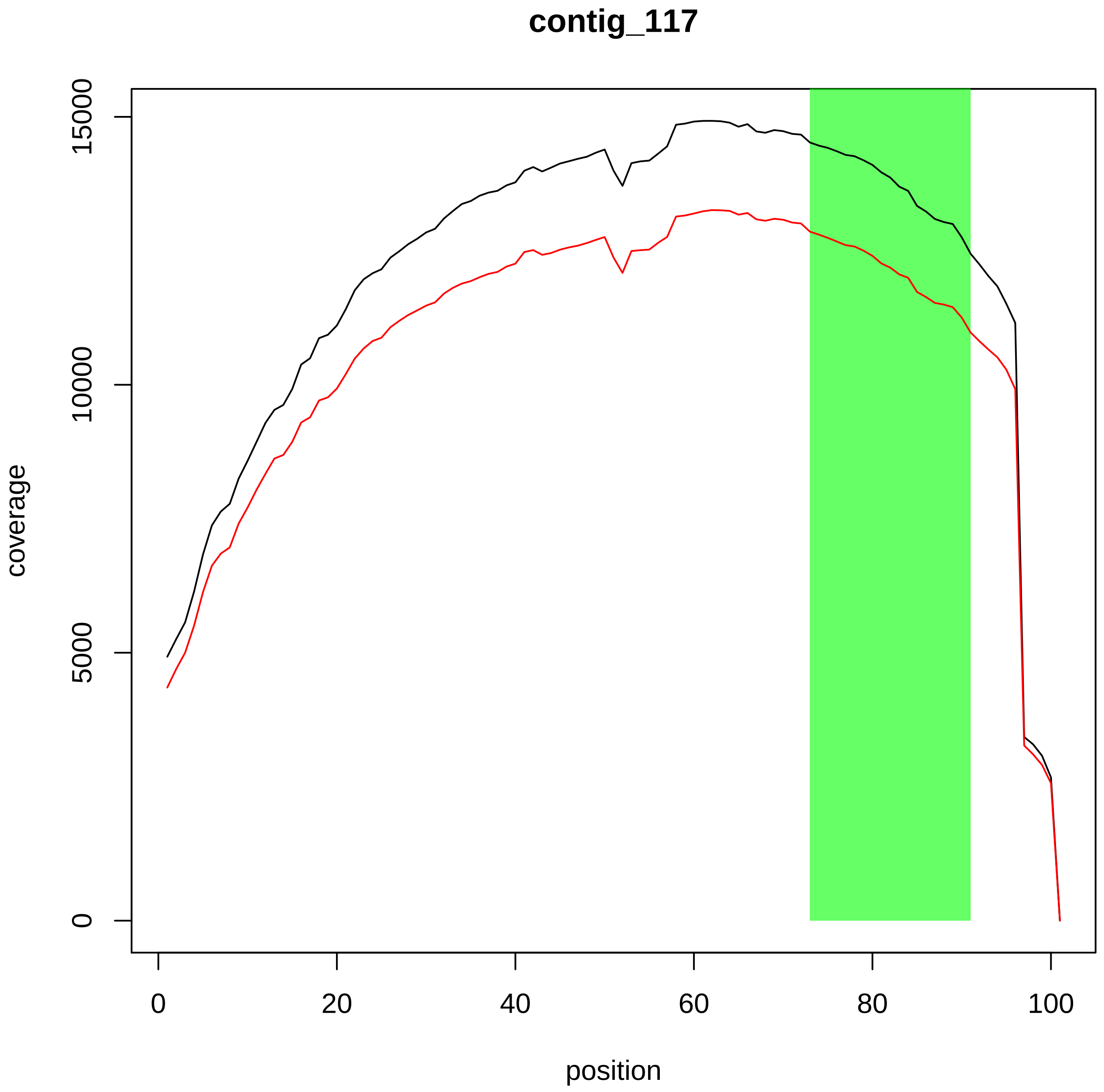
contig_117
coverage
15000
10000
5000
0
0
20
40
60
80
100
position

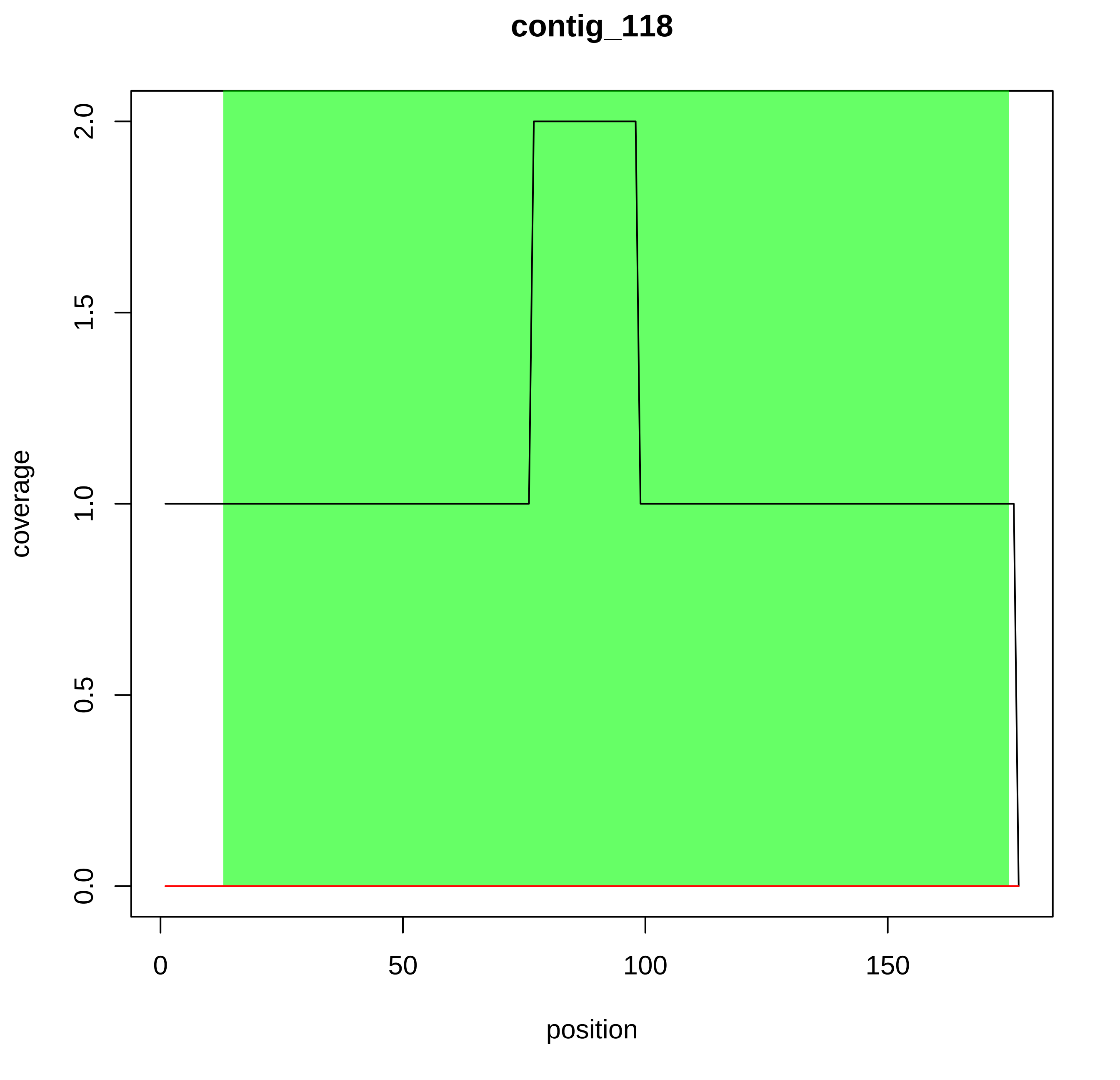
contig_118
coverage
0.0
0.5
1.0
1.5
2.0
0
50
100
150
position

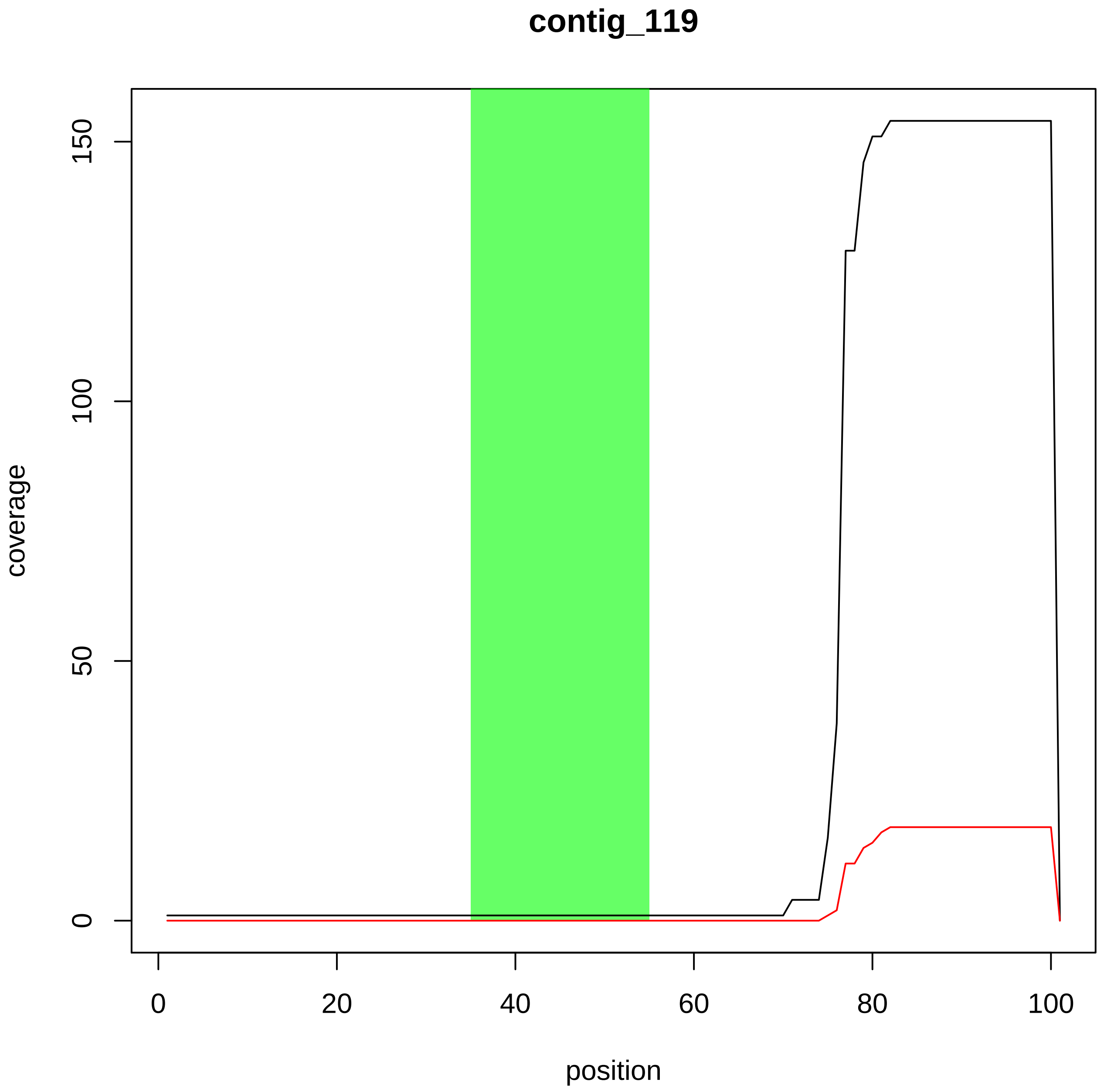

contig_119
coverage
0
50
100
150
0
20
40
60
80
100
position

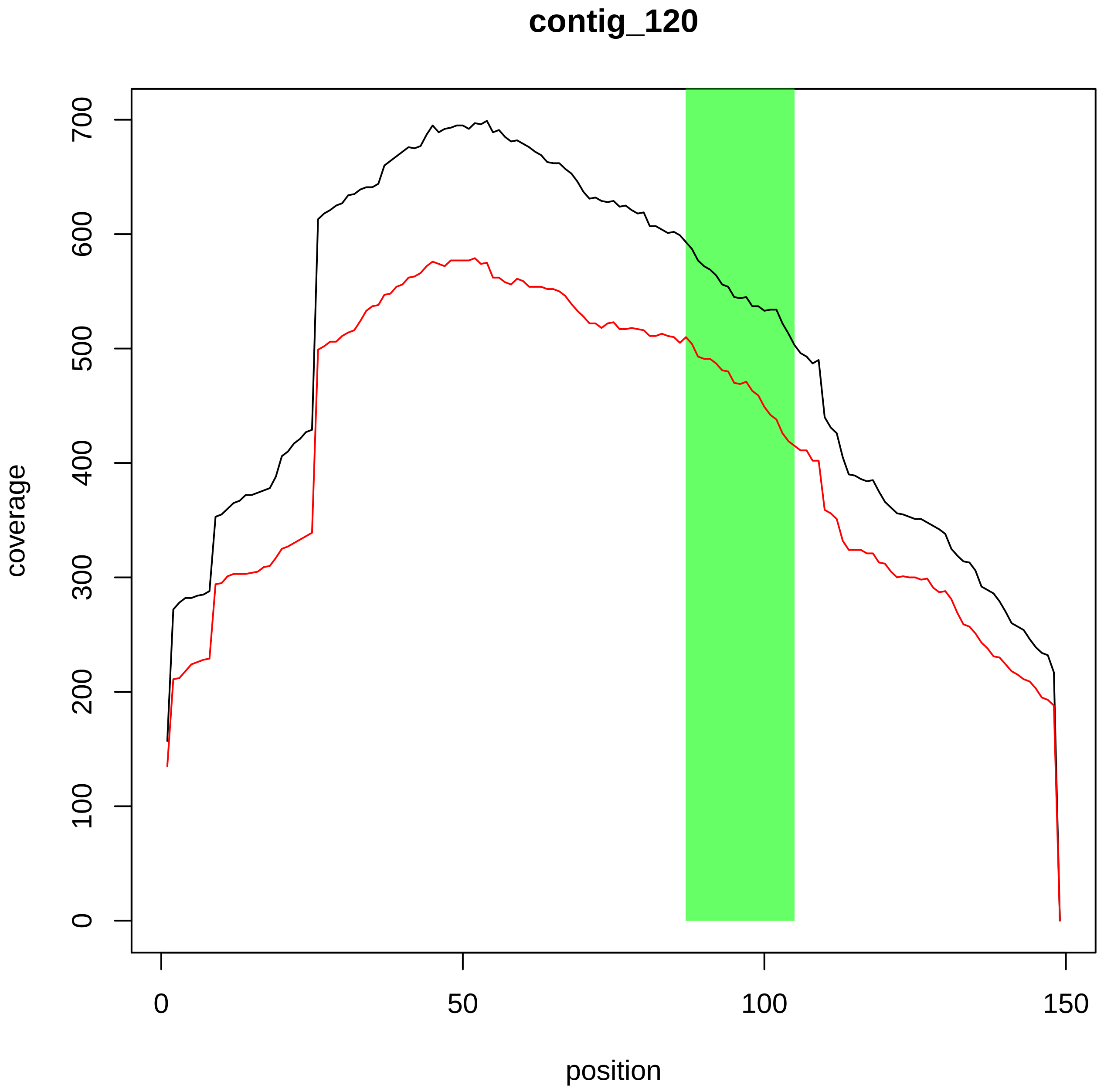
contig_120
coverage
0
100
200
300
400
500
600
700
0
50
100
150
position

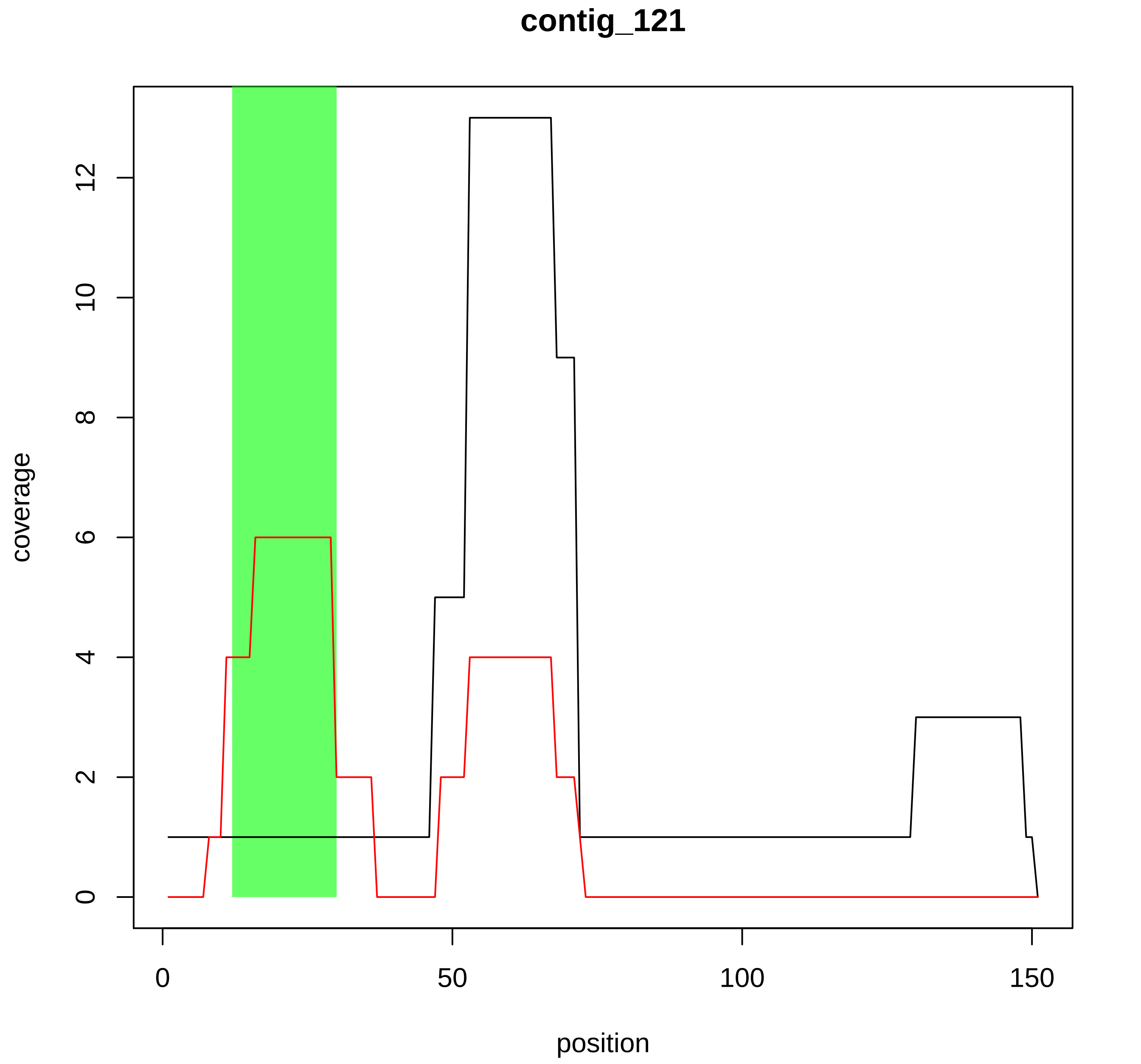

contig_121
coverage
position
0
2
4
6
8
10
12
0
50
100
150

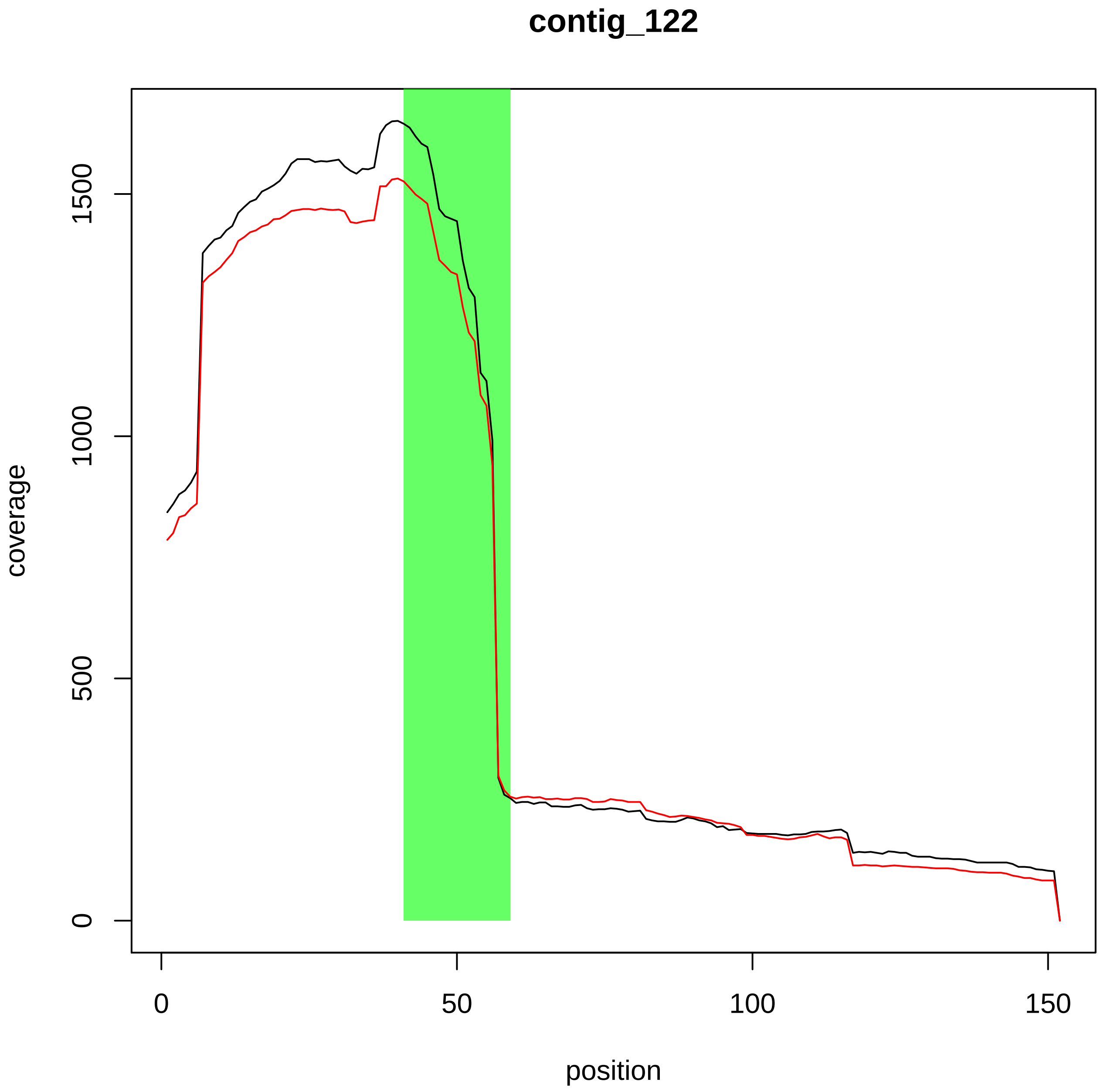

contig_122
coverage
position
0
500
1000
1500
0
50
100
150

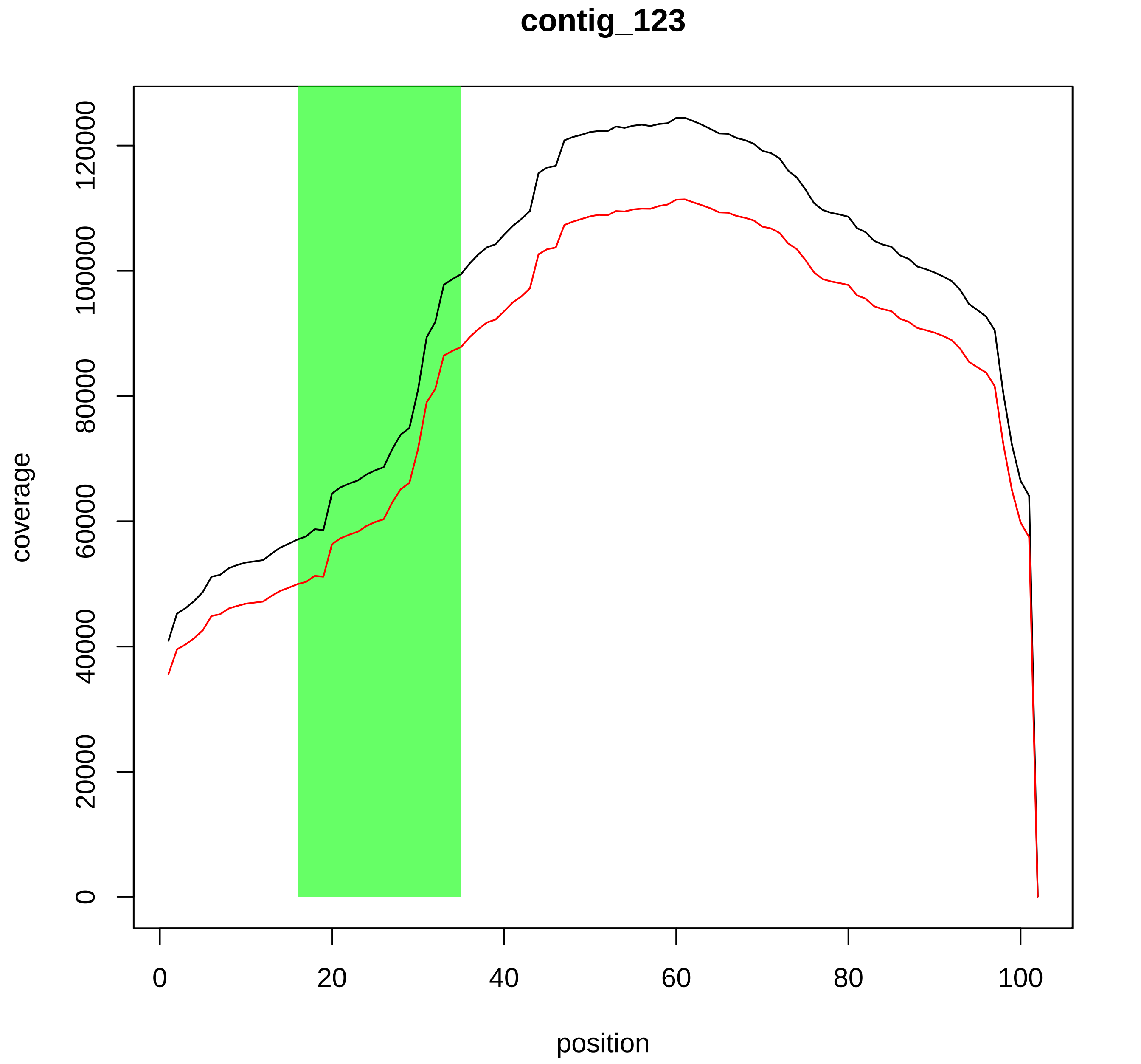
contig_123
coverage
0
20000
40000
60000
80000
100000
120000
0
20
40
60
80
100
position

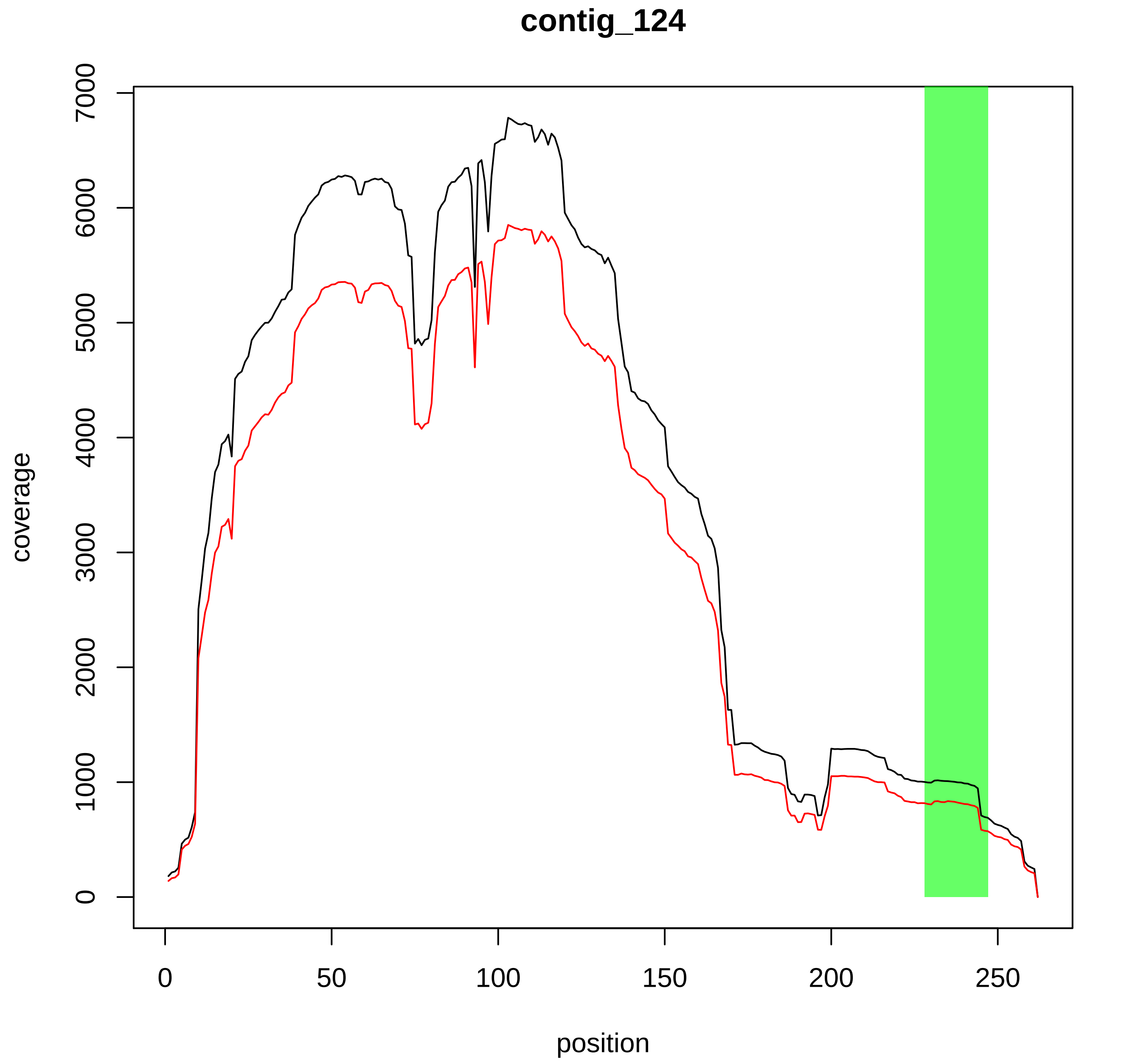
contig_124
coverage
position
0
1000
2000
3000
4000
5000
6000
7000
0
50
100
150
200
250
