# Supplementary material for: No Evidence of Unexpected Transgenic Insertions in T1190 – A Transgenic Apple Used in Rapid Cycle Breeding – Following Whole Genome Sequencing
Source: Front Plant Sci. 2021 Aug 11;12:715737. doi: 10.3389/fpls.2021.715737 (PMC8386123; doi:10.3389/fpls.2021.715737)
Supplement: Supporting Information 3 — Results of BLASTn analyses of contigs that contain sequence fragments, which are identical to the plant transformation vector pHTT602-CaMV35S::BpMADS4. [file Data_Sheet_3.pdf]

Supporting information 3. Results of blastn analyses of contigs that contain sequence fragments, which are identical to the plant transformation vector pHTT602-CaMV35S::BpMADS4.

Contig 81

Contig 81 was built from nine reads. Seven reads originated from experiment 1. Two reads originated from experiment 2. The sequence of this contig has length of 197 bp (supplementary information 1). The contig shows a sequence identity of 95.57% to a region on chromosome 3 of the GDDH13v1.1 reference genome sequence. The fragment, which is identical to the plant transformation vector, has a length of 20 bp (red font) and is identically present in the apple reference genome.

```
      1 AGCCTTTGGGCTGCATCTCAGGTTTGACTCGGGGAGAAAGAGGGGATGGGGACATAAGGG 60
        ||||||||||||||||||||||||||||||||||||||||||||||||||||||||||||
3064018 AGCCTTTGGGCTGCATCTCAGGTTTGACTCGGGGAGAAAGAGGGGATGGGGACATAAGGG 3064077

     61 CTGACAAATTGGAGAGAGGAGACAGCTGGTTGTGCATT------TGGCTGTGGCTGTGGC 120
        ||||||||||||||||||||||||||||||||||  |       ||||||||||||||||
3064078 CTGACAAATTGGAGAGAGGAGACAGCTGGTTGTGGCTGTGGCTGTGGCTGTGGCTGTGGC 3064137

    121 TGTGGCGAGAGGAGAGGAGGGGCACAGAGGGACGGACAGATTGGAGAGAGATGGGAGCTA 180
        ||||||||||||||||||||||||||||||||||||||||||||||||||||||||||||
3064138 TGTGGCGAGAGGAGAGGAGGGGCACAGAGGGACGGACAGATTGGAGAGAGATGGGAGCTA 3064197

    181 CGGGTGATCTTGATGCCGCGTGT 197
        |||||||||||||||||||||||
3064198 CGGGTGATCTTGATGCCGCGTGT 3064220
```

Two context sequences (one at each site of the contig) are available for contig 81. The context sequence at the left end of contig 81 has a length of 90 bp and shows a sequence identity of 100% (90/90 bp) to a region on chromosome 3, which is closely located to the region with sequence identity to the contig sequence.

```
      1 AGTTTATTTACAATATTGCCACTCGAATGCTTTTACCCATCTTTCTCTTTATTTACAAAA 60
        ||||||||||||||||||||||||||||||||||||||||||||||||||||||||||||
3063928 AGTTTATTTACAATATTGCCACTCGAATGCTTTTACCCATCTTTCTCTTTATTTACAAAA 3063987

     61 AAAACCCCTCAGGCTTCAAGCCCGATTTGT 90
        ||||||||||||||||||||||||||||||
3063988 AAAACCCCTCAGGCTTCAAGCCCGATTTGT 3064017
```

The context sequence at the right end of contig 81 has a length of 64 bp and shows a sequence identity of 100% (64/64 bp) to a region of chromosome 3, which is also closely located to the region with sequence identity to the contig sequence.

```
      1 GGGATTTGGTGAGGGGAAGGCATGCGGCTTGCAGAGATGAGGATGAGAAAGGGGGAGGGA 60
        ||||||||||||||||||||||||||||||||||||||||||||||||||||||||||||
3064381 GGGATTTGGTGAGGGGAAGGCATGCGGCTTGCAGAGATGAGGATGAGAAAGGGGGAGGGA 3064440

     61 CAAA 64
        ||||
3064441 CAAA 3064444
```

Contig 92

Contig 92 was built from three reads that originated from experiment 2. The sequence of this contig has length of 272 bp (supplementary information 1). Blast analyses resulted in sequence an identity of 95.71% (134/140 bp) to a sequence of chromosome 3 of the GDDH13v1.1 apple reference genome. The fragment of contig 92, which is identical to the plant transformation vector, has a length of 38 bp, out of which 36 bp (red font) were identically present in this sequence on chromosome 3 of GDDH13v1.1.

```
    133 TGTGGCTGTGGCTGTGGCTGTGGCTGTGGCTGTGGCGAGAGGAGCGGAGGGGCACAGAGG 192
        |||||||||||||||||||||||||||||||||||||||||||| |||||||||||||||
3064108 TGTGGCTGTGGCTGTGGCTGTGGCTGTGGCTGTGGCGAGAGGAGAGGAGGGGCACAGAGG 3064167

    193 GTCGGACAGAGTGGAGAGAGAAGGGAGCTACGGGTGATCTTGAAGCCGCGGGTTGATAAT 252
        | |||||||| |||||||||| ||||||||||||||||||||| |||||| |||||||||
3064168 GACGGACAGATTGGAGAGAGATGGGAGCTACGGGTGATCTTGATGCCGCGTGTTGATAAT 3064227

    253 CGTGGAAAGGTCGGGGTGCG 272
        ||||||||||||||||||||
3064228 CGTGGAAAGGTCGGGGTGCG 3064247
```

Blastn analyses against the *M. ×domestica* v3.0.a1 reference genome showed a sequence identity of 94.37% (134/142 bp) to contig MDC0200755.530. The 38 bp fragment (red font) with sequence identity to the plant transformation vector is identically present in the apple reference genome sequence.

```
Query:  131 GCTGTGGCTGTGGCTGTGGCTGTGGCTGTGGCTGTGGCGAGAGGAGCGGAGGGGCACAGA 190
            |||||||||||||||||||||||||||||||||||||||||||||| |||||||||||||
Sbjct: 5243 GCTGTGGCTGTGGCTGTGGCTGTGGCTGTGGCTGTGGCGAGAGGAGAGGAGGGGCACAGA 5184

Query:  191 GGGTCGGACAGAGTGGAGAGAGAAGGGAGCTACGGGTGATCTTGAAGCCGCGGGTTGATA 250
            ||| | |||||| |||||||||| ||||||||||||||||||||| |||||| |||||||
Sbjct: 5183 GGGACTGACAGATTGGAGAGAGATGGGAGCTACGGGTGATCTTGATGCCGCGTGTTGATA 5124

Query:  251 ATCGTGGAAAGGTCGGGGTGCG 272
            ||||||||||||| ||||||||
Sbjct: 5123 ATCGTGGAAAGGTAGGGGTGCG 5102
```

Blast analyses against the NCBI database failed to identify any sequence identity to other sequences for the first 130 bp of contig 92. This was also the case for the 121 bp context sequence at the left end of this contig.

Contig 98

Contig 98 was built from two reads that occurred in experiment 2. The sequence of this contig has length of 106 bp (supplementary information 1). Contig 98 contains two 20 bp fragments (red font), which are identical to the plant transformation vector. Sequences with high levels of sequence identity were found on all apple chromosomes. The highest level of sequence identity was found on chromosome 13 of the GDDH13v1.1 apple reference genome. A sequence identity of 97.47% (77/79 bp) was found for the region between base 28 and 106. The 20 bp fragment with identity to the plant transformation vector (red font) is identically present in the apple genome sequence.

```
      28 GCTTCCTATTATTATCATTTAATACTAGGAAGCAGAGCGAACAAGAAAGAACCAAGTATG 87
         |||||| ||||||||||||||||| |||||||||||||||||||||||||||||||||||
20584849 GCTTCCCATTATTATCATTTAATAATAGGAAGCAGAGCGAACAAGAAAGAACCAAGTATG 20584908

      88 GGATGCCCATAACAACAGG 106
         |||||||||||||||||||
20584909 GGATGCCCATAACAACAGG 20584927
```

A high level of sequence identity was also found on chromosome 13 for the first 41 bp of contig 98. The level of sequence identity was 100% (41/41 bp). The 20 bp fragment with identity to the plant transformation vector (red font) is identically present in the apple genome sequence.

```
       1 CATACTTGGTTCTTTCTTGTTCGCTCTGCTTCCTATTATTA 41
         |||||||||||||||||||||||||||||||||||||||||
20584908 CATACTTGGTTCTTTCTTGTTCGCTCTGCTTCCTATTATTA 20584868
```

One context sequence at the right end of contig 98 is available, which has a length of 151 bp. A high level of sequence identity was found between this context sequence an a region on chromosome 13 of the GDDH13v1.1 reference genome sequence. This sequence is closely located to the region with sequence identity to the remaining contig.

```
       1 GCAAGTGCGGGCAATGACTGAAGCTTGAAGAACGAATCTGCCCTTCTCTTCCAGAATAAT 60
         |||||||||||||||||| |||||  ||||||||||||||||||||||||||||||||||
20585046 GCAAGTGCGGGCAATGACCGAAGCCCGAAGAACGAATCTGCCCTTCTCTTCCAGAATAAT 20585105

      61 AATATAGCTACTCAACAACTGAGGGAGGAGTTAGCTGAATTAAGGAGGACGGTGGCTCAT 120
         |||||||||||||||||||||||||| |||||| |||||||||||||||||||||||| |
20585106 AATATAGCTACTCAACAACTGAGGGATGAGTTAACTGAATTAAGGAGGACGGTGGCTCGT 20585165

     121 AATGCTTAACCACAAGCTGGTCCAGTGTTCA 151
         |||||| |||||||||||  |||||||||||
20585166 AATGCTCAACCACAAGCTCATCCAGTGTTCA 20585196
```

Contig 119

Contig 119 was built from two reads that occurred in experiment 1. The sequence of this contig has a length of 100 bp (supplementary information 1). Sequences with high levels of sequence identity were found on chromosomes 13, 16, 14, 6, 17, and 9 of the GDDH13v1.1 apple reference genome. The highest level of sequence identity was found on chromosome 13. A sequence identity of 100% (77/77 bp) was found for the first 77 bp of this contig. Contig 119 contains one fragment with a length of 22 bp (red font), which is identical to the plant transformation vector. This fragment is identically present in the apple reference genome.

```
      1 GAATCTGTGGCGTACTCAAGGAGCTTCCCCTTGTTGGAGAAGACAATCAAAGCAACCTGA 60
        ||||||||||||||||||||||||||||||||||||||||||||||||||||||||||||
4128876 GAATCTGTGGCGTACTCAAGGAGCTTCCCCTTGTTGGAGAAGACAATCAAAGCAACCTGA 4128935

     61 GCATCACACAAGACAGA 77
        |||||||||||||||||
4128936 GCATCACACAAGACAGA 4128952
```

One context sequence at the left end of contig 119 is available, which has a length of 100 bp. A high level of sequence identity (100%, 100/100 bp) was found between this context sequence and a region on chromosome 13 of the GDDH13v1.1 reference genome sequence. This sequence is closely located to the region with sequence identity to the remaining contig.

```
      1 GGTATATGAAATTTTTTAGCTAGGTTGAGCTCGATTTCTTAGGCGGCGTTTCTAAATCCA 60
        ||||||||||||||||||||||||||||||||||||||||||||||||||||||||||||
4128677 GGTATATGAAATTTTTTAGCTAGGTTGAGCTCGATTTCTTAGGCGGCGTTTCTAAATCCA 4128736

     61 TTTACTTTCTTACAAATACAAATTTCTAGGTTTCTCGAAT 100
        ||||||||||||||||||||||||||||||||||||||||
4128737 TTTACTTTCTTACAAATACAAATTTCTAGGTTTCTCGAAT 4128776
```
