# Supplementary material for: No Evidence of Unexpected Transgenic Insertions in T1190 – A Transgenic Apple Used in Rapid Cycle Breeding – Following Whole Genome Sequencing
Source: Front Plant Sci. 2021 Aug 11;12:715737. doi: 10.3389/fpls.2021.715737 (PMC8386123; doi:10.3389/fpls.2021.715737)
Supplement: Supporting Information 4 — Contigs underrespresented in sequencing data. [file Data_Sheet_4.pdf]

Supporting information 4. Contigs underrepresented in sequencing data

				Context		Identity to Vector		Identity to other sequences		
Contig	bp	Reads[1]	FVI	Left	Right	bp	Position	Genebank[2]	bp	Sequence information
94	99	2	1	0	0	96	8,997…9,092	CP062969.1	96/99	*E. coli*
96	211	2	3	0	0	81<br>33<br>64	9,212…9,292<br>9,305…9,338<br>9,340…9,404	MT636814.1	198/211	*Bacillus* sp.
99	94	2	1	0	1	43	2,626…2,668	CP047586.1<br>AM946177.1	94/94	*Corynebacterium pseudotuberculosis*, *Fusarium verticillioides*
100	101	1	1	0	0	58	2,978…3,036	CP052879.1<br>AB293446.1	101/101	*E. coli*, *Saccharomyces cerevisiae*
104	151	2	1	1	0	21	5,948…5,968	XM_004341709.1	60/61<br>115/120[3]	*Acanthamoeba castellanii*
109	99	1	1	0	0	26	9,988…10,013	LT963440.1<br>AB225553.1	75/75	*Staphylococcus cohnii*, *Aspergillus oryzae*
118	176	2	1	0	0	163	2,978…3,141	CP052879.1<br>AB293446.1	176/176	E. coli, *Saccharomyces cerevisiae*

[1]All contigs of Table 2 were only found in the sequence data of experiment 1, except of contig 104. This contig was only found in the sequencing data of experiment 2. None of these contigs was present in the data of both sequencing experiments.
[2]Most contigs showed identical levels of sequence identity to several genebank accessions. The accession numbers given in this table are selected examples.
bp, base pairs; FVI, number of fragments within the contig sequence with sequence identity to the plant transformation vector pHTT602-CaMV35S::BpMADS4
[3]Context sequence
